# Ni-catalyzed arylation of alkynes with organoboronic acids and aldehydes to access stereodefined allylic alcohols†

**DOI:** 10.1039/d2sc05894d

**Published:** 2023-01-20

**Authors:** Si-Chen Tao, Fan-Cheng Meng, Tie Wang, Yan-Long Zheng

**Affiliations:** a Tianjin Key Laboratory of Drug Targeting and Bioimaging, Life and Health Intelligent Research Institute, Tianjin University of Technology Tianjin 300384 P. R. China ylzheng@email.tjut.edu.cn wangtie@email.tjut.edu.cn

## Abstract

A new, efficient and practical method for the three-component arylative coupling of aldehydes, alkynes and arylboronic acids has been developed through nickel catalysis. This transformation provides diverse *Z*-selective tetrasubstituted allylic alcohols without the use of any aggressive oragnometallic nucleophiles or reductants. Moreover, benzylalcohols are viable coupling partners *via* oxidation state manipulation and arylative coupling in one single catalytic cycle. This reaction features a direct and flexible approach for the preparation of stereodefined arylated allylic alcohols with broad substrate scope under mild conditions. The utility of this protocol is demonstrated through the synthesis of diverse biologically active molecular derivatives.

## Abstract 2

A direct, efficient and practical method for the three-component arylative coupling of aldehydes (or the corresponding alcohols), alkynes and arylboronic acids has been developed through nickel catalysis.

## Graphical abstract

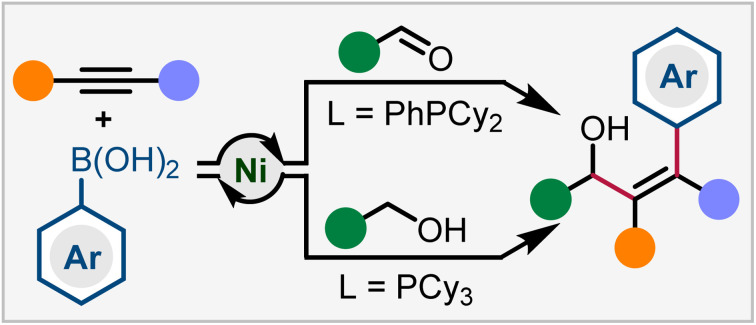

## Introduction

**Scheme 1:**
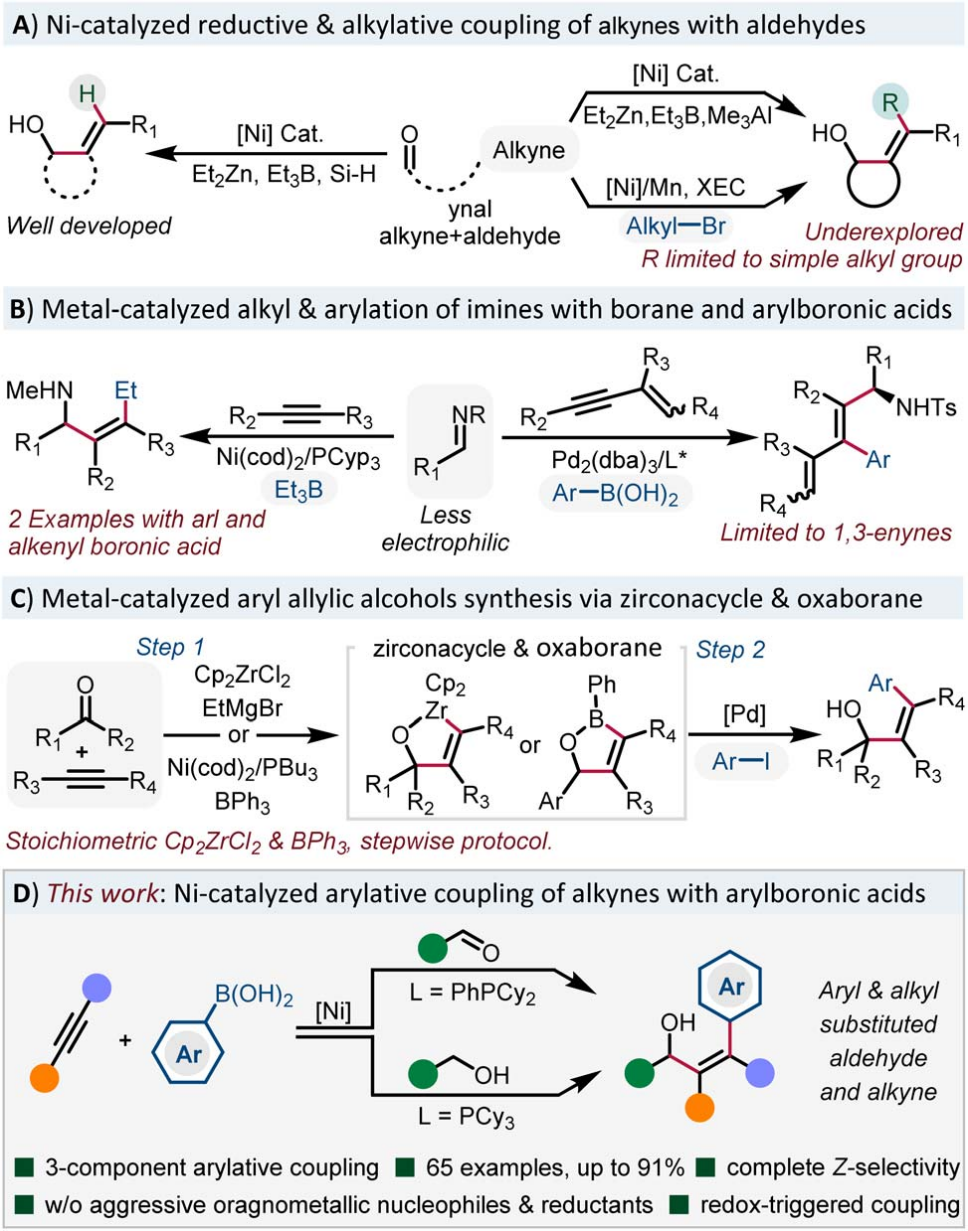
Metal-catalyzed reductive, alkylative and arylative coupling reactions based on oxa(aza)metalacycles.

All-carbon tetrasubstituted alkenes are the core structures in diverse biologically related molecules.^1^ As an important subset of alkenes, allylic alcohols represent an important and versatile building blocks in chemical synthesis, as they offer opportunities for further transformations to install diverse functional groups.^2^ Previous approaches for the synthesis of such compounds mainly relied on the nucleophilic addition to carbonyl compounds with preformed or *in situ* generated alkenylmetal reagents,^3^ which suffers from stoichiometric metallic waste and multistep manipulation. In the past two decades, the pioneering studies of Montgomery,^4^ Jamison^5^ and Ogoshi^6^ groups have demonstrated the low valent Ni(0) catalyzed reductive coupling of readily available alkynes with C=O π-bonds to form allylic alcohols using dialkylzinc,^7^ trialkylborane,^8^ as well as organosilane^9^ (Scheme 1A). The role of these alkylmetals serving as hydrides to form trisubstituted allylic alcohols is well developed, however, direct delivery of an alkyl group to the alkyne is underdeveloped. Simple methyl or ethyl groups are incorporated in most cases and more functionalized alkyl groups are still hampered by this strategy.^10^ Recently, the Montgomery group disclosed the cross-electrophile coupling of oxanickelacycles with diverse functionalized electrophiles to address these limitations.^11^ Although the electrophiles are limited to sp^3^-hybridized alkyl bromides, it still represents a major advance in alkylative coupling reactions. However, the arylative coupling reactions of alkynes with C=X bonds are still rare.

In 2003, Jamison reported an alkylative coupling of alkynes with imines and triethylborane to form tetrasubstituted allylamines (Scheme 1B).^12^ It's worth noting that the practicability of the reaction is significantly improved with phenyl and styrylboronic acids. Recently, Chen and co-workers reported a Pd(0)-catalyzed three-component coupling of 1,3-enynes, imines and arylboronic acids to form enantioenriched arylated allylamines.^13^ However, simple alkynes showed no reactivity. With respect to the synthesis of stereodefined arylated allylic alcohols (Scheme 1C), the Liu group developed a stepwise protocol, taking advantage of Pd-catalyzed coupling of aryl iodides with stable oxazirconacycles.^14^ Very recently, the Stanley group reported a Ni(0)-catalyzed dearylative cyclocondensation reaction from aldehydes, alkynes and triphenylboranes to form oxaboranes,^15^ which can be further transformed into tetrasubstituted allylic alcohols. However, the use of stoichiometric amounts of Cp_2_ZrCl_2_ and triphenylborane in these stepwise procedures hampers its further development. Thus, to develop a mild and practical methodology to access arylated tetrasubstituted allylic alcohols without the requirement of reactive main group organometallic reagents or reductants is highly desirable.

Inspired by the Ni-catalyzed functionalization of arynes^16^ and 1,3-dienes^17^ with stable and easily accessible B_2_(pin)_2_ and organoboronic acids, we envisioned that the use of arylboronic acids^18^ instead of sensitive organometallic reagents would significantly facilitate the synthesis of arylated tetrasubstituted allylic alcohols.^19^ However, the main challenge associated with this system is how to suppress the 1,2-addition chemistry with more electrophilic aldehydes.^20^ Herein, we report a Ni-catalyzed three-component arylative coupling of aldehydes and alkynes with organoboronic acids under mild conditions. Moreover, we can also merge the oxidation state manipulation of alcohols with the arylative cross-coupling reaction in one pot *via* a redox triggered process (Scheme 1D). This general and practical approach furnished the rapid access to diverse stereodefined arylated allylic alcohols.

## Results and discussion

**Scheme 2:**
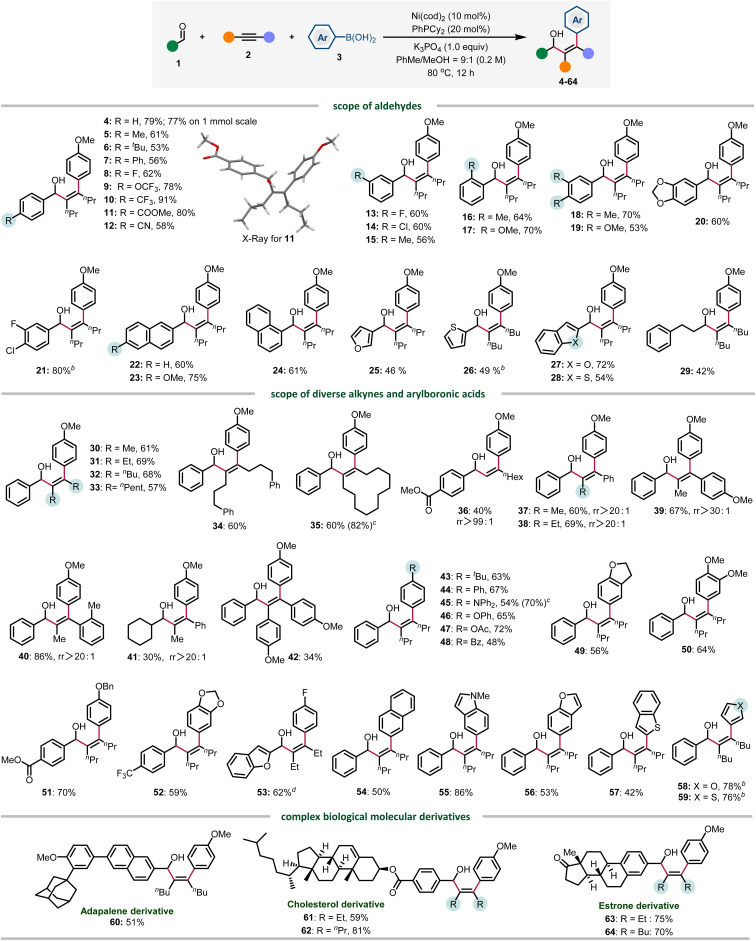
Reaction scope of aldehydes, alkynes, and aryl boronic acids. ^*a*^ Reaction conditions: 1 (0.2 mmol), 2 (0.3 mmol), 3 (0.4 mmol), Ni(cod)_2_ (10 mol%), PhPCy_2_ (20 mol%), K_3_PO_4_ (0.2 mmol), toluene/methanol = 9 : 1 (V/V, 0.2 M), 80 °C for 12 h. Isolated yields are reported. ^*b*^ PCy_3_ instead of PhPCy_2_. ^*c* 1^H NMR yield in the bracket. ^*d*^ Ni(cod)(DQ) instead of Ni(cod)_2_.

Our investigation began by the evaluation of reaction parameters with benzaldehyde (1a), 4-octyne (2a) and *p*-anisylboronic acid (3a). During our initial studies, the desired arylated product (4) was often formed alongside with the ethanol mediated reductive coupling product^21^ (4a) and 1,2-addition product^20*a*^ (4b) (see Table S1 in the ESI† for details). After optimization, the combination of Ni(cod)_2_ (10 mol%) and PhPCy_2_ (20 mol%) as the catalyst, with the addition of potassium phosphate (1 equiv.) as the base enabled the formation of 4 in 71% yield using a toluene/ethanol co-solvent (Table 1, entry 1). The choice of the PhPCy_2_ ligand is particularly important. Structurally similar and diverse monodentate phosphines (entries 2–7) either showed diminished reactivity or promoted the formation of 4b, while bidentate phosphine ligands totally shut down the reaction (entries 8 and 9). Two chiral monophosphines were also tested (entries 10 and 11),^22^ only (*S*)-NMDPP showed moderate reactivity.^23^ Similarly, KF, Na_3_PO_4_ and other carbonate salts were inferior for the reaction (entries 12–14). We also tested π-accepting and electron-deficient ligands (entries 15 and 16) to accelerate the reductive elimination step; however, only methyl methacrylate was found to be effective, with a slight sacrifice of selectivity (entry 16). The solvent effect was also investigated (entries 17 and 18). Benzene gave comparable yields while ethereal solvents were inferior. After evaluating the co-solvents, we found that methanol is superior to ethanol and isopropanol (entries 19 and 20) and gave 4 in 82% yield. We speculated that methanol may play a critical role in the cleavage of the oxanickelacycle or the transmetallation step (Scheme 2).

**Table 1:** Optimization of the arylative coupling reaction of aldehydes with alkenes^a^

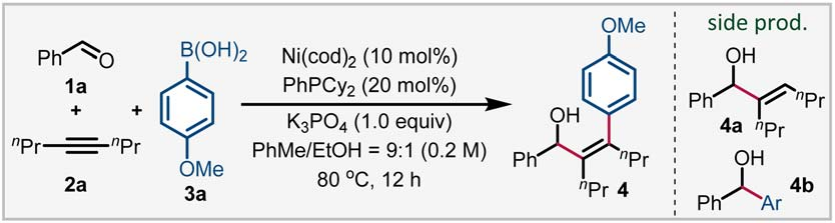				
Entry	Deviations	Yield^b^ (%)		
		4	4a	4b
1	None	71	8	6
2	PCy_3_ instead of PhPCy_2_	46	15	7
3	PCyp_3_ instead of PhPCy_2_	61	11	3
4	L = Ph_2_PCy, Ph_2_PMe, ^me^CgPPh	11–37	<8	22–40
5	L1 instead of PhPCy_2_	7	3	60
6	CyAPhos instead of PhPCy_2_	56	7	5
7	XPhos and CyJohnPhos as ligands	9–15	8–12	40–44
8	dcype instead of PhPCy_2_	7	n.d.	52
9	dppe and dppp as ligands	0	0	46–56
10	(*R*)-AntPhos as the ligand	7	3	55
11	(*S*)-NMDPP as the ligand	53^c^	10	36
12	KF instead of K_3_PO_4_	54	6	8
13	Na_3_PO_4_ instead of K_3_PO_4_	10	n.d.	n.d.
14	K_2_CO_3_, Cs_2_CO_3_, and Rb_2_CO_3_ as bases	42–53	10	12–16
15^d^	w/DMFU, P(OPh)_3_ or TBA	13–47	n.d.	6–26
16^d^	w/methyl methacrylate	74	12	2
17	Benzene/EtOH = 9 : 1 as the solvent	71	8	6
18	Dioxane, THF, and CPME as solvents	41–46	<6	8–15
19	PhMe/^i^PrOH = 9 : 1	58	6	10
20	PhMe/MeOH = 9 : 1	82(79)^e^	8	5
21	w/o MeOH	42	7	6

a Reaction conditions: 1a (0.2 mmol), 2a (0.3 mmol), 3a (0.4 mmol) at 0.2 M concentration.

b Yields were determined by ^1^H NMR spectroscopy using 1,1,2,2-tetrachloroethane as an internal standard.

c 2.4% ee was observed.

d 20 mmol% additive.

e Isolation yield. DMFU = dimethylfumarate, TBA = *tert*-butyl acrylate. PCyp_3_: tricyclopentylphosphine, ^me^CgPPh: 1,3,5,7-tetramethyl-8-phenyl-2,4,6-trioxa-8-phosphaadamantane, L1: ((2,4,6-tri-isopropyl)phenyl)di-cyclohexyl phosphine, CyAPhos: dicyclohexyl(4-(*N*,*N*-dimethylamino)phenyl)phosphine, dcype: 1,2-bis(dicyclohexylphosphanyl)ethane, (*R*)-AntPhos: (*R*)-4-(anthracen-9-yl)-3-(*tert*-butyl)-2,3-dihydrobenzo[*d*][1,3]oxaphosphole, (*S*)-NMDPP: (*S*)-(+)-neomenthyldiphenylphosphine.

Under the optimized conditions, the reaction scope of this three-component transformation was investigated. Firstly, the aldehyde was evaluated. Benzaldehydes bearing electron neutral (4–9) and electron-withdrawing groups (10–12) at the *para*-position were well tolerated, providing products from 53 to 91% yield. Various substitution patterns were also evaluated, *meta* (13–15), sterically hindered *ortho*-substituted (16 and 17) and disubstituted (18 and 20) all gave the arylated products in good yields. Notably, the C–Cl bond also survived in 21, providing a site for further functionalization. The π-extended naphthyl aldehydes showed good reactivity, affording 22–24 in 60–75% yield. Heteroaromatic aldehydes were also tolerated, providing 25–28 in 46–72% yields. Finally, 3-phenylpropanal gave 29 in moderate yield, presumably due to the competing aldehyde oligomerization.

We also evaluated the scope of alkynes and arylboronic acids under optimized conditions. Firstly, various symmetrical alkyl alkynes (30–34) and cyclododecyne (35) all reacted well to deliver corresponding products in good yields. Notably, the terminal alkyne was also compatible, generating 36 in 40% yields with excellent regioselectivity (rr >99 : 1). The reactivity of diverse electronically biased alkyl aryl alkynes is also good, providing 37–41 in a highly regioselective manner (rr >20 : 1). To our disappointment, the reactivity of diaryl alkynes is relatively lower in most cases, and 42 was obtained only in 34% yield. Next, the scope of arylboronic acids was investigated. Arylboronic acids bearing an alkyl group (43), electron-donating substituents (43–47) including Ph, NPh_2_, and OAc, electron-withdrawing groups such as benzoyl and fluoro (48 and 53), and other ethereal substituents (49–52) were similarly efficient. 2-Naphthyl boronic acid was also compatible, albeit with moderate reactivity (54). Notably, heteroarylboronic acids were also found to be competent coupling partners, and indole, benzofuran, benzothiophene, and furan boronic acids all reacted smoothly to afford 55–59 in 42–86% yields. Finally, adapalene, cholesterol and estrone derivatives were also amenable to this arylative coupling reaction, delivering diverse functionalized allylic alcohols in high yields (60–64). Aside from the successful examples, we also encountered several reactions that gave low or no yield of products, which are proven to be challenging in this three-component transformation. These scope limitations can be found in Fig. S1 in the ESI (Section 5.2).†

To improve the practicality of the arylative coupling reaction, 4 substrate scope examples (4, 29, 53 and 58) were selected to evaluate the performance of air-stable Ni-precatalysts in order to setup the reaction outside the glovebox (Table 2). Most Ni(ii) salts and Ni(TMEDA)(*o*-Tol)Cl^24^ were ineffective (Table 2, entries 2 and 3) except for Jamison's (PhPCy_2_)_2_Ni(*o*-Tol)Cl^25^ precatalyst (entry 4), which showed moderate reactivity compared with Ni(cod)_2_. For the air-stable Ni(0) precursors, Engle's Ni(cod)(DQ)^26^ precatalyst provided comparable or higher yields compared with Ni(cod)_2_ (entry 5), while Cornella's 16-electron Ni(0)–stilbene complex^27^ only gave appreciable yields (entry 6). This preliminary survey indicates that Ni(cod)(DQ) is a promising alternative for glovebox-free operations.

**Table 2:** The performance of air-stable Ni-precatalysts *versus* Ni(cod)_2_^a^

Entry	Ni-precat (10 mol%)	4	29	53	58
1	Ni(cod)_2_	82%	44%	21%	75%
2	NiBr_2_, Ni(OTf)_2_	<5%	—	—	—
3	Ni(TMEDA)(*o*-Tol)Cl	8%	—	—	28%
4^b^	(PhPCy_2_)_2_Ni(*o*-Tol)Cl	50%	24%	34%	61%
5	Ni(cod)(DQ)	88%^c^	32%	67%	66%
6	Ni(^*t*^Bustb)_3_	16%	17%	33%	31%
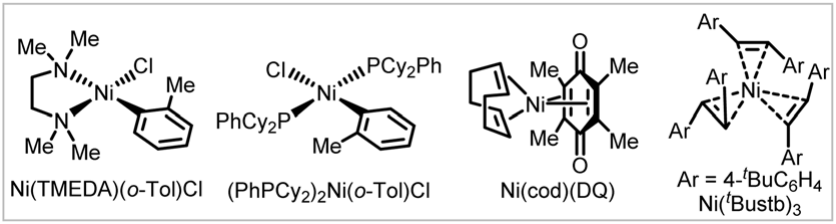					

a Reactions run at 0.2 mmol scale under standard conditions in the glovebox for simplicity, yields were determined by ^1^H NMR spectroscopy using 1,1,2,2-tetrachloroethane as an internal standard.

b Without external PhPCy_2_ ligand.

c 74% yield outside the glovebox.

**Scheme 3:**
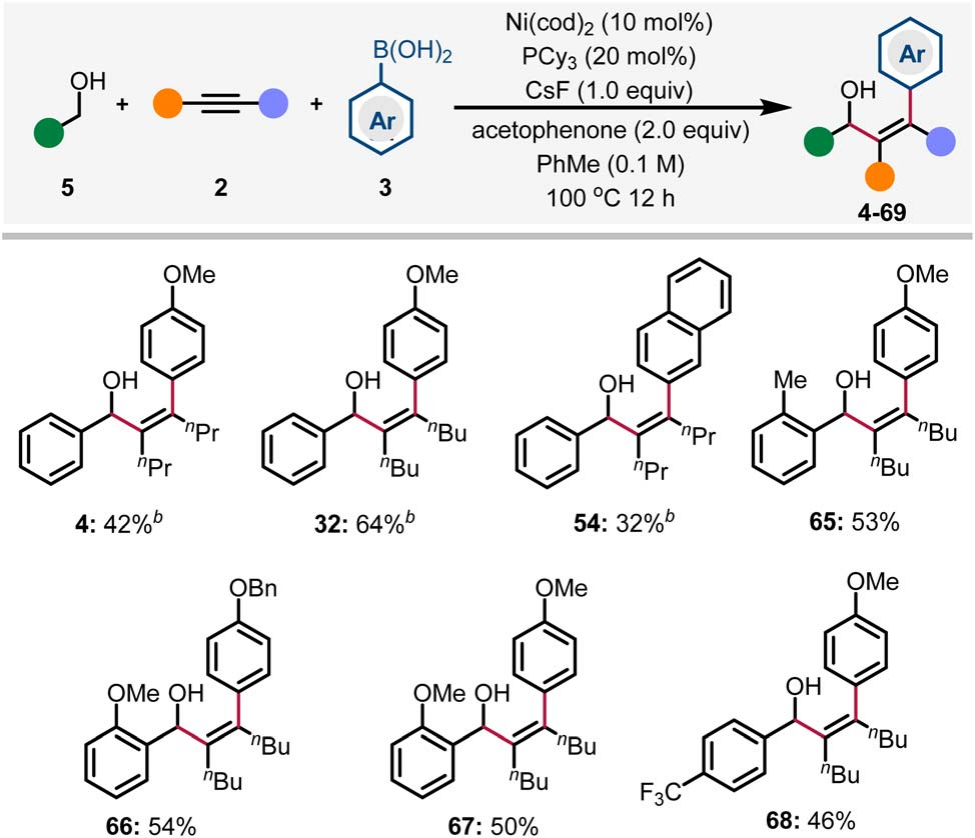
Redox-triggered arylative coupling of benzyl alcohols with alkynes and arylboronic acids. ^*a*^ Reaction conditions: 5 (0.2 mmol), 2 (0.4 mmol), 3 (0.4 mmol), Ni(cod)_2_ (10 mol%), PCy_3_ (20 mol%), CsF (0.2 mmol), acetophenone (0.4 mmol), toluene (0.1 M), 100 °C for 12 h. Isolated yields are reported. ^*b* 1^H NMR yields.

The use of alcohols as abundant and stable precursors of the corresponding aldehydes has been developed into a well-recognized process in organic synthesis.^28^ The Krische group has pioneered in the field of noble metal-catalyzed transfer hydrogenative coupling of alcohols with various π-unsaturated systems through a redox-neutral process.^29^ Recently, Matsubara & Kurahashi and Shi groups also discovered the Ni-catalyzed redox-neutral coupling reaction of benzyl alcohols and alkynes to form allylic alcohols in one single step.^30^ The main advantage of these reactions is the manipulation of the oxidation state in one catalytic cycle, thus ensuring the formation and consumption of the transient aldehyde *in situ*. Based on these findings, we envisioned that benzyl alcohol could be employed in this redox-triggered three-component arylative coupling reaction. After extensive optimizations (see Tables S2–S6 in the ESI† for details), we were pleased to find that 4 was formed in 42% yield. Here, the alkyne plays a dual role as a reactant as well as a sacrificial oxidant. Acetophenone is a co-oxidant, while other alternatives like chlorobenzene and ketones are less reactive.^31^ Herein, our preliminary results are shown in Scheme 3; diverse arylated allylic alcohols were generated in synthetically useful yields *via* the redox-triggered coupling of benzyl alcohols.

**Scheme 4:**
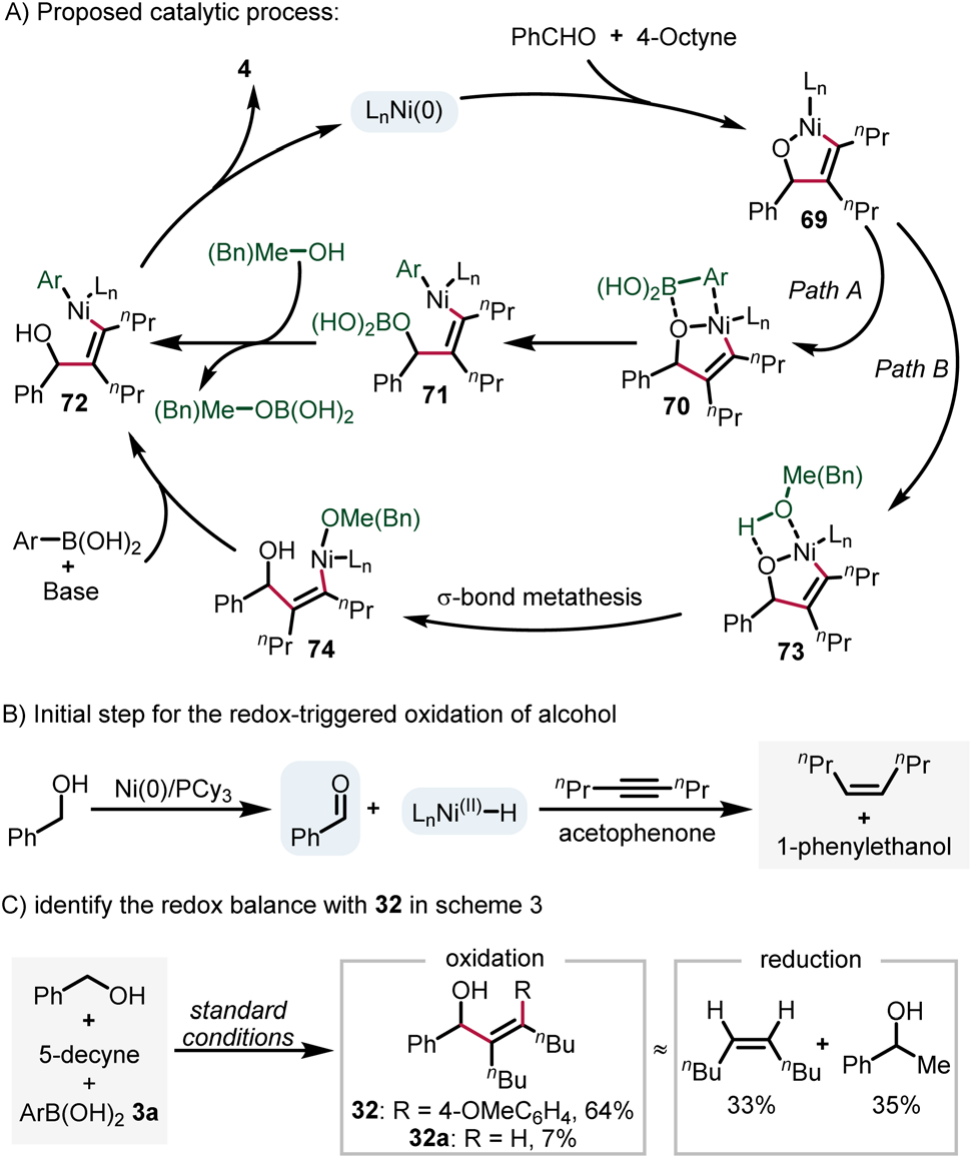
Proposed mechanism for the arylative coupling reactions.

Based on the results presented above and previous reports,^32^ we postulate a plausible reaction mechanism (Scheme 4A). Initially, oxidative cyclization of benzaldehyde and 4-octyne gives the nickelacycle intermediate 69. Subsequently, two possible pathways may account for the formation of aryl–Ni(ii)–vinyl species (72). Path A is direct transmetallation with arylboronic acid, while path B is the protonation of 71 by methanol or benzyl alcohol to form the Ni(ii)–alkoxide intermediate 74 and the subsequent transmetallation step. Finally, a reductive elimination step affords the product 4 and regenerates the catalyst. For the redox-triggered process described in Scheme 3, the reaction was initiated by alcohol dehydrogenation. The corresponding Ni–H species was trapped with a sacrificial alkyne and acetophenone (Scheme 4B),^30*a*^ which was supported by experimental data outlined in Scheme 4C*via* the identification of the redox-balance of 32.

## Conclusions

In summary, we have developed an efficient and practical method for the Ni-catalyzed arylative coupling reaction of alkynes with arylboronic acids and aldehydes to afford stereodefined multisubstituted allylic alcohols, which are not straightforwardly accessible by conventional methods. The reaction is completely *Z*-selective without the use of any aggressive organometallic nucleophiles or reductants. Moreover, benzylic alcohols are viable coupling partners *via* oxidation state manipulation and arylative coupling reaction in one single operation. Facile assembly of these feedstock chemicals with earth abundant Ni-catalysis enables this promising method for the efficient synthesis of stereodefined arylated allylic alcohols. Efforts to expand the scope of this arylative coupling chemistry to unlock more chemical space are still underway in our laboratory.

## Data availability

Optimization tables, experimental procedures and characterization data for all new compounds are provided in the ESI.†

## Author contributions

S.-C. T. and F.-C. M. contributed equally to this work. Y.-L. Z. conceived this project and optimized the conditions. S.-C. T. and F.-C. M. evaluated the reaction scope and performed other experiments. Y.-L. Z. and T. W. co-supervised this project and wrote the manuscript with input from all authors.

## Conflicts of interest

There are no conflicts to declare.

## Supplementary Material

SC-014-D2SC05894D-s001

SC-014-D2SC05894D-s002
