# Supplementary material for: Ni-catalyzed arylation of alkynes with organoboronic acids and aldehydes to access stereodefined allylic alcohols
Source: Chem Sci. 2023 Jan 20;14(8):2040–5. doi: 10.1039/d2sc05894d (PMC9945163; doi:10.1039/d2sc05894d)

Electronic Supplementary Material (ESI) for Chemical Science.
This journal is © The Royal Society of Chemistry 2023

Supporting Information

Ni-catalyzed arylation of alkynes with organoboronic acids and aldehydes to access stereodefined allylic alcohols

Si-Chen Tao,† Fan-Cheng Meng,† Tie Wang* and Yan-Long Zheng*

Tianjin Key Laboratory of Drug Targeting and Bioimaging, Life and Health Intelligent Research Institute,

Tianjin University of Technology, Tianjin, 300384, P. R. China.

E-mail: ylzheng@email.tjut.edu.cn, wangtie@email.tjut.edu.cn

†These authors contribute equally to this work

Table of Contents

1. General information S2
  1.1. General experimental details S2
  1.2. Instrumentation S2
  1.3. Materials S2
2. Reaction optimizations S2
3. Characterization data for the substrate scope S6
  3.1 General procedure A S6
  3.2 General procedure B S33
4. X-Ray crystal Structure of 7, 9, 10 and 11 .... S36
5. Scope limitations and miscellaneous experiments S41
  5.1 Scope limitations S41
  5.2 Miscellaneous experiments S42
6. NMR spectra S45

1. General information

1.1. General experimental details

Unless otherwise indicated, reactions were conducted under an atmosphere of argon in 8 mL screw-capped vials that were oven dried (120 °C). Column chromatography was performed manually using 200–300 mesh silica gel. Analytical thin layer chromatography (TLC) was conducted with glass-backed Silica Gel 60 F254 pre-coated plates. Visualization of developed plates was performed under UV light (254 nm) and/or using $KMnO_4$.

1.2. Instrumentation

^{1}H NMR and ^{13}C NMR spectra were recorded on a Bruker AVANCE 400 spectrometer. ^{1}H NMR spectra were internally referenced to the residual solvent signal (e.g., $CDCl_3$ = 7.26 ppm). ^{13}C NMR spectra were internally referenced to the residual solvent signal (e.g., $CDCl_3$ = 77.16 ppm). Data for ^{1}H NMR are reported as follows: chemical shift (δ ppm), multiplicity (s = singlet, d = doublet, t = triplet, q = quartet, m = multiplet), coupling constant (Hz), integration. NMR yields for optimization studies were obtained by ^{1}H NMR analysis of the crude reaction mixture using 1,1,2,2-tetrachloroethane as an internal standard. IR spectra were obtained using a AIM-9000 with a diamond ATR crystal (Shimadzu Corporation) and are reported in terms of frequency of absorption (cm^{-1}). Melting point ranges were determined on a RY-I melting point apparatus and uncorrected. High resolution mass spectrometric data were obtained on an AB 5600+ UPLC/MS spectrometer (ESITOF). X-ray single-crystal diffraction data were collected on a Rigaku Oxford Diffraction Rigaku XtaLAB Pro II AFC12 (RINC) diffractometer at 293(2) K with Cu K α radiation (λ = 1.54184 Å) in the ω scan mode.

1.3. Materials

Organic solvents were purified by rigorous degassing with nitrogen before passing through a Mikrouna solvent purification system. Low water content was confirmed by Karl Fischer titration (<20 ppm for all solvents). $Ni(cod)_2$, $NiBr_2$·glyme, Ni(TMEDA)(*o*-Tol)Cl and $(PhPCy_2)_2$Ni(*o*-Tol)Cl were purchased from Sigma-Aldrich. Ni(cod)(DQ), Ni(^{t}Bustb)$_3$ and commercial phosphine ligands were purchased from LaaJoo (a Sinocompound Company). Unless otherwise noted, the commercially available starting materials were obtained from TCI, Innochem, Adamas, Accela and BidePharm and used without further purification.

2. Reaction optimization

In addition to the data presented in Table 1 of the manuscript, a selection of further reaction optimization data on different reactions are given in Tables S1–S6 below.

Table S1 Screening of ligands, bases, solvents, additives, and reaction temperature.[a]

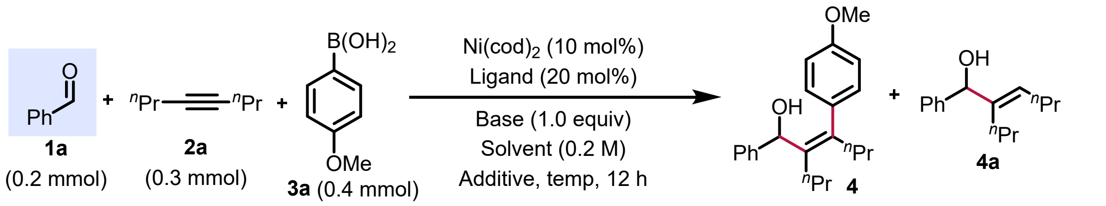

Entry	Ligand	Base	Solvent	Additive	temp. (°C)	Yield (%) [a] 4	Yield (%) [a] 4a
1	$PhPCy_2$	CsF	PhMe	—	60	8	0
2	$PhPCy_2$	K_2CO_3	PhMe	—	60	24	0
3	$PhPCy_2$	Cs_2CO_3	PhMe	—	60	13	0
4	$PhPCy_2$	K_3PO_4	PhMe	—	60	17	0
5	$PhPCy_2$	CsF	EtOH	—	60	28	11
6	$PhPCy_2$	CsF	PhMe/^{i}PrOH = 9:1	—	80	25	11
7	$PhPCy_2$	K_2CO_3	PhMe/^{i}PrOH = 9:1	—	80	46	trace
8	$PhPCy_2$	Na_2CO_3	PhMe/^{i}PrOH = 9:1	—	80	7	trace
9	$PhPCy_2$	Rb_2CO_3	PhMe/^{i}PrOH = 9:1	—	80	51	11
10	Ph_2PCy	Rb_2CO_3	PhMe/^{i}PrOH = 9:1	—	80	46	8
11	PAd_2Bu	Rb_2CO_3	PhMe/^{i}PrOH = 9:1	—	80	17	9
12	CyJohnPhos	Rb_2CO_3	PhMe/^{i}PrOH = 9:1	—	80	10	n.d.
13	dppp	Rb_2CO_3	PhMe/^{i}PrOH = 9:1	—	80	<5	n.d.
14	dppe	Rb_2CO_3	PhMe/^{i}PrOH = 9:1	—	80	<5	n.d.
15	$PhPCy_2$	Rb_2CO_3	PhMe/EtOH = 9:1	—	80	62	11
16	$PhPCy_2$	Rb_2CO_3	PhMe/EtOH = 9:1	H_2O (1.0 eq)	80	44	11
17	$PhPCy_2$	KF	PhMe/EtOH = 9:1	—	80	54	6
18	$PhPCy_2$	Na_3PO_4	PhMe/EtOH = 9:1	—	80	10	n.d.
19	$PhPCy_2$	K_3PO_4	PhMe/EtOH = 9:1	—	80	71	8
20	$PhPCy_2$	K_3PO_4	PhMe/EtOH = 9:1	—	70	68	7
21	$PhPCy_2$	K_3PO_4	PhMe/EtOH = 9:1	—	90	59	9
22	$PhPCy_2$	K_3PO_4	PhMe/EtOH = 9:1	—	100	48	7
23	$PhPCy_2$	K_3PO_4	PhMe/EtOH = 9:1 (0.1 M)	—	80	61	7
24	PCy_3	K_3PO_4	PhMe/EtOH = 9:1	—	80	46	15
25	$PCyp_3$	K_3PO_4	PhMe/EtOH = 9:1	—	80	61	11
26	Ph_2PMe	K_3PO_4	PhMe/EtOH = 9:1	—	80	11	3
27	(*R*)-AntPhos	K_3PO_4	PhMe/EtOH = 9:1	—	80	7	3
28	(*S*)-NMDPP	K_3PO_4	PhMe/EtOH = 9:1	—	80	53	10
29	$PhPCy_2$	K_3PO_4	PhMe/EtOH = 9:1	MMA (20 mol%)	80	74	12
30	$PhPCy_2$	K_3PO_4	PhMe/EtOH = 9:1	MMA (50 mol%)	80	66	8
31	$PhPCy_2$	K_3PO_4	PhMe/EtOH = 9:1	DMFU (20 mol%)	80	22	5
32	$PhPCy_2$	K_3PO_4	PhMe/EtOH = 9:1	$P(OPh)_3$ (20 mol%)	80	13	4
33	$PhPCy_2$	K_3PO_4	PhMe/EtOH = 9:1	*t*-butyl acrylate (20 mol%)	80	47	9
34	$PhPCy_2$	K_3PO_4	Benzene/EtOH = 9:1	—	80	71	8
35	$PhPCy_2$	K_3PO_4	Dioxane/EtOH = 9:1	—	80	46	5
36	$PhPCy_2$	K_3PO_4	THF/EtOH = 9:1	—	80	41	6
37	$PhPCy_2$	K_3PO_4	CPME/EtOH = 9:1	—	80	45	6
38	$PhPCy_2$	K_3PO_4	PhMe/MeOH = 9:1	—	80	82 (79)[b]	8
39	$PhPCy_2$	K_3PO_4	PhMe	—	80	42	7

a: Yieds were determinated by ^{1}H NMR using 1,1,2,2-tetrachloroethane as an internal standard. b: Isolation yield.
n.d. means "not determined".

Table S2 Initial evaluation of redox-triggered arylation reaction with various ligands.[a]

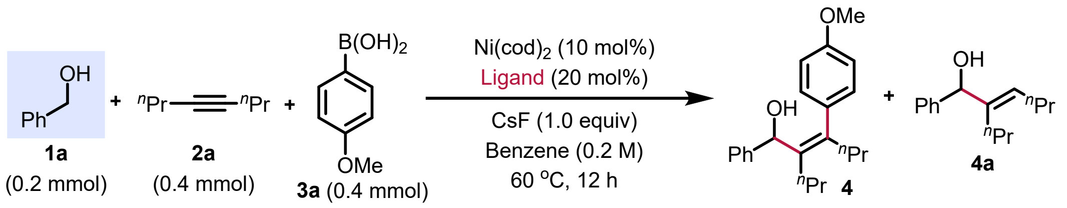

Entry	Ligand	Yield (%) [a] 4	4a
1	IPr·HCl	6	26
2	SIPr·HCl	8	25
3	IMes·HCl	7	43
4	SIMes·HCl	5	30
5	NHC-L1	0	20
6	PCy_3	20	16
7	$P^tBu_3 \cdot HBF_4$	9	13
8	dcype	0	33
9	Xphos	0	6
10	CyJohnPhos	trace	trace

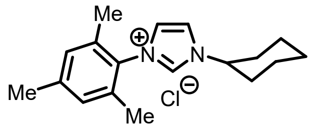

NHC-L1

a: Yieds were determined by ^{1}H NMR using 1,1,2,2-tetrachloroethane as an internal standard, 20 mol% *t*-BuOK was added when NHC ligands were employed.

Table S3 Evaluation of reaction temperature and solvent on redox-triggered arylation reaction.[a]

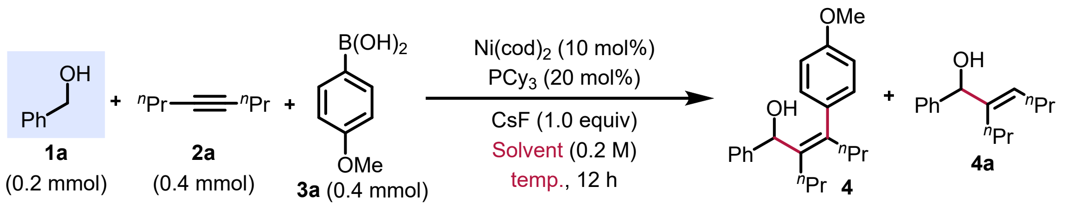

Entry	Solvent	temp. (°C)	Yield (%) [a] 4	4a
1	Dioxane	60	12	38
2	THF	60	10	40
3	DME	60	13	16
4	^{i}PrOH	60	15	20
5	Benzene/EtOH = 4:1	60	20	12
6	Benzene/^{i}PrOH = 4:1	60	32	16
7	Benzene/^{i}PrOH = 4:1	80	28	10
8	Benzene/^{i}PrOH = 4:1	100	26	6
9	Benzene	100	28	5
10	PhMe	100	32	9

a: Yieds were determined by ^{1}H NMR using 1,1,2,2-tetrachloroethane as an internal standard.

Table S4 Evaluation of ligand and base on redox-triggered arylation reaction.[a]

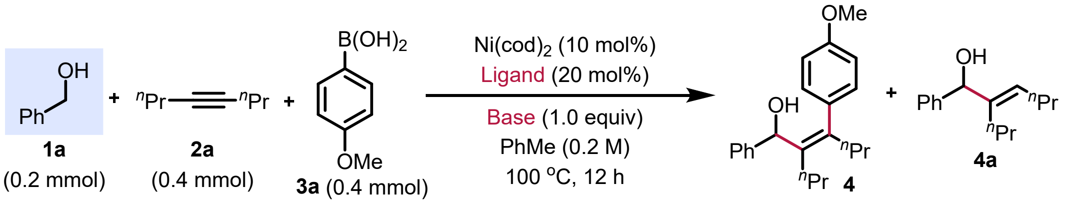

Entry	Ligand	Base	Yield (%) [a] 4	4a
1	$PhPCy_2$	CsF	19	5
2	$PCyp_3$	CsF	0	n.d.
3	PBu_3	CsF	0	0
4	IMes·HCl	CsF	12	22
5	PCy_3	Cs_2CO_3	27	10
6	PCy_3	K_3PO_4	29	8
7	PCy_3	KOAc	9	10
8	PCy_3	CsF (3.0 eq)	14	12
9	PCy_3	w/o CsF	0	10

a: Yieds were determined by ^{1}H NMR using 1,1,2,2-tetrachloroethane as an internal standard, 20 mol% *t*-BuOK was added when NHC ligands were employed. n.d. means "not determined".

Table S5 Evaluation of additive/oxidants on redox-triggered arylation reaction.[a]

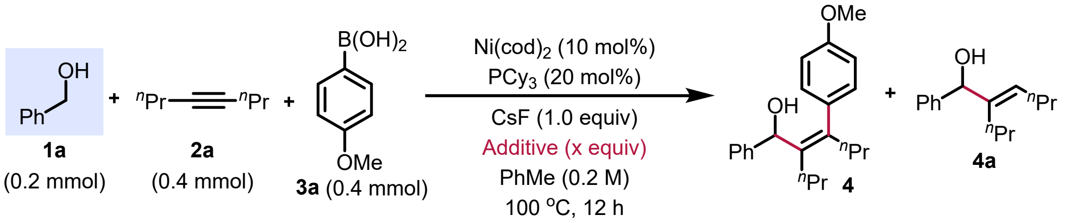

Entry	Additive	Yield (%) [a] 4	4a
1	$ZnCl_2$ (50 mol%)	0	n.d.
2	Acetophenone (1.0 eq)	33	12
3	Benzophenone (1.0 eq)	35	10
4	Benzophenone (2.0 eq)	40	9
5	Acetophenone (2.0 eq)	40	8
6	Acetophenone (2.0 eq) at 0.1 M	42	6
7	2,2,2-Trifluoroacetophenone (2.0 eq)	4	13
8	4-$CF_3C_6H_4COCH_3$ (2.0 eq)	38	6
9	1-indanone (2.0 eq)	37	10
10	Acetone (5.0 eq)	36	10
11	2-Pentanone (2.0 eq)	33	7
12	Cyclopentanone (2.0 eq)	34	7
13	Cyclohexanone (2.0 eq)	36	9
14	Pivaldehyde (2.0 eq)	7	7
15	PhCl (1.5 eq)	28	7
16	PhBr (1.5 eq)	10	n.d.

a: Yieds were determined by ^{1}H NMR using 1,1,2,2-tetrachloroethane as an internal standard. n.d. means "not determined".

Table S6 Evaluation of metal catalyst on redox-triggered arylation reaction.[a]

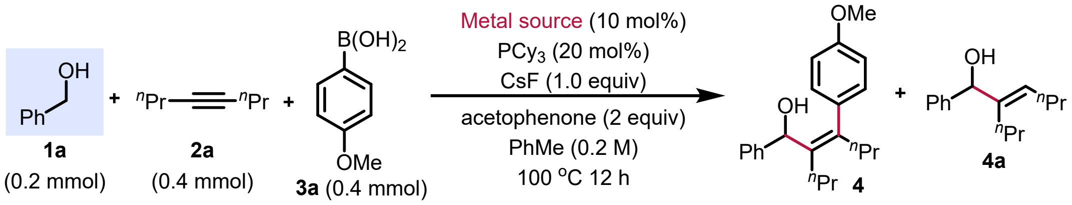

Entry	Metal source	Yield (%) [a] 4	Yield (%) [a] 4a
1	$Ni(OTf)_2$	0	10
2	$Ni(PPh_3)_2Cl_2$	0	trace
3	$Ni(dppp)Cl_2$	0	trace
4	Ni(TMEDA)(*o*–Tol)Cl	8	11
5	Ni(cod)(DQ)	19	6
6	$Ni(^tBustb)_3$	4	11
7	$CoCl_2$ + Zn dust (2.0 equiv)	0	ca. 10
8	$CoBr_2$ + Zn dust (2.0 equiv)	0	ca. 10
9	$Ru_3(CO)_{12}$	n.d.	n.d.
10	$Rh(nbd)_2BF_4$	trace	n.d.

a: Yieds were determined by ^{1}H NMR using 1,1,2,2-tetrachloroethane as an internal standard.
n.d. means "not determined".

3. Characterization data for the substrate scope

3.1 General procedure A

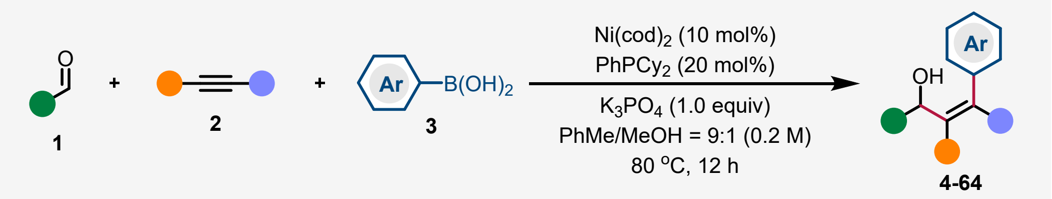

In a glovebox, an oven dried screw-capped 8 mL vial was charged with a magnetic stir bar, $Ni(cod)_2$ (5.5 mg, 0.02 mmol, 10 mol%), $PhPCy_2$ (11 mg, 0.04 mmol, 20 mol%), K_3PO_4 (42.5 mg, 0.2 mmol, 1.0 equiv.) and aryl boronic acids (0.4 mmol, 2.0 equiv.) were added successively. Then degassed toluene (0.9 mL) was added and the catalyst mixture was stirred at rt for 5 min. Aldehydes (0.2 mmol), alkynes (0.3 mmol) and MeOH (0.1 mL) were then added. The vial was sealed with a teflon-lined screw cap, shipped outside of the glovebox, and added to a pre-heated aluminum heating mental at 80 °C. After stirring for 12 h, the vial was removed and allowed to cool to rt. The reaction mixture was diluted with ethyl acetate and filtered through a short plug of silica gel. The crude solution was concentrated in vacuo and subjected to column chromatography to provide pure product.

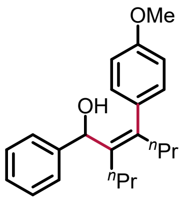

4: (*Z*)-3-(4-methoxyphenyl)-1-phenyl-2-propylhex-2-en-1-ol was prepared according to the general procedure A. Colorless oil after flash column chromatography (petroleum ether/EtOAc = 20:1), 51.2 mg, 79%. **^{1}H NMR** (400 MHz, $CDCl_3$) *δ* 7.31 – 7.27 (m, 4H), 7.22 – 7.18 (m, 1H), 7.13 – 7.09 (m, 2H), 6.89 – 6.86 (m, 2H), 5.38 (s, 1H), 3.81 (s, 3H), 2.37 – 2.31 (m, 2H), 2.15 – 2.07(m, 1H), 1.96 – 1.89 (m, 1H), 1.44 – 1.36 (m, 1H), 1.33 – 1.24 (m, 3H), 1.04 – 0.96 (m, 1H), 0.88 (t, J = 7.4 Hz, 3H), 0.80 (t, *J* = 7.2 Hz, 3H); **^{13}C NMR** (100 MHz, $CDCl_3$) *δ* 158.3, 143.3, 140.9, 137.4, 134.7, 129.8, 128.1, 126.8, 125.8, 113.7, 74.0, 55.4, 36.7, 30.0, 24.5, 21.3, 15.0, 14.3. **IR**: ν (cm^{-1}) 3333, 2959, 2932, 2870, 1609, 1508, 1464, 1450, 1287, 1246, 1175, 1036, 997, 831, 800. **HRMS** (ESI-TOF): m/z Calcd for $C_{22}H_{28}O_2Na$ $[M+Na]^+$ 423.2304, found 423.2300.

Note: the reaction could also be performed at 1 mmol scale, 250 mg **4** was isolated, 77% yield.

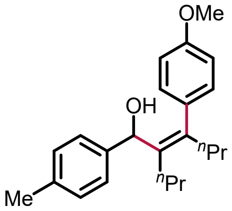

5: (*Z*)-3-(4-methoxyphenyl)-2-propyl-1-(p-tolyl)hex-2-en-1-ol was prepared according to the general procedure A. White solid (melting point 78–80 °C) after flash column chromatography (petroleum ether /EtOAc = 25:1), 41.3 mg, 61%. **^{1}H NMR** (400 MHz, $CDCl_3$) *δ* 7.25 – 7.23 (m, 2H), 7.10 – 7.07 (m, 2H), 6.99 – 6.94 (m, 2H), 6.89 – 6.86 (m, 2H), 5.35 (s, 1H), 3.81 (s, 3H), 2.38 – 2.26 (m, 5H), 2.12 – 2.05 (m, 1H), 1.95 – 1.87 (m, 1H), 1.57 (s, 1H), 1.43 – 1.33 (m, 1H), 1.31 – 1.23 (m, 2H), 1.03 – 0.94 (m, 1H), 0.87 (t, *J* = 7.2 Hz, 3H), 0.81 (t, *J* = 7.2 Hz, 3H), **^{13}C NMR** (100 MHz, $CDCl_3$) *δ* 158.3, 140.5, 140.4, 137.5, 136.3, 134.8, 129.8, 128.8, 125.8, 113.7, 73.9, 55.3, 36.7, 30.0, 24.5, 21.3, 21.2, 15.0, 14.3. **IR**: ν (cm^{-1}) 3331, 2999, 2957, 2932, 2870, 2837, 1609, 1510, 1468, 1456, 1377, 1287, 1242, 1175, 1036, 1001, 833,810, 777. **HRMS** (ESI-TOF): m/z Calcd for $C_{23}H_{30}O_2Na$ $[M+Na]^+$ 361.2150, found 361.2143.

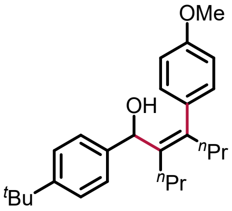

6: (*Z*)-1-(4-(tert-butyl)phenyl)-3-(4-methoxyphenyl)-2-propylhex-2-en-1-ol was prepared according to the general procedure A. White solid (melting point 88–90 °C) after flash column chromatography (petroleum ether /EtOAc = 20:1), 40.3 mg, 53%. **^{1}H NMR** (400 MHz, $CDCl_3$) *δ* 7.33 – 7.29 (m, 2H), 7.15 – 7.11 (m, 2H), 7.10 – 7.06 (m, 2H), 6.90 – 6.86 (m, 2H), 5.38 (d, *J* = 2.4 Hz, 1H), 3.81 (s, 3H), 2.38 – 2.27 (m, 2H), 2.12 – 2.05 (m, 1H), 1.95 – 1.88 (m, 1H), 1.60 (d, *J* = 2.8 Hz, 1H), 1.50 – 1.35 (m, 1H), 1.30 (s, 9H), 1.31 – 1.24 (m, 2H), 1.14 – 1.05 (m, 1H), 0.90 – 0.80 (m, 6H). **^{13}C NMR** (100 MHz, $CDCl_3$) *δ* 158.2, 149.6, 140.5, 140.3, 137.4, 134.8, 129.8, 125.6, 125.0, 113.7, 74.0, 55.3, 36.7, 34.5, 31.5, 30.1, 24.6, 21.3, 15.0, 14.3. **IR**: ν (cm^{-1}) 3298, 2957, 2870, 1607, 1508, 1468, 1242, 1180, 1103, 1038, 831, 810, 758. **HRMS** (ESI-TOF): m/z Calcd for $C_{26}H_{36}O_2Na$ $[M+Na]^+$ 403.2608, found 403.2613.

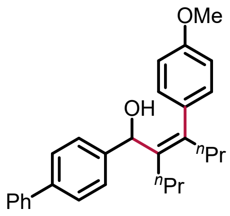

7: (*Z*)-1-([1,1'-biphenyl]-4-yl)-3-(4-methoxyphenyl)-2-propylhex-2-en-1-ol was prepared according to the general procedure A.White solid (melting point 96–99 °C) after flash column chromatography (petroleum ether/EtOAc = 20:1), 45 mg, 56%. **^{1}H NMR** (400 MHz, $CDCl_3$) *δ* 7.63 – 7.56 (m, 2H), 7.56 – 7.49 (m, 2H), 7.45 – 7.41 (m, 2H), 7.38 – 7.31 (m, 3H), 7.15 – 7.11 (m, 2H), 6.90 – 6.87 (m, 2H), 5.43 (d, *J* = 3.2 Hz, 1H), 3.81 (s, 3H), 2.38 – 2.33 (m, 2H), 2.19 – 2.12 (m, 1H), 2.01 – 1.93 (m, 1H), 1.64 (d, *J* = 3.6 Hz, 1H), 1.49 – 1.39 (m, 1H), 1.35 – 1.25 (m, 2H), 1.14 – 1.04 (m, 1H), 0.89 (t, *J* = 7.4 Hz, 3H), 0.83 (t, *J* = 7.2 Hz, 3H). **^{13}C NMR** (100 MHz, $CDCl_3$) *δ* 158.3, 142.5, 141.8, 140.9, 139.6, 137.3, 134.7, 129.8, 128.9, 127.2, 127.1, 126.8, 126.3, 113.7, 73.9, 55.4, 36.8, 30.1, 24.6, 21.3, 15.0, 14.3. **IR**: ν (cm^{-1}) 3316, 2953, 2361, 2340, 1607, 1508, 1244, 1179, 1032, 999, 836. **HRMS** (ESI-TOF): m/z Calcd for $C_{28}H_{32}O_2Na$ $[M+Na]^+$ 423.2295, found 423.2300.

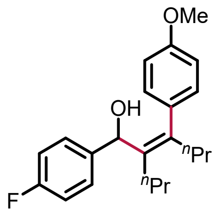

8: (*Z*)-1-(4-fluorophenyl)-3-(4-methoxyphenyl)-2-propylhex-2-en-1-ol was prepared according to the general procedure A. White solid (melting point 63–66 °C) after flash column chromatography (petroleum ether /EtOAc = 20:1), 40.2 mg, 62%. **^{1}H NMR** (400 MHz, $CDCl_3$) *δ* 7.25 – 7.23 (m, 2H), 7.10 – 7.07 (m, 2H), 6.99 – 6.94 (m, 2H), 6.89 – 6.86 (m, 2H), 5.35 (d, *J* = 3.2 Hz, 1H), 3.81 (s, 3H), 2.38 – 2.26 (m, 2H), 2.12 – 2.05 (m, 1H), 1.95

– 1.87 (m, 1H), 1.57 (s, 1H), 1.43 – 1.33 (m, 1H), 1.31 – 1.23 (m, 2H), 1.03 – 0.94 (m, 1H), 0.87 (t, *J* = 7.4 Hz, 3H), 0.81 (t, *J* = 7.4 Hz, 3H), **^{13}C NMR** (100 MHz, $CDCl_3$) *δ* 161.09 (*J* = 242.9 Hz), 158.4, 141.0, 139.0 (*J* = 3.0 Hz), 137.3, 134.6, 129.7, 127.4 (*J* = 7.9 Hz), 114.9 (*J* = 21.1 Hz), 113.8, 73.5, 55.4, 36.7, 29.9, 24.5, 21.2, 15.0, 14.3. **IR**: ν (cm^{-1}) 3314, 2960, 2934, 2872, 1609, 1506, 1468, 1456, 1285, 1244, 1217, 1173, 1153, 1026, 1013, 1001, 858, 837, 814, 789. **HRMS** (ESI-TOF): m/z Calcd for $C_{22}H_{27}FO_2Na$ $[M+Na]^+$ 365.1887, found 365.1893.

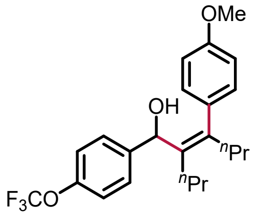

9: (*Z*)-3-(4-methoxyphenyl)-2-propyl-1-(4-(trifluoromethoxy)phenyl)hex-2-en-1-ol was prepared according to the general procedure A. Yellow solid (melting point 73–74 °C) after flash column chromatography (petroleum ether /EtOAc = 20:1), 63.7 mg, 78%. **^{1}H NMR** (400 MHz, $CDCl_3$) *δ* 7.33 – 7.29 (m, 2H), 7.15 – 7.11 (m, 2H), 7.10 – 7.06 (m, 2H), 6.90 – 6.86 (m, 2H), 5.38 (d, *J* = 2.4 Hz, 1H), 3.81 (s, 3H), 2.38 – 2.27(m, 2H), 2.12 – 2.05 (m, 1H), 1.95 – 1.88 (m, 1H), 1.60 (d, *J* = 2.8 Hz, 1H), 1.44 – 1.35 (m, 1H), 1.31 – 1.25 (m, 2H), 1.05 – 0.95 (m, 1H), 0.88 (t, *J* = 7.4 Hz, 3H), 0.82 (t, *J* = 7.2 Hz, 3H). **^{13}C NMR** (100 MHz, $CDCl_3$) δ 158.4, 148.1 (*J* = 1.9 Hz), 142.0, 141.4, 137.1, 134.5, 129.7, 127.2, 120.63 (*J* = 255.2 Hz), 120.59, 113.8, 73.5, 55.4, 36.7, 29.9, 24.5, 21.2, 15.0, 14.3. **IR**: ν (cm^{-1}) 3339, 2968, 2872, 1609, 1508, 1466, 1379, 1265, 1161, 1038, 920, 856, 799, 756. **HRMS** (ESI-TOF): m/z Calcd for $C_{23}H_{27}F_3O_3Na$ $[M+Na]^+$ 431.1805, found 431.1810.

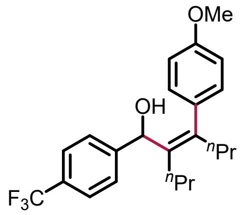

10: (*Z*)-3-(4-methoxyphenyl)-2-propyl-1-(4-(trifluoromethyl)phenyl)hex-2-en-1-ol was prepared according to the general procedure A. White solid (melting point 69–71 °C) after flash column chromatography (petroleum ether /EtOAc = 20:1), 71.4 mg, 91%. **^{1}H NMR** (400 MHz, $CDCl_3$) *δ* 7.54 (d, *J* = 8.0 Hz, 2H), 7.41 (d, *J* = 8.0 Hz, 2H), 7.11 – 7.08 (m, 2H), 6.90 – 6.87 (m, 2H), 5.42 (s, 1H), 3.81 (s, 3H), 2.38 – 2.28 (m, 2H), 2.13 – 2.05 (m, 1H), 1.91 – 1.83 (m, 1H), 1.66 (d, *J* = 3.2 Hz, 1H), 1.45 – 1.37 (m, 1H), 1.33 – 1.27 (m, 2H), 1.05 – 0.98 (m, 1H), 0.88 (t, *J* = 7.4 Hz, 3H), 0.81 (t, *J* = 7.4 Hz, 3H).**^{13}C NMR** (100 MHz, $CDCl_3$) *δ* 158.4, 147.4, 141.8, 136.9, 134.4, 129.7, 129.0 (q, *J* = 31.9 Hz), 126.2, 125.0 (q, *J* = 3.9 Hz), 124.4 (q, *J* = 270.2 Hz), 113.9, 73.7, 55.4, 36.7, 29.9, 24.5, 21.2, 14.9, 14.3. **IR**: ν (cm^{-1}) 3300, 2961, 2938, 2876, 1609, 1508, 1464, 1408, 1323, 1285, 1248, 1167,

1132, 1123, 1067, 1038, 858, 835, 820. **HRMS** (ESI-TOF): m/z Calcd for $C_{23}H_{30}O_3Na$ $[M+Na]^+$ 415.1868, found 415.1855.

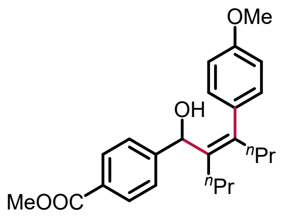

11: methyl (*Z*)-4-(1-hydroxy-3-(4-methoxyphenyl)-2-propylhex-2-en-1-yl)benzoate was prepared according to the general procedure A. White solid (melting point 86–88 °C) after flash column chromatography (petroleum ether /EtOAc = 25:1), 61.2 mg, 80%. **^{1}H NMR** (400 MHz, $CDCl_3$) δ 7.98 – 7.94 (m, 2H), 7.38 – 7.35 (m, 2H), 7.13 – 7.09 (m, 2H), 6.90 – 6.86 (m, 2H), 5.42 (d, *J* = 2.8 Hz, 1H), 3.90 (s, 3H), 3.81 (s, 3H), 2.37 – 2.28 (m, 2H), 2.11 – 2.04 (m, 1H), 1.92 – 1.85 (m, 1H), 1.66 (d, *J* = 3.2 Hz, 1H), 1.41 – 1.33 (m, 1H), 1.3 –1.23 (m, 2H), 0.98 – 0.86 (m, 1H), 0.87 (t, *J* = 7.2 Hz, 3H), 0.78 (t, *J* = 7.2 Hz, 3H). **^{13}C NMR** (100 MHz, $CDCl_3$) δ 167.3, 158.3, 148.7, 141.5, 136.9, 134.5, 129.7, 129.4, 128.5, 125.8, 113.8, 73.7, 55.3, 52.2, 36.7, 29.9, 24.4, 21.2, 14.9, 14.3. **IR**: ν (cm^{-1}) 3329, 3003, 2955, 2930, 2870, 2835, 1724, 1609, 1508, 1470, 1435, 1412, 1375, 1312, 1279, 1244, 1192, 1174, 1105, 1022, 1001, 970, 866, 835, 816, 800, 772. **HRMS** (ESI-TOF): m/z Calcd for $C_{24}H_{30}O_4Na$ $[M+Na]^+$ 405.2036, found 405.2042.

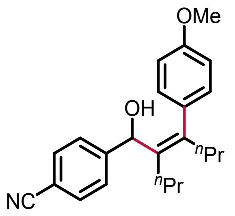

12: (*Z*)-4-(1-hydroxy-3-(4-methoxyphenyl)-2-propylhex-2-en-1-yl)benzonitrile was prepared according to the general procedure A. Colorless oil after flash column chromatography (petroleum ether /EtOAc = 25:1), 40.5 mg, 58%. **^{1}H NMR** (400 MHz, $CDCl_3$) δ 7.60 (s, 1H), 7.51 (t, *J* = 8.8 Hz, 2H), 7.38 (t, *J* = 7.8 Hz, 1H), 7.11 – 7.06 (m, 2H), 6.91 – 6.87 (m, 2H), 5.39 (d, *J* = 3.2 Hz, 1H), 3.81 (s, 3H), 2.38 – 2.27 (m, 2H), 2.11 – 2.03 (m, 1H), 1.89-1.81 (m, 1H), 1.67 (d, *J* = 3.6 Hz, 1H), 1.45 – 1.35 (m, 1H), 1.31 – 1.24 (m, 2H), 1.00 – 0.92 (m, 1H), 0.88 (t, *J* = 7.4 Hz, 3H), 0.81 (t, *J* = 7.4 Hz, 3H). **^{13}C NMR** (100 MHz, $CDCl_3$) δ 158.5, 144.9, 142.3, 136.6, 134.2, 130.5, 130.4, 129.6, 128.8, 119.3, 114.0, 112.1, 73.2, 55.4, 36.7, 29.8, 24.5, 21.2, 14.9, 14.3. **IR**: ν (cm^{-1}) 3329, 3003, 2955, 2930, 2870, 2835, 1724, 1609, 1574, 1508, 1470, 1435, 1412, 1375, 1312, 1279, 1244, 1192, 1175, 1105, 1053, 1022, 1001, 970, 866, 835, 816, 800, 772. **HRMS** (ESI-TOF): m/z Calcd for $C_{23}H_{27}NO_2Na$ $[M+Na]^+$ 372.1934, found 372.1939.

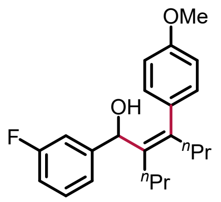

13: (*Z*)-1-(3-fluorophenyl)-3-(4-methoxyphenyl)-2-propylhex-2-en-1-ol was prepared according to the general procedure A. Colorless oil after flash column chromatography (petroleum ether /EtOAc = 20:1), 41.1 mg, 60%. **^{1}H NMR** (400 MHz, $CDCl_3$) δ 7.25 – 7.21 (m, 1H), 7.12 – 7.08 (m, 2H), 7.06 – 6.99 (m, 2H), 6.91 – 6.86 (m, 3H), 5.36 (d, *J* = 3.2 Hz, 1H), 3.81 (s, 3H), 2.39 – 2.27 (m, 2H), 2.13 – 2.06 (m, 1H), 1.94 – 1.86 (m, 1H), 1.59 (d, *J* = 4.0 Hz, 1H), 1.44 – 1.36 (m, 1H), 1.32 – 1.25 (m, 2H), 1.05 – 0.96 (m, 1H), 0.88 (t, *J* = 7.2 Hz, 3H), 0.82 (t, *J* = 7.4 Hz, 3H). **^{13}C NMR** (100 MHz, $CDCl_3$) δ 162.9 (*J* = 243.6 Hz), 158.4, 146.1 (*J* = 7.0 Hz), 141.4, 137.0, 134.5, 129.7, 129.5 (*J* = 8.3 Hz), 121.5 (*J* = 3.7 Hz), 113.8, 113.6 (*J* = 21.1 Hz), 112.9 (*J* = 22.1 Hz), 73.5 (*J* = 23.0 Hz), 55.4, 36.7, 29.9, 24.5, 21.2, 15.0, 14.3. **IR**: ν (cm^{-1}) 3455, 2959, 2932, 2872, 1609, 1589, 1508, 1485, 1466, 1458, 1445, 1283, 1246, 1177, 1123, 1036, 835, 804, 770. **HRMS** (ESI-TOF): m/z Calcd for $C_{22}H_{27}O_2FNa$ $[M+Na]^+$ 365.1887, found 365.1893.

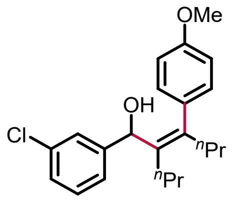

14: (*Z*)-1-(3-chlorophenyl)-3-(4-methoxyphenyl)-2-propylhex-2-en-1-ol was prepared according to the general procedure A. Colorless oil after flash column chromatography (petroleum ether /EtOAc = 20:1), 43 mg, 60%. **^{1}H NMR** (400 MHz, $CDCl_3$) δ 7.30 (s, 1H), 7.21 – 7.14 (m, 3H), 7.11 – 7.07 (m, 2H), 6.90 – 6.86 (m, 2H), 5.34 (d, *J* = 3.2 Hz, 1H), 3.81 (s, 3H), 2.39 – 2.27 (m, 2H), 2.13 – 2.05 (m, 1H), 1.93 – 1.86 (m, 1H), 1.61 (d, *J* = 3.6 Hz, 1H), 1.45 – 1.35 (m, 1H), 1.33 – 1.24 (m, 2H), 1.06 – 0.97 (m, 1H), 0.88 (t, *J* = 7.4 Hz, 3H), 0.82 (t, *J* = 7.2 Hz, 3H). **^{13}C NMR** (100 MHz, $CDCl_3$) δ 158.4, 145.5, 141.5, 136.9, 134.5, 134.1, 129.7, 129.3, 126.9, 126.1, 124.1, 113.8, 73.5, 55.4, 36.7, 29.9, 24.5, 21.2, 15.0, 14.3. **IR**: ν (cm^{-1}) 3449, 2959, 2932, 2870, 1609, 1574, 1508, 1466, 1425, 1377, 1285, 1244, 1177, 1094, 1036, 835, 802, 783. **HRMS** (ESI-TOF): m/z Calcd for $C_{22}H_{27}O_2ClNa$ $[M+Na]^+$ 381.1592, found 381.1597.

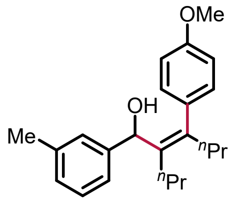

15: (*Z*)-3-(4-methoxyphenyl)-2-propyl-1-(m-tolyl)hex-2-en-1-ol was prepared according to the general procedure A. Colorless oil after flash column chromatography (petroleum ether /EtOAc = 20:1), 38 mg, 56%. **^{1}H NMR** (400 MHz, $CDCl_3$) *δ* 7.17 (t, *J* = 7.4 Hz, 1H), 7.12 – 7.09 (m, 3H), 7.04 (dd, *J* = 17.8, 7.4 Hz, 2H), 6.89 – 6.85 (m, 2H), 5.35 (d, *J* = 3.6 Hz, 1H), 3.80 (s, 3H), 2.36 – 2.31 (m, 5H), 2.15 – 2.08 (m, 1H), 1.97 – 1.89 (m, 1H), 1.57 (s, 1H), 1.46 – 1.37 (m, 1H), 1.33 – 1.24 (m, 2H), 1.09 – 0.99 (m, 1H), 0.88 (t, *J* = 7.2 Hz, 3H), 0.82 (t, *J* = 7.2 Hz, 3H). **^{13}C NMR** (100 MHz, $CDCl_3$) *δ* 158.2, 143.3, 140.7, 137.6, 137.4, 134.7, 129.8, 127.9, 127.5, 126.5, 123.0, 113.7, 74.0, 55.3, 36.7, 30.1, 24.5, 21.7, 21.3, 15.0, 14.3. **IR**: ν (cm^{-1}) 3449, 2959, 2932, 2870, 2172, 1607, 1508, 1464, 1377, 1285, 1244, 1177, 1146, 1103, 1036, 835, 806. **HRMS** (ESI-TOF): m/z Calcd for $C_{23}H_{30}O_2Na$ $[M+Na]^+$ 361.2138, found 361.2143.

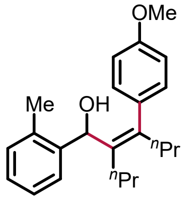

16: (*Z*)-3-(4-methoxyphenyl)-2-propyl-1-(o-tolyl)hex-2-en-1-ol was prepared according to the general procedure A. White solid (melting point 77–79 °C) after flash column chromatography (petroleum ether /EtOAc = 20:1), 43.3 mg, 64%. **^{1}H NMR** (400 MHz, $CDCl_3$) *δ* 7.63 (d, *J* = 7.2 Hz, 1H), 7.23 – 7.01 (m, 4H), 7.00 (d, *J* = 7.2 Hz, 1H), 6.89 (d, *J* = 8.8 Hz, 2H), 5.24 (d, *J* = 2.8 Hz, 1H), 3.82 (s, 3H), 2.44 – 2.39 (m, 1H), 2.29 – 2.25 (m, 1H), 2.07 – 1.95 (m, 2H), 1.88 (s, 3H), 1.61 (d, *J* = 3.2 Hz, 1H), 1.30 – 1.21 (m, 3H), 0.86 (t, *J* = 7.2 Hz, 3H), 0.68 (t, *J* = 7.2 Hz, 3H), 0.61 – 0.50 (m, 1H). **^{13}C NMR** (100 MHz, $CDCl_3$) *δ* 158.3, 141.7, 141.1, 135.3, 135.2, 134.4, 129.7, 129.6, 126.7, 125.6, 125.4, 113.4, 71.8, 55.2, 36.4, 30.3, 23.4, 21.3, 19.4, 14.7, 14.1. **IR**: ν (cm^{-1}) 3333, 3030, 2999, 2957, 2930, 2870, 2833, 1609, 1508, 1483, 1464, 1441, 1377, 1337, 1285, 1246, 1179, 1103, 1038, 1015, 1001, 831, 804, 793. **HRMS** (ESI-TOF): m/z Calcd for $C_{23}H_{30}O_2Na$ $[M+Na]^+$ 361.2138, found 361.2143.

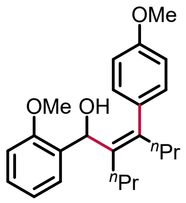

17: (*Z*)-1-(2-methoxyphenyl)-3-(4-methoxyphenyl)-2-propylhex-2-en-1-ol was prepared according to the general procedure A. Colorless oil after flash column chromatography (petroleum ether /EtOAc = 20:1), 49.6 mg, 70%. **^{1}H NMR** (400 MHz, $CDCl_3$) *δ* 7.36 (d, *J* = 8.8 Hz, 1H), 7.21 (td, *J* = 8.0, 1.6 Hz, 1H), 7.08 – 7.04 (m, 2H), 6.94 (td, *J* = 7.6, 1.2 Hz, 1H), 6.85 – 6.81 (m, 2H), 6.79 (d, *J* = 8.0 Hz, 1H), 5.45 (d, *J* = 2.8 Hz, 1H), 3.79 (s, 3H),

3.72 (s, 3H), 2.50 (d, *J* = 2.8 Hz, 1H), 2.37 – 2.33 (m, 2H), 2.20 – 2.13 (m, 1H), 2.07 – 1.99 (m, 1H), 1.50 – 1.41 (m, 1H), 1.32 – 1.23 (m, 2H), 1.06 – 0.96 (m, 1H), 0.88 (t, *J* = 7.4 Hz, 3H), 0.81 (t, *J* = 7.2 Hz, 3H). **^{13}C NMR** (100 MHz, $CDCl_3$) δ 158.1, 157.1, 140.4, 135.9, 135.0, 131.5, 129.8, 128.0, 127.4, 120.5, 113.2, 110.3, 71.1, 55.3, 55.1, 36.6, 30.7, 24.1, 21.3, 14.9, 14.2. **IR**: ν (cm^{-1}) 3491, 2932, 2870, 2835, 1607, 1508, 1489, 1464, 1287, 1244, 1277, 1111, 1034, 833, 756. HRMS (ESI-TOF): m/z Calcd for $C_{23}H_{30}O_2NaO_3$ [M+Na]$^+$ 377.2087, found 377.2090.

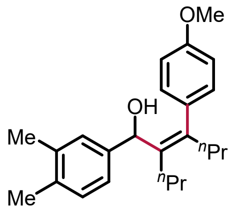

18: (*Z*)-1-(3,4-dimethylphenyl)-3-(4-methoxyphenyl)-2-propylhex-2-en-1-ol was prepared according to the general procedure A. Colorless oil after flash column chromatography (petroleum ether/EtOAc = 20:1), 49.3 mg, 70%. **^{1}H NMR** (400 MHz, $CDCl_3$) δ 7.11 – 7.04 (m, 4H), 7.00 (d, *J* = 8.0 Hz, 1H), 6.86 (d, *J* = 8.4 Hz, 2H), 5.32 (s, 1H), 3.80 (s, 3H), 2.34 (t, *J* = 7.8 Hz, 2H), 2.24 (s, 3H), 2.23 (s, 3H), 2.17 – 2.10 (m, 1H), 1.97 – 1.90 (m, 1H), 1.48 – 1.40 (m, 1H), 1.34 – 1.24 (m, 2H), 1.17 – 1.09 (m, 1H), 0.99 – 0.92 (m, 1H), 0.90 – 0.82 (m, 6H). **^{13}C NMR** (100 MHz, $CDCl_3$) δ 158.3, 140.8, 140.5, 137.5, 136.2, 134.9, 134.9, 129.8, 129.4, 127.1, 123.3, 113.7, 74.0, 55.4, 36.8, 30.2, 24.6, 21.3, 20.1, 19.5, 15.0, 14.3. **IR**: ν (cm^{-1})3476, 2959, 2932, 2870, 1609, 1508, 1456, 1285, 1246, 1177, 1036, 833. **HRMS** (ESI-TOF): m/z Calcd for $C_{24}H_{32}O_2Na$ [M+Na]$^+$ 375.2295, found 375.2300.

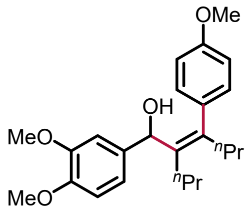

19: (*Z*)-1-(3,4-dimethoxyphenyl)-3-(4-methoxyphenyl)-2-propylhex-2-en-1-ol was prepared according to the general procedure A. Colorless oil after flash column chromatography (petroleum ether/EtOAc = 25:1), 40.7 mg, 53%. **^{1}H NMR** (400 MHz, $CDCl_3$) δ 7.1 – 7.07 (m, 2H), 6.88 – 6.85 (m, 3H), 6.81 – 6.80 (m, 2H), 5.33 (d, *J* = 3.6 Hz, 1H), 3.862 (s, 3H), 3.857 (s, 3H), 3.80 (s, 3H), 2.32 – 2.36 (m, 2H), 2.17 – 2.06 (m, 1H), 2.00 – 1.92 (m, 1H), 1.57 (s, 1H), 1.47 – 1.37 (m, 1H), 1.33 – 1.24 (m, 2H), 1.13 – 1.03 (m, 1H), 0.88 (t, *J* = 7.4 Hz, 3H), 0.84 (t, *J* = 7.2 Hz, 3H). **^{13}C NMR** (100 MHz, $CDCl_3$) δ 158.2, 140.8, 140.5, 137.4, 136.2, 134.9, 134.8, 129.8, 129.4, 127.2, 123.3, 113.7, 74.0, 55.4, 36.7, 30.2, 24.6, 21.3, 20.1, 19.6, 15.0, 14.3. **IR**: ν (cm^{-1}) 3474, 2959, 2932,

2870, 1609, 1508, 1458, 1285, 1244, 1177, 1036, 833. **HRMS** (ESI-TOF): m/z Calcd for $C_{24}H_{32}O_4Na$ [M+Na]$^+$ 407.2193, found 407.2198.

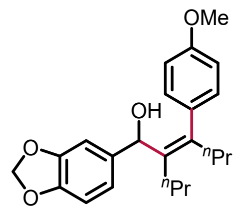

20: (*Z*)-1-(benzo[d][1,3]dioxol-5-yl)-3-(4-methoxyphenyl)-2-propylhex-2-en-1-ol was prepared according to the general procedure A. Colorless oil after flash column chromatography (petroleum ether /EtOAc = 25:1), 44.2 mg, 60%. **^{1}H NMR** (400 MHz, $CDCl_3$) δ 7.09 – 7.07 (m, 2H), 6.88 – 6.85 (m, 2H), 6.78 – 6.72 (m, 3H), 5.93 (s, 2H), 5.28 (s, 1H), 3.81 (s, 3H), 2.38 – 2.30 (m, 2H), 2.14 – 2.07 (m, 1H), 1.97 – 1.90 (m, 1H), 1.55 (s, 1H), 1.46 – 1.38 (m, 1H), 1.32 – 1.23 (m, 2H), 1.13 – 1.04 (m, 1H), 0.89 – 0.82 (m, 6H), **^{13}C NMR** (100 MHz, $CDCl_3$) δ 158.3, 147.5, 146.3, 140.7, 137.5, 137.4, 134.7, 129.8, 118.9, 113.7, 107.9, 106.7, 101.0, 73.8, 55.4, 36.7, 29.9, 24.6, 21.3, 15.1, 14.3. **IR**: ν (cm^{-1}) 3480, 2959, 2918, 2870, 2849, 1730, 1609, 1508, 1489, 1439, 1283, 1244, 1177, 1088, 1040, 934, 835. **HRMS** (ESI-TOF): m/z Calcd for $C_{23}H_{27}O_3$ [M-OH]$^+$ 351.1955, found 351.1948.

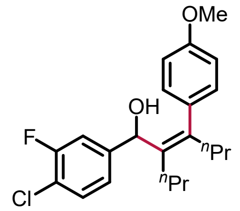

21: (*Z*)-1-(4-chloro-3-fluorophenyl)-3-(4-methoxyphenyl)-2-propylhex-2-en-1-ol was prepared according to the general procedure A. Colorless oil after flash column chromatography (petroleum ether /EtOAc = 25:1), 60.2 mg, 80%. **^{1}H NMR** (400 MHz, $CDCl_3$) δ 7.30 (d, *J* = 8.0 Hz, 1H), 7.17 – 7.06 (m, 3H), 6.99 (d, *J* = 8.4 Hz, 1H), 6.88 (d, *J* = 7.6 Hz, 2H), 5.32 (s, 1H), 3.81 (s, 3H), 2.37 – 2.28 (m, 2H), 2.09 (td, *J* = 12.8, 4.8 Hz, 1H), 1.87 (td, *J* = 12.4, 5.2 Hz, 1H), 1.62 (d, *J* = 2.4 Hz, 1H), 1.45 – 1.35 (m, 1H), 1.31 – 1.25 (m, 2H), 1.07 – 0.99 (m, 1H), 0.88 (t, *J* = 7.2 Hz, 3H), 0.83 (t, *J* = 7.6 Hz, 3H). **^{13}C NMR** (100 MHz, $CDCl_3$) δ 158.5, 158.0 (*J* = 246.7 Hz), 144.6 (*J* = 6.0 Hz), 141.9, 136.7, 134.3, 130.1, 129.6, 122.3 (*J* = 3.4 Hz), 118.9 (*J* = 17.6 Hz), 114.3 (*J* = 21.8 Hz), 113.9, 73.1, 55.4, 36.7, 29.9, 24.6, 21.2, 15.0, 14.3. **IR**: ν (cm^{-1}) 3441, 2970, 2936, 2914, 1605, 1582, 1510, 1491, 1464, 1443, 1420, 1387, 1275, 1246, 1180, 1063, 1047, 1013, 951, 880, 781, 760. **HRMS** (ESI-TOF): m/z Calcd for $C_{22}H_{26}O_2FClNa$ [M+Na]$^+$ 399.1498, found 399.1512.

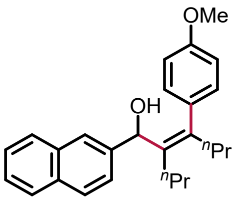

22: (*Z*)-3-(4-methoxyphenyl)-1-(naphthalen-2-yl)-2-propylhex-2-en-1-ol was prepared according to the general procedure A. Sticky colorless oil after flash column chromatography (petroleum ether /EtOAc = 25:1), 45 mg, 60%. **^{1}H NMR** (400 MHz, $CDCl_3$) δ 7.86 – 7.76 (m, 3H), 7.74 (d, J = 8.8 Hz, 1H), 7.51 – 7.39 (m, 2H), 7.30 (d, J = 8.4 Hz, 1H), 7.20 – 7.13 (d, J = 8.8 Hz, 2H), 6.89 (d, J = 8.8 Hz, 2H), 5.54 (s, 1H), 3.81 (s, 3H), 2.41 – 2.33 (m, 2H), 2.19 – 2.11(m, 1H), 1.99 – 1.92 (m, 1H), 1.74 (s, 1H), 1.46 – 1.39 (m, 1H), 1.38 – 1.25 (m, 2H), 1.04 – 0.98 (m, 1H), 0.90 (t, J = 7.2 Hz, 3H), 0.77 (t, J = 7.4 Hz, 3H). **^{13}C NMR** (100 MHz, $CDCl_3$) δ 158.3, 141.2, 140.9, 137.2, 134.7, 133.4, 132.6, 129.8, 128.2, 127.7, 127.6, 126.0, 125.6, 124.7, 124.0, 113.9, 74.2, 55.4, 36.8, 30.0, 24.5, 21.3, 15.0, 14.3. **IR**: ν (cm^{-1}) 3435, 2959, 2932, 2870, 1607, 1508, 1464, 1285, 1244, 1177, 1119, 1034, 833, 820, 762. **HRMS** (ESI-TOF): m/z Calcd for $C_{26}H_{30}O_2Na$ $[M+Na]^+$ 397.2138, found 397.2143.

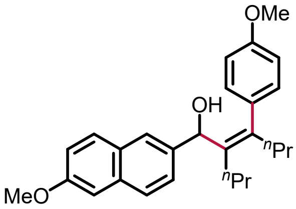

23: (*Z*)-1-(6-methoxynaphthalen-2-yl)-3-(4-methoxyphenyl)-2-propylhex-2-en-1-ol was prepared according to the general procedure A. Colorless oil after flash column chromatography (petroleum ether /EtOAc = 25:1), 60.6 mg, 75%. **^{1}H NMR** (400 MHz, $CDCl_3$) δ 7.75 (s, 1 H), 7.72 (d, J = 8.8 Hz, 1H), 7.64 (d, J = 8.4 Hz, 1H), 7.28 (dd, J_1 = 13.2 Hz, J_2 = 2.0 Hz, 1H), 7.17 – 7.10 (m, 4H), 6.91 – 6.87 (m, 2H), 5.50 (d, J = 3.2 Hz, 1H), 3.91 (s, 3H), 3.81 (s, 3H), 2.40 – 2.32 (m, 2H), 2.19 – 2.11 (m, 1H), 2.00 – 1.92 (m, 1H), 1.70 (d, J = 3.6 Hz, 1H), 1.45 – 1.37 (m, 1H), 1.36 – 1.26 (m, 2H), 1.06 – 0.96 (m, 1H), 0.90 (t, J = 7.4 Hz, 3H), 0.77 (t, J = 7.2 Hz, 3H). **^{13}C NMR** (100 MHz, $CDCl_3$) δ 158.3, 157.5, 141.0, 138.6, 137.3, 134.8, 133.7, 129.8, 129.6, 128.8, 126.5, 125.3, 123.9, 118.8, 113.7, 105.8, 74.1, 55.4, 55.3, 36.8, 30.0, 24.5, 21.3, 15.0, 14.3. **IR**: ν (cm^{-1}) 3487, 2999, 2959, 2932, 2849, 2837, 1634, 1607, 1483, 1464, 1441, 1389, 1283, 1263, 1244, 1217, 1175, 1165, 1150, 1034, 853, 835, 810. **HRMS** (ESI-TOF): m/z Calcd for $C_{27}H_{31}O_2$ $[M-OH]^+$ 387.2319, found 387.2312.

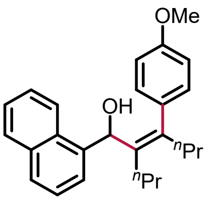

24: (*Z*)-3-(4-methoxyphenyl)-1-(naphthalen-1-yl)-2-propylhex-2-en-1-ol was prepared according to the general procedure A. Sticky colorless oil after flash column chromatography (petroleum ether /EtOAc = 25:1), 45.7 mg, 61%. **^{1}H NMR** (400 MHz, $CDCl_3$) δ 7.81 (t, *J* = 8.4 Hz, 2H), 7.73 (d, *J* = 8.4 Hz, 1H), 7.54 – 7.44 (m, 2H), 7.41 (t, *J* = 7.6 Hz, 1H), 7.36 – 7.30 (m, 3H), 6.96 (d, *J* = 8.4 Hz, 2H), 5.88 (s, 1H),3.83 (s, 3H), 2.46 – 2.42 (m, 1H), 2.32 – 2.25 (m, 1H), 2.11 – 2.04 (m, 1H), 1.95 – 1.88 (m, 1H), 1.80 (s, 1H), 1.33 – 1.26 (m, 2H), 1.15 – 1.06 (m, 1H), 0.87 (t, *J* = 7.4 Hz, 3H), 0.60 (t, *J* = 7.2 Hz, 3H), 0.40 – 0.31 (m, 1H). **^{13}C NMR** (100 MHz, $CDCl_3$) δ 158.6, 141.2, 139.1, 136.7, 134.5, 133.4, 130.9, 129.9, 128.6, 127.6, 125.5, 125.4, 124.1, 123.2, 114.9, 113.8, 71.9, 55.4, 36.6, 30.5, 23.6, 21.4, 14.7, 14.2. **IR**: ν (cm^{-1}) 3318, 2955, 2870, 1609, 1508, 1464, 1285, 1244, 1177, 1043, 982, 831, 783. **HRMS** (ESI-TOF): m/z Calcd for $C_{26}H_{30}O_2Na$ $[M+Na]^+$ 397.2138, found 397.2143.

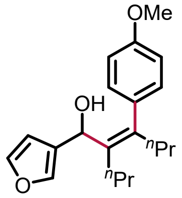

25: (*Z*)-1-(furan-3-yl)-3-(4-methoxyphenyl)-2-propylhex-2-en-1-ol was prepared according to the general procedure A. Sticky colorless oil after flash column chromatography (petroleum ether /EtOAc = 25:1), 28.9 mg, 46%. **^{1}H NMR** (400 MHz, $CDCl_3$) δ 7.37 – 7.28 (m, 2H), 7.02 (d, *J* = 8.0 Hz, 2H), 6.84 (d, *J* = 8.0 Hz, 2H), 6.24 (s, 1H), 5.26 (s, 1H), 3.80 (s, 3H), 2.39 – 2.26 (m, 2H), 2.23 – 2.15 (m, 1H), 2.11 – 2.01 (m, 1H), 1.57 – 1.45 (m, 2H), 1.37 – 1.21 (m, 3H), 0.91 (t, *J* = 7.2 Hz, 3H), 0.87 (t, *J* = 7.2 Hz, 3H). **^{13}C NMR** (100 MHz, $CDCl_3$) δ 158.3, 143.0, 140.1, 139.3, 136.7, 134.4, 129.7, 128.5, 113.6, 109.5, 69.1, 55.4, 36.7, 30.1, 24.8, 21.2, 15.1, 14.2. **IR**: ν (cm^{-1}) 3451, 2959, 2932, 2872, 1609, 1508, 1466, 1285, 1246, 1177, 1159, 1034, 1024, 874, 833. **HRMS** (ESI-TOF): m/z Calcd for $C_{27}H_{31}O_2$ $[M-OH]^+$ 297.1849, found 297.1841.

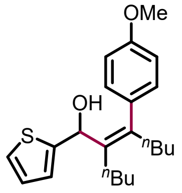

26: (*Z*)-2-butyl-3-(4-methoxyphenyl)-1-(thiophen-2-yl)hept-2-en-1-ol was prepared according to the general procedure A. Colorless oil after flash column chromatography (petroleum ether/EtOAc = 30:1), 35 mg, 49%. **^{1}H NMR** (400 MHz, $CDCl_3$) δ 7.20 (d, *J* = 4.8 Hz, 1H), 7.07 (d, *J* = 8.0 Hz, 2H), 6.95 (t, *J* = 4.4 Hz, 1H), 6.89 – 6.81 (m, 3H), 5.51 (d, *J* = 4.0 Hz, 1H), 3.80 (s, 3H), 2.44 – 2.22 (m, 3H), 2.11 – 2.04 (m, 1H), 1.85 (br, 1H), 1.5 – 1.43 (m, 1H), 1.37 – 1.18 (m, 7H), 0.90 – 0.84 (m, 6H); **^{13}C NMR** (100 MHz, $CDCl_3$) δ 158.3, 148.3, 140.6, 136.7, 134.3, 129.7, 126.7, 124.2, 123.4, 113.6, 71.9, 55.3, 34.4, 33.7, 30.2, 27.7, 23.7, 22.9, 14.1, 14.0. **IR**: ν (cm^{-1})

2955, 2932, 2361, 2342, 1609, 1508, 1285, 1244, 1177, 1036, 833. **HRMS** (ESI-TOF): m/z Calcd for $C_{22}H_{29}OS$ $[M-OH]^+$ 341.1934, found 341.1933.

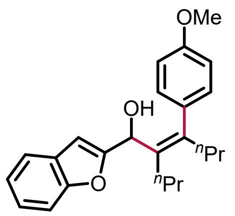

27: (*Z*)-1-(benzofuran-2-yl)-3-(4-methoxyphenyl)-2-propylhex-2-en-1-ol was prepared according to the general procedure A. Sticky colorless oil after flash column chromatography (petroleum ether /EtOAc = 25:1), 52.5 mg, 72%. **^{1}H NMR** (400 MHz, $CDCl_3$) δ 7.52 (d, *J* = 7.2 Hz, 1H), 7.44 (d, *J* = 7.6 Hz, 1H), 7.24 – 7.18 (m, 2H), 7.14 (d, *J* = 8.4 Hz, 2H), 6.86 (d, *J* = 8.4 Hz, 2H), 6.58 (s, 1H), 5.43 (s, 1H), 3.80 (s, 3H), 2.47 – 2.26 (m, 3H), 2.19 – 2.10 (m, 1H), 1.94 (s, 1H), 1.50 (m, 1H), 1.28 (m, 3H), 0.92 – 0.85 (m, 6H). **^{13}C NMR** (100 MHz, $CDCl_3$) δ 159.3, 158.5, 154.9, 142.2, 134.5, 134.1, 129.9, 128.5, 123.8, 122.8, 120.9, 113.6, 111.3, 103.0, 69.9, 55.3, 36.7, 30.4, 24.3, 21.3, 15.0, 14.2. **IR**: ν (cm^{-1}) 3445, 2959, 2932, 2870, 1609, 1508, 1456, 1285, 1246, 1177, 1036, 955, 835, 808. **HRMS** (ESI-TOF): m/z Calcd for $C_{24}H_{27}O_2$ $[M-OH]^+$ 347.2006, found 347.2003.

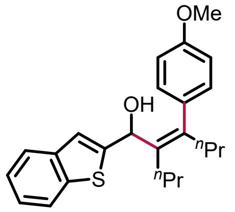

28: (*Z*)-1-(benzo[b]thiophen-2-yl)-3-(4-methoxyphenyl)-2-propylhex-2-en-1-ol was prepared according to the general procedure A. Sticky colorless oil after flash column chromatography (petroleum ether /EtOAc = 25:1), 41.1 mg, 54%. **^{1}H NMR** (400 MHz, $CDCl_3$) δ 7.78 – 7.76 (m, 1H), 7.72 – 7.67 (m, 1H), 7.34 – 7.24 (m, 2H), 7.15 – 7.11 (m, 3H), 6.88 – 6.84 (m, 2H), 5.59 (s, 1H), 3.80 (s, 3H), 2.45 – 2.24 (m, 3H), 2.13 – 2.05 (m, 1H), 1.94 (d, *J* = 4.4 Hz, 1H), 1.56 – 1.51 (m, 1H), 1.41 – 1.26 (m, 3H), 0.94 – 0.87 (m, 6H). **^{13}C NMR** (100 MHz, $CDCl_3$) δ 158.5, 149.1, 141.4, 140.0, 139.7, 136.4, 134.1, 129.7, 124.2, 123.8, 123.4, 122.4, 119.9, 113.7, 72.3, 55.4, 36.7, 30.4, 24.8, 21.2, 15.1, 14.3. **IR**: ν (cm^{-1}) 3468, 2959, 2932, 2870, 1726, 1607, 1508, 1458, 1437, 1285, 1246, 1177, 1105, 1034, 833. **HRMS** (ESI-TOF): m/z Calcd for $C_{24}H_{27}OS$ $[M-OH]^+$ 363.1777, found 363.1769.

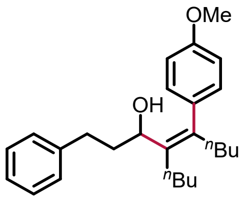

29: (*Z*)-4-butyl-5-(4-methoxyphenyl)-1-phenylnon-4-en-3-ol was prepared according to the general procedure A. Sticky colorless oil after flash column chromatography (petroleum ether /EtOAc = 25:1), 31.9 mg, 42%. **^{1}H NMR** (400 MHz, $CDCl_3$) δ 7.26 – 7.19 (m, 2H), 7.18 – 7.12 (m, 1H), 7.11 – 7.06 (m, 2H), 6.91 (d, *J* = 8.4 Hz, 2H), 6.80 (d, *J* = 8.0 Hz, 2H), 4.20 (t, *J* = 6.8 Hz, 1H), 3.82 (s, 3H), 2.62 – 2.55 (m, 1H), 2.47 – 2.40 (m, 1H), 2.31 – 2.21 (m, 3H), 2.18 – 2.10 (m, 1H), 1.93 – 1.84 (m, 1H), 1.76 – 1.67 (m, 1H), 1.55 – 1.47 (m, 1H), 1.45 – 1.37 (m, 2H), 1.32 – 1.15 (m, 6H), 0.96 (t, *J* = 7.2 Hz, 3H), 0.83 (t, *J* = 7.0 Hz, 3H). **^{13}C NMR** (100 MHz, $CDCl_3$) δ 158.1, 142.3, 139.8, 137.5, 134.8, 129.7, 128.5, 128.4, 125.8, 113.5, 72.6, 55.3, 37.5, 34.4, 33.9, 32.6, 30.4, 26.8, 23.8, 23.0, 14.2. **IR**: ν (cm^{-1}) 3462, 3063, 3028, 2963, 2859, 2835, 1726, 1607, 1574, 1510, 1466, 1456, 1416, 1377, 1287, 1242, 1177, 1105, 1038, 920, 833, 810, 797. **HRMS** (ESI-TOF): m/z Calcd for $C_{26}H_{35}O$ [M-OH]$^+$ 363.2682, found 363.2669.

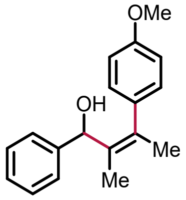

30: (*Z*)-3-(4-methoxyphenyl)-2-methyl-1-phenylbut-2-en-1-ol was prepared according to the general procedure A. Colorless oil after flash column chromatography (petroleum ether/EtOAc = 20:1), 33 mg, 61%. **^{1}H NMR** (400 MHz, $CDCl_3$) δ 7.33 – 7.28 (m, 4H), 7.24 – 7.20 (m, 1H), 7.16 (d, *J* = 8.2 Hz, 2H), 6.88 (d, *J* = 8.2 Hz, 2H), 5.48 (s, 1H), 3.81 (s, 3H), 2.01 (s, 3H), 1.71 (br, 1H), 1.64 (s, 3H); **^{13}C NMR** (100 MHz, $CDCl_3$) δ 158.3, 143.1, 136.4, 135.2, 132.2, 129.2, 128.2, 126.9, 125.8, 113.9, 73.4, 55.4, 21.8, 12.6. **IR**: ν (cm^{-1}) 3412, 2918, 1609, 1514, 1449, 1252, 1177, 1028, 837. **HRMS** (ESI-TOF): m/z Calcd for $C_{18}H_{20}O_2Na$ [M+Na]$^+$ 291.1356, found 291.1369.

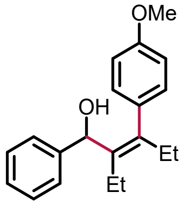

31: (*Z*)-2-ethyl-3-(4-methoxyphenyl)-1-phenylpent-2-en-1-ol was prepared according to the general procedure A. White solid (melting point 79–81 °C) after flash column chromatography (petroleum ether/EtOAc = 20:1), 41 mg, 69%. **^{1}H NMR** (400 MHz, $CDCl_3$) δ 7.30 (m, 4H), 7.22 – 7.19 (m, 1H), 7.15 – 7.11 (m, 2H), 6.91 – 6.87 (m, 2H), 5.39 (s, 1H), 3.81 (s, 3H), 2.48 – 2.31 (m, 2H), 2.19 (m, 1H), 2.03 (m,1H), 1.61 (d, *J* = 3.6 Hz, 1H), 0.91 (t, *J* = 7.6 Hz, 3H), 0.83 (t, *J* = 7.6 Hz, 3H). **^{13}C NMR** (100 MHz, $CDCl_3$) δ 158.3, 143.2, 142.0, 138.0, 134.5, 130.0, 128.1, 126.8, 125.8, 113.7, 74.0, 55.4, 27.5, 20.2, 15.7, 12.8. **IR**: ν (cm^{-1}) 3256, 2963, 2909, 2872, 2835, 1607,

1576, 1508, 1495, 1466, 1450, 1371, 1335, 1300, 1285, 1242, 1180, 1175, 1107, 1057, 1038, 1018, 916, 870, 837, 820, 775. **HRMS** (ESI-TOF): m/z Calcd for $C_{20}H_{24}O_2Na$ $[M+Na]^+$ 319.1669, found 319.1686.

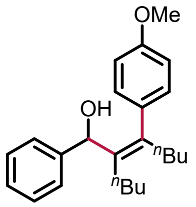

32: (*Z*)-2-butyl-3-(4-methoxyphenyl)-1-phenylhept-2-en-1-ol was prepared according to the general procedure A. White solid (melting point 80–81 °C) after flash column chromatography (petroleum ether/EtOAc = 20:1), 48 mg, 68%. **^{1}H NMR** (400 MHz, $CDCl_3$) δ 7.32 – 7.27 (m, 4H), 7.20 (m, 1H), 7.13 – 7.08 (m, 2H), 6.91 – 6.86 (m, 2H), 5.38 (s,1H), 3.81 (s, 3H), 2.34 (m, 2H), 2.18 – 2.08 (m, 1H), 1.93 (m, 1H), 1.57 (br, 1H), 1.44 – 1.33 (m, 1H), 1.30 – 1.13 (m, 6H), 0.96 – 0.91 (m, 1H), 0.85 (t, J = 6.8 Hz, 3H), 0.80 (t, J = 7.2 Hz, 3H); **^{13}C NMR** (100 MHz, $CDCl_3$) δ 158.3, 143.3, 140.9, 137.2, 134.9, 129.8, 128.1, 126.8, 125.9, 113.7, 74.0, 55.3, 34.5, 33.4, 30.3, 27.4, 23.6, 23.0, 14.2, 13.9. **IR**: v (cm^{-1}) 3312, 2957, 2932, 2874, 2859, 1607, 1574, 1508, 1450, 1379, 1282, 1244, 1192, 1179, 1105, 1045, 1036, 1013, 914, 831, 810. **HRMS** (ESI-TOF): m/z Calcd for $C_{24}H_{32}O_2Na$ $[M+Na]^+$ 375.2295, found 375.2302.

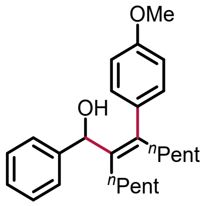

33: (*Z*)-2-pent-3-(4-methoxyphenyl)-1-phenylhept-2-en-1-ol was prepared according to the general procedure A. Colorless oil after flash column chromatography (petroleum ether/EtOAc = 20:1), 27mg, 57%. **^{1}H NMR** (400 MHz, $CDCl_3$) δ 7.29 (d, J = 4.0 Hz, 4H), 7.22 – 7.19 (m, 1H), 7.11 (d, J = 8.2 Hz, 2H), 6.88 (d, J = 8.0 Hz, 2H), 5.38 (s, 1H), 3.81 (s, 3H), 2.38 – 2.29 (m, 2H), 2.15 – 2.08 (m, 1H), 1.97 – 1.89 (m, 1H),1.60 (s, 1H), 1.43 – 1.34 (m, 1H), 1.28 – 1.11 (m, 10H), 1.01 – 0.91 (m, 1H), 0.90 – 0.79 (m, 6H). **^{13}C NMR** (100 MHz, $CDCl_3$) δ 158.3, 143.3, 140.9, 137.3, 134.8, 129.8, 128.1, 126.8, 125.8, 113.7, 74.0, 55.4, 34.7, 32.7, 32.1, 30.9, 27.8, 27.7, 22.7, 22.4, 14.2, 14.1. **IR**: v (cm^{-1}) 3472, 2957, 2930, 2870, 2860, 1734, 1607, 1508, 1466, 1287, 1244, 1175, 1036, 833. **HRMS** (ESI-TOF): m/z Calcd for $C_{26}H_{35}O$ $[M-OH]^+$ 363.2682, found 363.2677.

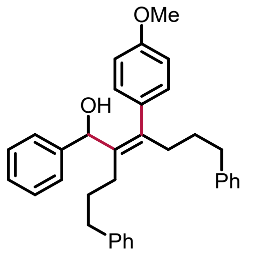

34: (*Z*)-3-(4-methoxyphenyl)-1,6-diphenyl-2-(3-phenylpropyl)hex-2-en-1-ol was prepared according to the general procedure A. Colorless oil after flash column chromatography (petroleum ether/EtOAc = 15:1), 58 mg, 60%. **^{1}H NMR** (400 MHz, $CDCl_3$) δ 7.33 – 7.11 (m, 11H), 7.08 – 7.02 (m, 6H), 6.85 (d, J = 8.1 Hz, 2H), 5.36 (s, 1H), 3.78 (s, 3H), 2.52 – 2.22 (m, 6H), 2.12 – 2.03 (m, 1H), 1.96 – 1.89 (m, 1H), 1.72 – 1.47 (m, 4H), 1.21 – 1.10 (m, 1H); **^{13}C NMR** (100 MHz, $CDCl_3$) δ 158.3, 143.1, 142.43, 142.41, 140.6, 137.4, 134.44, 129.8, 128.6, 128.5, 128.4, 128.3, 128.1, 126.9, 125.8, 125.79, 125.75, 113.8, 73.8, 55.3, 36.6, 36.0, 34.3, 32.8, 29.9, 27.1. **IR**: v (cm^{-1}) 3561, 3458, 3086, 3065, 3001, 2965, 2835, 2529, 2054, 1948, 1886, 1809, 1728, 1611, 1572, 1516, 1441, 1229, 1169, 1157, 1107, 1082, 995, 916, 847, 795. **HRMS** (ESI-TOF): m/z Calcd for $C_{34}H_{35}O$ $[M-OH]^+$ 459.2682, found 459.2676.

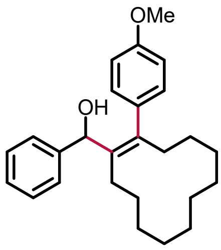

35: (*Z*)-(2-(4-methoxyphenyl)cyclododec-1-en-1-yl)(phenyl)methanol was prepared according to the general procedure A. White solid (melting point 104–105 °C) after flash column chromatography (petroleum ether /EtOAc = 25:1), 43.2 mg, 72%. **^{1}H NMR** (400 MHz, $CDCl_3$) δ 7.32 – 7.26 (m, 4H), 7.21 – 7.18 (m, 1H), 7.15 (d, J = 8.0 Hz, 2H), 6.89 (d, J = 8.0 Hz, 2H), 5.39 (s, 1H), 3.81 (s, 3H), 2.62 – 2.55 (m, 1H), 2.33 – 2.22 (m, 2H), 1.92 – 1.85 (m, 1H), 1.65 (s, 1H), 1.55 – 1.51 (m, 3H), 1.45 – 1.25 (m, 13H). **^{13}C NMR** (100 MHz, $CDCl_3$) δ 158.3, 143.5, 141.7, 138.0, 135.4, 129.8, 128.1, 126.6, 125.6, 113.8, 74.6, 55.4, 32.2, 29.2, 27.2, 26.6, 26.5, 25.8, 25.6, 25.2, 23.0, 22.7. **IR**: v (cm^{-1}) 3308, 2926, 2847, 1607, 1508, 1470, 1287, 1242, 1173, 1107, 1032, 1005, 835. **HRMS** (ESI-TOF): m/z Calcd for $C_{26}H_{33}O$ $[M-OH]^+$ 361.2526, found 361.2524.

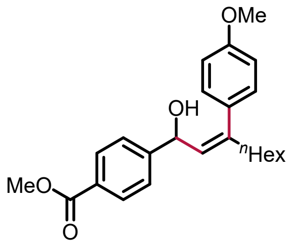

36: methyl (*Z*)-4-(1-hydroxy-3-(4-methoxyphenyl)non-2-en-1-yl)benzoate was prepared according to the general procedure A. Colorless oil after flash column chromatography (petroleum ether/EtOAc = 15:1), rr > 99:1, 31 mg, 50%. **^{1}H NMR** (400 MHz, $CDCl_3$) δ 8.00 (d, *J* = 8.0 Hz, 2H), 7.39 (d, *J* = 8.0 Hz, 2H), 7.13 (d, *J* = 8.0 Hz, 2H), 6.91 (d, *J* = 8.0 Hz, 2H), 5.61 (d, *J* = 9.6 Hz, 1H), 5.21 (dd, J_1 = 8.8 Hz, J_2 = 3.2 Hz, 1H), 3.91 (s, 3H), 3.83 (s, 3H), 2.35 (t, *J* = 6.8 Hz, 2H), 1.82 (d, *J* = 3.6 Hz, 1H), 1.34 – 1.19 (m, 8H), 0.88 – 0.80 (m, 3H); **^{13}C NMR** (100 MHz, $CDCl_3$) δ 158.9, 149.2, 145.3, 132.43, 132.40, 130.0, 129.4, 129.2, 127.8, 126.1, 113.8, 71.2, 55.4, 52.2, 39.5, 31.7, 29.0, 27.9, 22.7, 14.2. **IR**: ν (cm^{-1}) 3505, 2955, 2930, 2857, 1724, 1609, 1510, 1458, 1437, 1281, 1248, 1281, 1115, 1034, 966, 835, 773. **HRMS** (ESI-TOF): m/z Calcd for $C_{24}H_{30}O_4Na$ $[M+Na]^+$ 405.2036, found 405.2048.

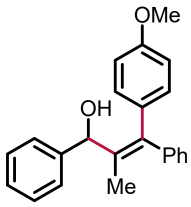

37: (*Z*)-3-(4-methoxyphenyl)-1-phenyl-2-propylhex-2-en-1-ol was prepared according to the general procedure A. Colorless oil after flash column chromatography (petroleum ether/EtOAc = 15:1), rr > 20:1, 40 mg, 60%. **^{1}H NMR** (400 MHz, $CDCl_3$) δ 7.41 – 7.13 (m, 12H), 6.88 – 6.82 (m, 2H), 5.69 (s, 1H), 3.79 (s, 3H), 1.95 (s, 1H), 1.63 (s, 3H) ; **^{13}C NMR** (100 MHz, $CDCl_3$) δ 158.6, 142.9, 142.7, 140.8, 135.0, 134.7, 130.6, 129.6, 128.3, 128.1, 127.1, 126.7, 125.8, 113.8, 73.7, 55.4, 14.3. **IR**: ν (cm^{-1}) 3433, 3059, 3028, 3001, 2953, 2932, 2835, 1607, 1508, 1493, 1464, 1450, 1443, 1410, 1375, 1301, 1283, 1246, 1175, 1109, 1074, 1036, 1009, 835, 822, 793, 772. **HRMS** (ESI-TOF): m/z Calcd for $C_{23}H_{22}O_2Na$ $[M+Na]^+$ 353.1512, found 353.1519.

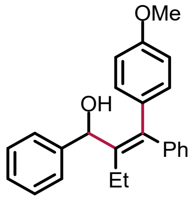

38: (*Z*)-2-((4-methoxyphenyl)(phenyl)methylene)-1-phenylbutan-1-ol was prepared according to the general procedure A. White solid (melting point 103–104 °C) after flash column chromatography (petroleum ether/EtOAc

= 15:1), rr > 20:1, 48 mg, 69%. **^{1}H NMR** (400 MHz, $CDCl_3$) δ 7.41 – 7.36 (m, 2H), 7.35 – 7.16 (m, 10H), 6.82 (d, J = 8.2 Hz, 2H), 5.71 (s, 1H), 3.75 (s, 3H), 2.20 – 2.07 (m, 1H), 1.95 – 1.86 (m, 2H), 0.73 (t, J = 7.6 Hz, 3H); **^{13}C NMR** (100 MHz, $CDCl_3$) δ 158.5, 143.2, 142.9, 141.9, 141.1, 134.9, 130.2, 128.9, 128.3, 128.2, 127.0, 126.6, 125.8, 113.9, 74.1, 55.4, 21.6, 15.3. **IR**: ν (cm^{-1}) 3441, 3063, 2967, 2934, 2361, 1603, 1508, 1450, 1279, 1242, 1182, 1113, 1072, 1020, 912, 843, 812, 791, 764. **HRMS** (ESI-TOF): m/z Calcd for $C_{24}H_{24}O_2Na$ [M+Na]$^+$ 367.1669, found 367.1676.

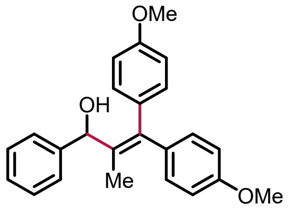

39: 3,3-bis(4-methoxyphenyl)-2-methyl-1-phenylprop-2-en-1-ol was prepared according to the general procedure A. Colorless oil after flash column chromatography (petroleum ether/EtOAc = 10:1), rr > 30:1, 48 mg, 67%. **^{1}H NMR** (400 MHz, $CDCl_3$) δ 7.39 – 7.32 (m, 4H), 7.28 – 7.23 (m, 1H), 7.20 – 7.14 (m, 2H), 7.10 – 7.04 (m, 2H), 6.88 – 6.80 (m, 4H), 5.65 (s, 1H), 3.79 (s, 6H), 1.89 (br, 1H), 1.64 (s, 3H). **^{13}C NMR** (100 MHz, $CDCl_3$) δ 158.6, 158.3, 143.0, 140.4, 135.1, 135.0, 134.4, 130.9, 130.6, 128.3, 127.0, 125.8, 113.8, 113.5, 73.8, 55.4, 55.3, 14.4. **IR**: ν (cm^{-1}) 3474, 2955, 2934, 2835, 1726, 1607, 1510, 1506, 1450, 1279, 1246, 1173, 1036, 833. **HRMS** (ESI-TOF): m/z Calcd for $C_{24}H_{23}O_2$ [M-OH]$^+$ 343.1693, found 343.1686.

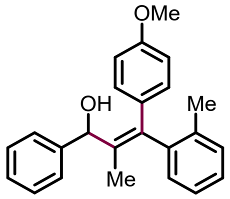

40: (*E*)-3-(4-methoxyphenyl)-2-methyl-1-phenyl-3-(o-tolyl)prop-2-en-1-ol was prepared according to the general procedure A. Colorless oil after flash column chromatography (petroleum ether/EtOAc = 10:1), rr > 20:1, 59 mg, 86%. **^{1}H NMR** (400 MHz, $CDCl_3$) δ 7.43 – 7.37 (m, 1H), 7.36 – 7.29 (m, 3H), 7.25 – 7.07 (m, 7H), 6.80 (t, J = 7.6 Hz, 2H), 5.81 (s, 1H), 3.74 (s, 3H), 2.16 (s, 3H), 1.97 (s, 1H), 1.40 (d, J = 7.2 Hz, 3H); **^{13}C NMR** (100 MHz, $CDCl_3$) δ 158.52, 158.50, 143.1, 142.9, 142.31, 142.25, 140.2, 139.8, 136.0, 135.4, 135.2, 134.9, 133.5, 133.2, 130.5, 130.4, 130.3, 130.2, 129.5, 129.2, 128.4, 128.3, 127.2, 127.1, 127.03, 126.95, 125.94, 125.92, 125.8, 125.7, 113.7, 73.2, 73.1, 55.4, 19.9, 14.18, 14.15. **IR**: ν (cm^{-1}) 3449, 3061, 3028, 2953, 2924, 2837, 2604, 1607, 1508, 1493, 1450, 1285, 1248, 1175, 1113, 1036, 835. **HRMS** (ESI-TOF): m/z Calcd for $C_{24}H_{23}O$ [M-OH]$^+$ 327.1743, found 327.1744.

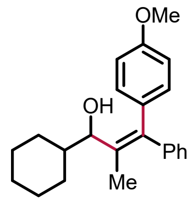

41: (*Z*)-1-cyclohexyl-3-(4-methoxyphenyl)-2-methyl-3-phenylprop-2-en-1-ol was prepared according to the general procedure A. Colorless oil after flash column chromatography (petroleum ether/EtOAc = 15:1), rr > 20:1, 20 mg, 30%. **^{1}H NMR** (400 MHz, $CDCl_3$) δ 7.31 – 7.24 (m, 2H), 7.22 – 7.16 (m, 1H), 7.15 – 7.09 (m, 2H), 7.08 – 7.02 (m, 2H), 6.85 – 6.79 (m, 2H), 4.01 (d, *J* = 9.6 Hz, 1H), 3.79 (s, 3H), 2.02 (d, *J* = 12.8 Hz, 1H), 1.74 (s, 3H), 1.66 – 1.47 (m, 6H), 1.27 – 1.16 (m, 2H), 1.13 – 1.03 (m, 1H), 0.85 – 0.67 (m, 2H); **^{13}C NMR** (100 MHz, $CDCl_3$) δ 158.3, 143.3, 141.2, 135.0, 134.8, 130.5, 129.4, 128.1, 126.6, 113.7, 77.0, 55.3, 41.6, 30.0, 29.2, 26.5, 26.3, 26.0, 13.9. **IR**: ν (cm^{-1}) 3455, 2926, 2851, 1732, 1607, 1508, 1449, 1443, 1283, 1244, 1175, 1036, 1001, 891, 827, 766. **HRMS** (ESI-TOF): m/z Calcd for $C_{23}H_{27}O$ $[M\text{-}OH]^+$ 319.2056, found 319.2060.

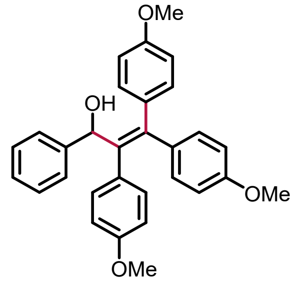

42: 2,3,3-tris(4-methoxyphenyl)-1-phenylprop-2-en-1-ol was prepared according to the general procedure A. Colorless oil after flash column chromatography (petroleum ether/EtOAc = 8:1), 32 mg, 34%. **^{1}H NMR** (400 MHz, $CDCl_3$) δ 7.34 – 7.29 (m, 6H), 7.25 – 7.21 (m, 1H), 6.92 (d, *J* = 8.0 Hz, 2H), 6.84 (d, *J* = 8.4 Hz, 2H), 6.73 (d, *J* = 8.0 Hz, 2H), 6.62 – 6.52 (m, 4H), 5.96 (d, *J* = 7.6 Hz, 1H), 3.82 (s, 3H), 3.70 (s, 3H), 3.68 (s, 3H), 1.92 (d, *J* = 7.6 Hz, 1H); **^{13}C NMR** (100 MHz, $CDCl_3$) δ 158.4, 157.9, 143.3, 139.3, 135.2, 135.0, 132.5, 131.8, 131.1, 130.1, 128.2, 126.9, 126.0, 114.0, 113.3, 113.0, 73.8, 55.4, 55.2, 55.1. **IR**: ν (cm^{-1}) 3478, 2955, 2835, 1607, 1508, 1464, 1287, 1244, 1177, 1034, 831. **HRMS** (ESI-TOF): m/z Calcd for $C_{30}H_{27}O_3$ $[M\text{-}OH]^+$ 435.1955, found 435.1957.

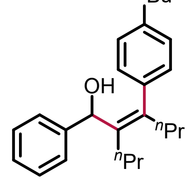

43: (*Z*)-3-(4-(tert-butyl)phenyl)-1-phenyl-2-propylhex-2-en-1-ol was prepared according to the general procedure A. White solid (melting point 73–75 °C) after flash column chromatography (petroleum ether/EtOAc

= 50:1), 44 mg, 63%. **^{1}H NMR** (400 MHz, $CDCl_3$) δ 7.34 – 7.29 (m, 6H), 7.22 – 7.20 (m, 1H), 7.11 (d, J = 8.0 Hz, 2H), 5.37 (s, 1H), 2.36 – 2.32 (m, 2H), 2.14 – 2.07 (m, 1H), 1.95 – 1.88 (m, 1H), 1.47 – 1.28 (m, 13H), 1.05 – 0.94 (m, 1H), 0.89 (t, J = 7.6 Hz, 3H), 0.80 (t, J = 7.6 Hz, 3H); **^{13}C NMR** (100 MHz, $CDCl_3$) δ 149.3, 143.3, 141.4, 139.4, 137.2, 128.4, 128.1, 126.7, 125.8, 125.2, 73.9, 36.7, 34.6, 31.5, 30.0, 24.5, 21.3, 15.0, 14.4. **IR**: ν (cm^{-1}) 3321, 2959, 2870, 1732, 1464, 1450, 1335, 1192, 997, 920, 833. **HRMS** (ESI-TOF): m/z Calcd for $C_{25}H_{33}$ [M-OH]$^+$ 333.2577, found 333.2578.

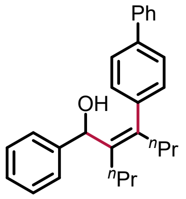

44: (Z)-3-([1,1'-biphenyl]-4-yl)-1-phenyl-2-propylhex-2-en-1-ol was prepared according to the general procedure A. White solid (melting point 66–68 °C) after flash column chromatography (petroleum ether/EtOAc = 40:1), 50 mg, 67%. **^{1}H NMR** (400 MHz, $CDCl_3$) δ 7.58 – 7.49 (m, 4H), 7.42 – 7.35 (m, 2H), 7.31 – 7.19 (m, 7H), 7.18 – 7.13 (m, 1H), 5.38 (s, 1H), 2.36 – 2.32 (m, 2H), 2.13 – 2.06 (m, 1H), 1.95 – 1.87 (m, 1H), 1.59 (br, 1H), 1.42 – 1.24 (m, 3H), 1.02 – 0.91 (m, 1H), 0.85 (t, J = 7.2 Hz, 3H), 0.77 (t, J = 7.2 Hz, 3H); **^{13}C NMR** (100 MHz, $CDCl_3$) δ 143.2, 141.6, 140.95, 140.94, 139.4, 137.6, 129.3, 128.9, 128.1, 127.3, 127.1, 127.0, 126.9, 125.8, 74.0, 36.6, 30.0, 24.5, 21.3, 15.0, 14.3. **IR**: ν (cm^{-1}) 3302, 3028, 2959, 2870, 1487, 1466, 1450, 1248, 1196, 1022, 841, 768. **HRMS** (ESI-TOF): m/z Calcd for $C_{27}H_{29}$ [M-OH]$^+$ 353.2264, found 353.2255.

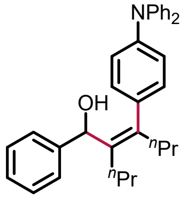

45: (Z)-3-(4-(diphenylamino)phenyl)-1-phenyl-2-propylhex-2-en-1-ol was prepared according to the general procedure A. Colorless oil after flash column chromatography (petroleum ether/EtOAc = 50:1), 50 mg, 54%. **^{1}H NMR** (400 MHz, $CDCl_3$) δ 7.34 – 7.18 (m, 9H), 7.12 – 6.96 (m, 10H), 5.47 (s, 1H), 2.3 – 2.31 (m, 2H), 2.15 – 2.07 (m, 1H), 1.96 – 1.89 (m, 1H), 1.64 (br, 1H), 1.45 – 1.30 (m, 3H), 1.05 – 0.96 (m, 1H), 0.91 (t, J = 7.2 Hz, 3H), 0.80 (t, J = 7.2 Hz, 3H); **^{13}C NMR** (100 MHz, $CDCl_3$) δ 147.9, 146.3, 143.3, 141.1, 137.4, 136.6, 129.6, 129.4, 128.1, 126.8, 125.9, 124.4, 123.6, 122.9, 74.0, 36.6, 30.0, 24.5, 21.4, 15.0, 14.4. **IR**: ν (cm^{-1}) 3453, 3026, 2959, 2930, 2870, 1589, 1506, 1493, 1450, 1375, 1315, 1277, 1177, 1030, 837. **HRMS** (ESI-TOF): m/z Calcd for $C_{33}H_{34}N$ [M-OH]$^+$ 444.2686, found 444.2682.

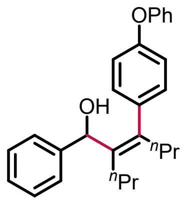

46: (*Z*)-3-(4-phenoxyphenyl)-1-phenyl-2-propylhex-2-en-1-ol was prepared according to the general procedure A. White solid (melting point 57-59 °C) after flash column chromatography (petroleum ether/EtOAc = 30:1), 50 mg, 65%. ^{1}**H NMR** (400 MHz, $CDCl_3$) δ 7.37 – 7.28 (m, 6H), 7.25 – 7.19 (m, 1H), 7.17 – 7.07 (m, 3H), 7.05 – 7.00 (m, 2H), 6.99 – 6.95 (m, 2H), 5.40 (s, 1H), 2.41 – 2.29 (m, 2H), 2.16 – 2.09 (m, 1H), 1.98 – 1.90 (m, 1H), 1.62 (s, 1H), 1.47 – 1.35 (m, 1H), 1.47 – 1.26 (m, 2H), 1.07 – 0.94 (m, 1H), 0.90 (t, *J* = 7.2 Hz, 3H), 0.81 (t, *J* = 7.2 Hz, 3H); ^{13}**C NMR** (100 MHz, $CDCl_3$) δ 157.2, 156.0, 143.2, 140.6, 137.7, 137.4, 130.1, 129.9, 128.1, 126.9, 125.8, 123.4, 119.1, 118.6, 74.0, 36.7, 30.0, 24.5, 21.3, 15.0, 14.3. **IR**: ν (cm^{-1}) 3331, 3061, 3030, 2959, 2928, 2870, 2727, 1589, 1503, 1489, 1468, 1450, 1377, 1331, 1271, 1240, 1192, 1167, 1099, 1072, 1024 , 1015, 997, 918, 887, 872, 853, 768. **HRMS** (ESI-TOF): m/z Calcd for $C_{27}H_{29}O$ [M-OH]$^+$ 369.2213, found 369.2206.

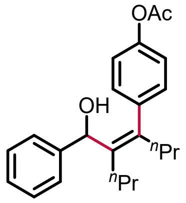

47: (*Z*)-4-(5-(hydroxy(phenyl)methyl)oct-4-en-4-yl)phenyl acetate was prepared according to the general procedure A. White solid (melting point 70–72 °C) after flash column chromatography (petroleum ether /EtOAc = 25:1), 50.7 mg, 72%. ^{1}**H NMR** (400 MHz, $CDCl_3$) δ 8.02 (d, *J* = 8.0 Hz, 2H), 7.45 – 7.27 (m, 4H), 7.26 – 7.19 (m, 3H), 5.28 (s, 1H), 3.91 (s, 3H), 2.43 – 2.31 (m, 2H), 2.18 – 2.11 (m, 1H), 2.01 – 1.93 (m, 1H), 1.63 (s, 1H), 1.46 – 1.37 (m, 1H), 1.31 – 1.22 (m, 2H), 1.05 – 0.97 (m, 1H), 0.90 – 0.80 (m, 6H). ^{13}**C NMR** (100 MHz, $CDCl_3$) δ 167.1, 147.8, 142.9, 140.3, 138.1, 129.7, 129.0, 128.6, 128.2, 127.0, 125.7, 73.9, 52.3, 36.4, 29.9, 24.4, 21.2, 15.0, 14.3. **IR**: ν (cm^{-1}) 3335, 3013, 2959, 2930, 2870, 2837, 1607, 1508, 1464, 1450, 1377, 1333, 1287, 1246, 1175, 1034, 1013, 997, 831. **HRMS** (ESI-TOF): m/z Calcd for $C_{23}H_{29}O_3$ [M+H]$^+$ 353.2111, found 353.2103.

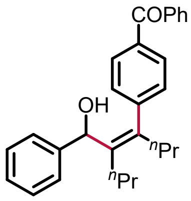

48: (*Z*)-(4-(5-(hydroxy(phenyl)methyl)oct-4-en-4-yl)phenyl)(phenyl)methanone was prepared according to the general procedure A. White solid (melting point 110 °C) after flash column chromatography (petroleum ether/EtOAc = 20:1), 38 mg, 48%. ^{1}**H NMR** (400 MHz, $CDCl_3$) δ 7.80 – 7.77 (m, 4H), 7.58 – 7.54 (m, 1H), 7.48

– 7.44 (m, 2H), 7.33 – 7.18 (m, 7H), 5.32 (s, 1H), 2.43 – 2.31 (m, 2H), 2.16 – 2.09 (m, 1H), 1.98 – 1.91 (m, 1H), 1.65 (s, 1H), 1.45 – 1.37 (m, 1H), 1.31 – 1.25 (m, 2H), 1.03 – 0.95 (m, 1H), 0.87 (t, *J* = 7.2 Hz, 3H), 0.80 (t, *J* = 7.2 Hz, 3H). **^{13}C NMR** (100 MHz, $CDCl_3$) *δ* 196.6, 147.4, 142.9, 140.4, 138.1, 137.8, 135.9, 132.5, 130.3, 130.2, 128.9, 128.4, 128.2, 127.1, 125.8, 74.0, 36.4, 30.0, 24.4, 21.3, 15.0, 14.3. **IR**: ν (cm^{-1}) 3455, 2959, 2870, 1641, 1601, 1447, 1398, 1319, 1287, 1177, 1049, 1030, 926, 854, 802. **HRMS** (ESI-TOF): m/z Calcd for $C_{28}H_{29}O$ $[M\text{-}OH]^+$ 381.2213, found 381.2211.

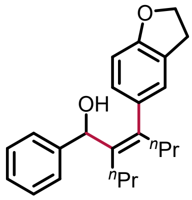

49: (*Z*)-3-(2,3-dihydrobenzofuran-5-yl)-1-phenyl-2-propylhex-2-en-1-ol was prepared according to the general procedure A. Sticky colorless oil after flash column chromatography (petroleum ether /EtOAc = 25:1), 37.6 mg, 56%. **^{1}H NMR** (400 MHz, $CDCl_3$) *δ* 7.26 – 7.21 (m, 4H), 7.23 – 7.18 (m, 1H), 6.97 (s, 1H), 6.91 (d, *J* =6.8 Hz, 1H), 6.87 (d, *J* = 8.0 Hz, 1H), 5.41 (s, 1H), 4.57 (t, *J* = 8.8 Hz, 2H), 3.21 (t, *J* = 8.4 Hz, 2H), 2.38 – 2.26 (m, 2H), 2.07 (td, *J* = 11.6, 4.8 Hz, 1H), 1.88 (td, *J* = 11.6, 4.8 Hz, 1H), 1.41 – 1.31 (m, 1H), 1.30 – 1.20 (m, 3H), 1.02 – 0.92 (m, 1H), 0.85 (t, *J* = 7.2 Hz, 3H), 0.77 (t, *J* = 7.2 Hz, 3H). **^{13}C NMR** (100 MHz, $CDCl_3$) *δ* 158.8, 143.4, 141.3, 137.3, 134.7, 128.4, 128.1, 127.0, 126.8, 125.8, 125.3, 108.9, 74.0, 71.3, 36.9, 30.03, 29.97, 24.5, 21.3, 15.0, 14.3. **IR**: ν (cm^{-1}) 3499, 3026, 2959, 2930, 2870, 1726, 1605, 1489, 1449, 1377, 1281, 1234, 1186, 1107, 1030, 984, 943, 822. **HRMS** (ESI-TOF): m/z Calcd for $C_{23}H_{27}O$ $[M\text{-}OH]^+$ 319.2056, found 319.2058.

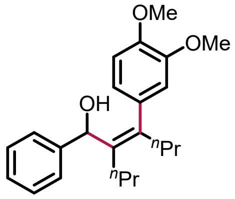

50: (*Z*)-3-(3,4-dimethoxyphenyl)-1-phenyl-2-propylhex-2-en-1-ol was prepared according to the general procedure A. Colorless oil after flash column chromatography (petroleum ether/EtOAc = 15:1), 46 mg, 64%. **^{1}H NMR** (400 MHz, $CDCl_3$) *δ* 7.34 – 7.28 (m, 4H), 7.23 – 7.19 m, 1H), 6.83 (d, *J* = 8.0 Hz, 1H), 6.73 (dd, J_1 = 8.0 Hz, J_2 = 2.0 Hz, 1H), 6.68 (d, *J* = 2.0 Hz, 1H), 5.40 (s, 1H), 3.88 (s, 3H), 3.82 (s, 3H), 2.36 – 2.32 (m, 2H), 2.17 – 2.09 (m, 1H), 2.00 – 1.92 (m, 1H), 1.62 (s, *J* = 4.0 Hz, 1H), 1.48 – 1.36 (m, 1H), 1.34 – 1.26 (m, 2H), 1.09 – 0.97 (m, 1H), 0.89 (t, *J* = 7.2 Hz, 3H), 0.82 (t, *J* = 7.2 Hz, 3H). **^{13}C NMR** (100 MHz, $CDCl_3$) *δ* 148.6, 147.7, 143.4, 140.8, 137.2, 135.0, 128.1, 126.9, 125.9, 120.9, 112.1, 110.9, 74.1, 56.0, 36.7, 30.1, 24.5, 21.4, 15.0, 14.3. **IR**:

ν (cm^{-1}) 3524, 2959, 2870, 1512, 1464, 1252, 1163, 1140, 1030, 810, 762. **HRMS** (ESI-TOF): m/z Calcd for $C_{23}H_{30}O_3Na$ $[M+Na]^+$ 377.2087, found 377.2100.

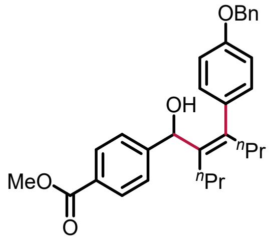

51: methyl (*Z*)-4-(3-(4-(benzyloxy)phenyl)-1-hydroxy-2-propylhex-2-en-1-yl) benzoate was prepared according to the general procedure A. Colorless oil after flash column chromatography (petroleum ether/EtOAc = 15:1), 64 mg, 70%. **^{1}H NMR** (400 MHz, $CDCl_3$) *δ* 7.96 (dd, J_1 = 8.4 Hz, J_2 = 1.6 Hz, 2H), 7.39 (m, 7H), 7.15 – 7.08 (m, 2H), 7.00 – 6.94 (m, 2H), 5.43 (s, 1H), 5.06 (s, 2H), 3.90 (s, 3H), 2.39 – 2.27 (m, 2H), 2.13 – 2.05 (m, 1H),1.93 – 1.85 (m, 1H), 1.65 (br, 1H), 1.43 – 1.27 (m, 3H), 0.97 – 0.92 (m, 1H), 0.88 (t, *J* = 7.6 Hz, 3H), 0.78 (t, *J* = 7.2 Hz, 3H). **^{13}C NMR** (100 MHz, $CDCl_3$) *δ* 167.3, 157.7, 148.7, 141.5, 137.1, 137.0, 134.8, 129.8, 129.4, 128.7, 128.6, 128.1, 127.7, 125.8, 114.7, 73.8, 70.2, 52.1, 36.7, 29.9, 24.4, 21.2, 14.9, 14.3. **IR**: ν (cm^{-1}) 3501, 3034, 2959, 2932, 2870, 1721, 1705, 1609, 1576, 1508, 1456, 1437, 1410, 1379, 1310, 1281, 1240, 1177, 1109, 1020, 835, 810. **HRMS** (ESI-TOF): m/z Calcd for $C_{30}H_{33}O_3$ $[M-OH]^+$ 441.2424, found 441.2419.

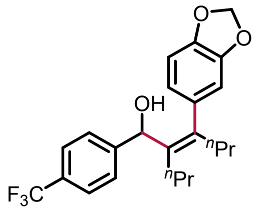

52: (*Z*)-3-(benzo[d][1,3]dioxol-5-yl)-2-propyl-1-(4-(trifluoromethyl)phenyl)hex-2-en-1-ol was prepared according to the general procedure A. Colorless oil after flash column chromatography (petroleum ether/EtOAc = 20:1), 48mg, 59%. **^{1}H NMR** (400 MHz, $CDCl_3$) *δ* 7.54 (d, *J* = 8.0 Hz, 2H), 7.41 (d, *J* = 8.0 Hz, 2H), 6.79 (d, *J* = 7.6 Hz, 1H), 6.68 (s, 1H), 6.63 (d, *J* = 7.6 Hz, 1H), 5.96 (s, 2H), 5.45 (s, 1H), 2.38 – 2.25 (m, 2H), 2.12 – 2.04 (m, 1H), 1.90 – 1.82 (m, 1H), 1.67 (s, 1H), 1.43 – 1.25 (m, 3H), 1.04 – 0.93 (m, 1H), 0.89 (t, *J* = 7.2 Hz, 3H), 0.81 (t, *J* = 7.2 Hz, 3H); **^{13}C NMR** (100 MHz, $CDCl_3$) *δ* 147.7, 147.2, 146.4, 141.7, 137.1, 136.0, 129.0 (q, *J* = 32.0 Hz), 126.1, 125.0 (q, *J* = 3.9 Hz), 124.4 (q, *J* = 270.3 Hz), 121.7, 109.2, 108.3, 101.2, 73.6, 36.7, 29.9, 24.5, 21.2, 14.9, 14.3. **IR**: ν (cm^{-1}) 3439, 2961, 2934, 2874, 1724, 1618, 1504, 1487, 1433, 1327, 1238, 1165, 1126, 1069, 1042, 1018, 937, 860, 818. **HRMS** (ESI-TOF): m/z Calcd for $C_{23}H_{24}F_3O_2$ $[M-OH]^+$ 389.1723, found 389.1725.

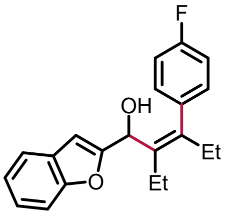

53: (*Z*)-1-(benzofuran-2-yl)-2-ethyl-3-(4-fluorophenyl)pent-2-en-1-ol was prepared according to the general procedure A. White solid (melting point 72–73 °C)after flash column chromatography (petroleum ether /EtOAc = 25:1), 40.2 mg, 62%. **^{1}H NMR** (400 MHz, $CDCl_3$) δ 7.53 (d, *J* = 7.6 Hz, 1H), 7.44 (d, *J* = 8.0 Hz, 1H), 7.25 – 7.18 (m, 4H), 7.03 (t, *J* = 8.4 Hz, 2H), 6.59 (s, 1H), 5.36 (s, 1H), 2.53 – 2.20 (m, 4H), 1.93 (s, 1H), 0.98 – 0.89 (m, 6H). **^{13}C NMR** (100 MHz, $CDCl_3$) δ 161.9 (*J* = 244.0 Hz), 158.9, 154.9, 142.7, 137.4 (*J* = 3.4 Hz), 135.7, 130.5 (*J* = 7.7 Hz), 128.4, 123.9, 122.9, 121.0, 115.2 (*J* = 21.1 Hz), 111.3, 103.0, 69.8, 27.6, 20.6, 15.4, 12.8. **IR**: ν (cm^{-1}) 3294, 2959, 2932, 2874, 2548, 2170, 1732, 1653, 1601, 1549, 1508, 1456, 1435, 1383, 1292, 1221, 1126, 1076, 1040, 959, 837, 820, 791, 772. **HRMS** (ESI-TOF): m/z Calcd for $C_{21}H_{20}FO$ [M-OH]$^+$ 307.1493, found 307.1486.

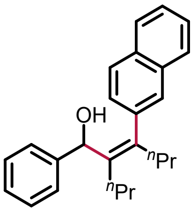

54: (*Z*)-3-(naphthalen-2-yl)-1-phenyl-2-propylhex-2-en-1-ol was prepared according to the general procedure A. White solid (melting point 99–100 °C) after flash column chromatography (petroleum ether/EtOAc = 40:1), 35 mg, 50%. **^{1}H NMR** (400 MHz, $CDCl_3$) δ 7.81 – 7.77 (m, 3H), 7.62 (s, 1H), 7.48 – 7.40 (m, 2H), 7.32 (d, *J* = 8.8 Hz, 1H), 7.29 – 7.21 (m, 4H), 7.18 – 7.15 (m, 1H), 5.38 (s, 1H), 2.48 – 2.36 (m, 2H), 2.19 – 2.11 (m, 1H), 2.01 – 1.93 (m, 1H), 1.63 (s, 1H), 1.48 – 1.38 (m, 1H), 1.32 – 1.23 (m, 2H), 1.06 – 0.95 (m, 1H), 0.88 – 0.79 (m, 6H); **^{13}C NMR** (100 MHz, $CDCl_3$) δ 143.1, 141.1, 140.0, 137.7, 133.3, 132.2, 128.0, 127.96, 127.95, 127.7, 127.5, 127.1, 126.8, 126.2, 125.81, 125.80, 74.0, 36.6, 29.9, 24.4, 21.3, 15.0, 14.3. **IR**: ν (cm^{-1}) 3321, 3059, 3028, 2957, 2928, 2870, 1495, 1470, 1452, 1194, 1032, 1005, 858, 822. **HRMS** (ESI-TOF): m/z Calcd for $C_{25}H_{28}ONa$ [M+Na]$^+$ 367.2032, found 367.2041.

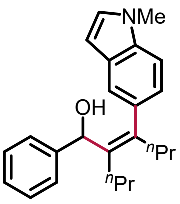

55: (*Z*)-3-(1-methyl-1H-indol-5-yl)-1-phenyl-2-propylhex-2-en-1-ol was prepared according to the general procedure A. White solid (melting point 116–118 °C) after flash column chromatography (petroleum ether /EtOAc = 25:1), 59.7 mg, 86%. **^{1}H NMR** (400 MHz, $CDCl_3$) *δ* 7.39 (s, 1H), 7.30 – 7.19 (m, 5H), 7.16 – 7.12 (m, 1H), 7.04 – 7.00 (m, 2H), 6.41 (d, *J* = 3.2 Hz, 1H), 5.39 (s, 1H), 3.74 (s, 3H), 2.42 – 2.30 (m, 2H), 2.09 (td, *J* = 12.4, 4.8 Hz, 1H), 1.92 (dt, *J* = 12.0, 5.2 Hz, 1H), 1.56 (br, 1H), 1.43 – 1.32 (m, 1H), 1.30 – 1.21 (m, 2H), 1.03 – 0.93 (m, 1H), 0.83 (t, *J* = 7.2 Hz, 3H) , 0.78 (t, *J* = 7.2 Hz, 3H). **^{13}C NMR** (100 MHz, $CDCl_3$) *δ* 143.5, 142.3, 137.1, 135.7, 133.6, 129.3, 128.4, 128.0, 126.6, 125.9, 122.9, 120.6, 109.0, 101.0, 74.1, 37.2, 33.0, 30.0, 24.5, 21.4, 15.0, 14.4. **IR**: v (cm^{-1}) 3314, 3026, 2955, 2868, 1603, 1514, 1487, 1450, 1329, 1242, 1192, 1078, 1024, 1105, 881, 806, 766. **HRMS** (ESI-TOF): m/z Calcd for $C_{24}H_{28}N$ $[M-OH]^+$ 330.2216, found 330.2218.

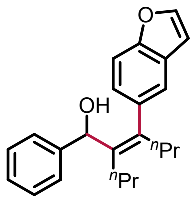

56: (*Z*)-3-(benzofuran-5-yl)-1-phenyl-2-propylhex-2-en-1-ol was prepared according to the general procedure A. White solid (melting point 94-95 °C) after flash column chromatography (petroleum ether/EtOAc = 30:1), 36 mg, 53%. **^{1}H NMR** (400 MHz, $CDCl_3$) *δ* 7.59 (s, 1H), 7.44 (d, *J* = 8.4 Hz, 1H), 7.38 (s, 1H), 7.30 – 7.21 (m, 4H), 7.19 – 7.15 (m, 1H), 7.09 (dd, J_1 = 8.4 Hz, J_2 = 2.0 Hz, 1H), 6.71 (s, 1H), 5.34 (s, 1H), 2.42 – 2.30 (m, 2H), 2.15 – 2.08 (m, 1H), 1.97 – 1.90 (m, 1H), 1.59 (br, 1H), 1.44-1.36 (m, 1H), 1.32 – 1.23(m, 2H), 1.06– 0.92 (m, 1H), 0.85 (t, *J* = 7.2 Hz, 3H), 0.79 (t, *J* = 7.2 Hz, 3H); **^{13}C NMR** (100 MHz, $CDCl_3$) *δ* 153.9, 145.4, 143.3, 141.3, 137.6, 137.1, 128.1, 127.5, 126.8, 125.8, 125.3, 121.1, 111.2, 106.7, 74.0, 37.1, 30.0, 24.5, 21.3, 15.0, 14.3. **IR**: v (cm^{-1}) 3316, 3026, 2957, 2928, 2870, 1603, 1533, 1495, 1466, 1450, 1431, 1381, 1246, 1111, 1026, 1005, 943, 891, 883, 844, 824, 814, 772. **HRMS** (ESI-TOF): m/z Calcd for $C_{23}H_{25}O$ $[M-OH]^+$ 317.1900, found 317.1896.

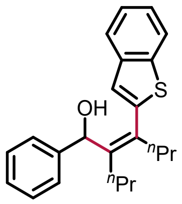

57: (*Z*)-3-(benzo[b]thiophen-2-yl)-1-phenyl-2-propylhex-2-en-1-ol was prepared according to the general procedure A. White solid (melting point 94–96 °C) after flash column chromatography (petroleum ether/EtOAc = 40:1), 30 mg, 42%. **^{1}H NMR** (400 MHz, $CDCl_3$) *δ* 7.80 (d, *J* = 7.6 Hz, 1H), 7.71 (d, *J* = 8.0 Hz, 1H), 7.40 – 7.20 (m, 7H), 7.09 (s, 1H), 5.75 (s, 1H), 2.43 (t, *J* = 7.8 Hz, 2H), 2.19 – 2.12 (m, 1H), 2.02 – 1.94 (m, 1H), 1.76 (s, *J* = 3.6 Hz, 1H), 1.49 – 1.36 (m, 3H), 1.07 – 0.98 (m, 1H), 0.93 (t, *J* = 7.2 Hz, 3H), 0.82 (t, *J* = 7.2 Hz, 3H). **^{13}C**

NMR (100 MHz, $CDCl_3$) δ 144.3, 142.6, 142.1, 139.99, 139.98, 133.6, 128.2, 127.0, 125.9, 124.4, 124.1, 123.4, 122.6, 122.2, 73.9, 36.9, 30.4, 24.3, 21.7, 15.0, 14.2. **IR**: ν (cm^{-1}) 3304, 3059, 3028, 2959, 2930, 2872, 1603, 1495, 1470, 1435, 1377, 1337, 1304, 1250, 1194, 1173, 1153, 1113, 1032, 1022, 984, 935, 920, 910, 858, 833, 760. **HRMS** (ESI-TOF): m/z Calcd for $C_{23}H_{25}S$ $[M-OH]^+$ 333.1671, found 333.1674.

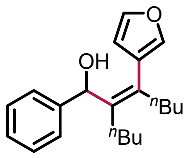

58: (*Z*)-2-butyl-3-(furan-3-yl)-1-phenylhept-2-en-1-ol was prepared according to the general procedure A. White solid (melting point 42–43 °C) after flash column chromatography (petroleum ether/EtOAc = 40:1), 49 mg, 78%. **^{1}H NMR** (400 MHz, $CDCl_3$) δ 7.41 (t, *J* = 1.6 Hz, 1H), 7.35 – 7.30 (m, 4H), 7.29 (s, 1H), 7.25 – 7.20 (m, 1H), 6.35 (s, 1H), 5.67 (s, 1H), 2.37 – 2.25 (m, 2H), 2.17 – 2.09 (m, 1H), 1.97 – 1.89 (m, 1H), 1.70 (br, 1H), 1.44 – 1.13 (m, 7H), 1.04 – 0.93 (m, 1H), 0.90 – 0.87 (m, 3H), 0.80 (t, *J* = 7.2 Hz, 3H); **^{13}C NMR** (100 MHz, $CDCl_3$) δ 143.2, 142.8, 139.6, 138.6, 131.6, 128.2, 127.0, 126.0, 125.1, 111.8, 74.0, 33.7, 33.4, 30.8, 27.8, 23.6, 22.9, 14.2, 13.9. **IR**: ν (cm^{-1}) 3291, 3061, 3028, 2955, 2932, 2872, 2860, 1603, 1495, 1468, 1450, 1379, 1331, 1246, 1194, 1159, 1049, 1024, 1013, 874, 785. **HRMS** (ESI-TOF): m/z Calcd for $C_{21}H_{27}O$ $[M-OH]^+$ 295.2056, found 295.2054.

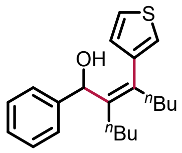

59: (*Z*)-2-butyl-1-phenyl-3-(thiophen-3-yl)hept-2-en-1-ol was prepared according to the general procedure A. Colorless oil after flash column chromatography (petroleum ether/EtOAc = 40:1), 50 mg, 76%. **^{1}H NMR** (400 MHz, $CDCl_3$) δ 7.31 – 7.28 (m, 5H), 7.26 – 7.21 (m, 1H), 7.02 (d, *J* = 2.0 Hz, 1H), 6.98 (d, *J* = 4.8 Hz, 1H), 5.49 (s, 1H), 2.37 – 2.33 (m, 2H), 2.14 (td, J_1 = 13.2 Hz, J_2 = 4.8 Hz, 1H), 1.94 (td, J_1 = 12.4 Hz, J_2 = 5.2 Hz, 1H), 1.66 (s, 1H), 1.45 – 1.14 (m, 7H), 1.03 – 0.92 (m, 1H), 0.86 (t, *J* = 6.8 Hz, 3H), 0.80 (t, *J* = 7.2 Hz, 3H); **^{13}C NMR** (100 MHz, $CDCl_3$) δ 143.2, 142.4, 138.4, 136.1, 128.7, 128.2, 126.9, 125.9, 125.2, 121.8, 74.0, 34.1, 33.3, 30.6, 27.7, 23.6, 22.9, 14.2, 13.9. **IR: ν (cm^{-1})** 3306, 2955, 2930, 2872, 2859, 1605, 1493, 1468, 1450, 1379, 1331, 1256, 1194, 1173, 1103, 1013, 910, 849, 785. **HRMS** (ESI-TOF): m/z Calcd for $C_{21}H_{27}S$ $[M-OH]^+$ 311.1828, found 311.1835.

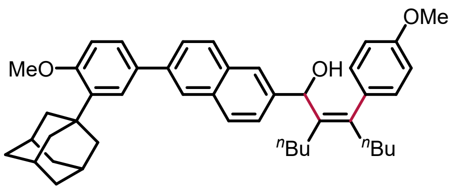

60:(*Z*)-1-(6-(3-adamantan-1-yl)-4-methoxyphenyl)naphthalen-2-yl)-2-butyl-3-(4-methoxyphenyl)hept-2-en-1-ol was prepared according to the general procedure A. White solid (melting point 167–168 °C) after flash column chromatography (petroleum ether /EtOAc = 25:1), 65.6 mg, 51%. **^{1}H NMR** (400 MHz, $CDCl_3$) δ 7.93 (s, 1H), 7.86 (d, *J* = 8.4 Hz, 1H), 7.83 (s, 1H), 7.78 (d, *J* = 8.8 Hz, 1H), 7.70 (dd, J_1 = 8.8 Hz, J_2 = 2.0 Hz, 1H), 7.57 (d, *J* = 2.4 Hz, 1H), 7.54 – 7.50 (m, 1H), 7.30 (dd, J_1 = 8.4 Hz, J_2 = 2.0 Hz,1H), 7.19 – 7.15 (m, 2H), 6.98 (d, *J* = 8.4 Hz, 1H), 6.92 – 6.86 (m, 2H), 5.53 (d, *J* = 2.8 Hz, 1H), 3.89 (s, 3H), 3.82 (s, 3H), 2.43 – 2.36 (m, 2H), 2.21 – 2.15 (m, 6H), 2.09 (s, 3H), 2.01 – 1.93 (m, 1H), 1.82 – 1.77 (m, 5H), 1.73 (d, *J* = 3.6 Hz, 1H), 1.44 – 1.40 (m, 2H), 1.30 – 1.25 (m, 4H), 1.20 – 1.14 (m, 2H), 1.03 – 0.94 (m, 2H), 0.87 (t, *J* = 6.8 Hz, 3H), 0.76 (t, *J* = 7.2 Hz, 3H). **^{13}C NMR** (100 MHz, $CDCl_3$) δ 168.2, 158.6, 158.3, 141.3, 140.6, 139.0, 138.8, 137.1, 134.9, 133.4, 133.0, 132.2, 132.1, 1313, 129.9, 129.0, 128.5, 127.8, 126.0, 125.9, 125.7, 125.1, 124.9, 123.8, 113.8, 112.2, 74.3, 55.4, 55.3, 52.8, 40.8, 37.31, 37.28, 34.6, 33.4, 30.4, 29.3, 27.4, 23.5, 23.0, 14.2, 13.9. **IR**: ν (cm^{-1}) 3310, 2934, 2857, 1701, 1655, 1607, 1506, 1485, 1452, 1265, 1227, 1161, 1121, 1034, 926, 893, 854, 808, 789. **HRMS** (ESI-TOF): m/z Calcd for $C_{45}H_{53}O_2$[M-OH]$^+$ 625.4040, found 625.4044.

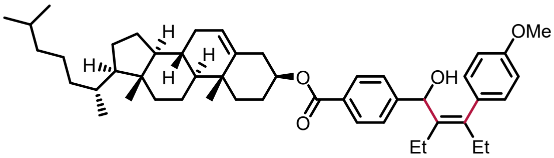

61: (3*S*,8*S*,9*S*,10*R*,13*R*,14*S*,17*R*)-10,13-dimethyl-17-((*R*)-6-methylheptan-2-yl)-2,3,4,7,8,9,10,11,12,13,14,15,16,17-tetradecahydro-1H-cyclopenta[a]phenanthren-3-yl 4-((*Z*)-2-ethyl-1-hydroxy-3-(4-methoxyphenyl)pent-2-en-1-yl)benzoate was prepared according to the general procedure A. Sticky colorless oil after flash column chromatography (petroleum ether /EtOAc = 25:1), 83.6 mg, 59%. **^{1}H NMR** (400 MHz, $CDCl_3$) δ 7.96 (d, *J* = 8.0 Hz, 2H), 7.37 (d, *J* = 8.0 Hz, 2H), 7.12 (d, *J* = 8.4 Hz, 2H), 6.90 (d, *J* = 6.8 Hz, 2H), 5.42 (s, 2H), 4.84 – 4.82 (m, 1H), 3.82 (s, 3H), 2.45 (d, *J* = 7.6 Hz, 2H), 2.37 (m, 2H), 2.18 – 2.12 (m, 1H), 2.03 – 1.96 (m, 4H), 1.93 – 1.74 (m, 4H), 1.70 – 1.65 (m, 3H), 1.52 – 1.45 (m, 5H), 1.35 – 1.33 (m, 2H), 1.25 – 1.11 (m, 10H), 1.06 (s, 3H), 1.03 – 0.99 (m, 3H), 0.91 (t, *J* = 6.4 Hz,3H), 0.86 (d, *J* = 8.0 Hz, 6H), 0.80 (t, *J* = 7.6 Hz, 3H), 0.69 (s, 3H).**^{13}C NMR** (100 MHz, $CDCl_3$) δ 166.1, 158.5, 148.4, 142.7, 139.9, 137.6, 134.3, 129.9, 129.42, 129.35, 125.8, 122.9, 113.9, 74.6, 73.9, 56.8, 56.3, 55.4, 50.2, 42.5, 39.9, 39.7, 38.4, 37.2, 36.8, 36.3, 36.0, 32.1, 32.0, 28.4, 28.2, 28.0, 27.6, 24.5, 24.0, 23.0, 22.7, 21.2, 20.2, 19.5, 18.9, 15.8, 12.8, 12.0. **IR**:

v (cm^{-1}) 3491, 3032, 2870, 1717, 1699, 1609, 1576, 1508, 1466, 1410, 1373, 1319, 1275, 1248, 1177, 1119, 1107, 1088, 1040, 995, 978, 947, 926, 880, 833, 799, 777. **HRMS** (ESI-TOF): m/z Calcd for $C_{48}H_{67}O_3$[M-OH]$^+$ 691.5085, found 691.5086.

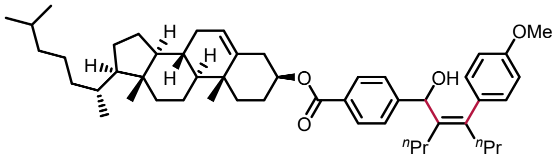

62: (3*S*,8*S*,9*S*,10*R*,13*R*,14*S*,17*R*)-10,13-dimethyl-17-((*R*)-6-methylheptan-2-yl)-2,3,4,7,8,9,10,11,12,13,14,15,16,17-tetradecahydro-1H-cyclopenta[a]phenanthren-3-yl 4-((*Z*)-2-ethyl-1-hydroxy-3-(4-methoxyphenyl)pent-2-en-1-yl)benzoate was prepared according to the general procedure A. Sticky colorless oil after flash column chromatography (petroleum ether /EtOAc = 25:1), 119.4 mg, 81%. **^{1}H NMR** (400 MHz, $CDCl_3$) δ 7.96 (d, *J* = 8.0 Hz, 2H), 7.36 (d, *J* = 8.0 Hz, 2H), 7.10 (d, *J* = 8.4 Hz, 2H), 6.88 (d, *J* = 8.4 Hz, 2H), 5.42 (s, 2H), 4.85 – 4.84 (m, 1H), 3.81 (s, 3H), 2.45 (d, *J* = 7.6 Hz, 2H), 2.39 – 2.30 (m, 2H), 2.20 – 2.11 (m, 1H), 2.13 – 1.97 (m, 4H), 1.92 – 1.82 (m, 4H), 1.74 – 1.59 (m, 3H), 1.56 – 1.43 (m, 5H), 1.38 – 1.31 (m, 2H), 1.26 – 1.09 (m, 10H), 1.06 (s, 3H), 1.02 – 0.97 (m, 3H), 0.94 – 0.89 (m, 3H), 0.87 (d, *J* = 6.8 Hz, 6H), 0.79 (t, *J* = 7.6 Hz, 3H), 0.69 (s, 3H). **^{13}C NMR** (100 MHz, $CDCl_3$) δ 166.2, 158.4, 148.5, 141.6, 139.9, 137.0, 134.5, 129.7, 129.4, 129.3, 125.8, 122.9, 113.8, 74.6, 73.9, 56.8, 56.3, 55.4, 50.2, 42.5, 39.9, 39.7, 38.4, 37.2, 36.8, 36.7, 36.3, 36.0, 32.1, 32.0, 30.0, 29.9, 28.4, 28.2, 28.0, 24.5, 24.4, 24.0, 23.0, 22.7, 21.2, 19.5, 18.9, 15.0, 14.3, 12.0. **IR**: v (cm^{-1}) 3503, 3032, 2955, 2870, 1717, 1699, 1609, 1576, 1508, 1466, 1410, 1375, 1319, 1275, 1246, 1175, 1117, 1107, 1036, 1018, 997, 978, 947, 926, 864, 833, 800. **HRMS** (ESI-TOF): m/z Calcd for $C_{50}H_{71}O_3$[M-OH]$^+$ 719.5398, found 719.5391.

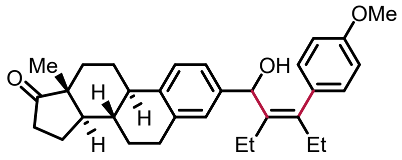

63: (8*S*,9*R*,13*R*,14*R*)-3-((*Z*)-2-ethyl-1-hydroxy-3-(4-methoxyphenyl)pent-2-en-1-yl)-13-methyl-6,7,8,9,11,12,13,14,15,16-decahydro-17H-cyclopenta[a]phenanthren-17-one was prepared according to the general procedure A. White solid (melting point 79–81 °C) after flash column chromatography (petroleum ether /EtOAc = 25:1), 70.9 mg, 75%. **^{1}H NMR** (400 MHz, $CDCl_3$) δ 7.23 (d, *J* = 7.2 Hz, 1H), 7.16 – 7.11 (m, 2H), 7.10 – 7.03 (m, 2H), 6.92 – 6.88 (m, 2H), 5.35 (s, 1H), 3.83 (s, 3H), 2.94 – 2.90 (m, 2H), 2.56 – 2.40 (m, 4H), 2.36 – 2.22 (m, 2H), 2.21 – 1.94 (m, 6H), 1.70 – 1.64 (m, 1H), 1.57 – 1.40 (m, 5H), 0.98 – 0.92 (m, 9H). **^{13}C NMR** (100 MHz, $CDCl_3$) δ 221.2, 158.3, 141.9, 141.9, 140.7, 140.7, 138.2, 138.2, 137.9, 137.9, 136.2, 136.2, 134.5, 130.0,

126.5, 126.3, 125.0, 123.5, 123.4, 113.7, 74.0, 74.0, 55.4, 50.7, 48.2, 44.5, 44.5, 38.3, 36.0, 31.8, 29.7, 27.6, 26.7, 25.9, 21.7, 20.4, 16.0, 16.0, 14.0, 12.9. **IR**: ν (cm^{-1}) 3491, 3103, 3055, 2959, 2932, 2872, 1709, 1605, 1584, 1562, 1552, 1520, 1504, 1468, 1433, 1406, 1373, 1358, 1337, 1283, 1269, 1244, 1231, 1169, 1153, 1113, 1101, 1013, 957, 872, 858, 818, 770. **HRMS** (ESI-TOF): m/z Calcd for $C_{32}H_{39}O_2[M-OH]^+$ 455.2945, found 455.2943.

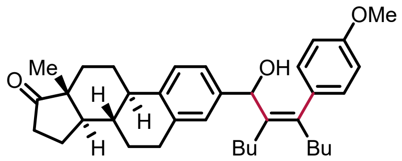

64: (8S,9R,13R,14R)-3-((Z)-2-butyl-1-hydroxy-3-(4-methoxyphenyl)hept-2-en-1-yl)-13-methyl-6,7,8,9,11,12,13,14,15,16-decahydro-17H-cyclopenta[a]phenanthren-17-one was prepared according to the general procedure A. White solid (melting point 78–80 °C) after flash column chromatography (petroleum ether /EtOAc = 25:1), 74 mg, 70%. **^{1}H NMR** (400 MHz, $CDCl_3$) δ 7.20 (d, J = 8.4 Hz, 1H), 7.09 (d, J = 8.8 Hz, 2H), 7.07 – 6.99 (m, 2H), 6.86 (d, J = 8.0 Hz, 2H), 5.32 (s, 1H), 3.80 (s, 3H), 2.89 (s, 2H), 2.54 – 2.47 (m, 1H), 2.42 – 2.26 (m, 5H), 2.15 – 1.93 (m, 5H), 1.68 – 1.59 (m, 2H), 1.55 – 1.41 (m, 5H), 1.30 –1.20 (m, 6H), 1.09 – 1.05 (m, 2H), 0.91 (s, 3H), 0.84 (dd, J = 6.4 Hz, 6H). **^{13}C NMR** (100 MHz, $CDCl_3$) δ 221.2, 158.2, 140.8, 140.7, 140.7, 138.2, 138.2, 137.1, 136.2, 136.2, 134.8, 129.8, 126.5, 126.4, 125.0, 123.5, 123.4, 113.7, 74.0, 55.4, 50.7, 48.2, 44.5, 38.4, 36.0, 34.5, 33.6, 31.8, 30.4, 29.7, 27.6, 26.7, 25.9, 23.6, 23.0, 21.7, 14.17, 14.04, 14.0, 14.0. **IR**: ν (cm^{-1}) 3482, 2957, 2928, 2855, 1742, 1607, 1508, 1458, 1375, 1244, 1103, 1082, 1038, 1009, 968, 908, 833, 772. **HRMS** (ESI-TOF): m/z Calcd for $C_{34}H_{43}O_2[M-OH]^+$ 483.3258, found 483.3256.

3.2 General procedure B

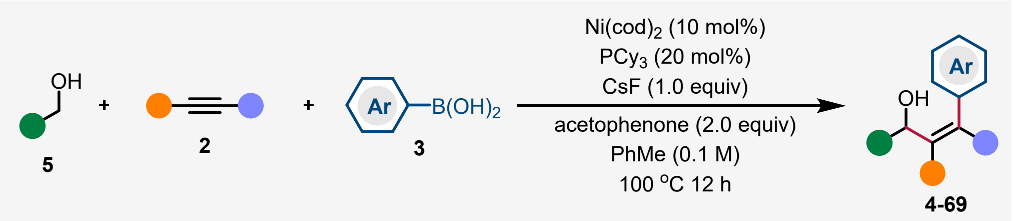

In a glovebox, an oven dried screw-capped 8 mL vial was charged with a magnetic stir bar, $Ni(cod)_2$ (5.5 mg, 0.02 mmol, 10 mol%), PCy_3 (11.2 mg, 0.04 mmol, 20 mol%), CsF (30.4 mg, 0.2 mmol, 1.0 equiv.) and aryl boronic acids (0.4 mmol, 2.0 equiv.) were added successively. Then degassed toluene (2.0 mL) was added and the catalyst mixture was stirred at rt for 5 min. Alcohols (0.2 mmol), alkynes (0.4 mmol) and acetophenone (46.6

μL, 0.4 mmol, 2.0 equiv.) were then added. The vial was sealed with a teflon-lined screw cap, shipped outside of the glovebox, and added to a pre-heated aluminum heating mental at 100 °C. After stirring for 12 h, the vial was removed and allowed to cool to rt. The reaction mixture was diluted with ethyl acetate and filtered through a short plug of silica gel. The crude solution was concentrated *in vacuo* and subjected to column chromatography to provide pure product.

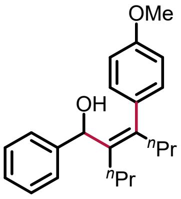

4: (*Z*)-3-(4-methoxyphenyl)-1-phenyl-2-propylhex-2-en-1-ol was prepared according to the general procedure B. 42% yield (determined by ^{1}H NMR using 1,1,2,2-tetrachloroethane as an internal standard).

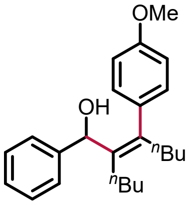

32: (*Z*)-2-butyl-3-(4-methoxyphenyl)-1-phenylhept-2-en-1-ol was prepared according to the general procedure B. 64% yield (determined by ^{1}H NMR using 1,1,2,2-tetrachloroethane as an internal standard).

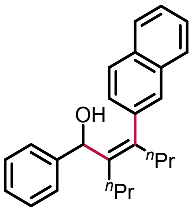

54: (*Z*)-3-(naphthalen-2-yl)-1-phenyl-2-propylhex-2-en-1-ol was prepared according to the general procedure B. 32% yield (determined by ^{1}H NMR using 1,1,2,2-tetrachloroethane as an internal standard).

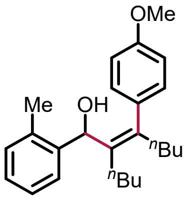

65: (*Z*)-2-butyl-3-(4-methoxyphenyl)-1-(*o*-tolyl)hept-2-en-1-ol was prepared according to the general procedure B. Colorless oil after flash column chromatography (petroleum ether /EtOAc = 25:1), 38.8 mg, 53%. **^{1}H NMR** (400 MHz, $CDCl_3$) δ 7.63 (d, *J* = 7.6 Hz, 1H), 7.22 – 7.14 (m, 4H), 7.11 (d, *J* = 7.2 Hz, 1H), 7.00 (d, *J* = 7.6 Hz, 1H), 6.89 (d, *J* = 8.0 Hz, 2H), 5.24 (s, 1H), 3.82 (s, 3H), 2.49 – 2.42 (m, 1H), 2.28 – 2.21 (m, 1H), 2.09 – 2.02 (m, 1H), 1.97 – 1.89 (m, 1H), 1.62 (d, *J* = 7.6 Hz, 2H), 1.33 – 1.15 (m, 6H), 1.15 – 1.01 (m, 2H), 0.84 (t, *J* = 7.2 Hz, 3H), 0.70 (t, J = 7.4 Hz, 3H), 0.54 –0.45 (m, 1H). **^{13}C NMR** (100 MHz, $CDCl_3$) δ 158.4, 141.9, 141.2, 135.5, 135.1, 134.6, 129.8, 129.8, 126.8, 125.7, 125.6, 113.5, 71.9, 55.4, 34.3, 32.3, 30.5, 27.7, 23.3, 22.9, 19.6, 14.2, 13.8. **IR**: ν (cm^{-1}) 3329, 2957, 2934, 2859, 2833, 1609, 1574, 1508, 1464, 1379, 1290, 1244, 1179, 1107, 1003, 833, 810. **HRMS** (ESI-TOF): m/z Calcd for $C_{25}H_{33}O$ $[M-OH]^+$ 349.2526, found 349.2523.

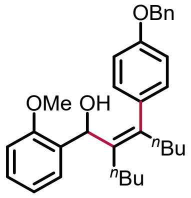

66: (*Z*)-3-(4-(benzyloxy)phenyl)-2-butyl-1-(2-methoxyphenyl)hept-2-en-1-ol was prepared according to the general procedure B. Colorless oil after flash column chromatography (petroleum ether /EtOAc = 30:1), 49.6 mg, 54%. **^{1}H NMR** (400 MHz, $CDCl_3$) δ 7.46 – 7.31 (m, 6H), 7.21 (t, *J* = 7.6 Hz, 1H), 7.09 – 7.05 (m, 2H), 6.96 – 6.91 (m, 3H), 6.79 (d, *J* = 8.4 Hz, 1H), 5.46 (s, 1H), 5.05 (s, 2H), 3.71 (s, 3H), 2.49 (s, 1H), 2.36 (t, *J* = 7.4 Hz, 2H), 2.20 (dt, *J* = 20.4, 4.8 Hz, 1H), 2.05 (dt, *J* = 20.0, 5.2 Hz, 1H), 1.49 – 1.40 (m, 1H), 1.33 – 1.15 (m, 6H), 1.00 – 0.90 (m, 1H), 0.84 (m, 6H). **^{13}C NMR** (100 MHz, $CDCl_3$) δ 157.4, 157.1, 140.5, 137.3, 135.7, 135.4, 131.5, 129.8, 128.7, 128.1, 128.0, 127.7, 127.5, 120.5, 114.1, 110.3, 71.2, 70.1, 55.2, 34.4, 33.0, 30.4, 28.1, 23.5, 22.9, 14.2, 14.0. **IR**: ν (cm^{-1}) 3510, 2999, 2931, 2859, 2160, 1732, 1605, 1587, 1508, 1498, 1464, 1437, 1379, 1288, 833, 754. **HRMS** (ESI-TOF): m/z Calcd for $C_{31}H_{37}O_2$ $[M-OH]^+$ 441.2788, found 441.2784.

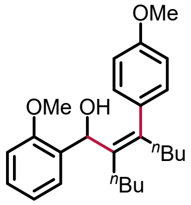

67: (*Z*)-2-butyl-1-(2-methoxyphenyl)-3-(4-methoxyphenyl)hept-2-en-1-ol was prepared according to the general procedure B. Colorless oil after flash column chromatography (petroleum ether /EtOAc = 25:1), 38.3 mg, 50%. **^{1}H NMR** (400 MHz, $CDCl_3$) δ 7.36 (d, *J* = 7.6 Hz, 1H), 7.20 (t, *J* = 8.0 Hz, 1H), 7.08 – 7.04 (m, 2H), 6.94 (t, *J* = 7.6 Hz, 1H), 6.84 – 6.82 (m, 2H), 6.77 (d, *J* = 8.4, 1H), 5.44 (s, 1H), 3.80 (s, 3H), 3.72 (s, 3H), 2.51 (s, 1H), 2.35

(t, *J* = 8.2 Hz, 2H), 2.22 – 2.14 (m, 1H), 2.07 – 2.00 (m, 1H), 1.48 – 1.83 (m, 1H), 1.32 – 1.14 (m, 6H), 0.99 – 0.91 (m, 1H), 0.87 – 0.78 (m, 6H). **^{13}C NMR** (100 MHz, $CDCl_3$) δ 158.1, 157.1, 140.5, 135.7, 135.2, 131.5, 129.8, 128.0, 127.4, 120.5, 113.2, 110.3, 71.1, 55.3, 55.2, 34.4, 33.0, 30.4, 28.1, 23.5, 22.9, 14.2, 14.0. **IR**: ν (cm^{-1}) 3318, 2957, 2932, 2859, 2835, 1607, 1587, 1510, 1491, 1460, 1375, 1285, 1240, 1177, 1107, 1036, 1003, 833, 754. **HRMS** (ESI-TOF): m/z Calcd for $C_{25}H_{33}O_2$ $[M-OH]^+$ 365.2473, found 365.2478.

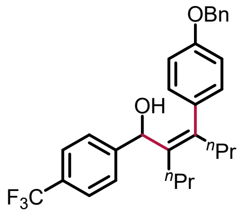

68: (*Z*)-3-(4-(benzyloxy)phenyl)-2-propyl-1-(4-(trifluoromethyl)phenyl)hex-2-en-1-ol was prepared according to the general procedure B. Colorless oil after flash column chromatography (petroleum ether /EtOAc = 25:1), 43.2 mg, 46%. **^{1}H NMR** (400 MHz, $CDCl_3$) δ 7.54 (d, *J* = 8.0 Hz, 2H), 7.45 – 7.31 (m, 7H), 7.10 (d, *J* = 8.4 Hz, 2H), 6.96 (d, *J* = 8.0 Hz, 2H), 5.42 (s, 1H), 5.05 (s, 2H), 2.36 – 2.31 (m, 2H), 2.13 – 2.06 (m, 1H), 1.91 – 1.83 (m, 1H), 1.64 (s, 1H), 1.42 – 1.37 (m, 1H), 1.33 – 1.25 (m, 2H), 1.03 – 0.98 (m, 1H), 0.89 (t, *J* = 7.2 Hz, 3H), 0.81 (t, *J* = 7.2 Hz, 3H). **^{13}C NMR** (100 MHz, $CDCl_3$) δ 157.7, 147.3, 141.8, 137.1, 136.9, 134.7, 129.7, 129.0(*J* = 32.2 Hz), 128.8, 128.2, 127.7, 126.2, 125.0(*J* = 3.7 Hz), 124.4(*J* = 270.3 Hz), 114.8, 73.7, 70.2, 36.7, 30.0, 24.5, 21.2, 15.0, 14.3. **IR**: ν (cm^{-1}) 3462, 3036, 2872, 1607, 1508, 1456, 1412, 1379, 1327, 1240, 1165, 1069, 1018, 858, 833, 770. **HRMS** (ESI-TOF): m/z Calcd for $C_{29}H_{30}F_3O$ $[M-OH]^+$ 451.2243, found 451.2246.

4. X-Ray crystal structure of 7, 9, 10 and 11.

X-ray crystal structure of **7** (CCDC 2209527)

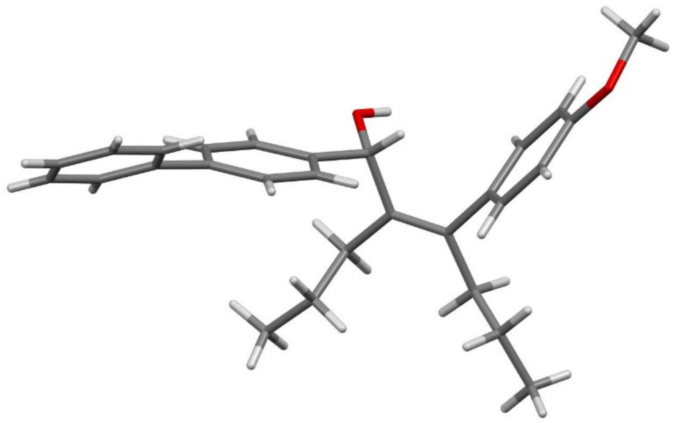

Bond precision: C-C = 0.0058 A Wavelength=1.54184

Cell: a=17.9673(7) b=15.1856(5) c=18.5806(8)

alpha=90 beta=103.304(4) gamma=90

Temperature: 293 K

	Calculated	Reported
Volume	4933.6(3)	4933.6(3)
Space group	P 21/n	P 1 21/n 1
Hall group	-P 2yn	-P 2yn
Moiety formula	C28 H32 O2	2(C28 H32 O2)
Sum formula	C28 H32 O2	C56 H64 O4
Mr	400.54	801.07
Dx,g cm-3	1.079	1.078
Z	8	4
Mu (mm-1)	0.510	0.510
F000	1728.0	1728.0
F000’	1732.64	
h,k,lmax	20, 17, 21	20, 17, 21
Nref	8121	8104
Tmin,Tmax	0.970, 0.980	0.019, 1.000

Tmin’ 0.970

Correction method= # Reported T Limits: Tmin=0.019 Tmax=1.000

AbsCorr = MULTI-SCAN

Data completeness= 0.998 Theta(max)= 63.687

R(reflections)= 0.0955(4800)

wR2(reflections)= 0.3144(8104)

S = 1.081 Npar= 549

X-ray crystal structure of **9** (CCDC 2209525)

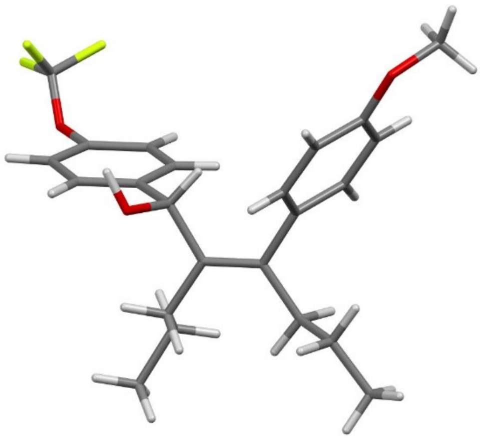

Bond precision: C-C = 0.0060 A Wavelength=1.54184

Cell: a=12.5957(3) b=13.0422(4) c=15.1090(5)

alpha=84.924(3) beta=76.057(2) gamma=69.976(2)

Temperature: 293 K

	Calculated	Reported
Volume	2263.24(12)	2263.24(12)
Space group	P -1	P -1
Hall group	-P 1	-P 1
Moiety formula	C23 H27 F3 O3	2(C23 H27 F3 O3)
Sum formula	C23 H27 F3 O3	C46 H54 F6 O6
Mr	408.45	816.89
Dx,g cm-3	1.199	1.199
Z	4	2
Mu (mm-1)	0.794	0.794
F000	864.0	864.0
F000’	867.04	
h,k,lmax	14, 15, 17	14, 15, 17
Nref	7994	7780
Tmin,Tmax	0.953, 0.969	0.564, 1.000
Tmin’	0.953	

Correction method= # Reported T Limits: Tmin=0.564 Tmax=1.000

AbsCorr = MULTI-SCAN

Data completeness= 0.973 Theta(max)= 66.581

R(reflections)= 0.0881(4776)

wR2(reflections)= 0.3349(7780)

S = 1.220 Npar= 534

X-ray crystal structure of **10** (CCDC 2209526)

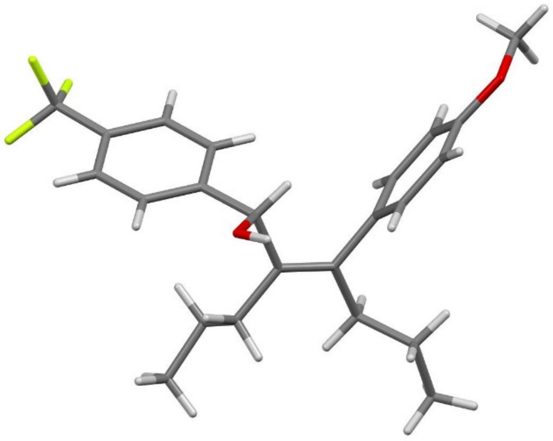

Bond precision: C-C = 0.0056 A Wavelength=1.54184

Cell: a=12.3606(7) b=13.3752(10) c=14.6048(8)

alpha=84.368(5) beta=77.529(5) gamma=69.589(6)

Temperature: 293 K

	Calculated	Reported
Volume	2208.9(3)	2208.9(3)
Space group	P -1	P -1
Hall group	-P 1	-P 1
Moiety formula	C23 H27 F3 O2	2(C23 H27 F3 O2)
Sum formula	C23 H27 F3 O2	C46 H54 F6 O4
Mr	392.45	784.89
Dx,g cm-3	1.180	1.180
Z	4	2
Mu (mm-1)	0.758	0.758
F000	832.0	832.0
F000’	834.84	
h,k,lmax	14, 15, 17	14, 15, 17
Nref	7810	7576
Tmin,Tmax	0.948, 0.963	0.696, 1.000
Tmin’	0.948	

Correction method= # Reported T Limits: Tmin=0.696 Tmax=1.000

AbsCorr = MULTI-SCAN

Data completeness= 0.970 Theta(max)= 66.591

R(reflections)= 0.0965(4900)

wR2(reflections)=0.3433(7576)

S = 1.222 Npar= 513

X-ray crystal structure of **11** (CCDC 2209215)

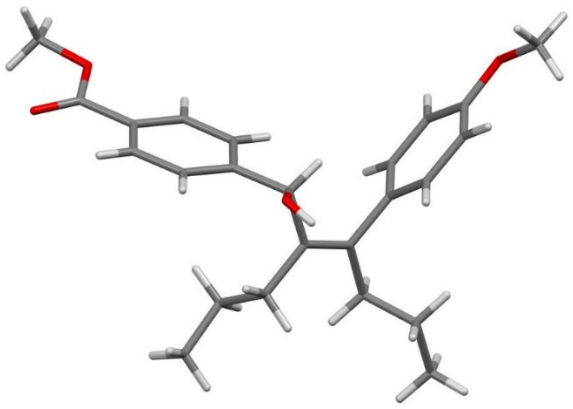

Bond precision: C-C = 0.0046 A Wavelength=1.54184

Cell: a=15.9821(4) b=14.8327(3) c=19.0411(4)

alpha=90 beta=94.264(2) gamma=90

Temperature: 293 K

	Calculated	Reported
Volume	4501.35(17)	4501.34(17)
Space group	P 21/n	P 1 21/n 1
Hall group	-P 2yn	-P 2yn
Moiety formula	C24 H30 O4	2(C24 H30 O4)
Sum formula	C24 H30 O4	C48 H60 O8
Mr	382.48	764.96
Dx,g cm-3	1.129	1.129
Z	8	4
Mu (mm-1)	0.603	0.603
F000	1648.0	1648.0
F000’	1652.87	
h,k,lmax	19,18, 23	19, 17, 22
Nref	8451	8230
Tmin,Tmax	0.964, 0.976	0.152,1.000
Tmin’	0.947	

Correction method= # Reported T Limits: Tmin=0.152 Tmax=1.000

AbsCorr = MULTI-SCAN

Data completeness= 0.974 Theta(max)= 69.500

R(reflections)= 0.0946(6313)

wR2(reflections)=0.2958(8230)

S = 1.139 Npar= 513

5. Scope limitations and miscellaneous experiments

5.1. Scope limitations

During the evaluation of the scope, several reactions that gave low or no yield of products. Aldehydes bound to certain Lewis basic heterocycles such as pyridine and indole were found to be unreactive or low reactive. No desired three-component product was obtained with bromo substituted benzaldehyde. The aliphatic aldehyde with α-hydrogen is proven to be challenging, presumably due to the completing aldehyde oligomerization. Pyridinyl, quinoline and alkylboronic acids were similarly inert. Lastly, alkynoates and terminal alkynes are not well tolerated. A selection of these low yielding substrate scope is shown below.

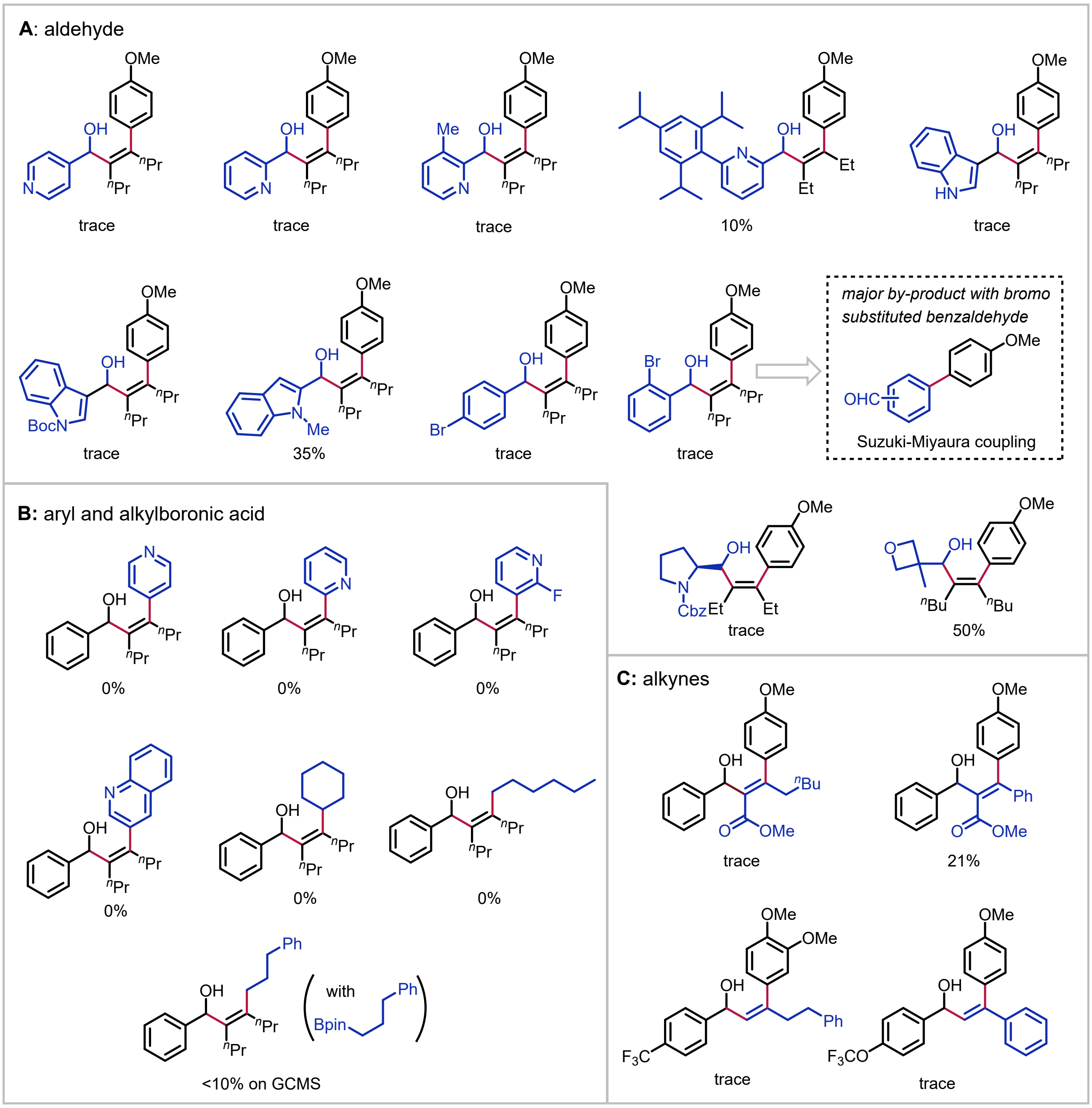

Figure S1. Low yielding reactions.

5.2. Miscellaneous experiments

A. The experiments on the ratios of toluene/MeOH

Table S7 Evaluation of the ratios of Toluene/MeOH.[a]

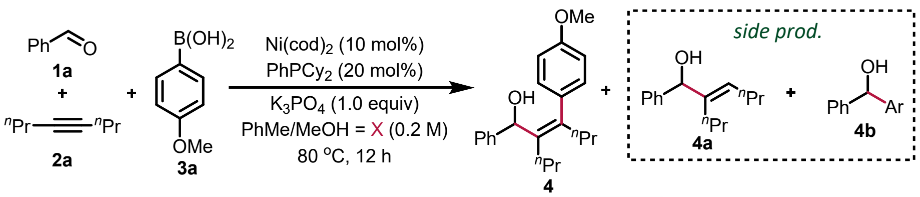

Entry	X	Yield (%) 4	4a	4b	RSM 1a
1	9:1	82	8	5	0
2	3:1	74	8	9	0
3	1:1	26	12	14	23
4	1:3	17	11	17	31

methyl benzoate and intractable mixtures were detected on GCMS

[a]Yieds were determined by ^{1}H NMR

Comments: We check the ratio of co-solvent, these data are summarized. 1, Increase the ratio of methanol, the yield of **4** is gradually decreased, especially in Entry 3 and 4, fairly amount of benzaldehyde was left over. 2, The 1,2-addition by-product **4b** increased gradually with the increase of methanol, which may be attribute to the faster transmetalation step facilitated by alcohol solvent. 3, In entry 3 and 4, we observed fairly amount of methyl benzoate (generated from nickel catalyzed oxidative esterification,10.1002/anie.201410322) on the GCMS, as well as intractable mixtures.

B. Run the model reaction at 1 mmol scale.

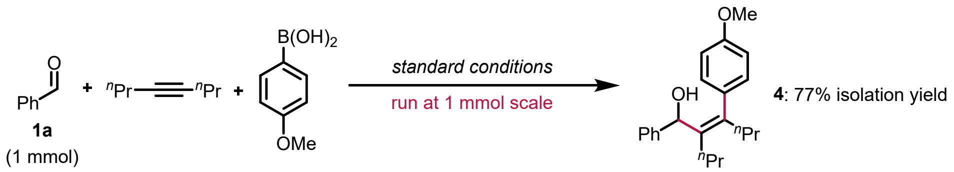

Comments: We tried the model reaction at 1 mmol scale, it works smoothly, the isolation yield is 77%.

C. Run the model reaction outside the glovebox with air stable Ni(cod)(DQ).

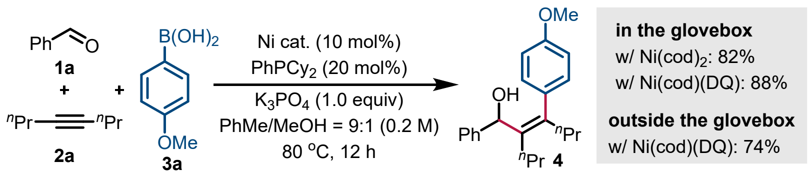

Comments: This reaction procced with comparable efficiency outside the glovebox, 74% yield was obtained when this reaction was setup in the Schlenk tube.

D. The test of the Ph_3B in this reaction.

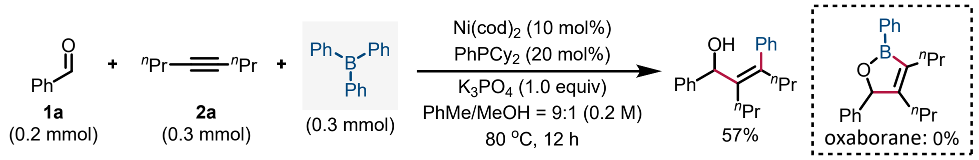

Comments: Three component arylative coupling product was obtained in 57% yield, no oxaborane product was observed either on GCMS or ^{1}H NMR.

E. Additional experiments on the loading of arylboronic acid

Table S8 Evaluation of the loading of arylboronic acid.[a]

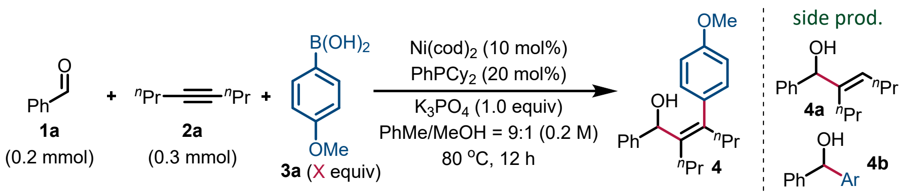

Entry	X	Yield (%) 4	4a	4b
1	2.0	82	8	5
2	1.2	70	9	11
3	1.5	84	8	8

Yieds were determined by ^{1}H NMR

slightly higher compared with entry 1

F. The explanation on the generation of Ni(II)-H intermediate in Scheme 4B

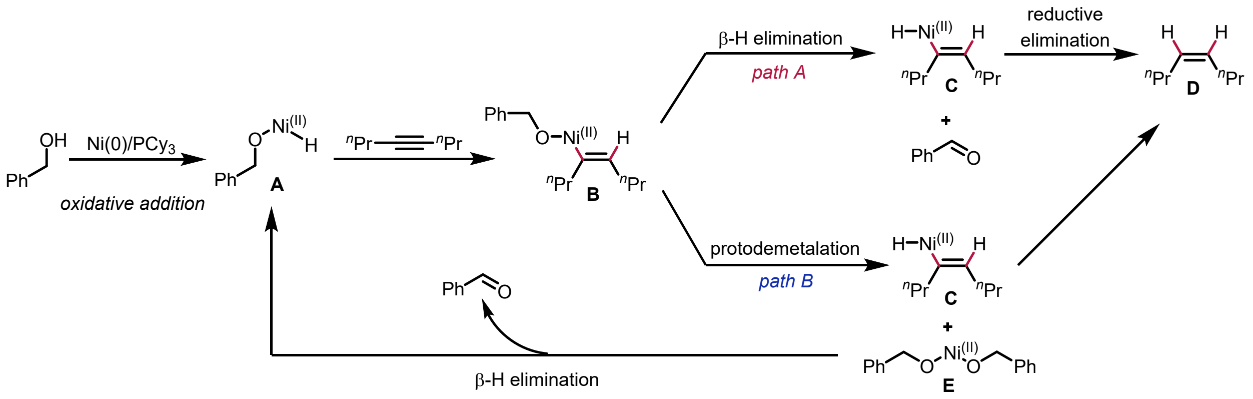

Comments: Given that the oxidative addition of Ni(0) complex into a O−H bond has been reported (10.1002/anie.201710735, 10.1038/s41467-019-12949-1), we proposed that the oxidation of the alcohol (namely the generation of Ni-H species) may proceed in an step-wise manner based on the literature precedents. Firstly, the intermediate **A** undergoes insertion into alkyne to afford the **B**. Then, two pathways can be raised. β-Hydride elimination followed by reductive elimination could produce the corresponding benzaldehyde (Path A). The intermediate **B** undergoes protodemetalation with benzyl alcohol could afford the **C** and Ni-alkoxide species **E**, which also could generate benzaldehyde upon β-hydride elimination.

G. The ee value with (*S*)-NMDPP ligand described in the optimization Table 1 (entry 11)

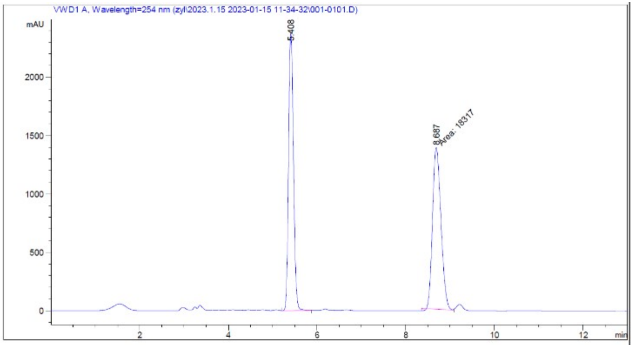

Area Percent Report

Sorted By : Signal
Multiplier : 1.0000
Dilution : 1.0000
Use Multiplier & Dilution Factor with ISTDs

Signal 1: VWD1 A, Wavelength=254 nm

Peak #	RetTime [min]	Type	Width [min]	Area [mAU*s]	Height [mAU]	Area %
1	5.408	BB	0.1206	1.82354e4	2375.69531	49.8884
2	8.687	MM	0.2206	1.83170e4	1383.88586	50.1116
Totals :				3.65524e4	3759.58118	

racemic sample

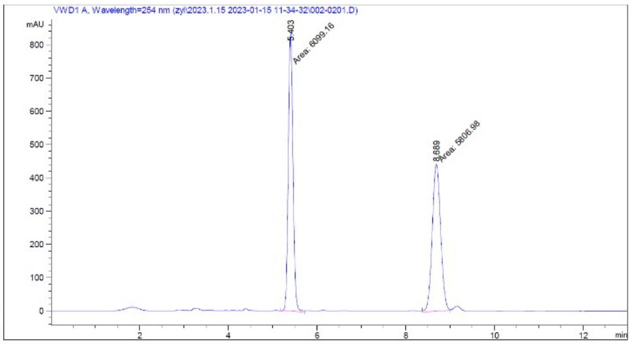

Area Percent Report

Sorted By : Signal
Multiplier : 1.0000
Dilution : 1.0000
Use Multiplier & Dilution Factor with ISTDs

Signal 1: VWD1 A, Wavelength=254 nm

Peak #	RetTime [min]	Type	Width [min]	Area [mAU*s]	Height [mAU]	Area %
1	5.403	MM	0.1219	6099.16113	834.10547	51.2270
2	8.689	MM	0.2190	5806.97998	441.92184	48.7730
Totals :				1.19061e4	1276.02731	

sample of entry 11 in the table 1

Comments: We tested the ee value with chiral HPLC (Chiralpark AD-H, 25 ^{o}C, flow rate: 1 mL/min, hexane/isopropanol: 90/10, 254 nm, t_1 = 5.408 min, t_2 = 8.687 min.), however, only 2.4% ee was observed, indicating that enantiocontrol of (*S*)-NMDPP is bad for this reaction.

6. NMR spectra

4: (*Z*)-3-(4-methoxyphenyl)-1-phenyl-2-propylhex-2-en-1-ol (^{1}H NMR, $CDCl_3$, 400 MHz)

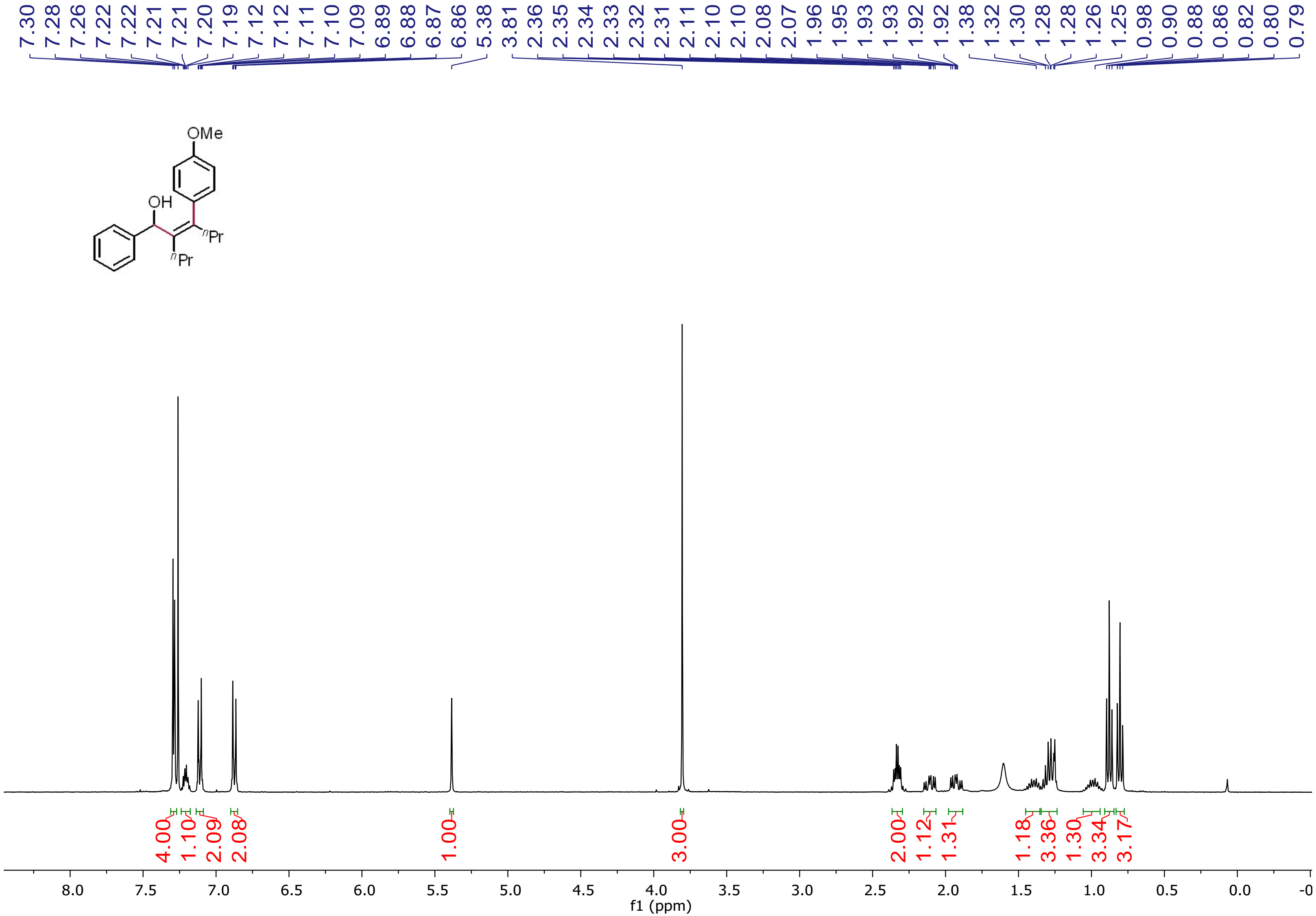

4: (*Z*)-3-(4-methoxyphenyl)-1-phenyl-2-propylhex-2-en-1-ol (^{13}C NMR, $CDCl_3$, 100 MHz)

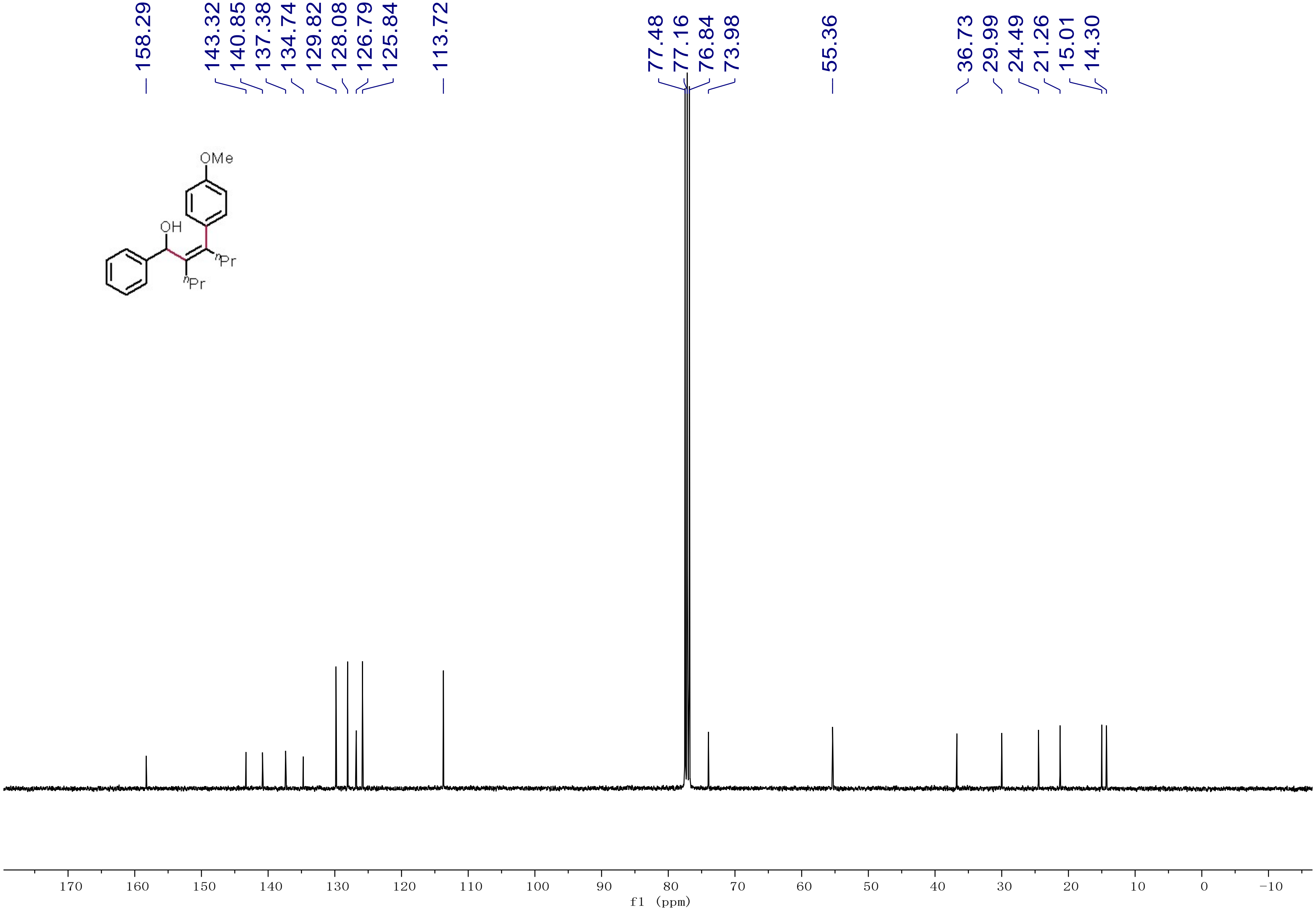

5: (*Z*)-3-(4-methoxyphenyl)-2-propyl-1-(p-tolyl)hex-2-en-1-ol (^{1}H NMR, $CDCl_3$, 400 MHz)

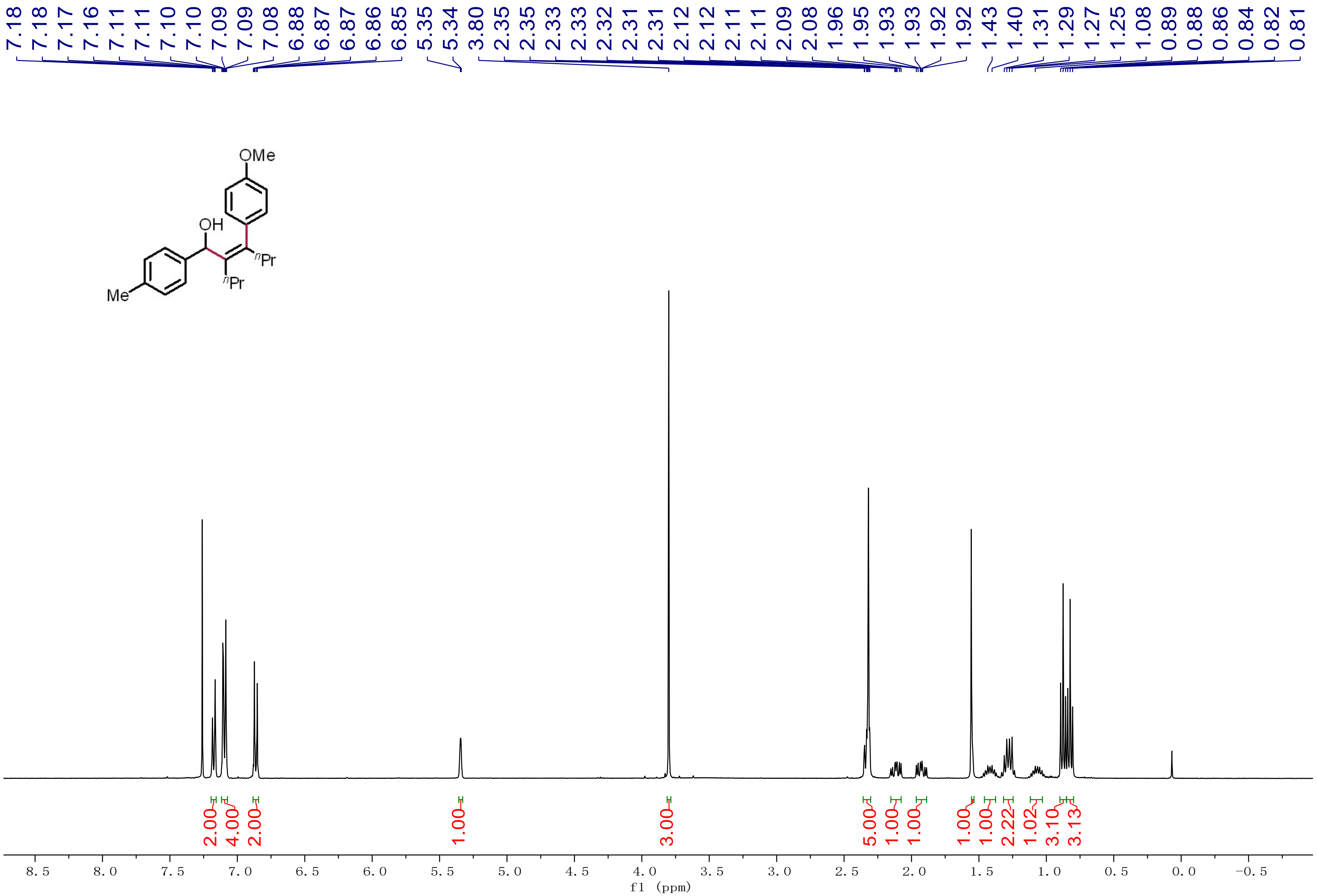

5: (*Z*)-3-(4-methoxyphenyl)-2-propyl-1-(p-tolyl)hex-2-en-1-ol (^{13}C NMR, $CDCl_3$, 100 MHz)

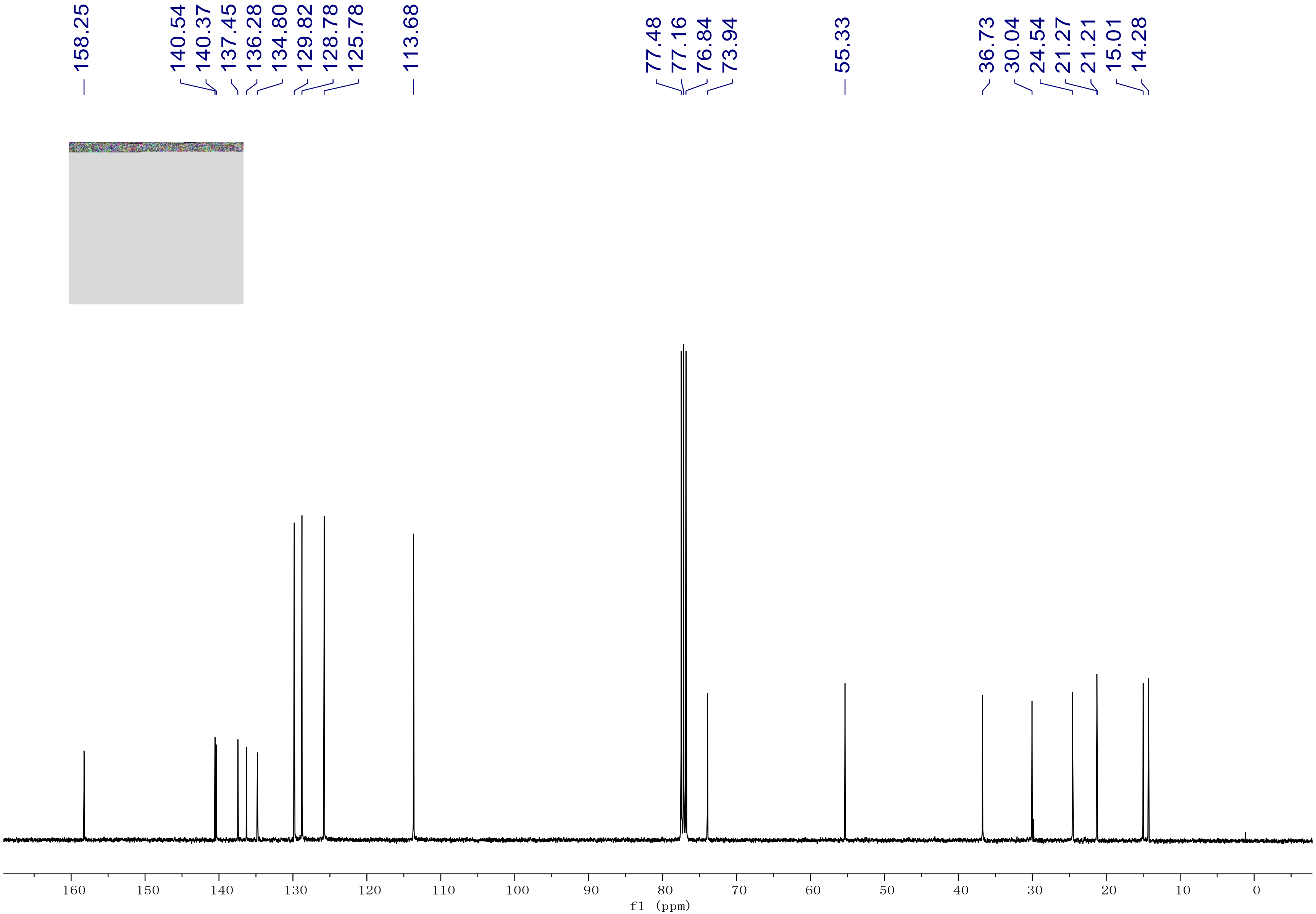

6: (*Z*)-1-(4-(tert-butyl)phenyl)-3-(4-methoxyphenyl)-2-propylhex-2-en-1-ol (^{1}H NMR, $CDCl_3$, 400 MHz)

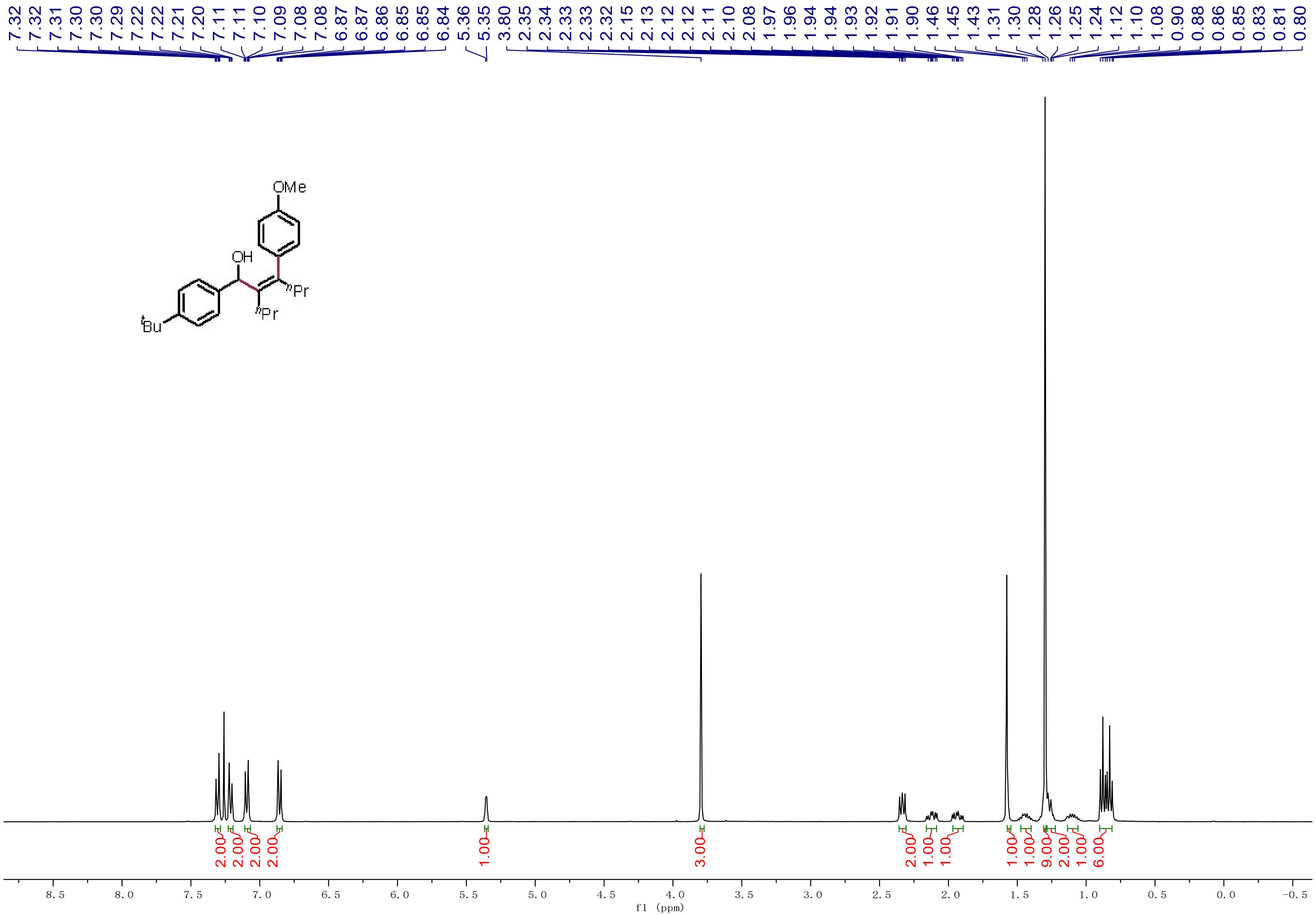

6: (*Z*)-1-(4-(tert-butyl)phenyl)-3-(4-methoxyphenyl)-2-propylhex-2-en-1-ol (^{13}C NMR, $CDCl_3$, 100 MHz)

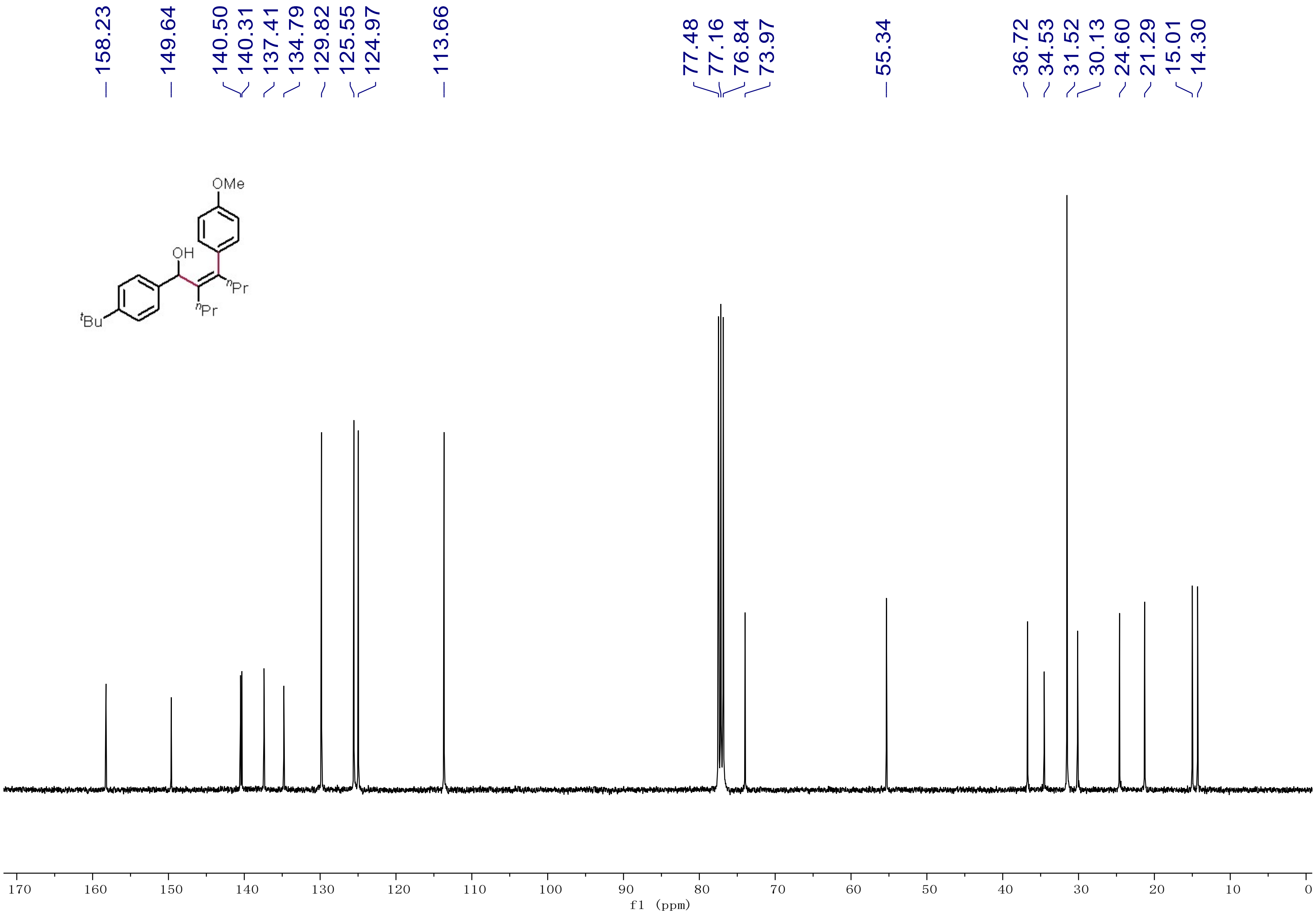

7: (*Z*)-1-([1,1'-biphenyl]-4-yl)-3-(4-methoxyphenyl)-2-propylhex-2-en-1-ol (^{1}H NMR, $CDCl_3$, 400 MHz)

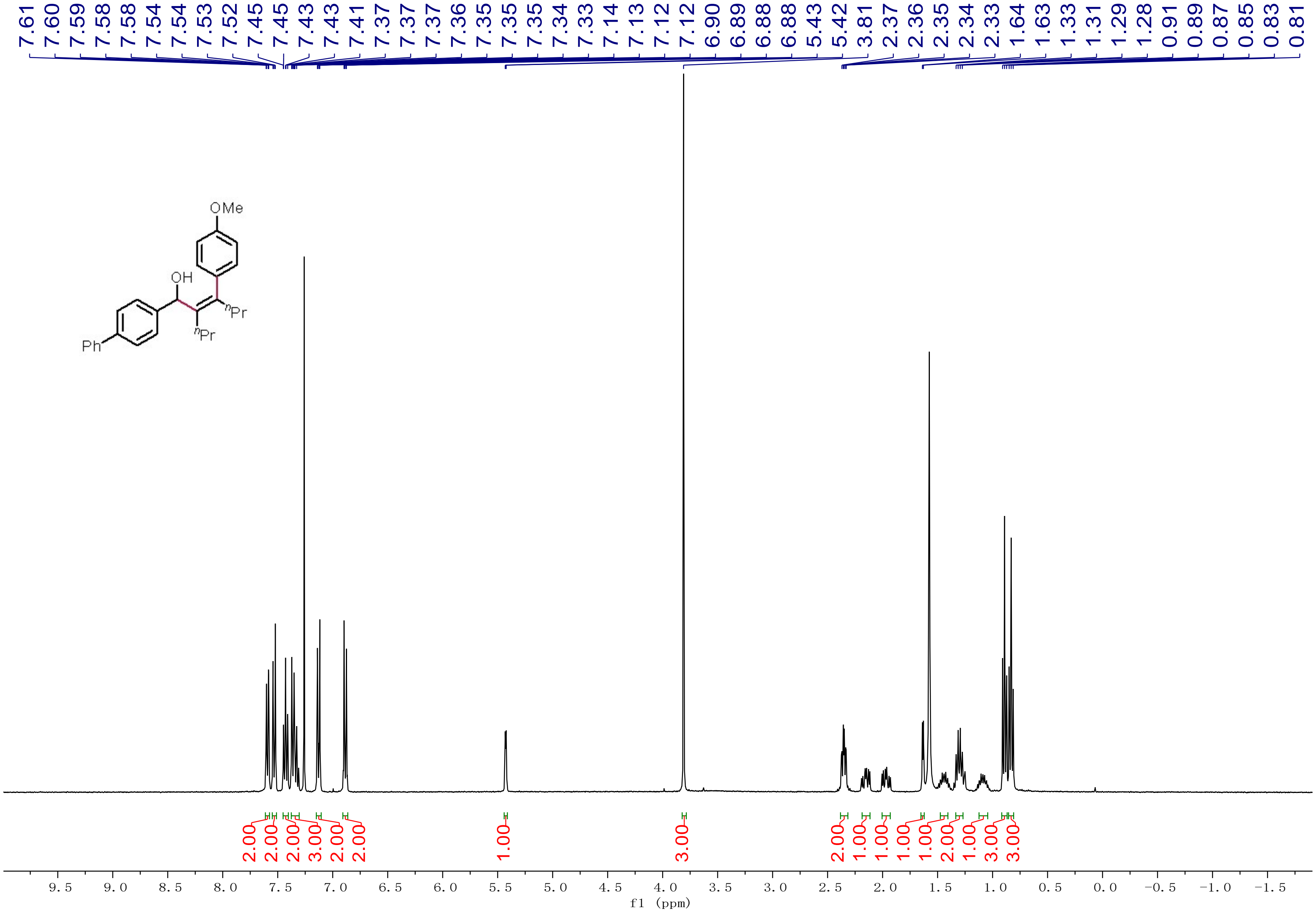

7: (*Z*)-1-([1,1'-biphenyl]-4-yl)-3-(4-methoxyphenyl)-2-propylhex-2-en-1-ol (^{13}C NMR, $CDCl_3$, 100 MHz)

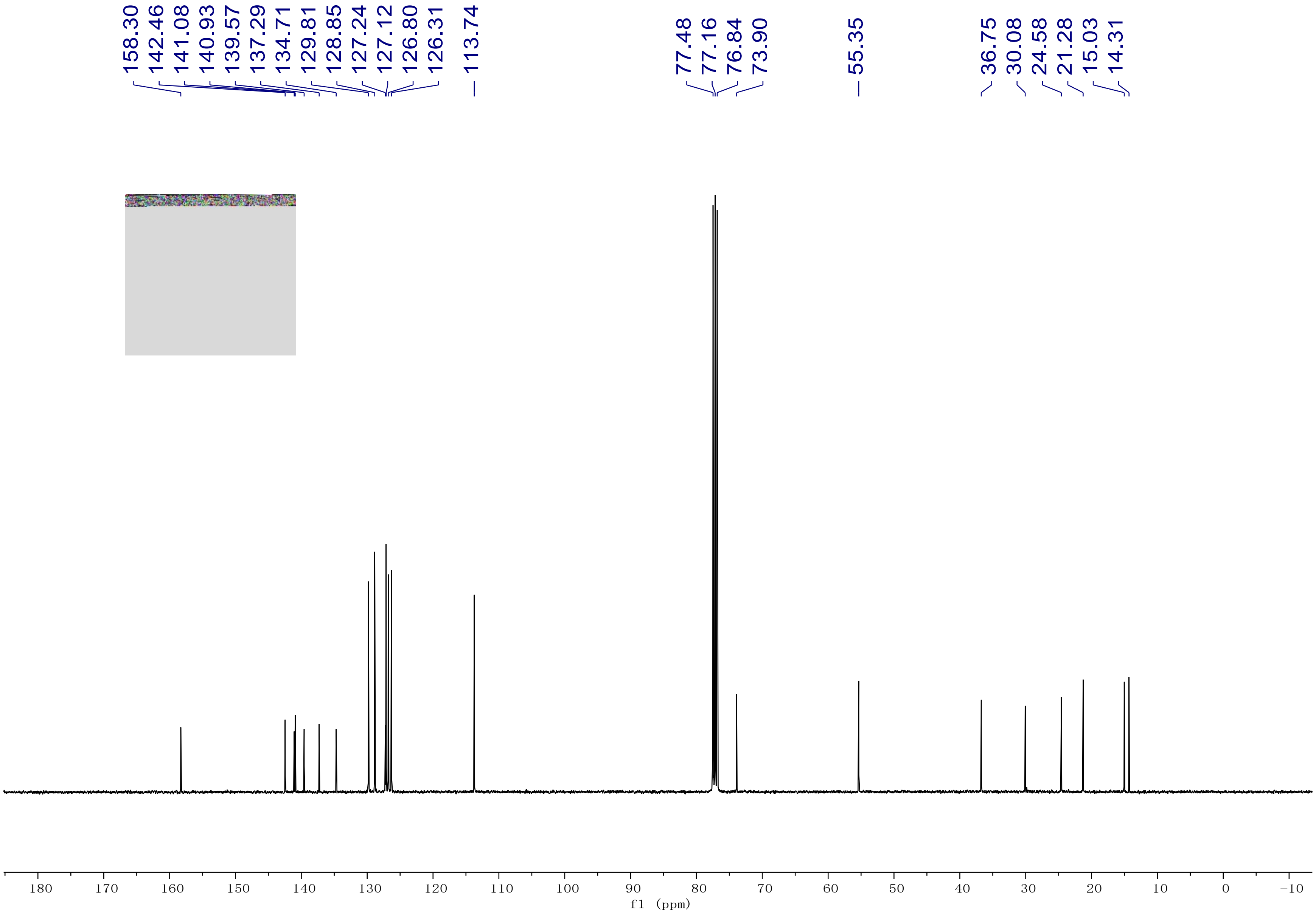

8: (*Z*)-1-(4-fluorophenyl)-3-(4-methoxyphenyl)-2-propylhex-2-en-1-ol (^{1}H NMR, $CDCl_3$, 400 MHz)

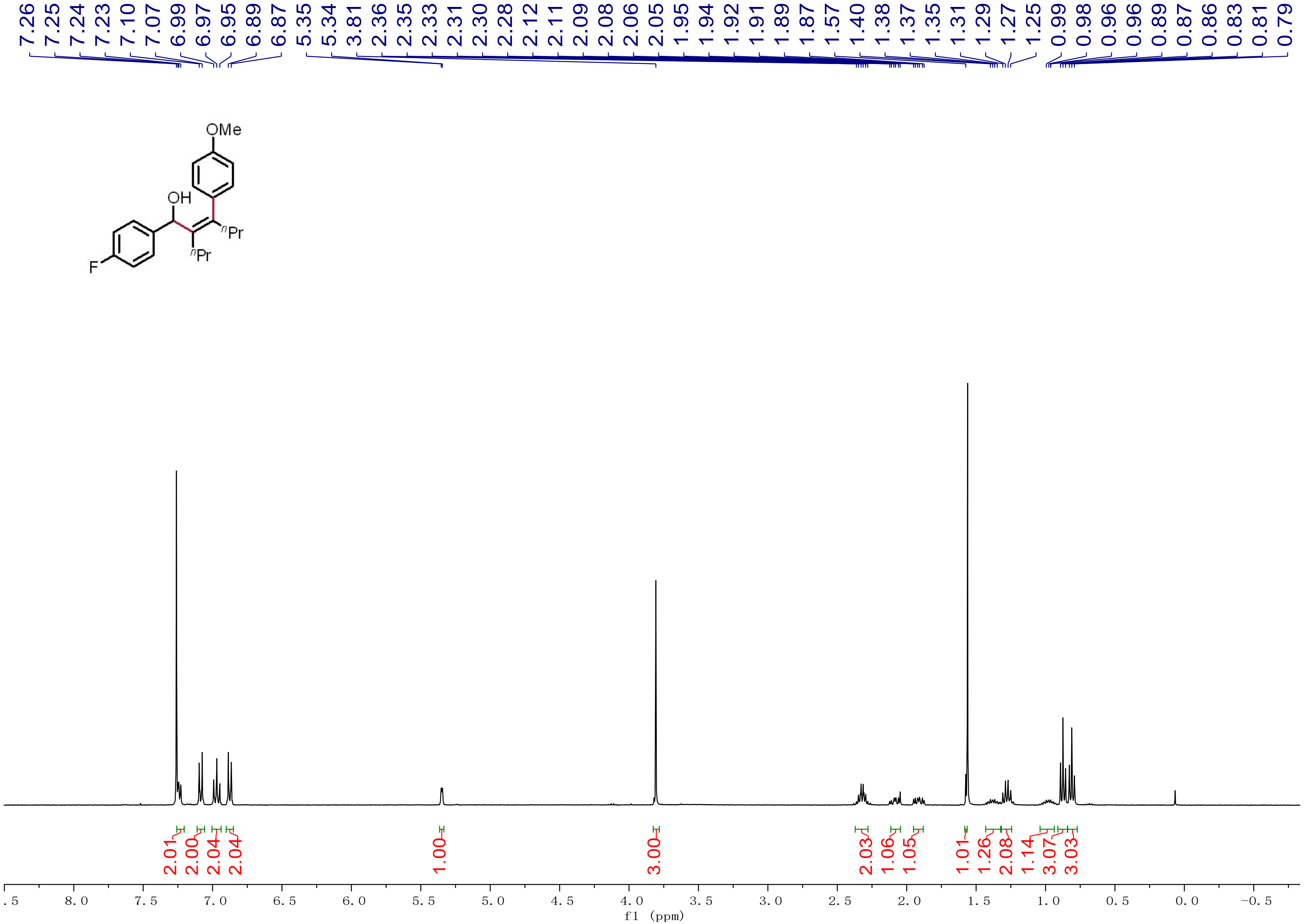

8: (*Z*)-1-(4-fluorophenyl)-3-(4-methoxyphenyl)-2-propylhex-2-en-1-ol (^{13}C NMR, $CDCl_3$, 100 MHz)

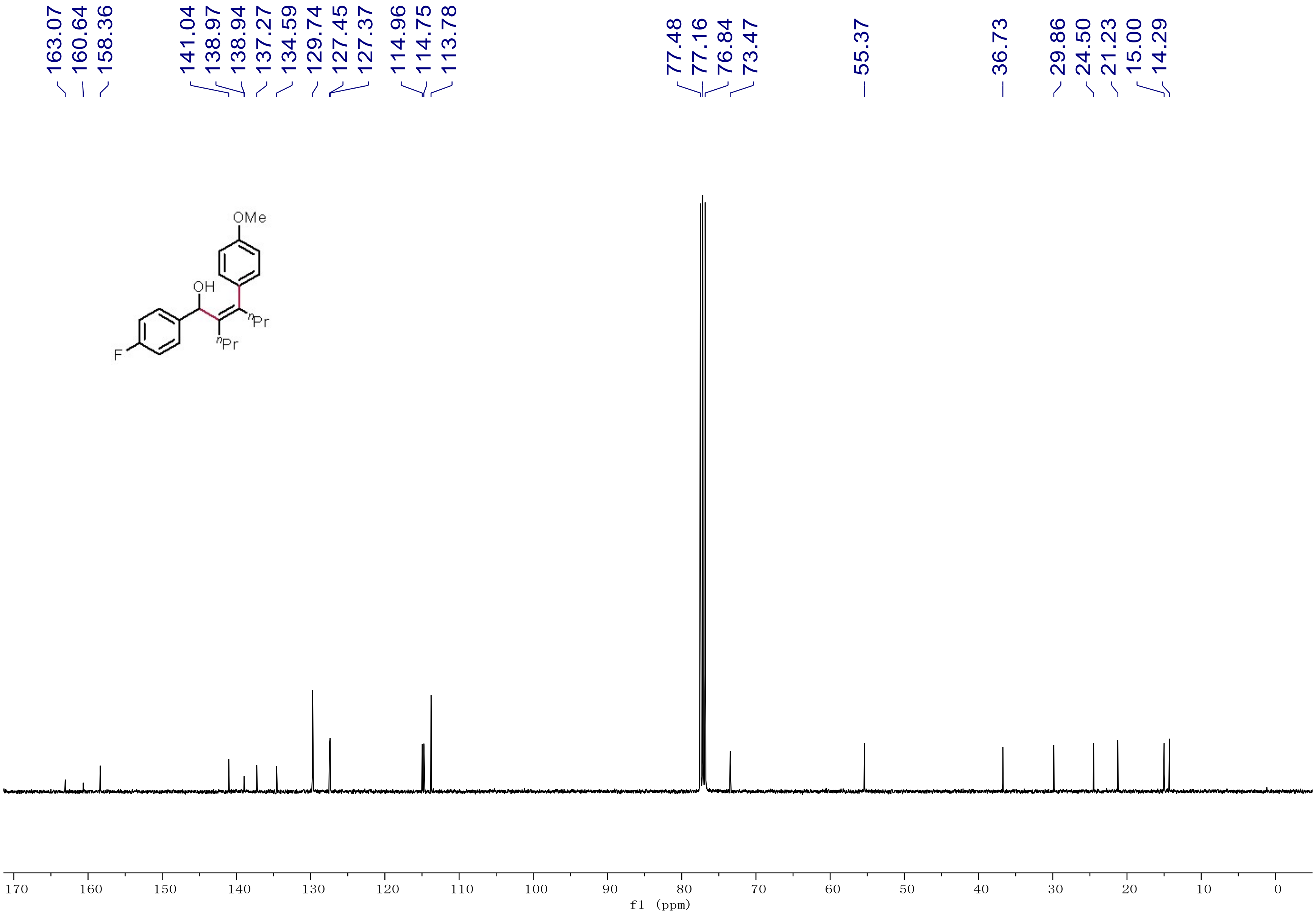

9: (*Z*)-3-(4-methoxyphenyl)-2-propyl-1-(4-(trifluoromethoxy)phenyl)hex-2-en-1-ol (^{1}H NMR, $CDCl_3$, 400 MHz)

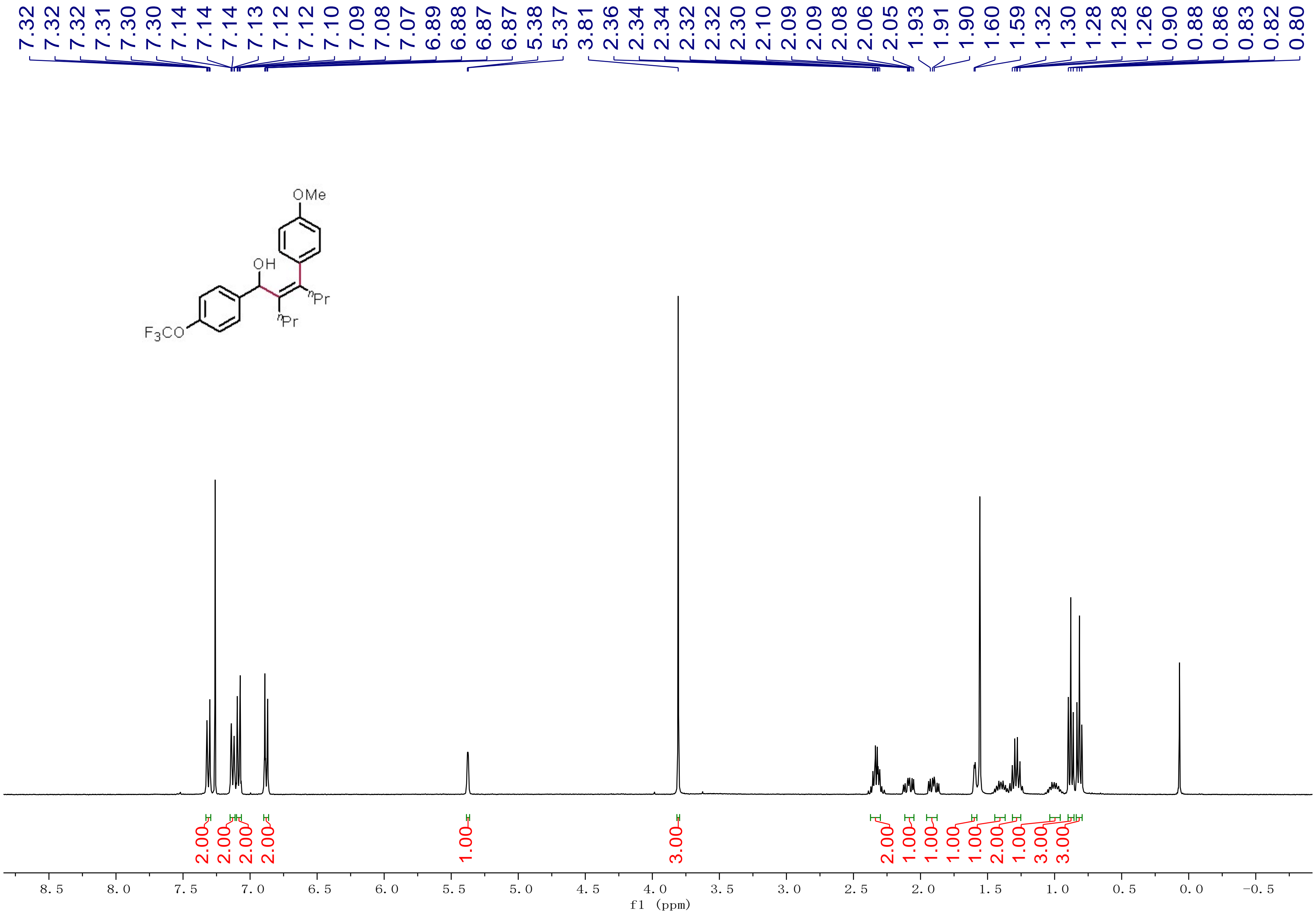

9: (*Z*)-3-(4-methoxyphenyl)-2-propyl-1-(4-(trifluoromethoxy)phenyl)hex-2-en-1-ol (^{13}C NMR, $CDCl_3$, 100 MHz)

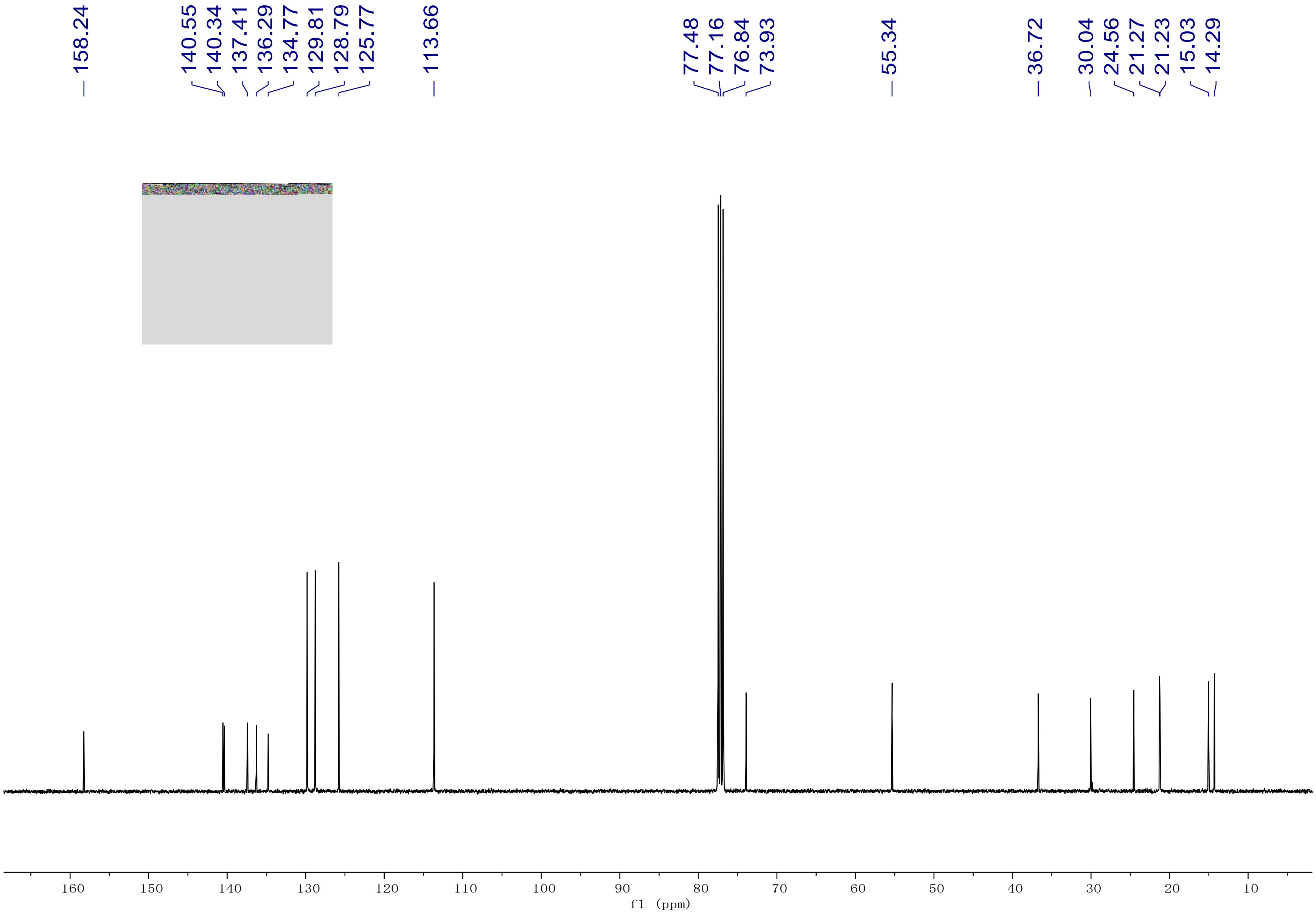

10: (*Z*)-3-(4-methoxyphenyl)-2-propyl-1-(4-(trifluoromethyl)phenyl)hex-2-en-1-ol (^{1}H NMR, $CDCl_3$, 400 MHz)

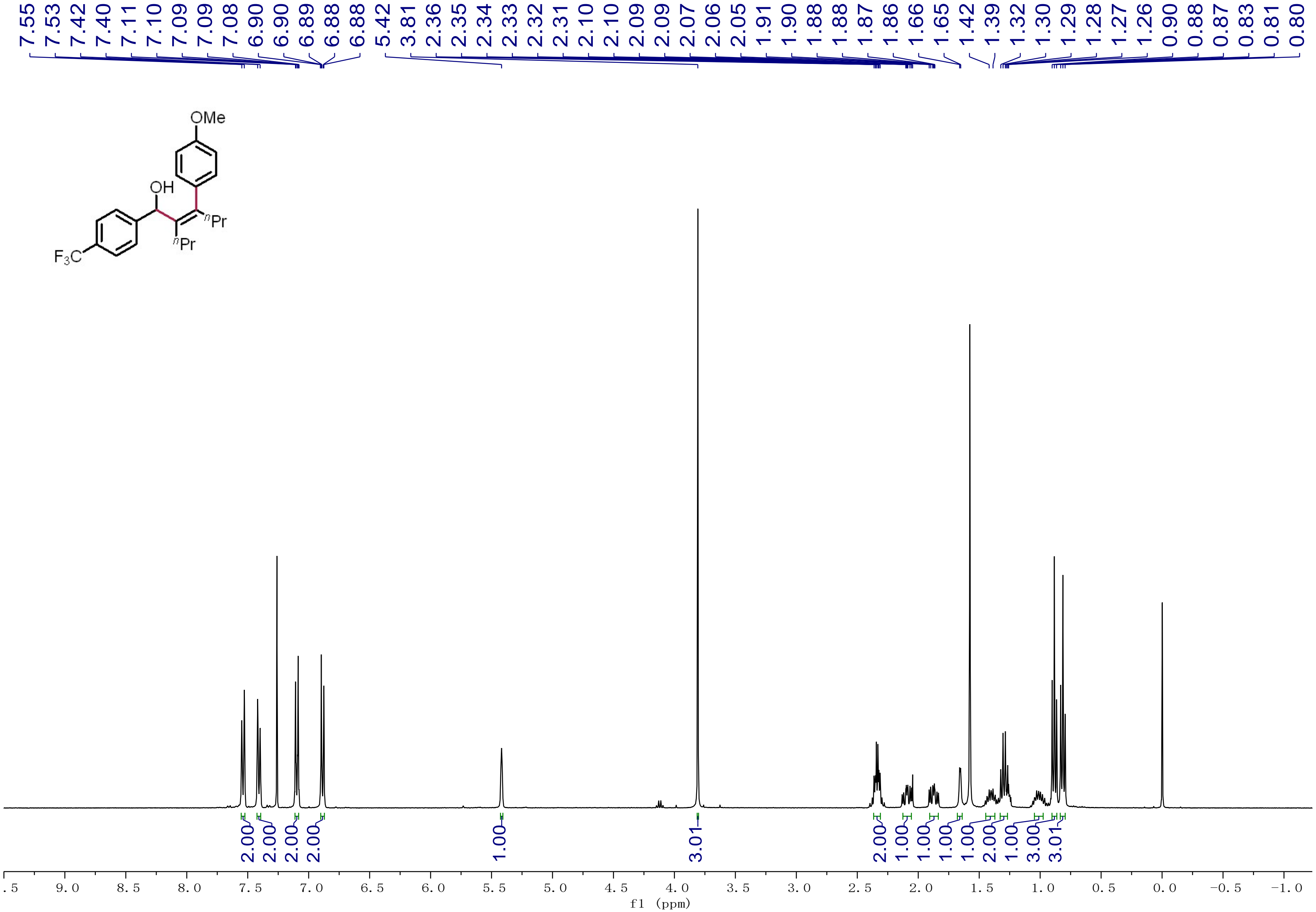

10: (*Z*)-3-(4-methoxyphenyl)-2-propyl-1-(4-(trifluoromethyl)phenyl)hex-2-en-1-ol (^{13}C NMR, $CDCl_3$, 100 MHz)

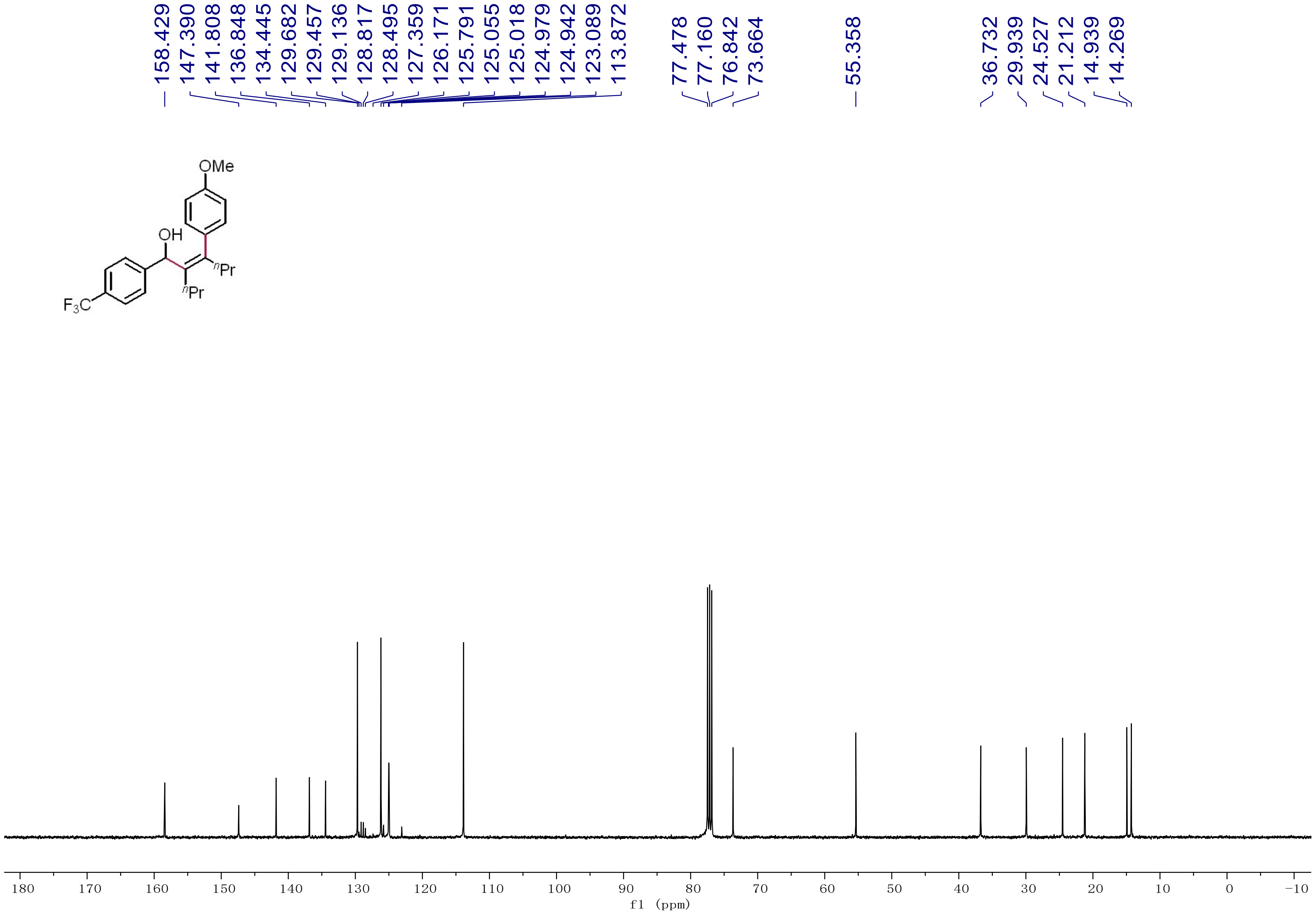

11：methyl (*Z*)-4-(1-hydroxy-3-(4-methoxyphenyl)-2-propylhex-2-en-1-yl)benzoate (^1H NMR, $CDCl_3$, 400 MHz)

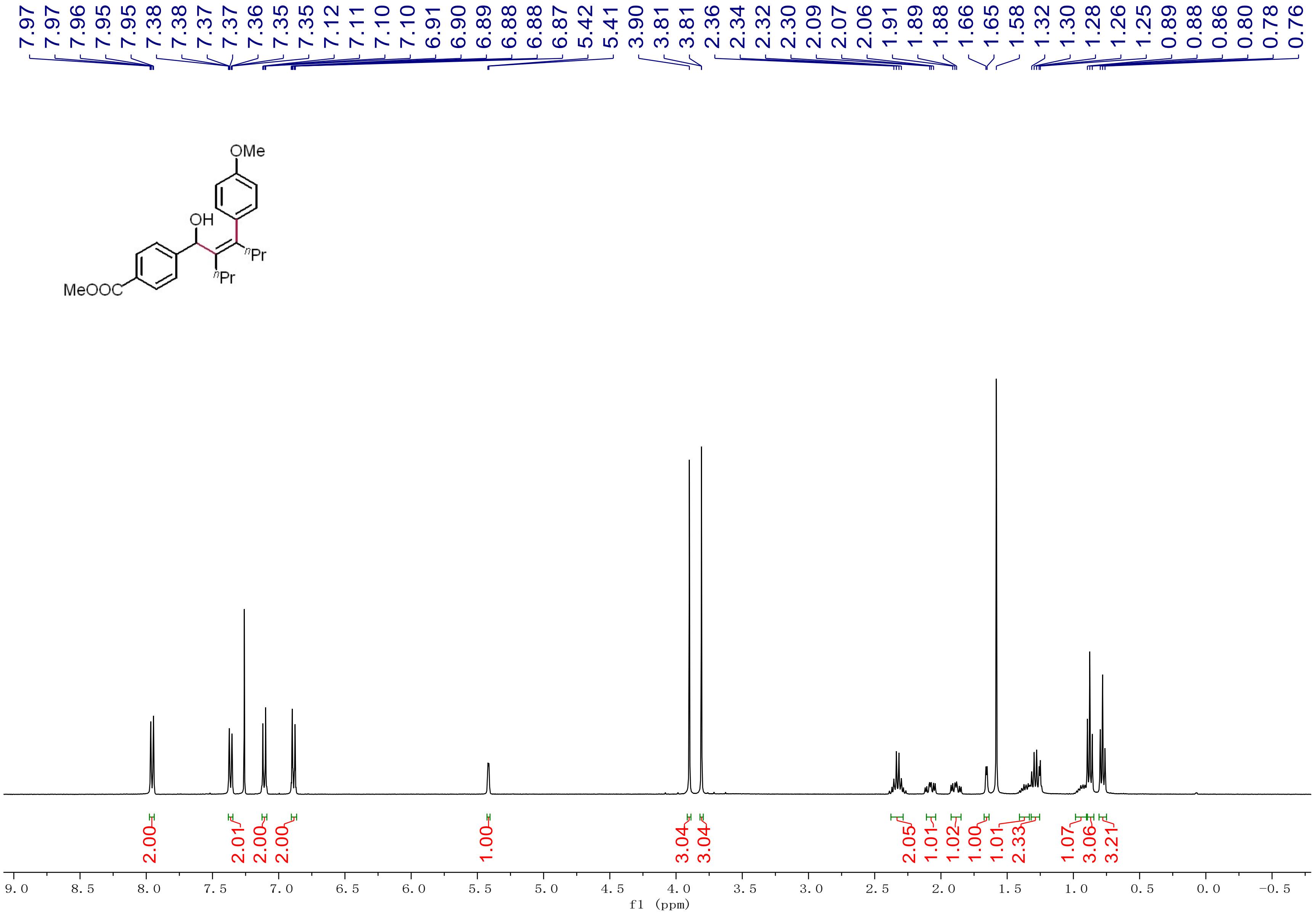

11：methyl (*Z*)-4-(1-hydroxy-3-(4-methoxyphenyl)-2-propylhex-2-en-1-yl)benzoate (^{13}C NMR, $CDCl_3$, 100 MHz)

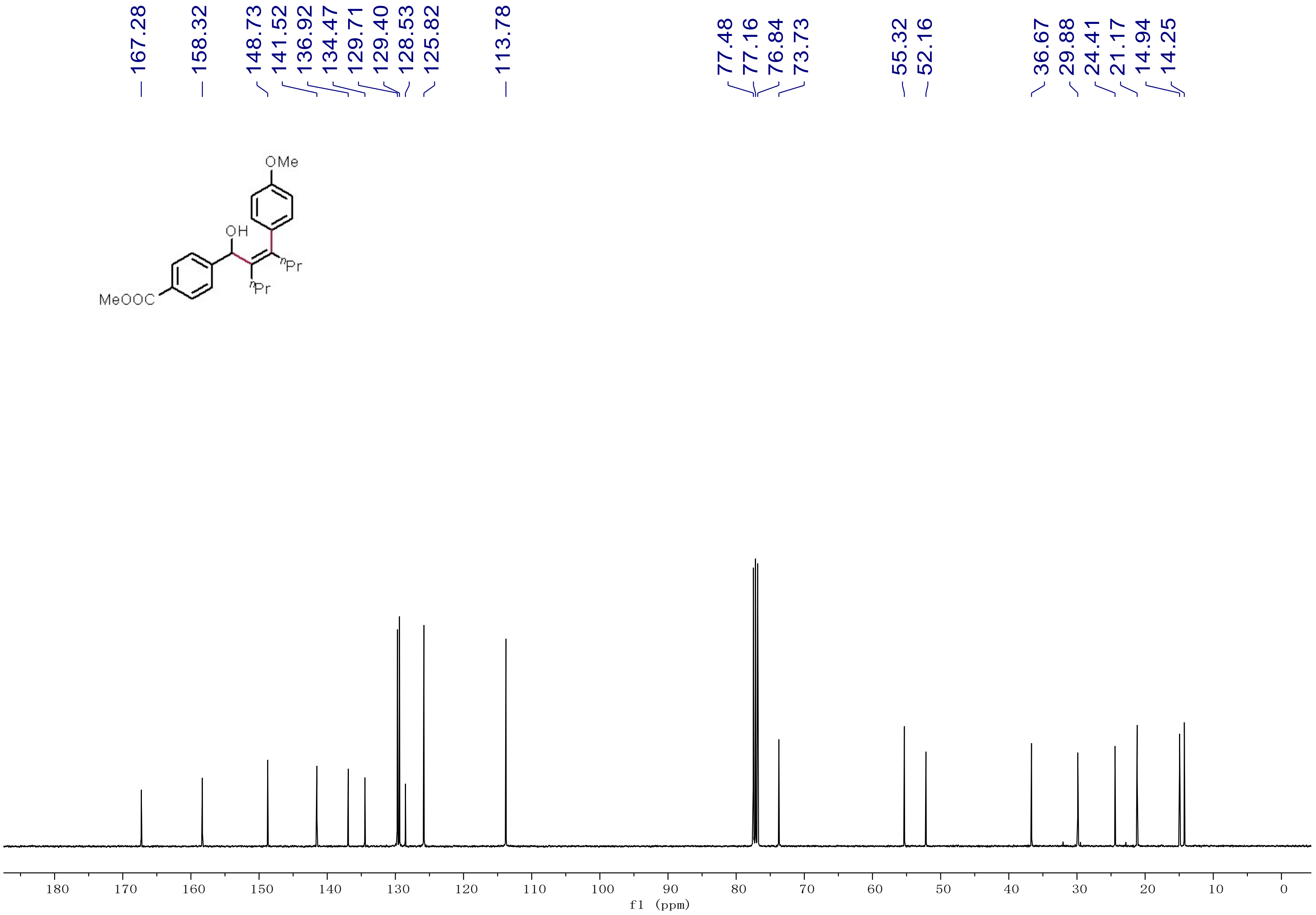

12: (*Z*)-4-(1-hydroxy-3-(4-methoxyphenyl)-2-propylhex-2-en-1-yl)benzonitrile (^{1}H NMR, $CDCl_3$, 400 MHz)

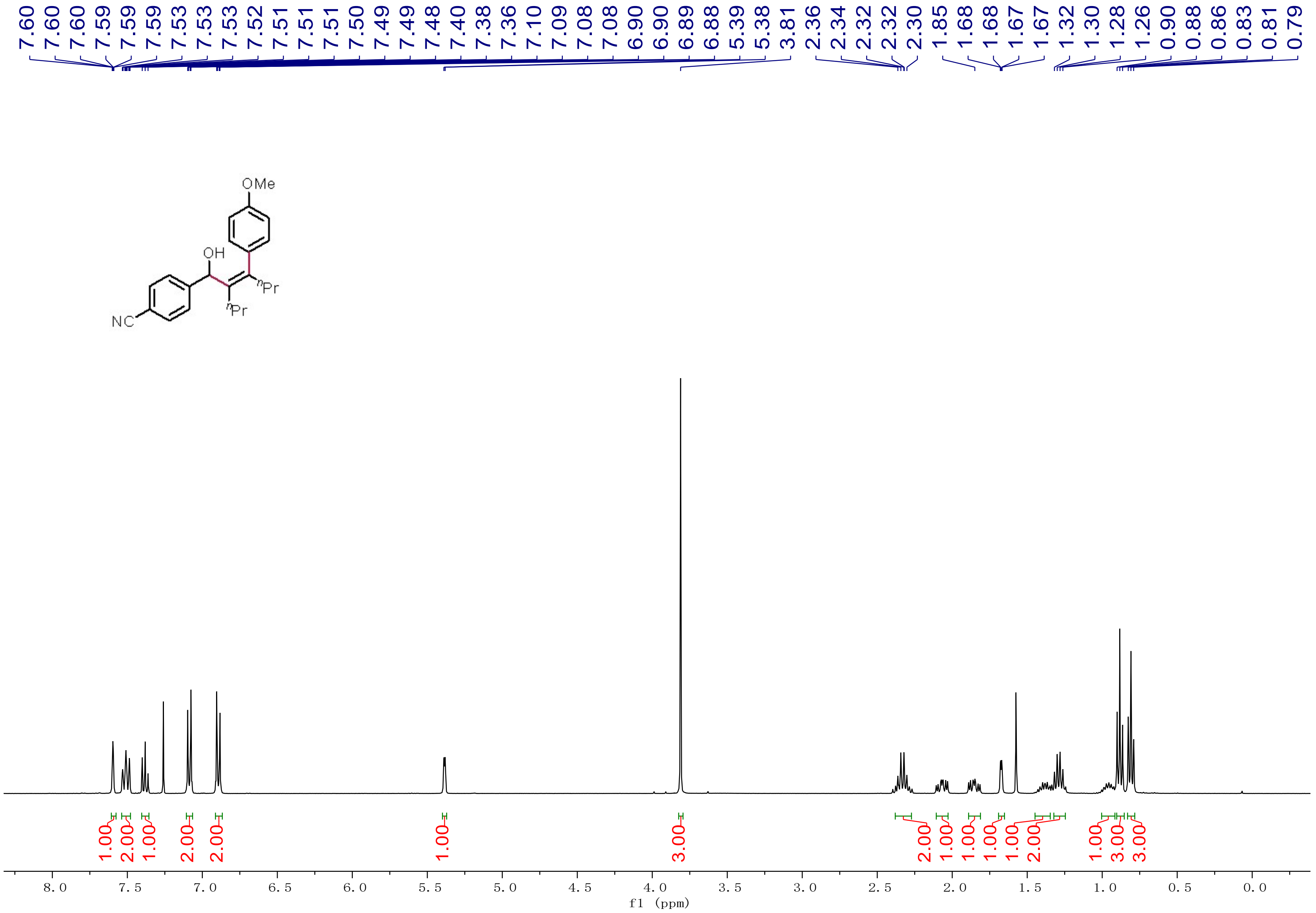

12：(*Z*)-4-(1-hydroxy-3-(4-methoxyphenyl)-2-propylhex-2-en-1-yl)benzonitrile (^{13}C NMR, $CDCl_3$, 100 MHz)

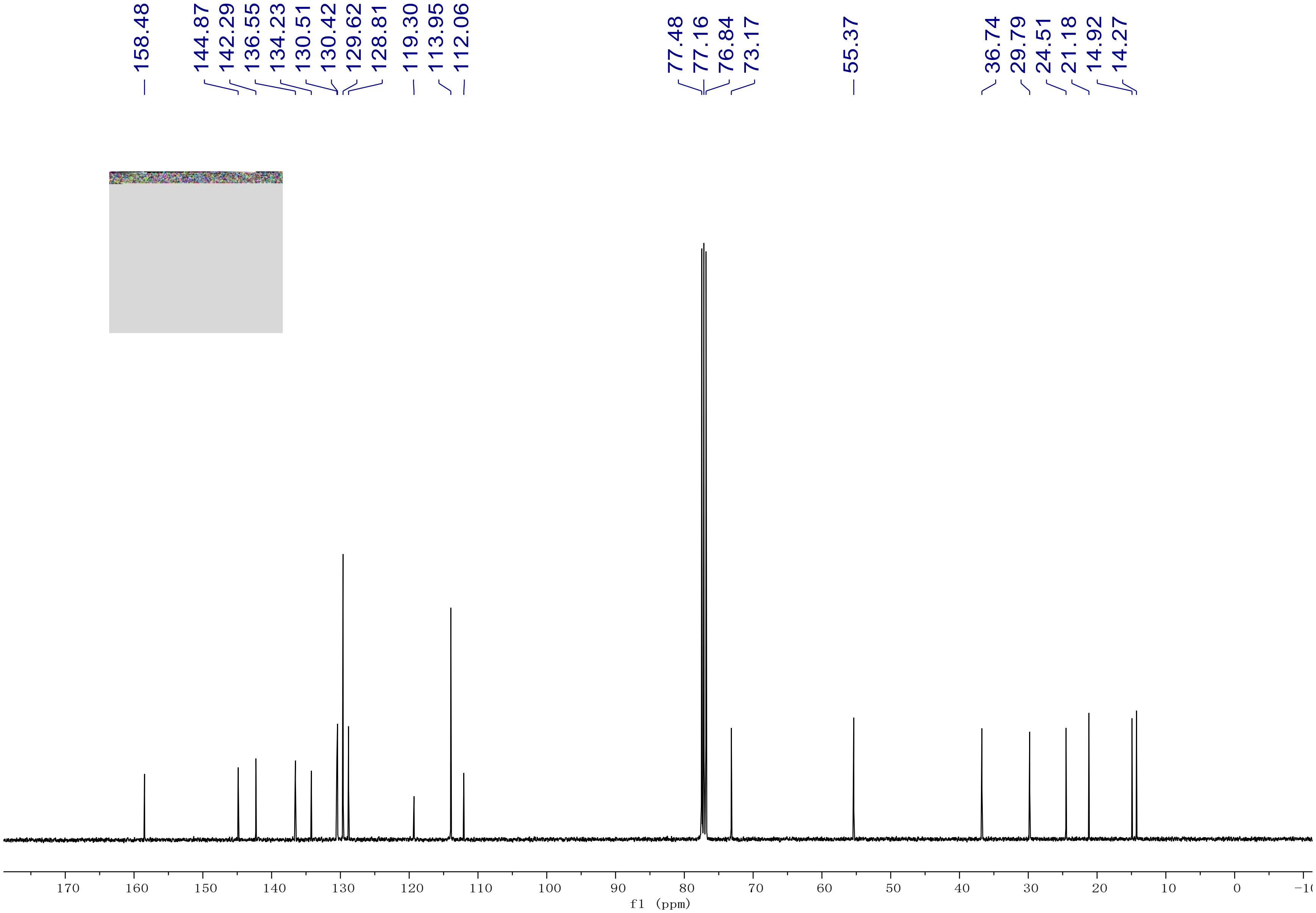

13: (*Z*)-1-(3-fluorophenyl)-3-(4-methoxyphenyl)-2-propylhex-2-en-1-ol (^{1}H NMR, $CDCl_3$, 400 MHz)

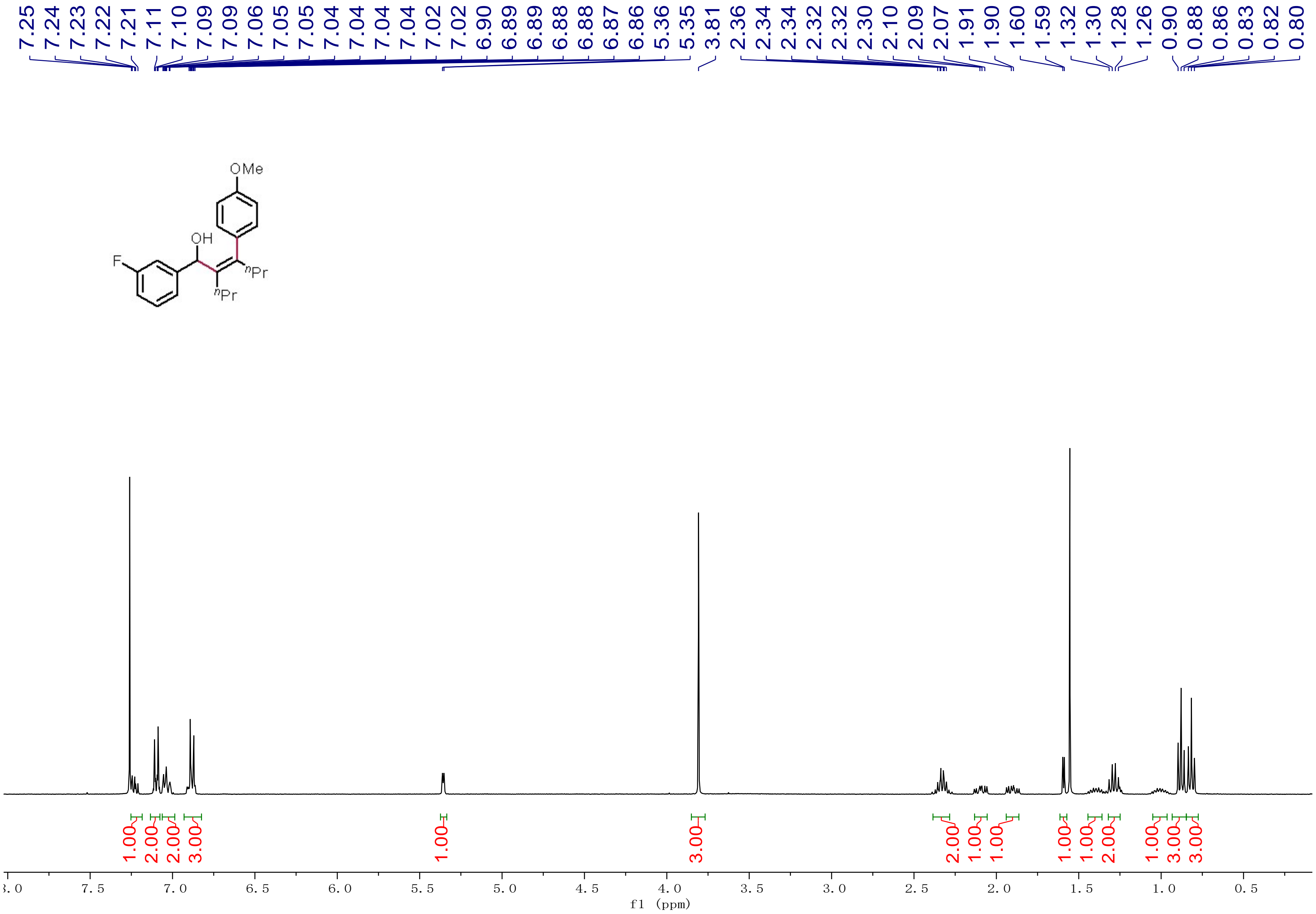

13：(*Z*)-1-(3-fluorophenyl)-3-(4-methoxyphenyl)-2-propylhex-2-en-1-ol (^{13}C NMR, $CDCl_3$, 100 MHz)

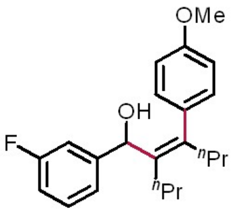

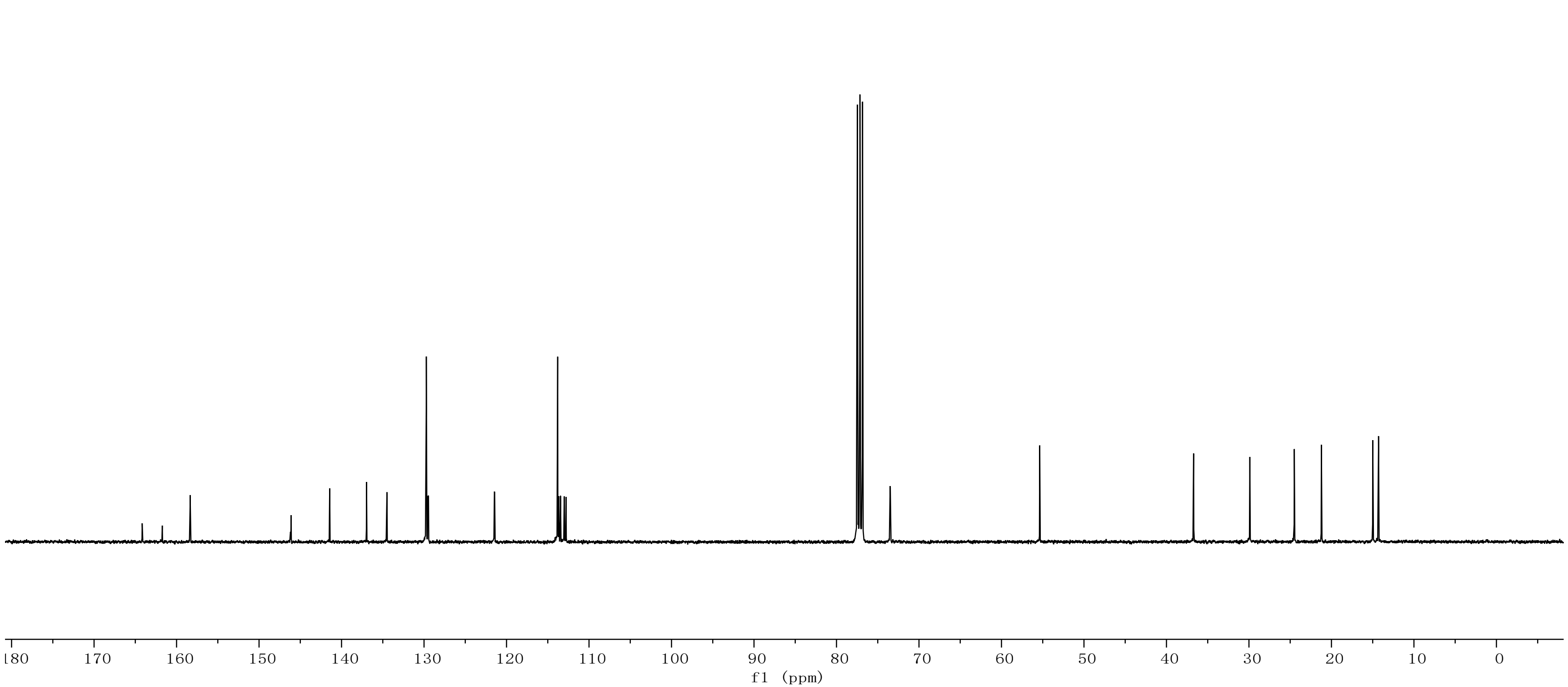

14: (*Z*)-1-(3-chlorophenyl)-3-(4-methoxyphenyl)-2-propylhex-2-en-1-ol (^{1}H NMR, $CDCl_3$, 400 MHz)

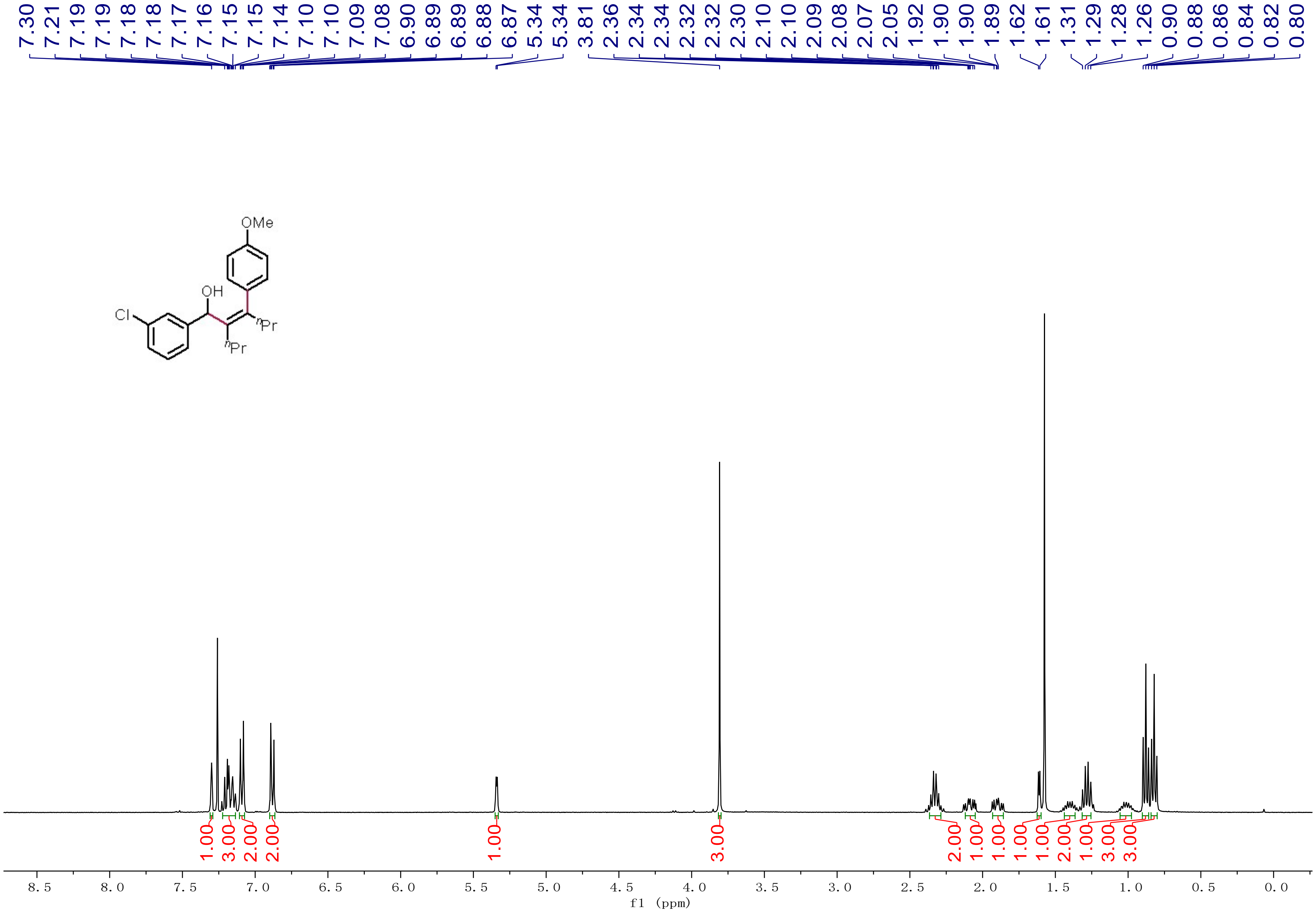

14: (*Z*)-1-(3-chlorophenyl)-3-(4-methoxyphenyl)-2-propylhex-2-en-1-ol (^{13}C NMR, $CDCl_3$, 100 MHz)

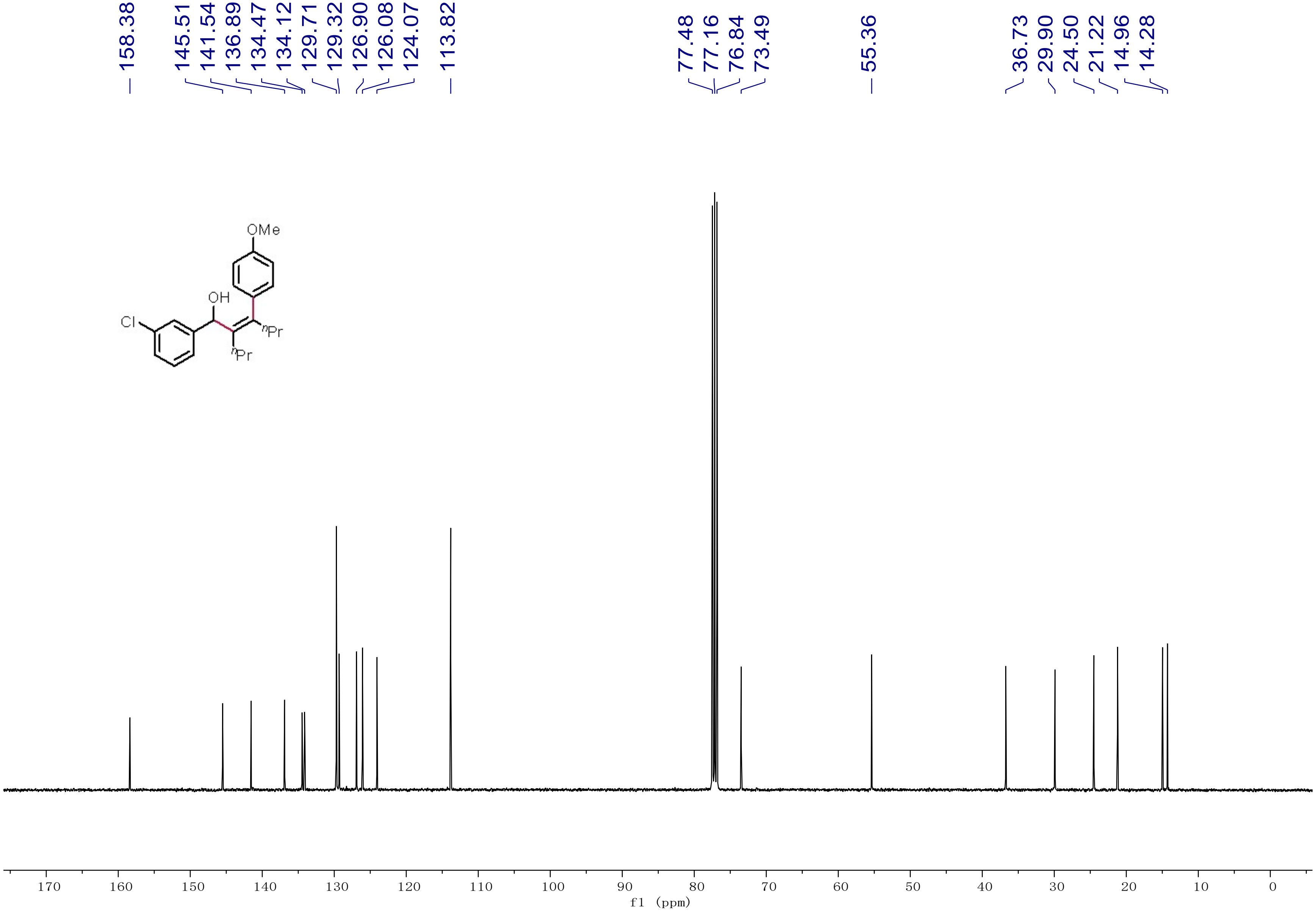

15: (*Z*)-3-(4-methoxyphenyl)-2-propyl-1-(m-tolyl)hex-2-en-1-ol (^{1}H NMR, $CDCl_3$, 400 MHz)

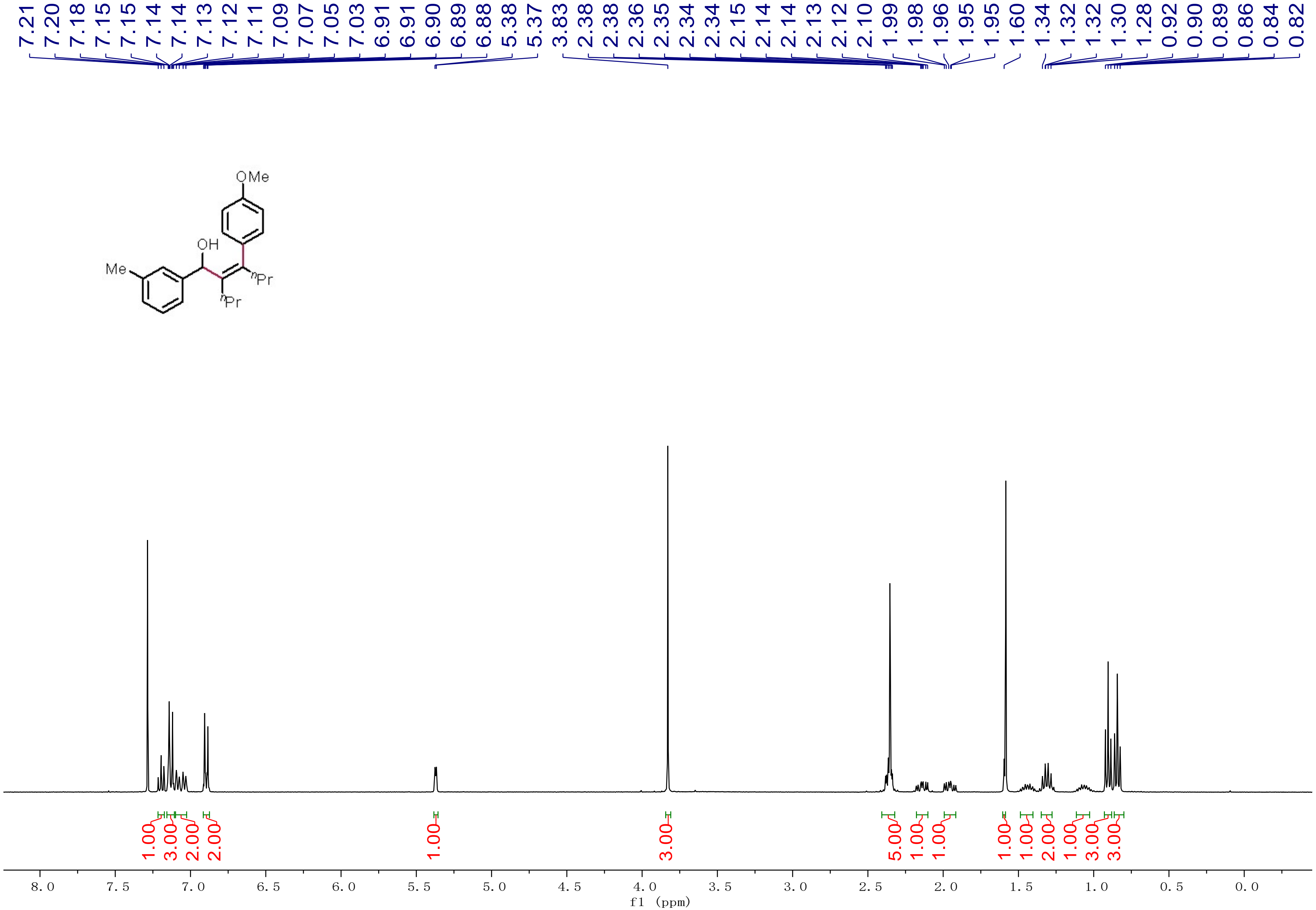

15: (*Z*)-3-(4-methoxyphenyl)-2-propyl-1-(m-tolyl)hex-2-en-1-ol (^{13}C NMR, $CDCl_3$, 100 MHz)

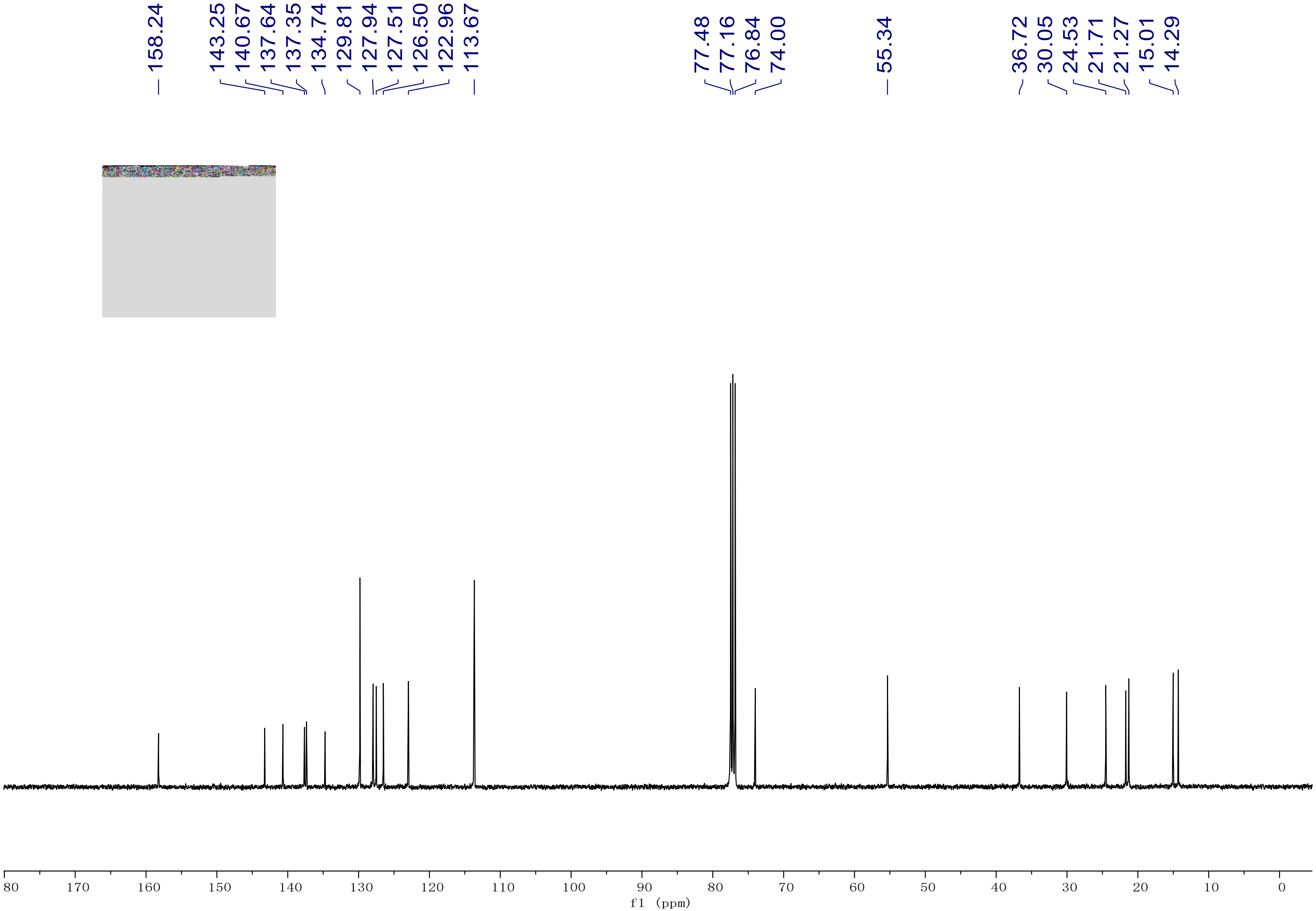

16: (*Z*)-3-(4-methoxyphenyl)-2-propyl-1-(o-tolyl)hex-2-en-1-ol (^{1}H NMR, $CDCl_3$, 400 MHz)

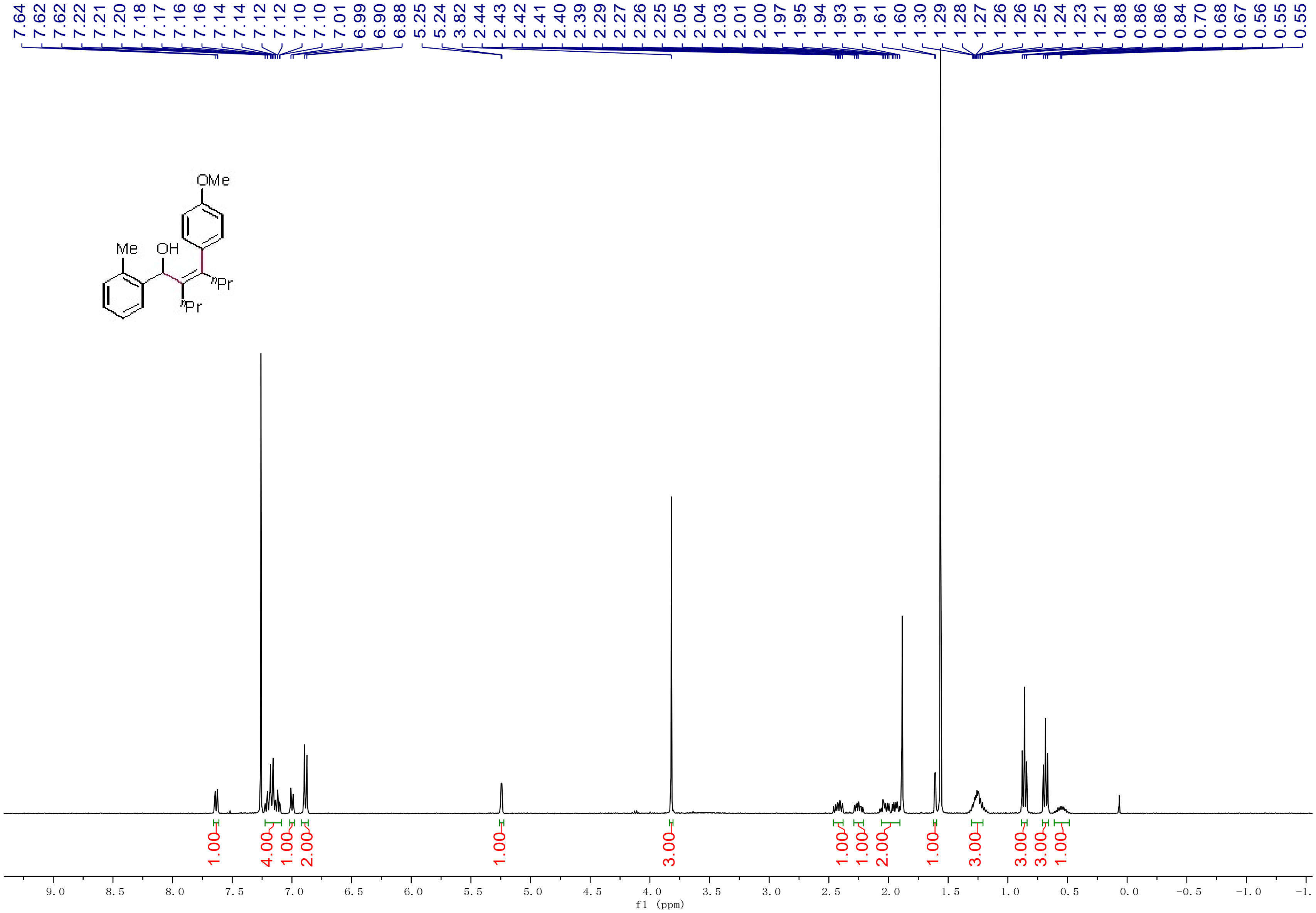

16: (Z)-3-(4-methoxyphenyl)-2-propyl-1-(o-tolyl)hex-2-en-1-ol (^{13}C NMR, $CDCl_3$, 100 MHz)

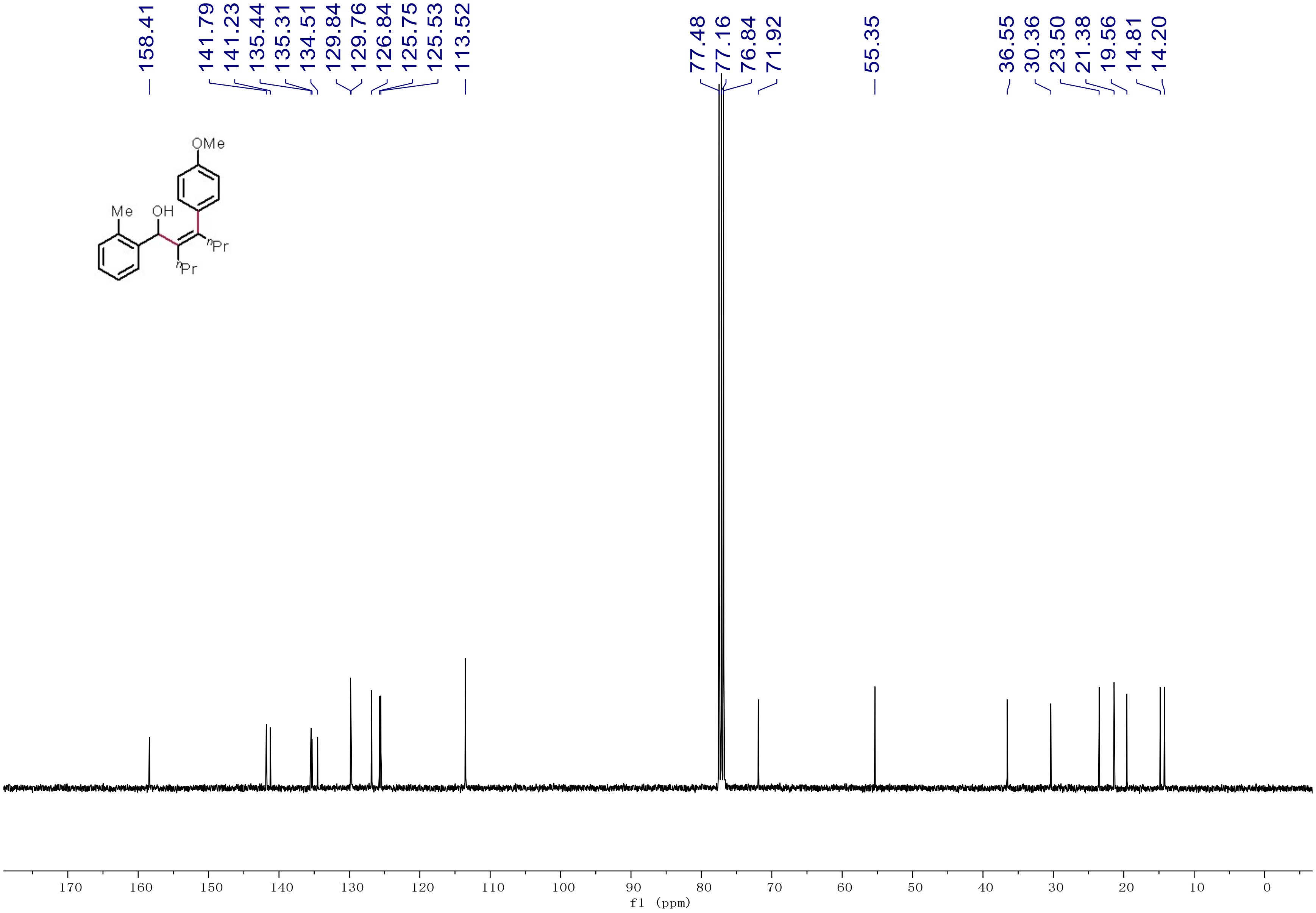

17: (Z)-1-(2-methoxyphenyl)-3-(4-methoxyphenyl)-2-propylhex-2-en-1-ol (^{1}H NMR, $CDCl_3$, 400 MHz)

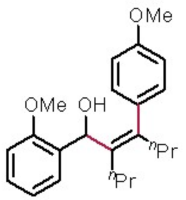

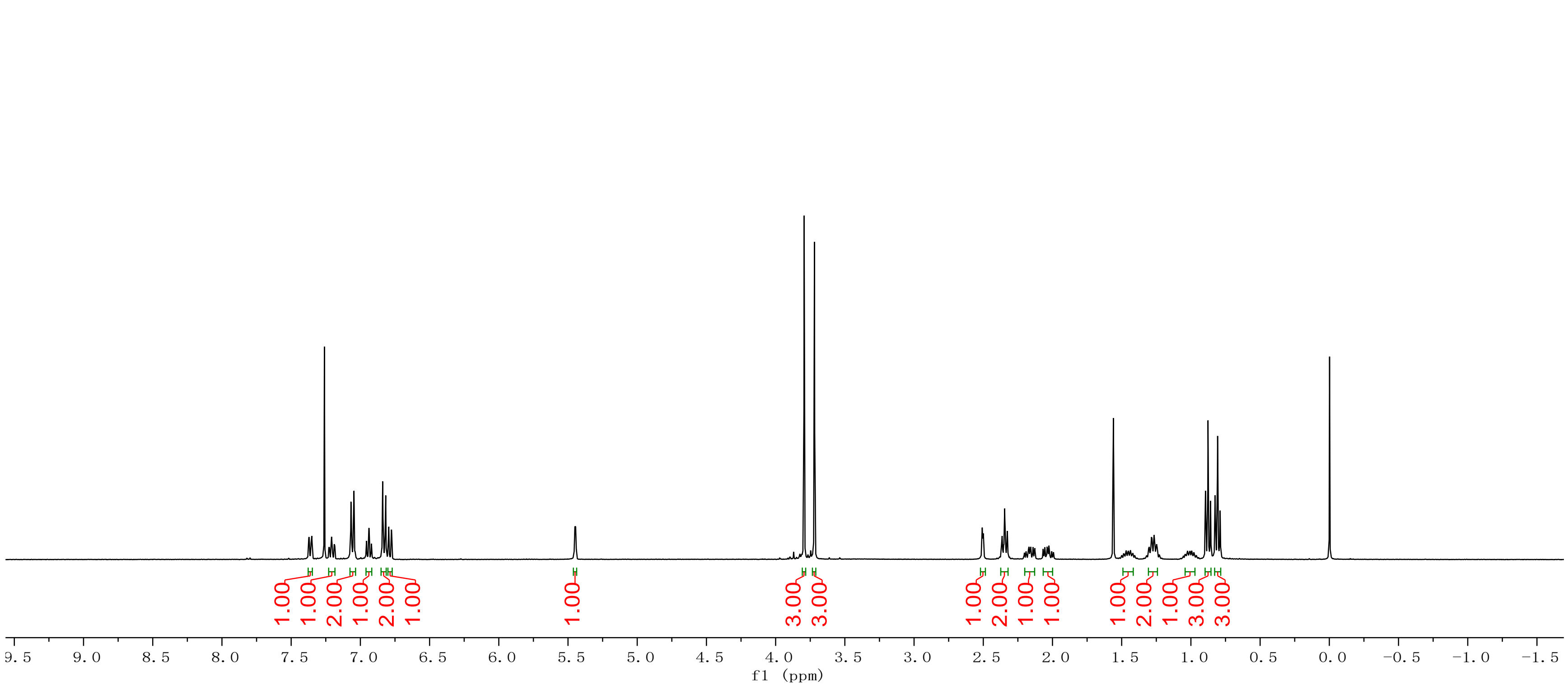

17: (Z)-1-(2-methoxyphenyl)-3-(4-methoxyphenyl)-2-propylhex-2-en-1-ol (^{13}C NMR, $CDCl_3$, 100 MHz)

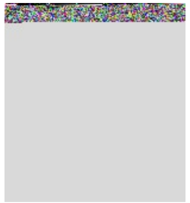

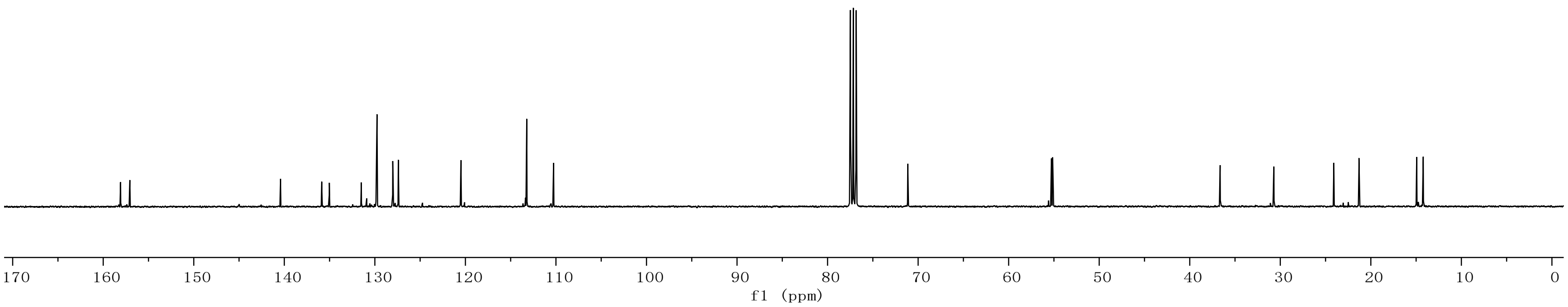

18: (Z)-1-(3,4-dimethylphenyl)-3-(4-methoxyphenyl)-2-propylhex-2-en-1-ol (^{1}H NMR, $CDCl_3$, 400 MHz)

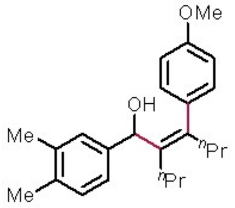

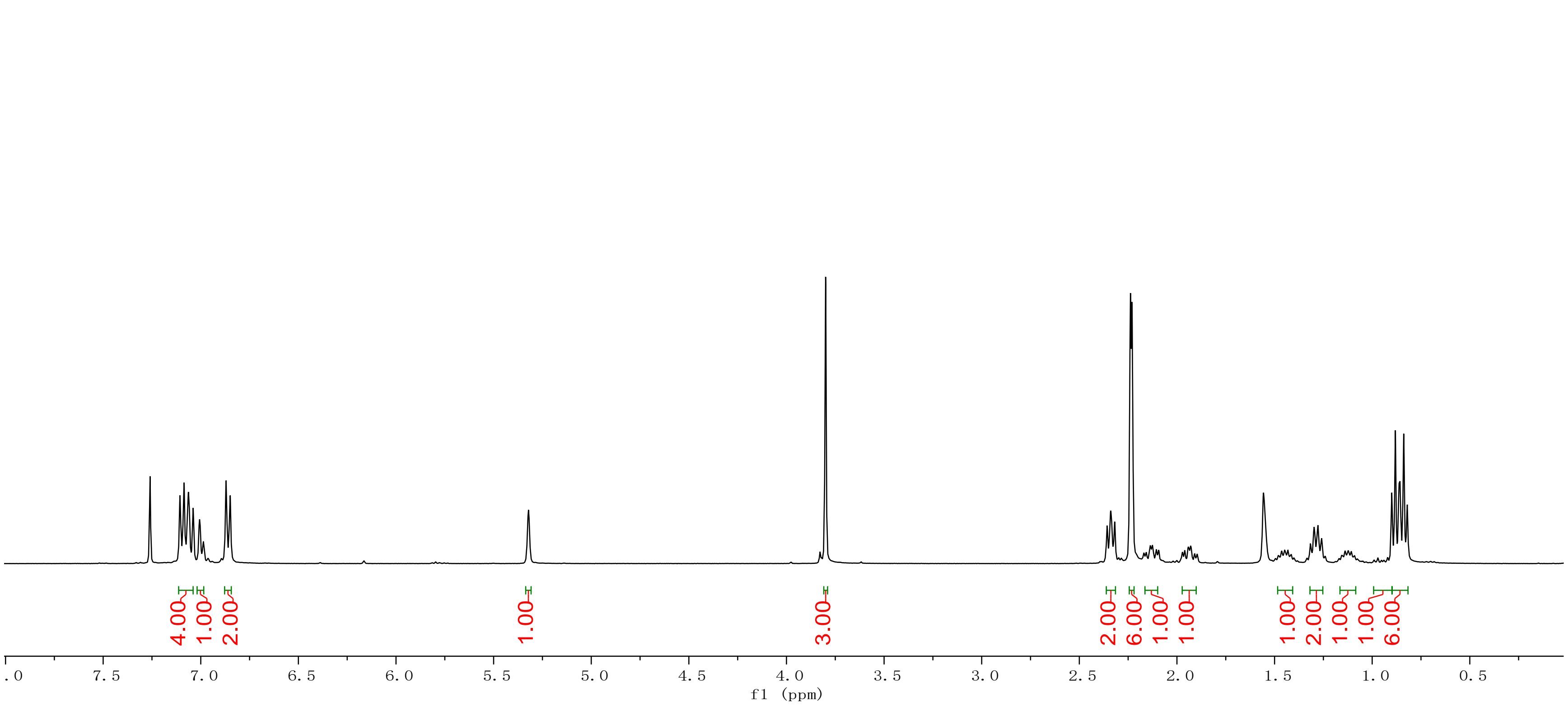

18: (Z)-1-(3,4-dimethylphenyl)-3-(4-methoxyphenyl)-2-propylhex-2-en-1-ol (^{13}C NMR, $CDCl_3$, 100 MHz)

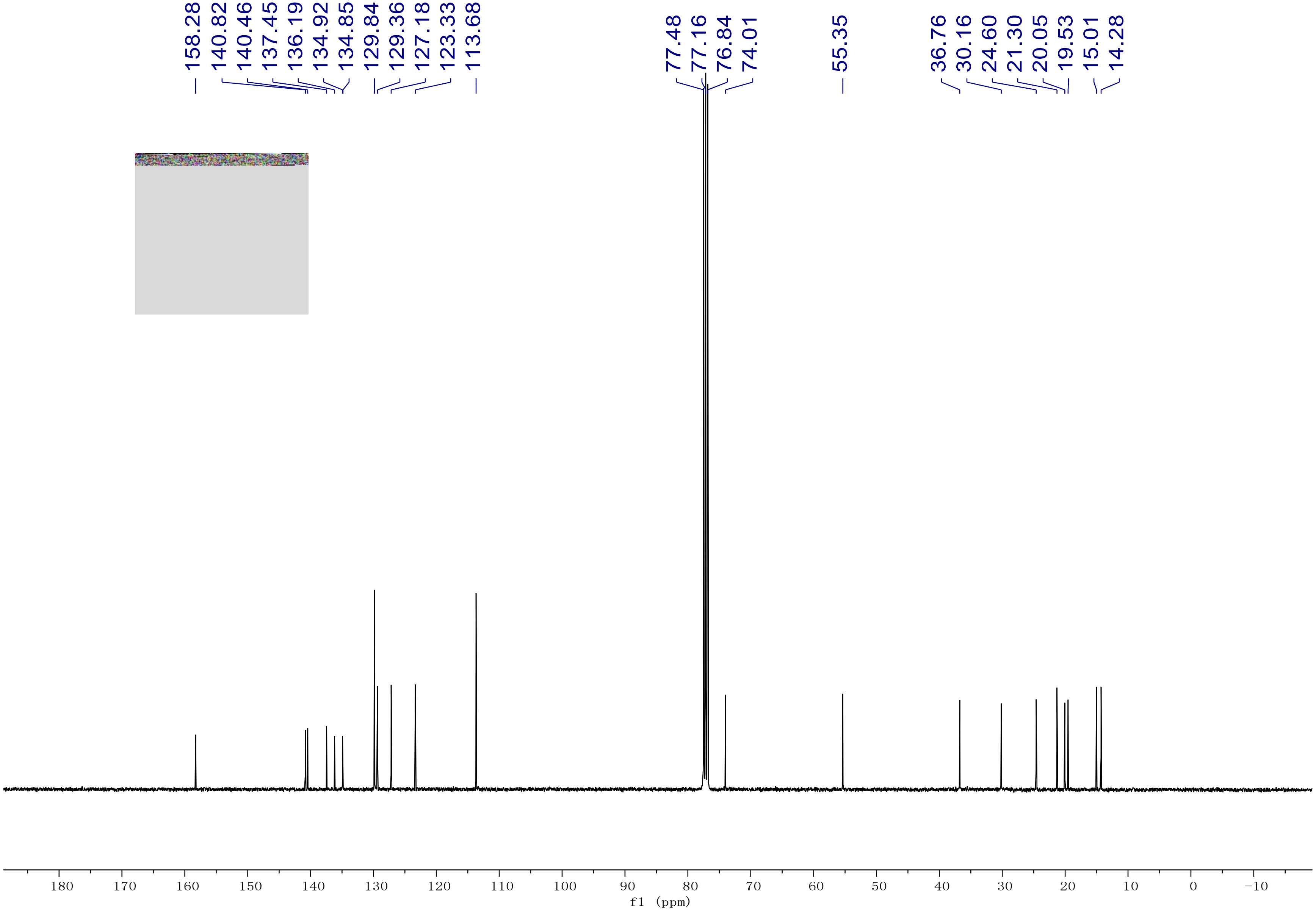

19：(*Z*)-1-(3,4-dimethoxyphenyl)-3-(4-methoxyphenyl)-2-propylhex-2-en-1-ol (^{1}H NMR, $CDCl_3$, 400 MHz)

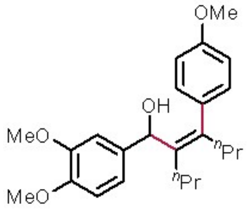

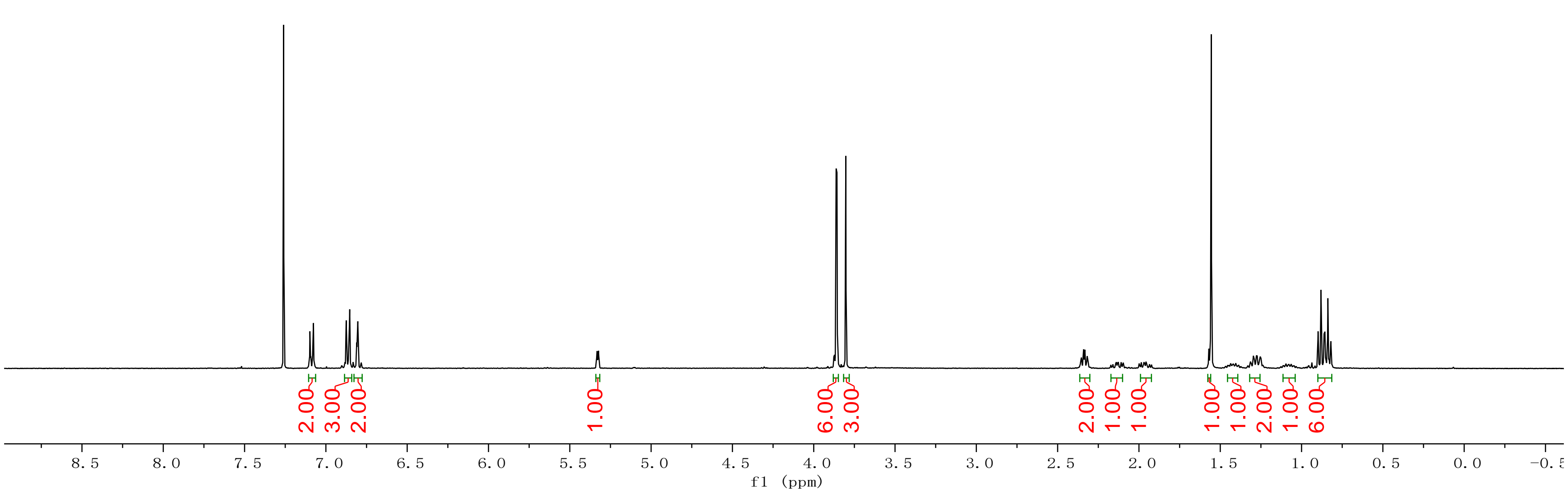

19：(*Z*)-1-(3,4-dimethoxyphenyl)-3-(4-methoxyphenyl)-2-propylhex-2-en-1-ol (^{13}C NMR, $CDCl_3$, 100 MHz)

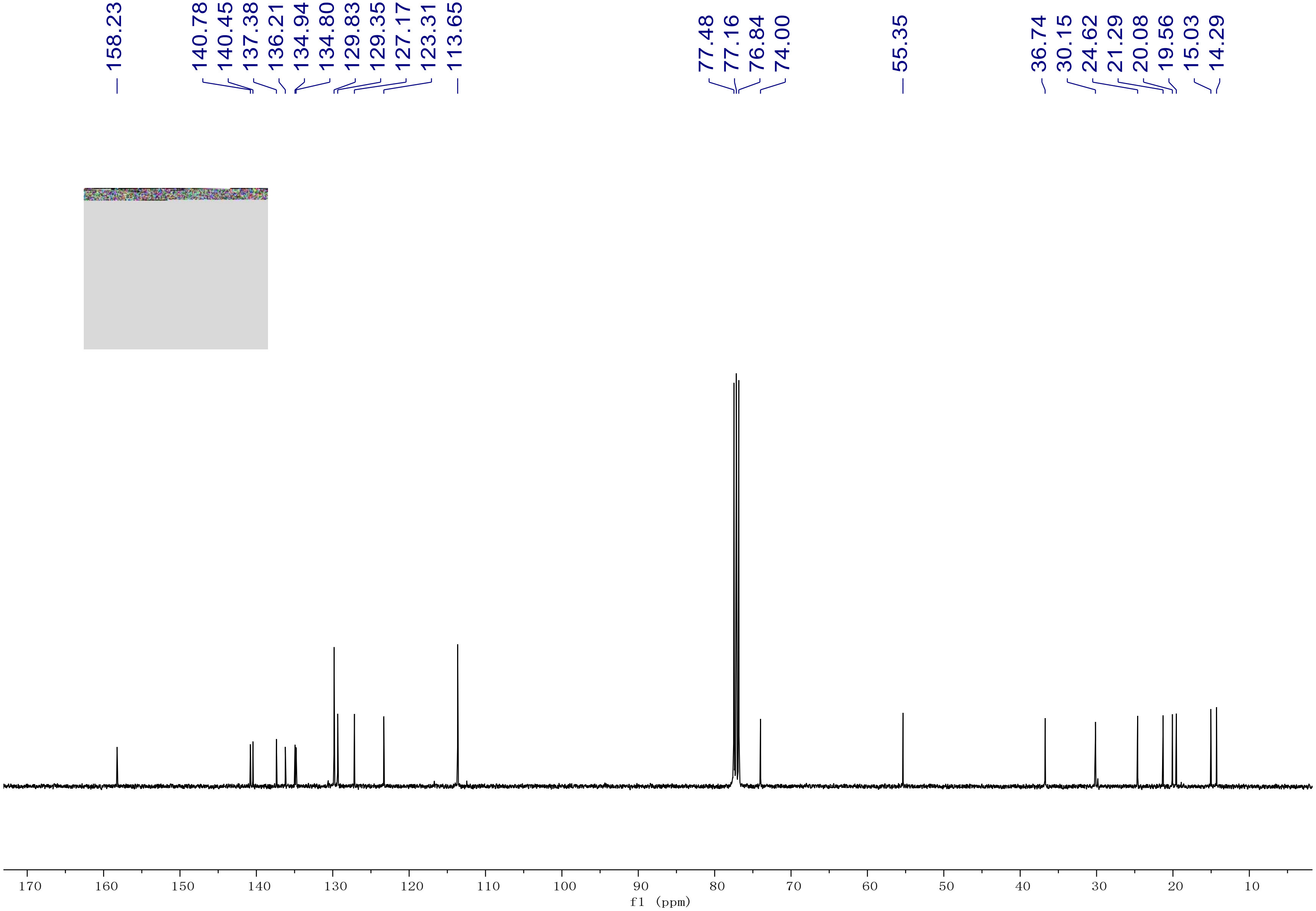

20：(*Z*)-1-(benzo[d][1,3]dioxol-5-yl)-3-(4-methoxyphenyl)-2-propylhex-2-en-1-ol (^{1}H NMR, $CDCl_3$, 400 MHz)

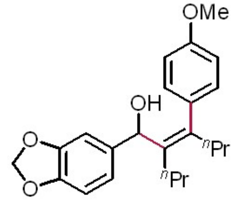

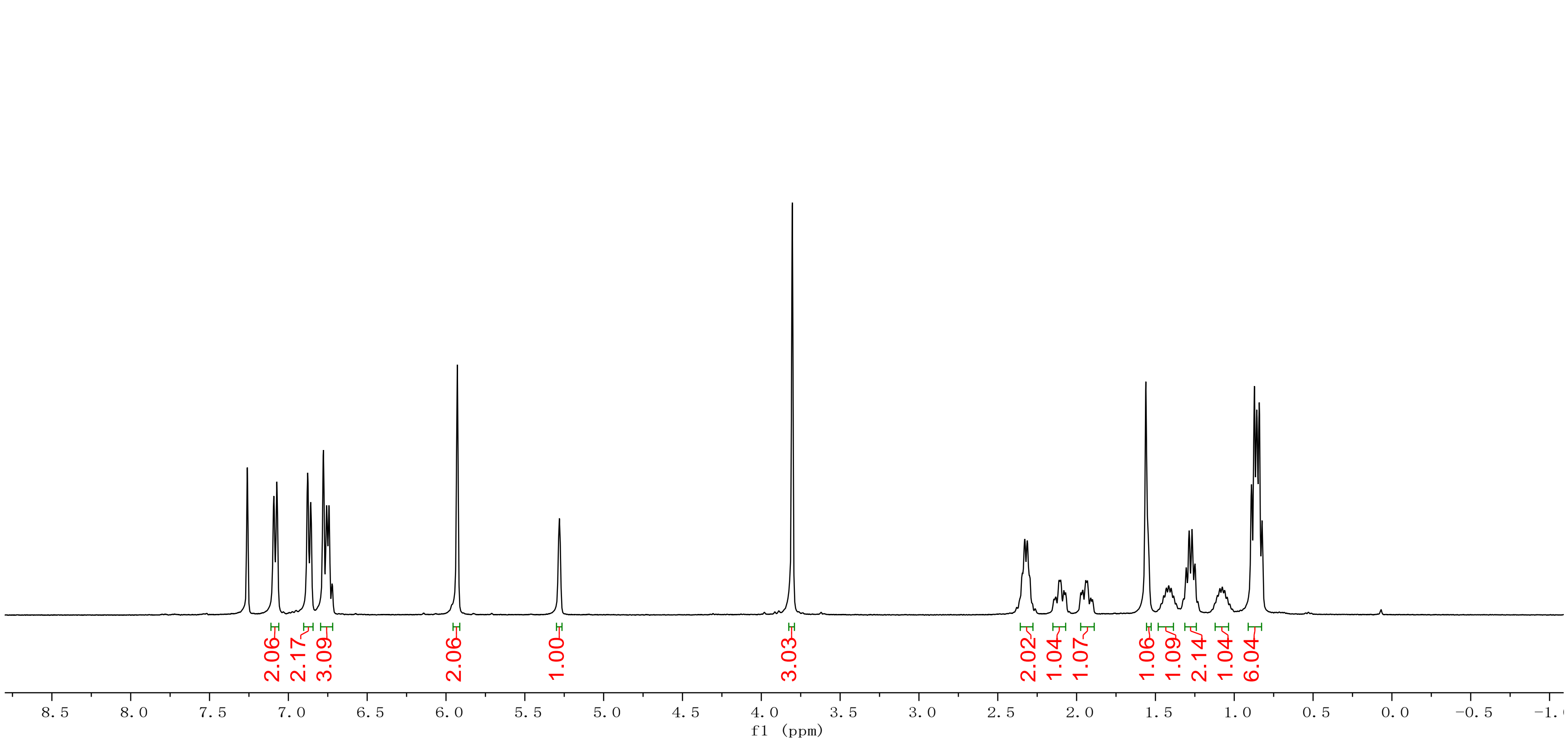

20：(*Z*)-1-(benzo[d][1,3]dioxol-5-yl)-3-(4-methoxyphenyl)-2-propylhex-2-en-1-ol (^{13}C NMR, $CDCl_3$, 100 MHz)

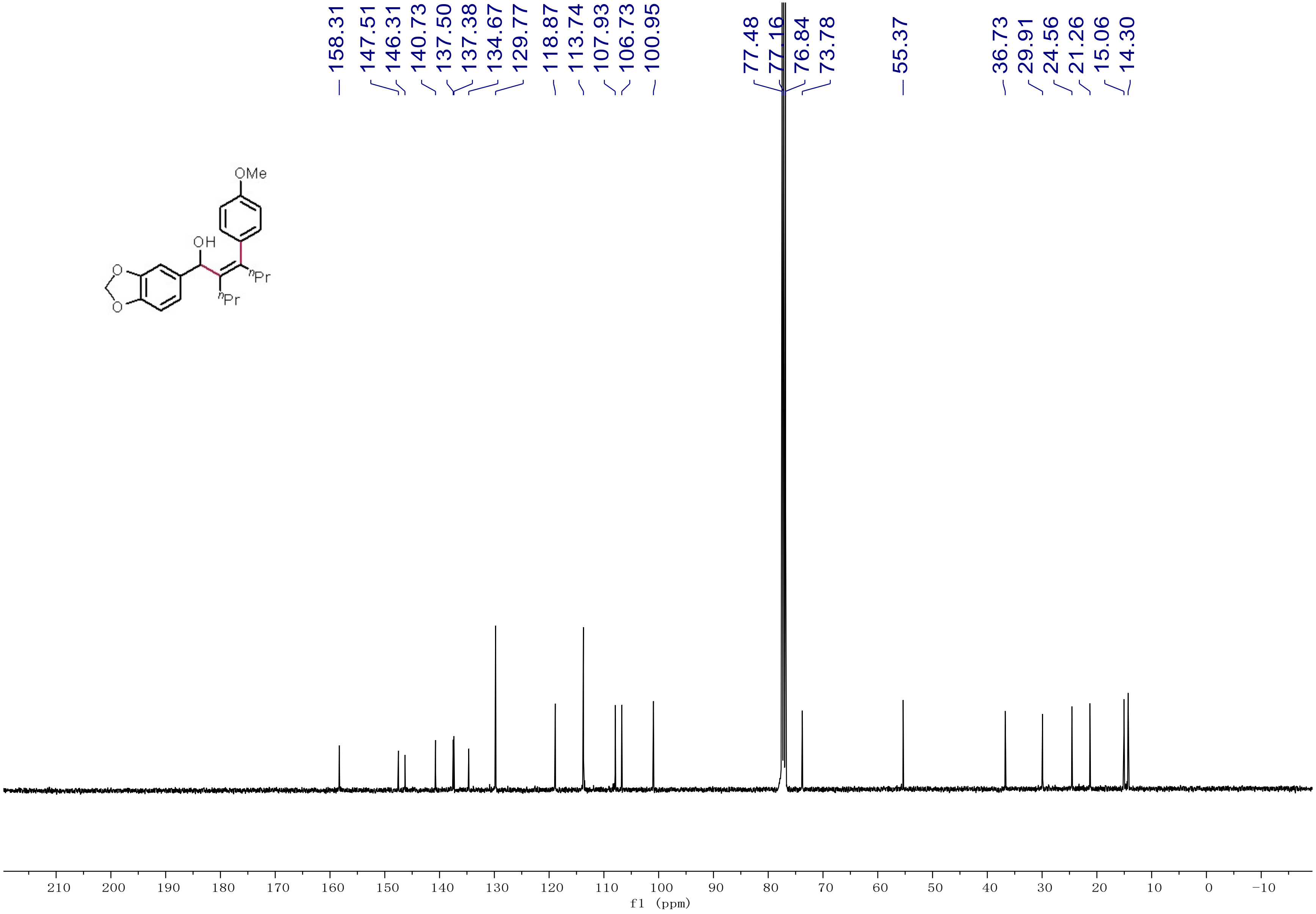

21：(*Z*)-1-(4-chloro-3-fluorophenyl)-3-(4-methoxyphenyl)-2-propylhex-2-en-1-ol (^{1}H NMR, $CDCl_3$, 400 MHz)

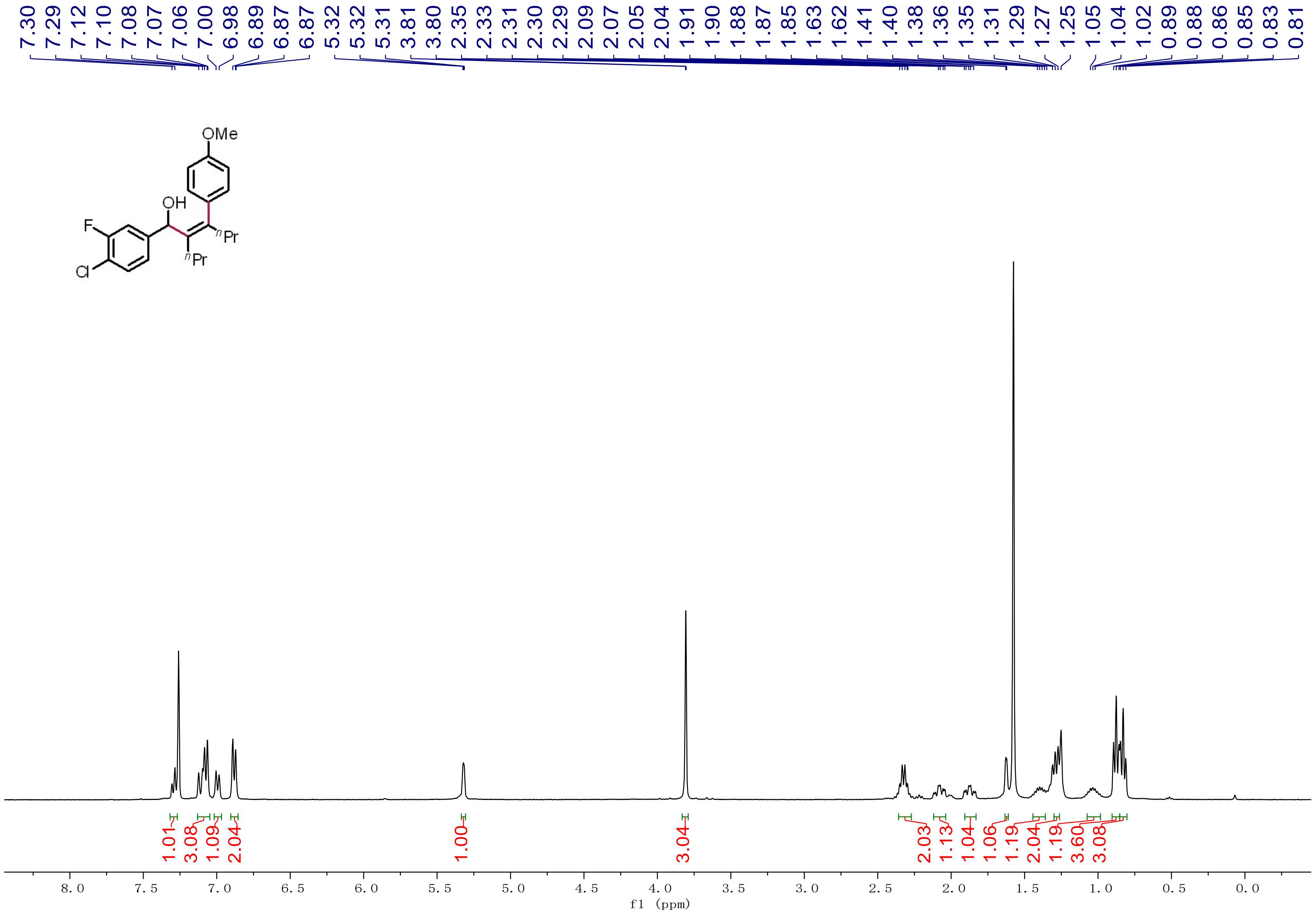

21：(*Z*)-1-(4-chloro-3-fluorophenyl)-3-(4-methoxyphenyl)-2-propylhex-2-en-1-ol (^{13}C NMR, $CDCl_3$, 100 MHz)

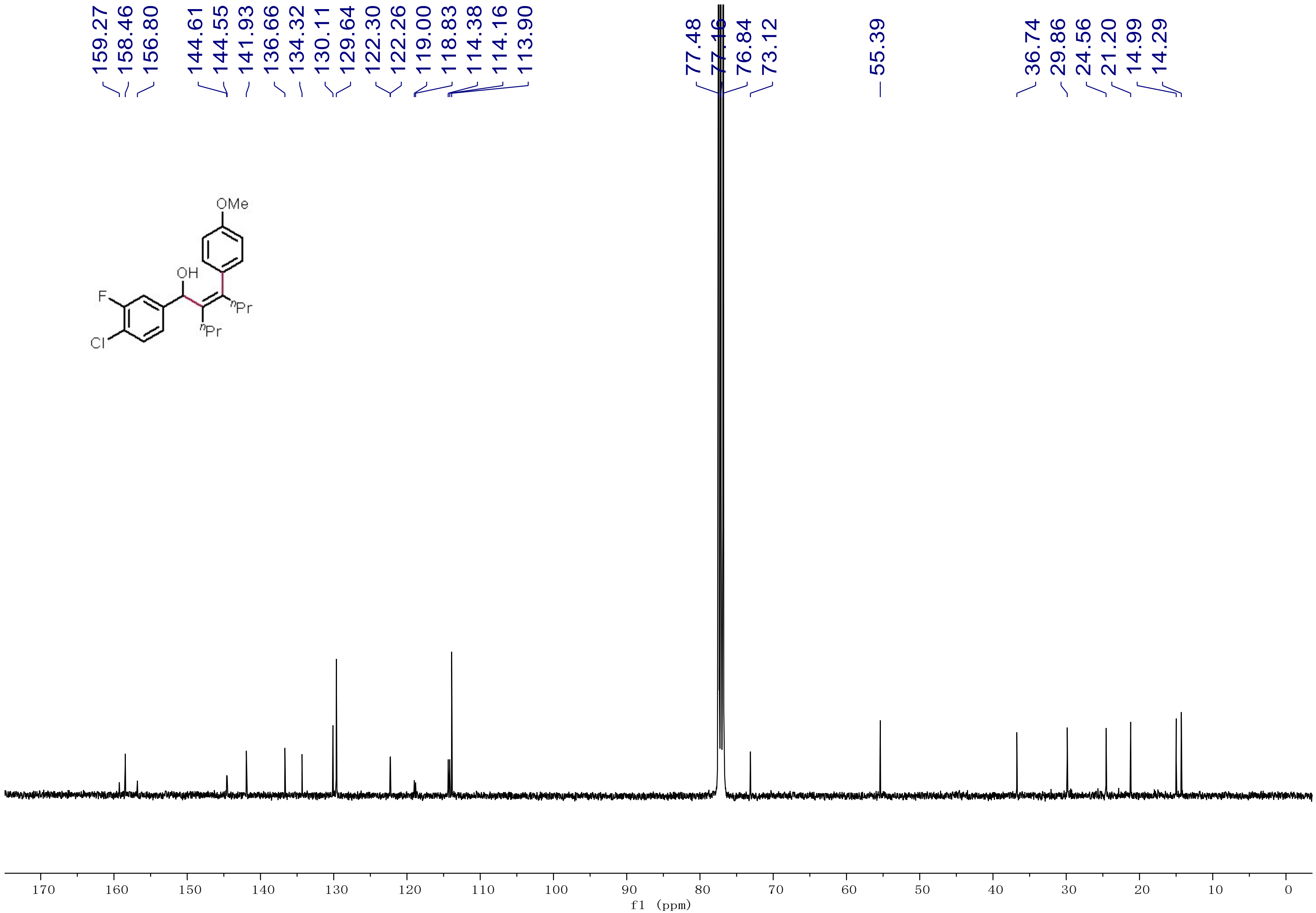

22: (*Z*)-3-(4-methoxyphenyl)-1-(naphthalen-2-yl)-2-propylhex-2-en-1-ol (^{1}H NMR, $CDCl_3$, 400 MHz)

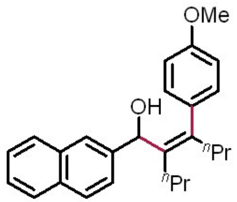

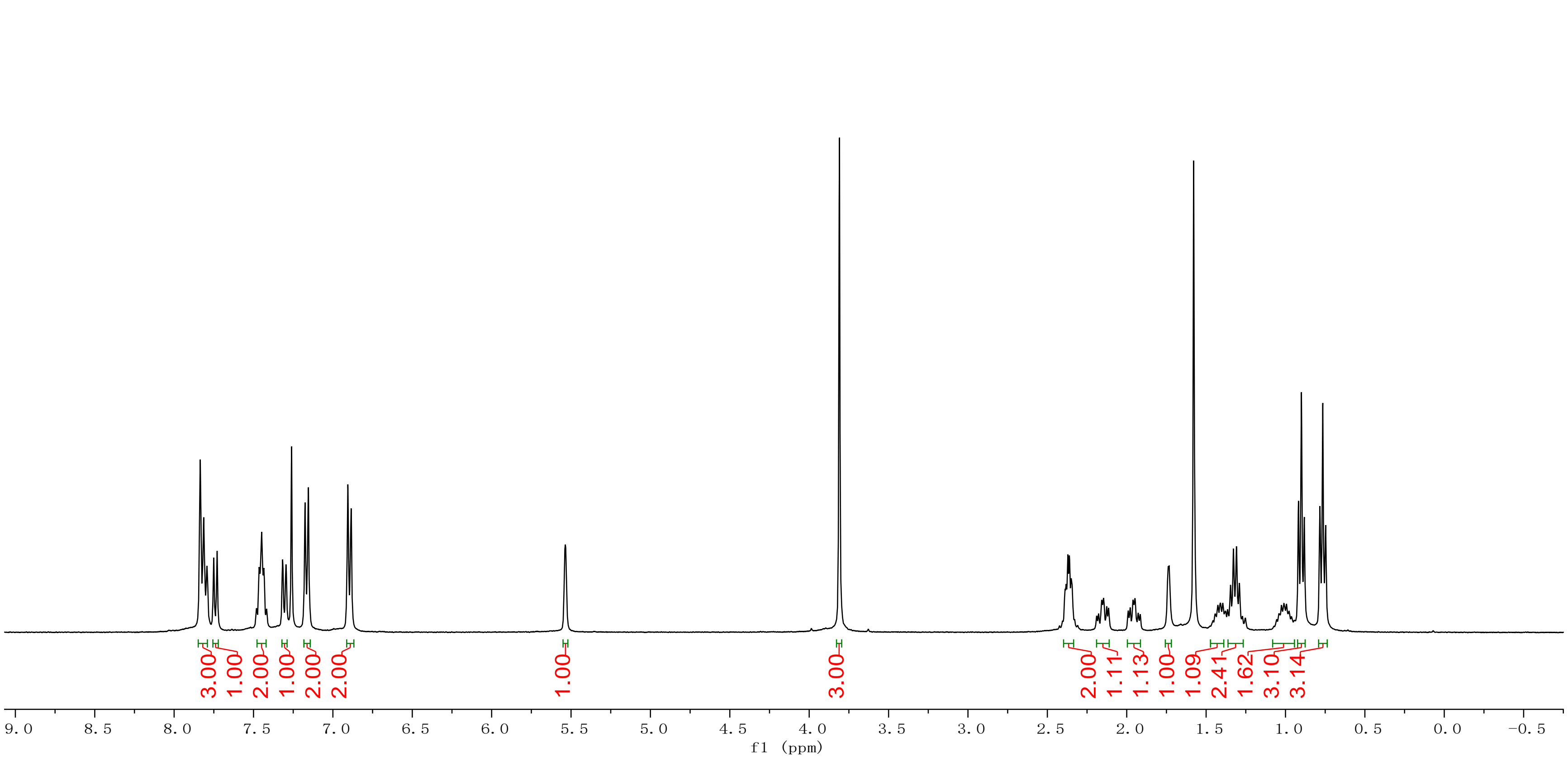

22：(*Z*)-3-(4-methoxyphenyl)-1-(naphthalen-2-yl)-2-propylhex-2-en-1-ol (^{13}C NMR, $CDCl_3$, 100 MHz)

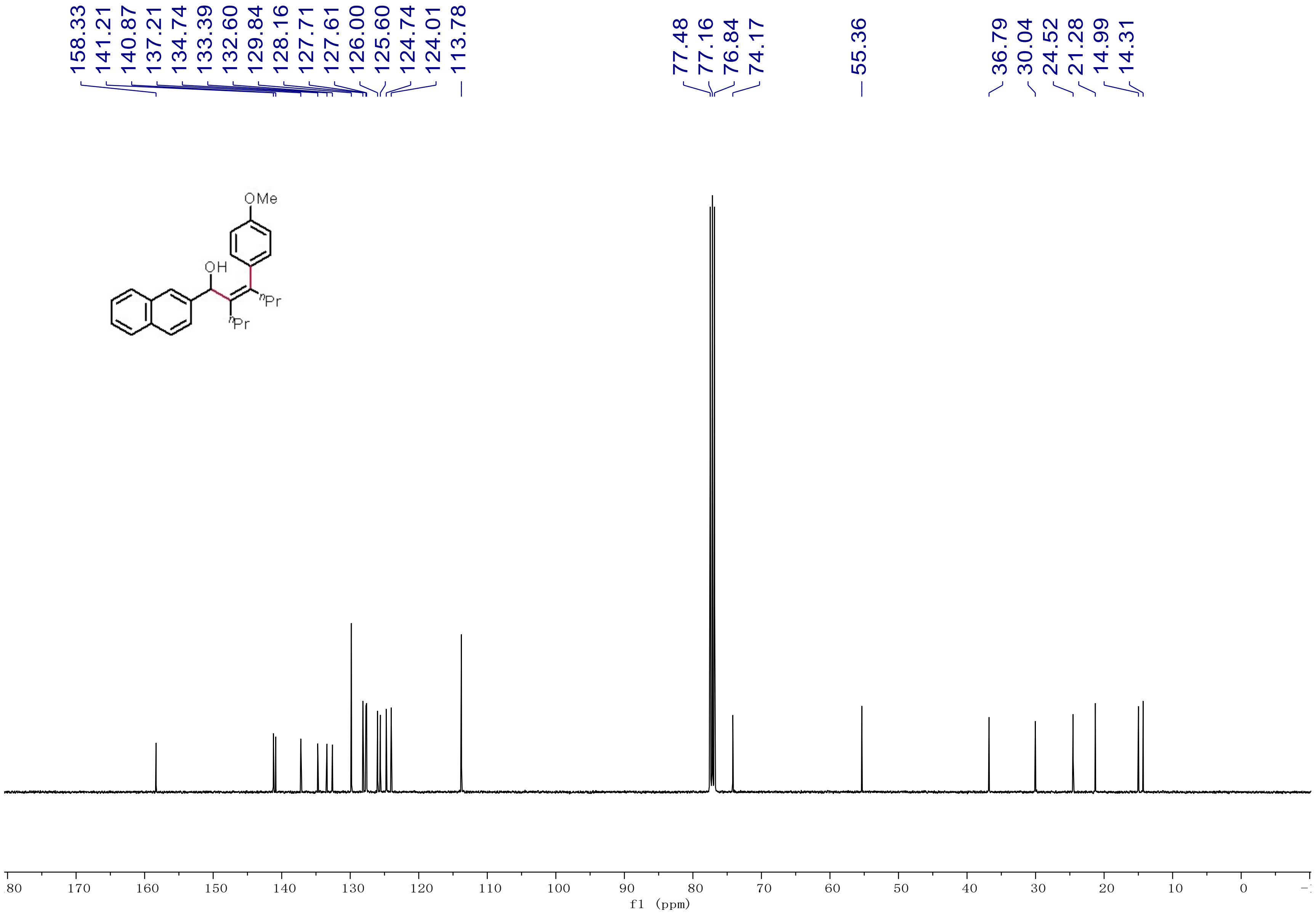

23: (*Z*)-1-(6-methoxynaphthalen-2-yl)-3-(4-methoxyphenyl)-2-propylhex-2-en-1-ol (^{1}H NMR, $CDCl_3$, 400 MHz)

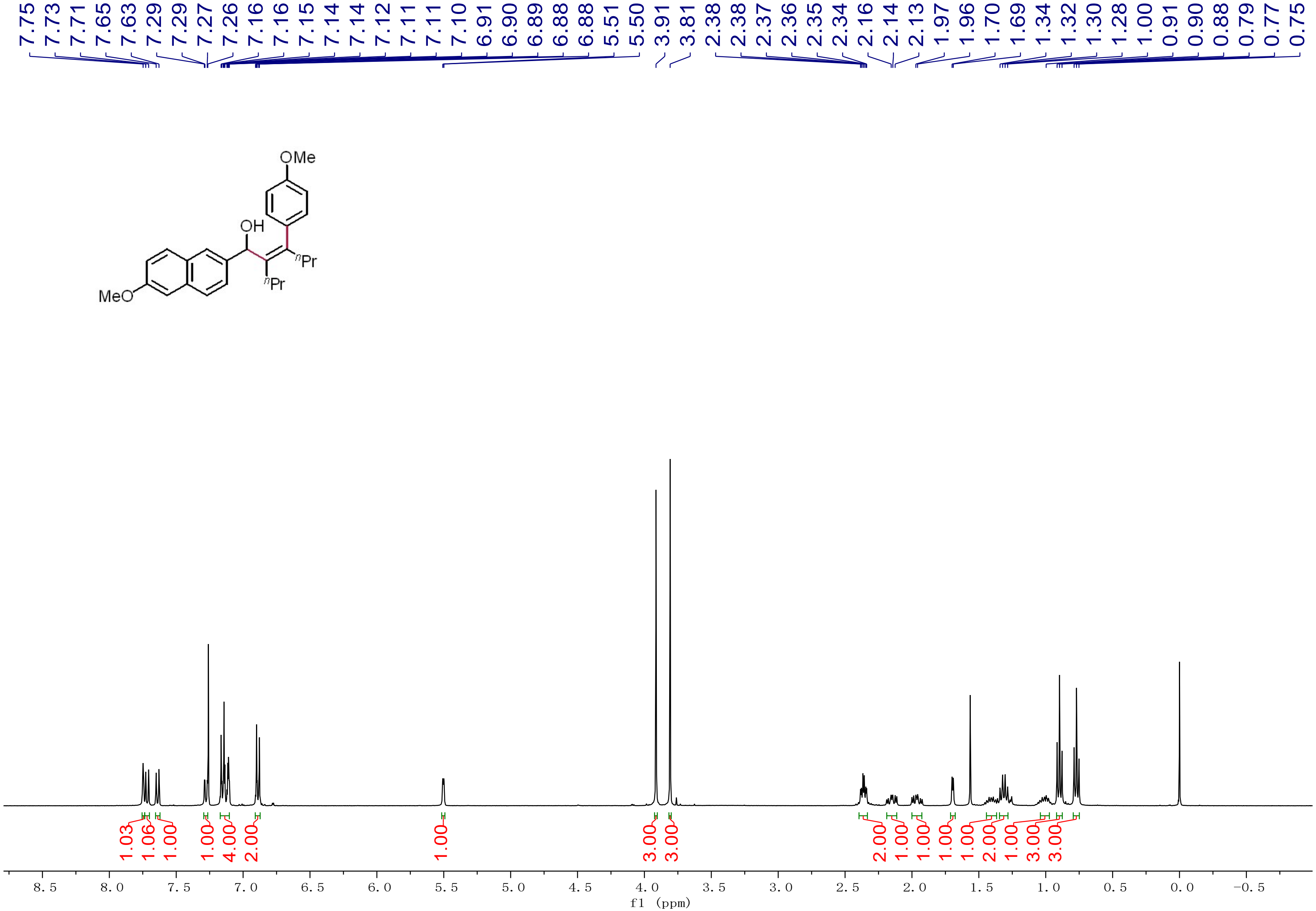

23: (*Z*)-1-(6-methoxynaphthalen-2-yl)-3-(4-methoxyphenyl)-2-propylhex-2-en-1-ol (^{13}C NMR, $CDCl_3$, 100 MHz)

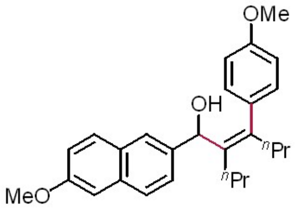

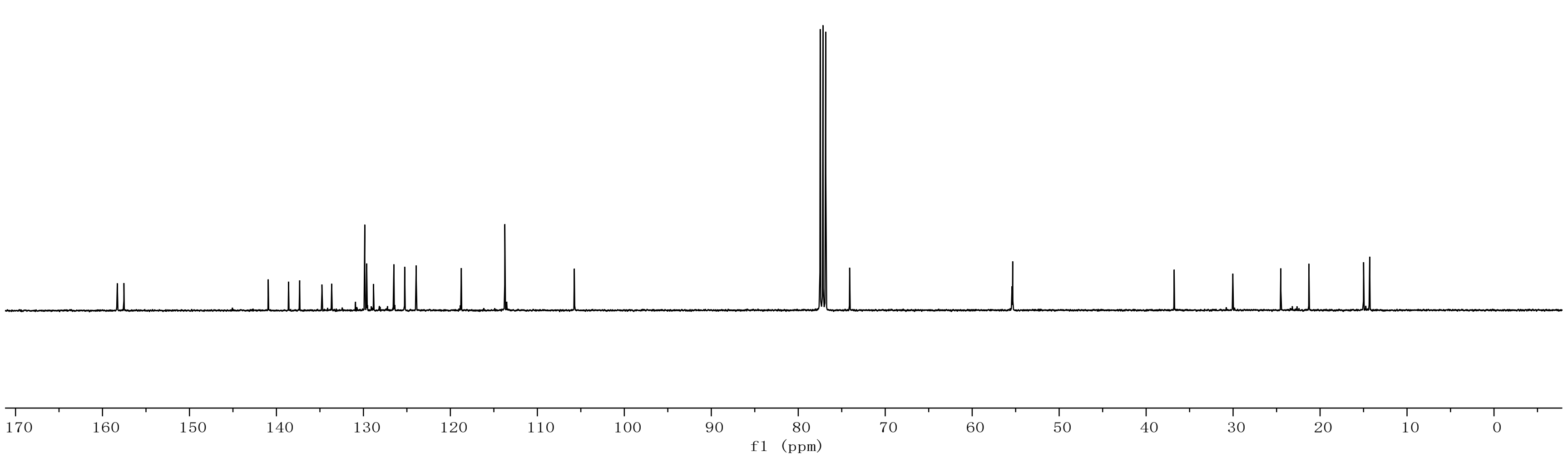

24: (*Z*)-3-(4-methoxyphenyl)-1-(naphthalen-1-yl)-2-propylhex-2-en-1-ol (^{1}H NMR, $CDCl_3$, 400 MHz)

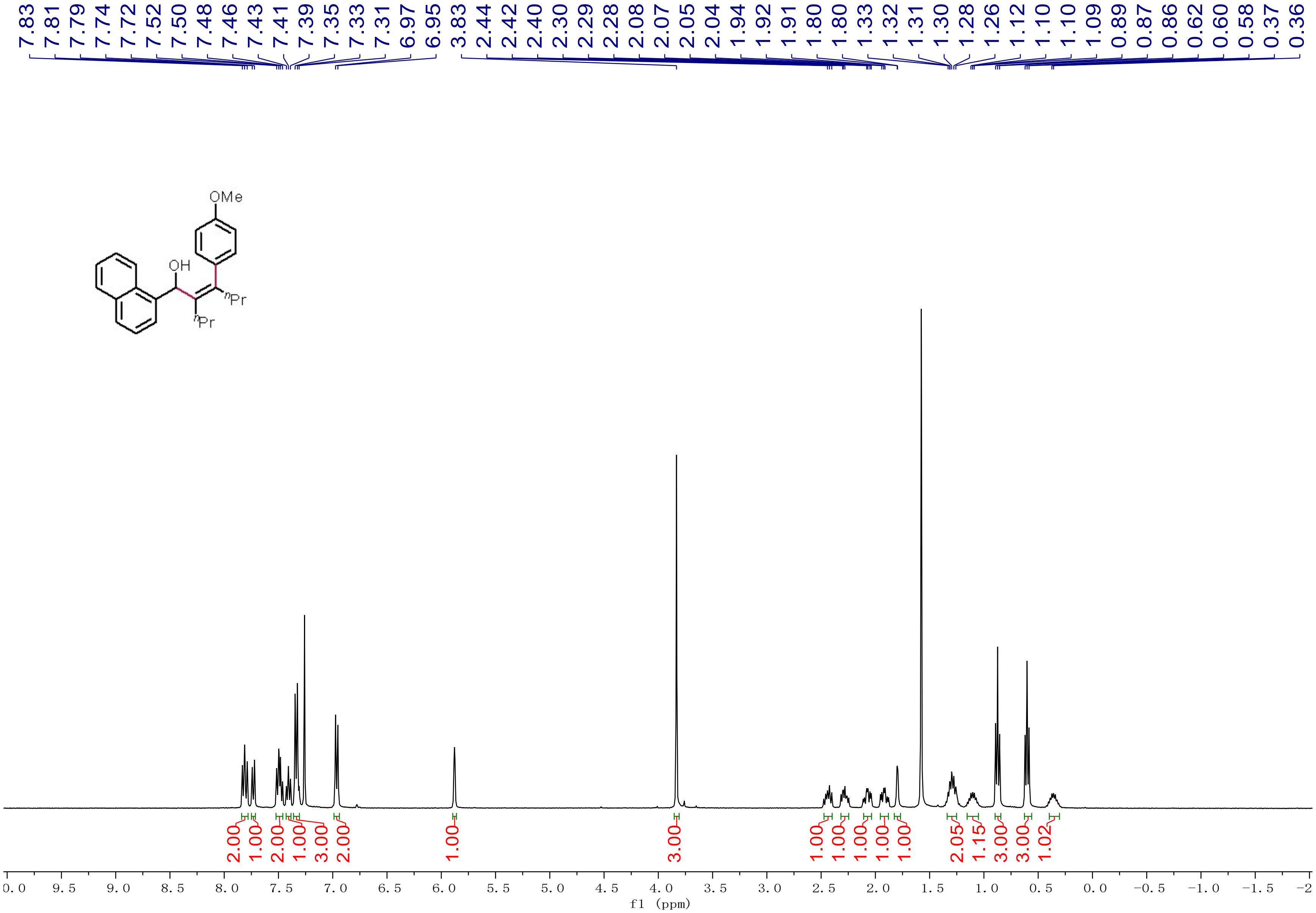

24：(*Z*)-3-(4-methoxyphenyl)-1-(naphthalen-1-yl)-2-propylhex-2-en-1-ol (^{13}C NMR, $CDCl_3$, 100 MHz)

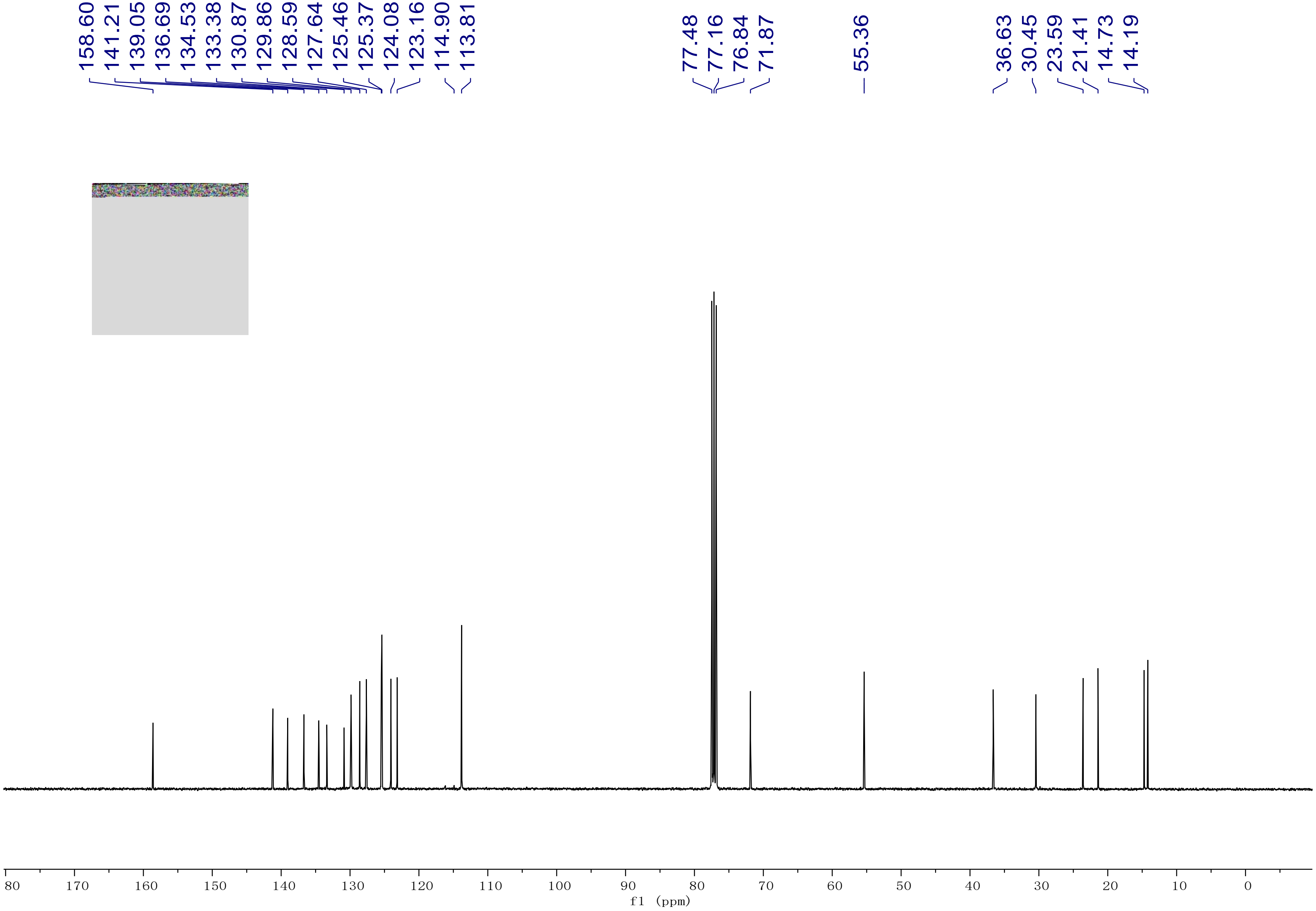

25: (*Z*)-2-butyl-1-(furan-3-yl)-3-(4-methoxyphenyl)hept-2-en-1-ol (^{1}H NMR, $CDCl_3$, 400 MHz)

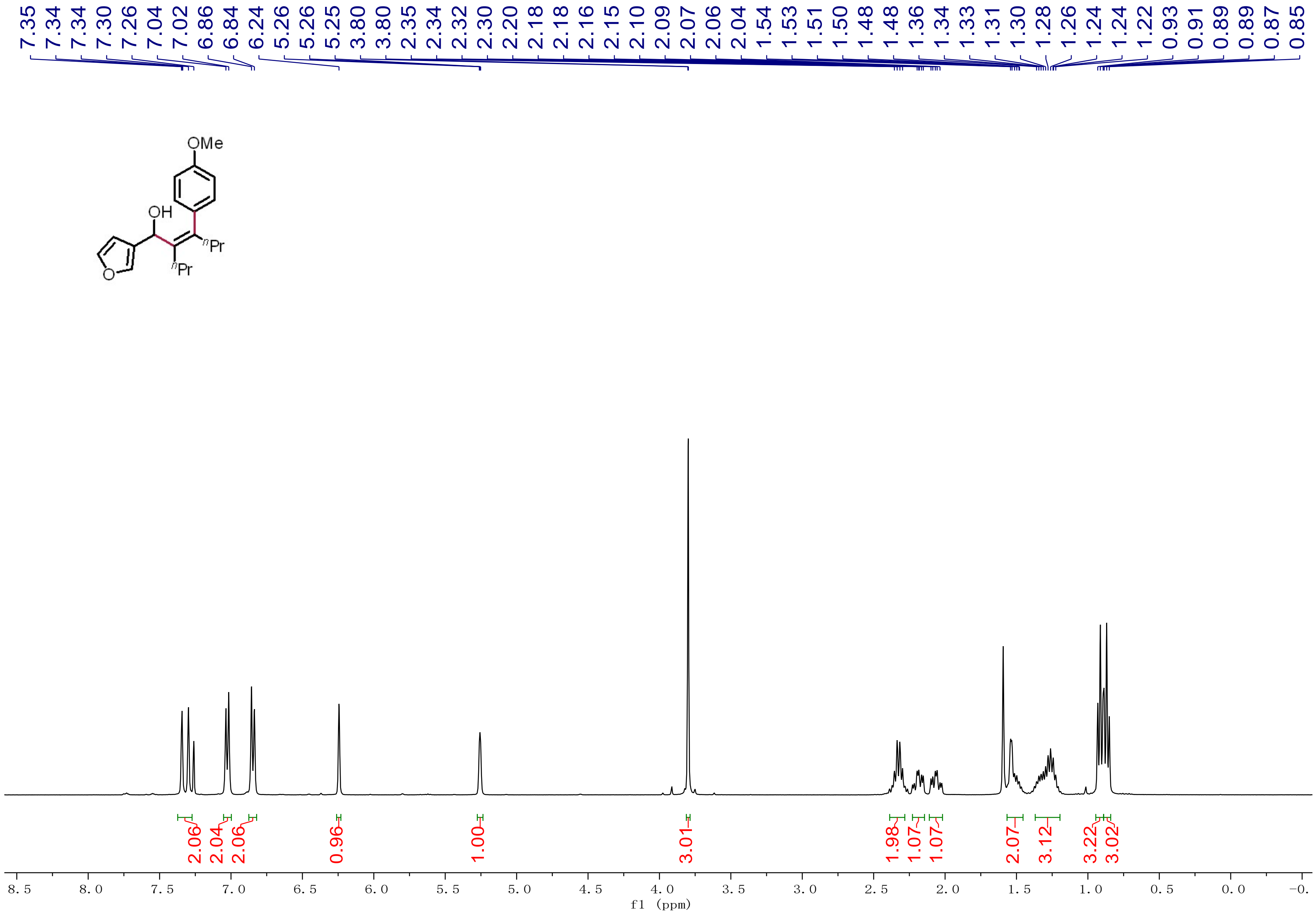

25: (*Z*)-2-butyl-1-(furan-3-yl)-3-(4-methoxyphenyl)hept-2-en-1-ol (^{13}C NMR, $CDCl_3$, 100 MHz)

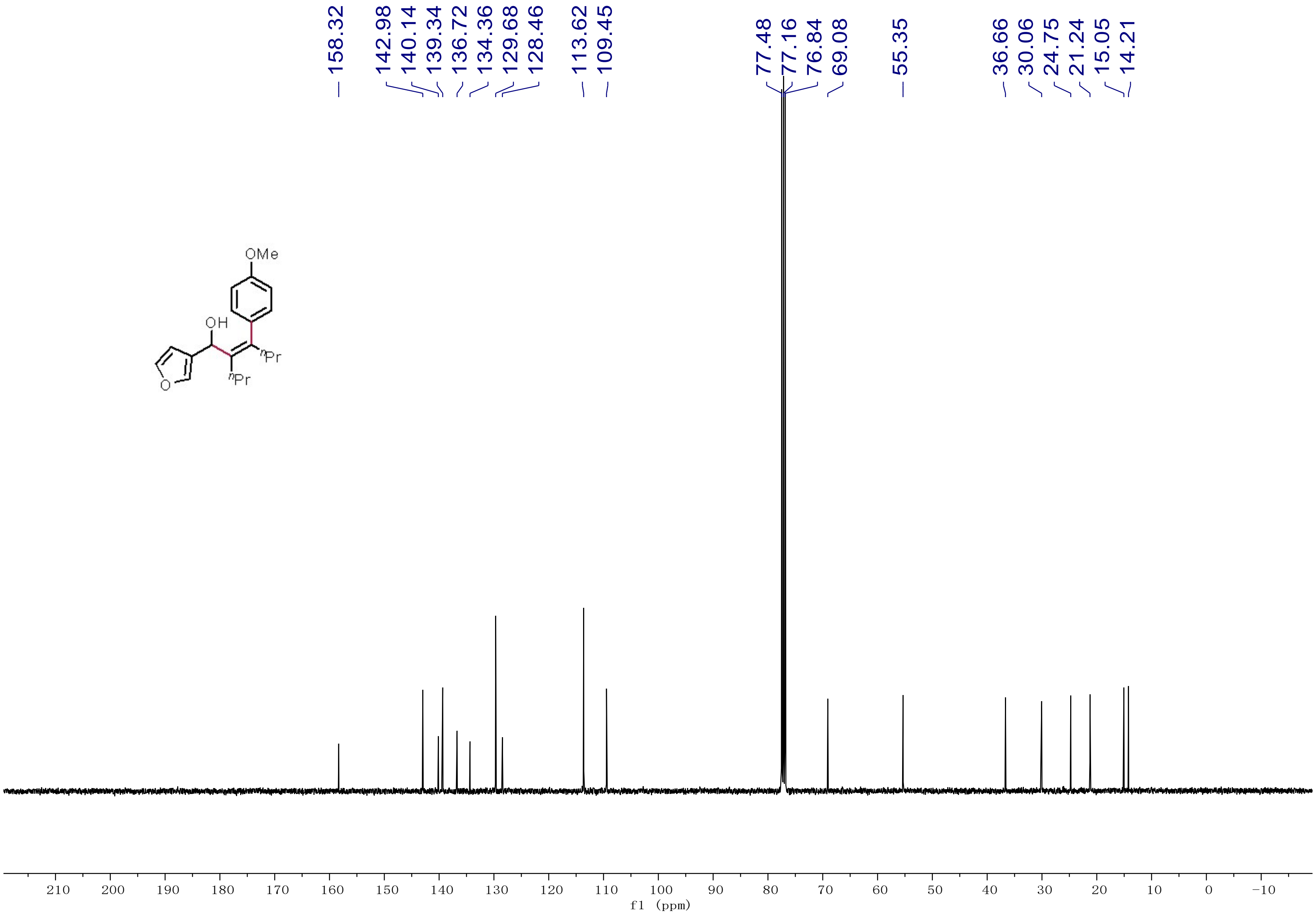

26: (Z)-2-butyl-3-(4-methoxyphenyl)-1-(thiophen-2-yl)hept-2-en-1-ol (^{1}H NMR, $CDCl_3$, 400 MHz)

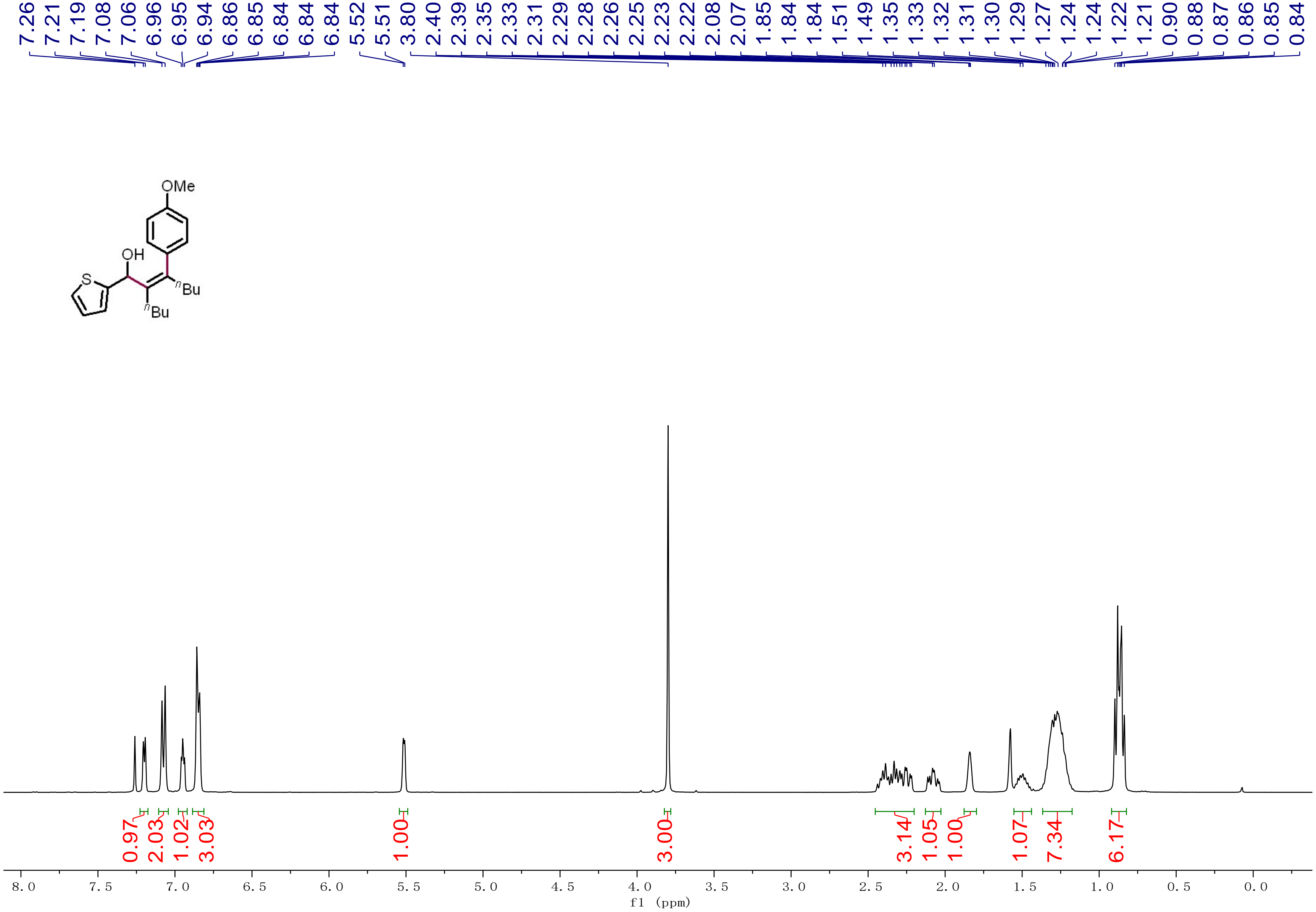

26: (Z)-2-butyl-3-(4-methoxyphenyl)-1-(thiophen-2-yl)hept-2-en-1-ol (^{13}C NMR, $CDCl_3$, 100 MHz)

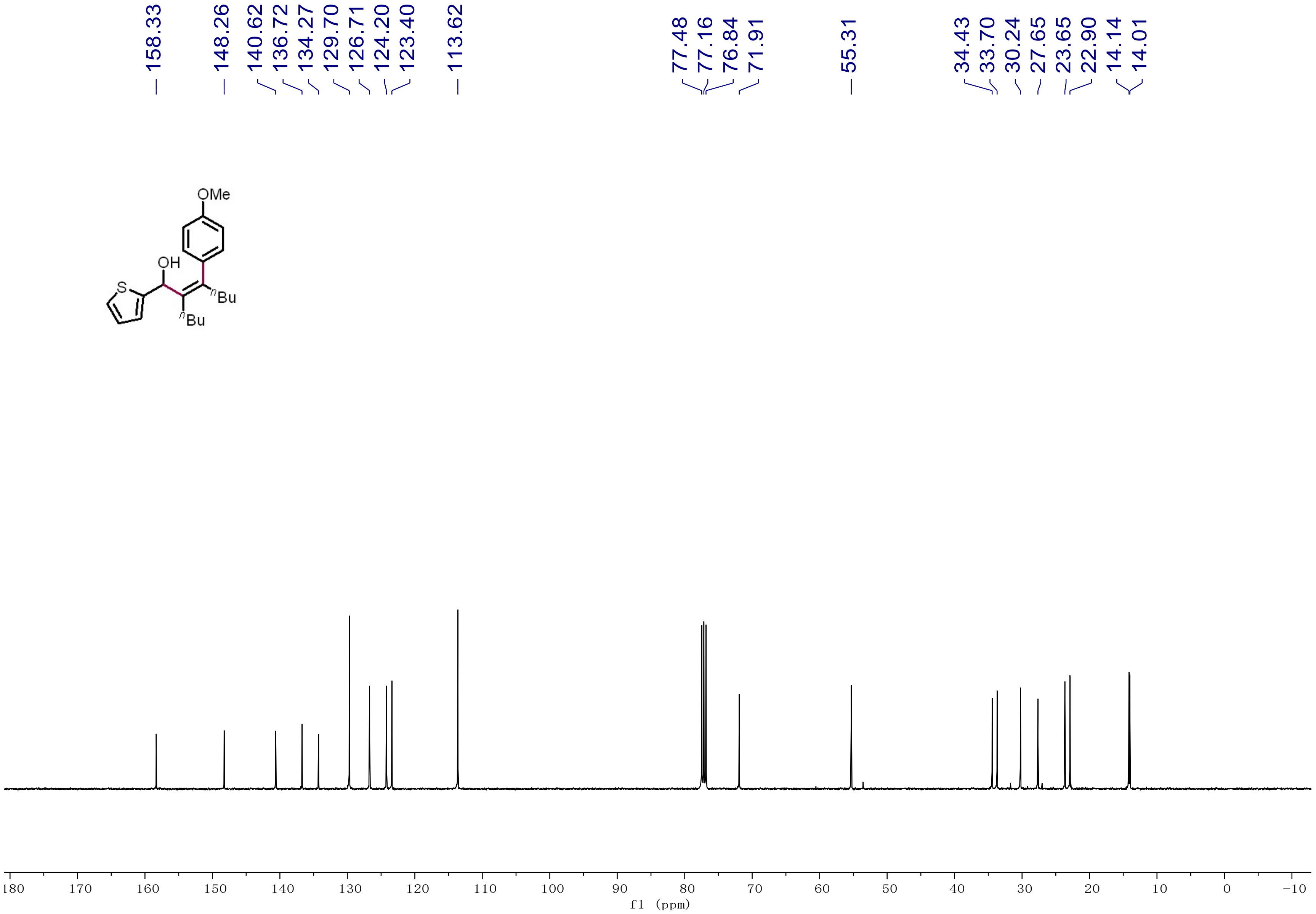

27: (*Z*)-1-(benzofuran-2-yl)-2-butyl-3-(4-methoxyphenyl)hept-2-en-1-ol (^{1}H NMR, $CDCl_3$, 400 MHz)

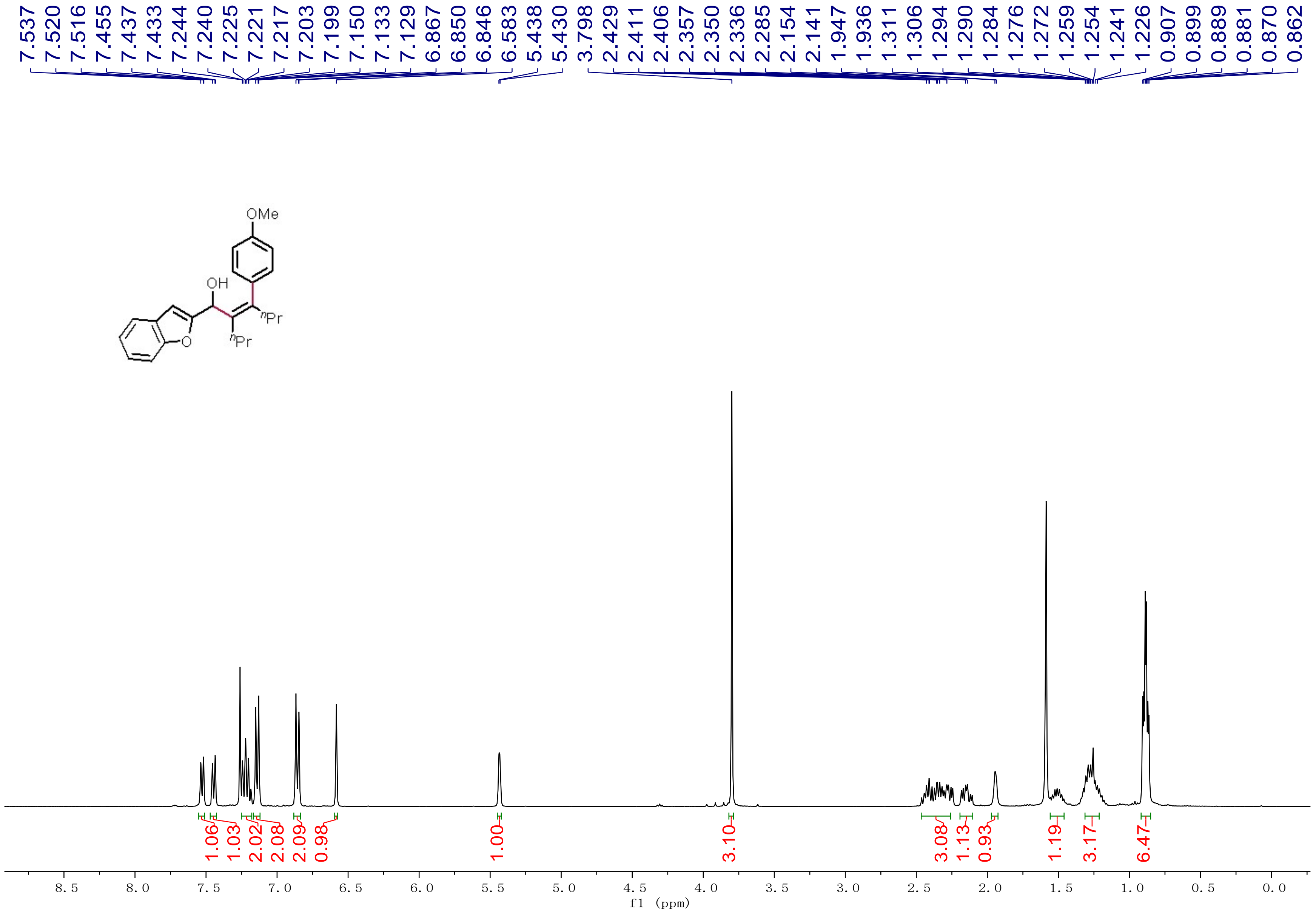

27： (*Z*)-1-(benzofuran-2-yl)-2-butyl-3-(4-methoxyphenyl)hept-2-en-1-ol (^{13}C NMR, $CDCl_3$, 100 MHz)

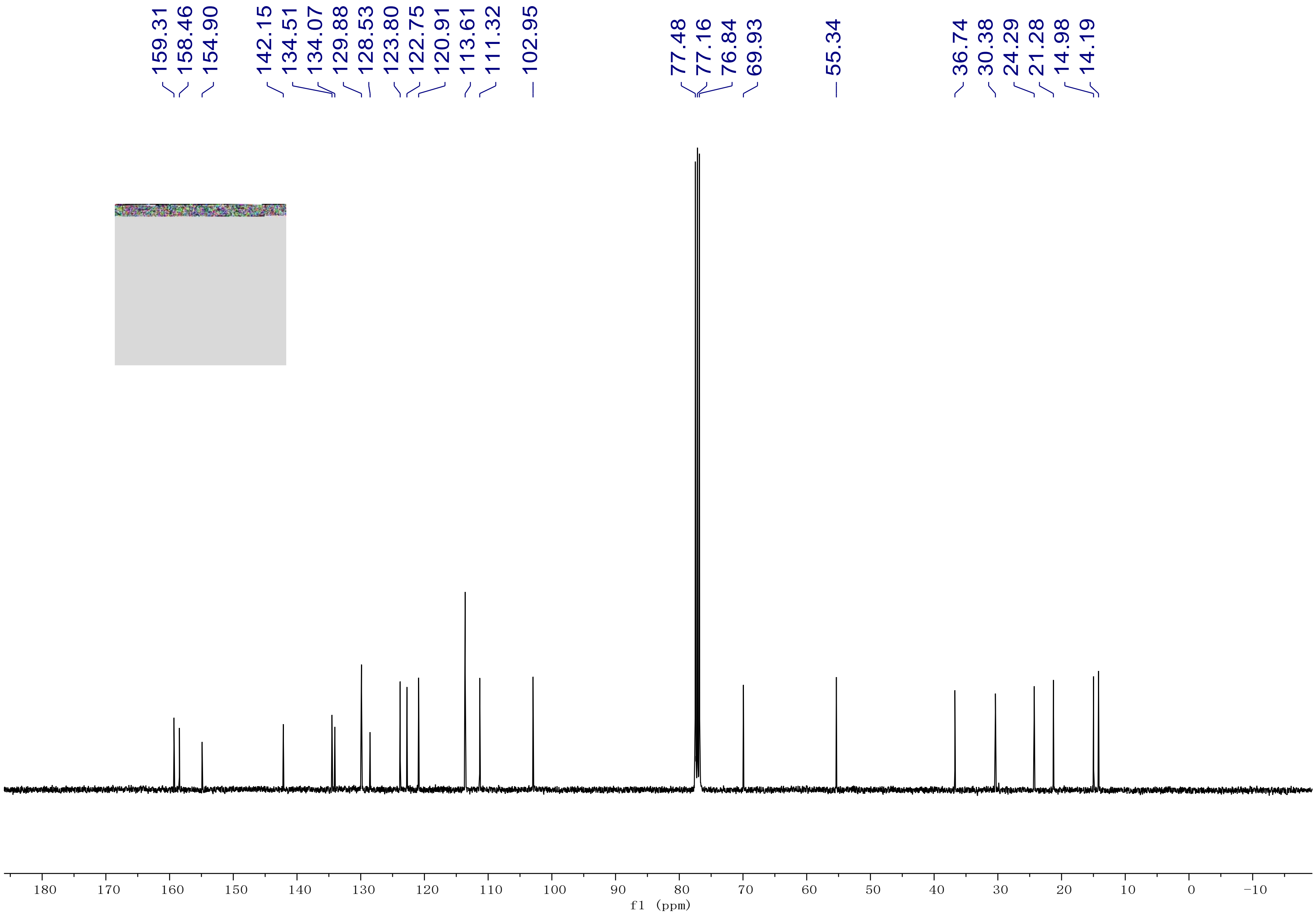

28: (*Z*)-1-(benzo[b]thiophen-2-yl)-2-butyl-3-(4-methoxyphenyl)hept-2-en-1-ol (^{1}H NMR, $CDCl_3$, 400 MHz)

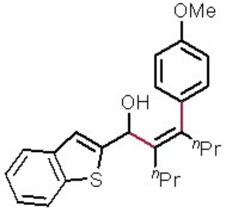

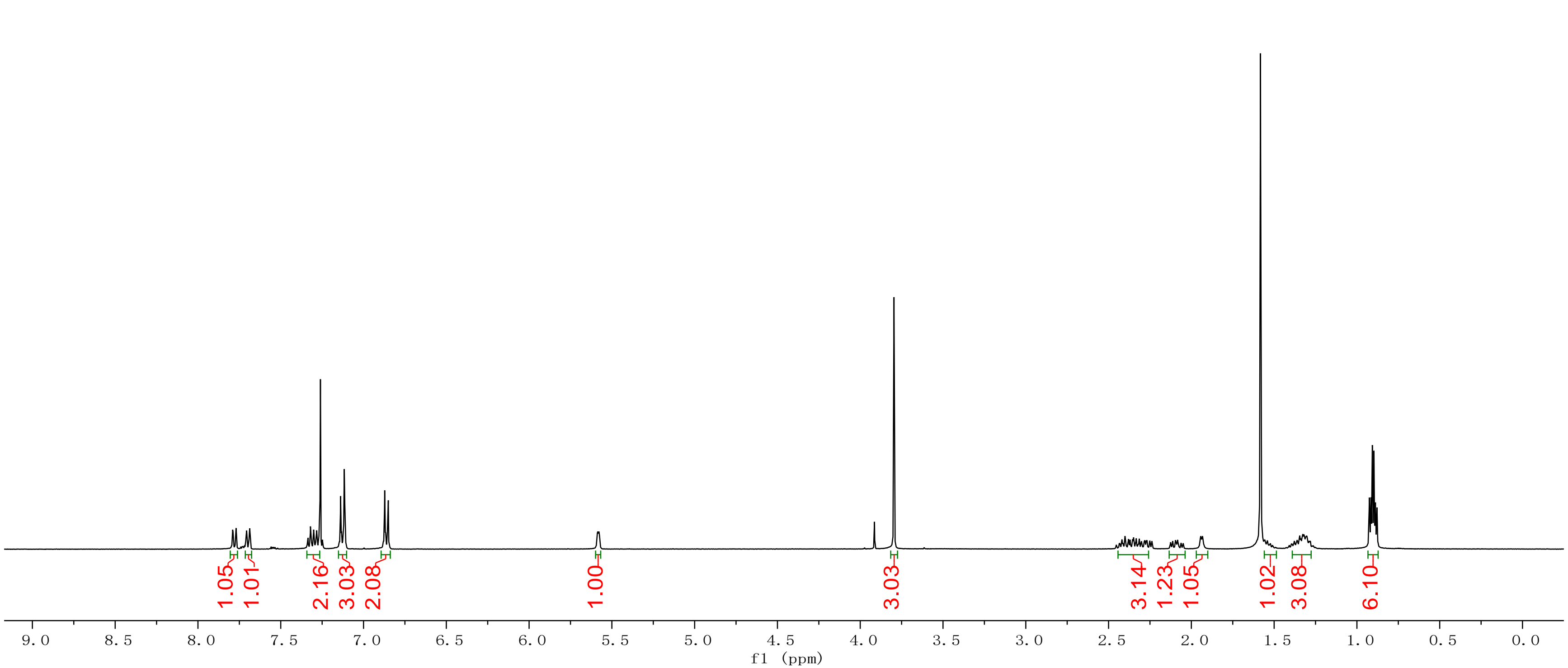

28： (*Z*)-1-(benzo[b]thiophen-2-yl)-2-butyl-3-(4-methoxyphenyl)hept-2-en-1-ol (^{13}C NMR, $CDCl_3$, 100 MHz)

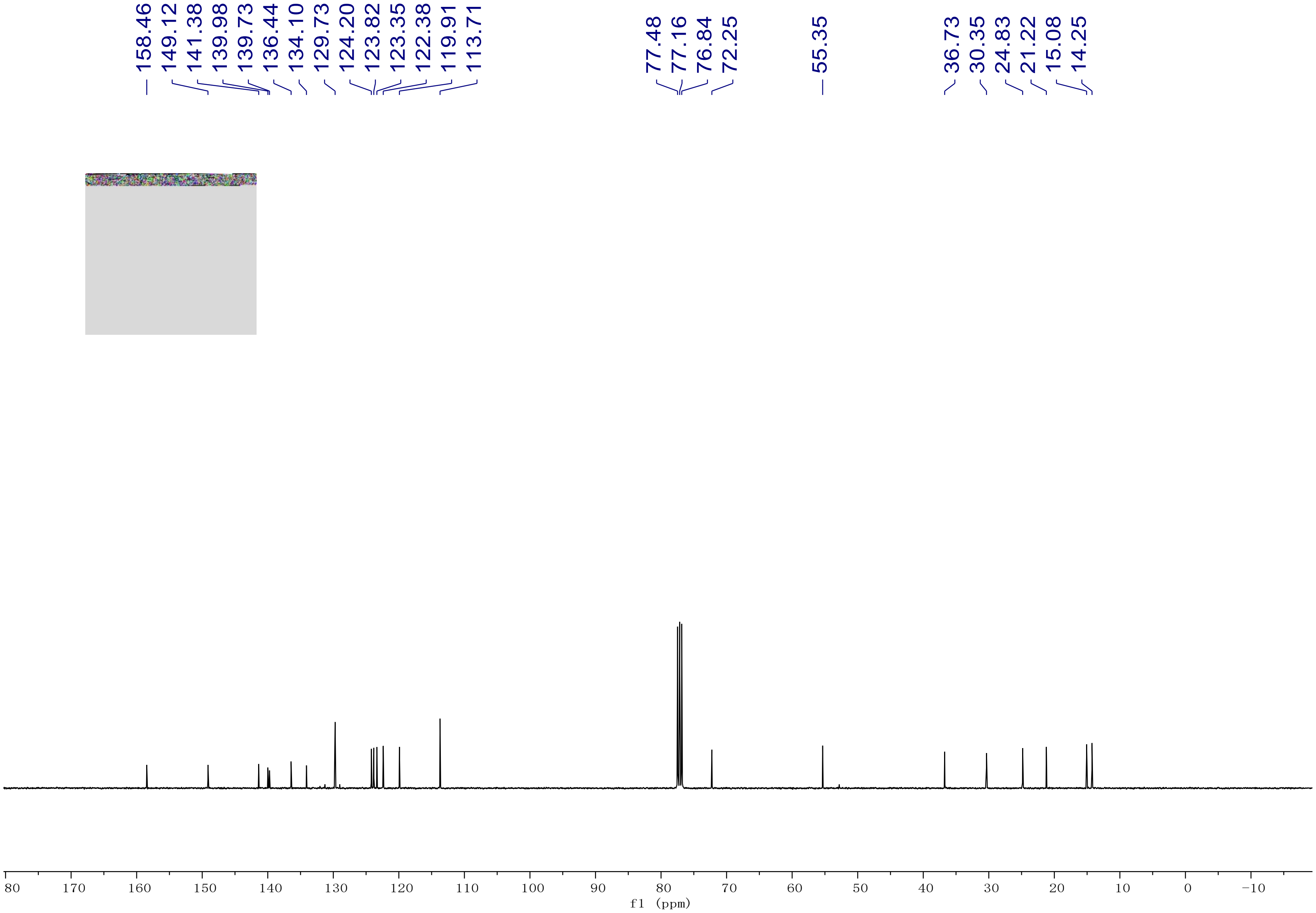

29: (*Z*)-4-butyl-5-(4-methoxyphenyl)-1-phenylnon-4-en-3-ol (^{1}H NMR, $CDCl_3$, 400 MHz)

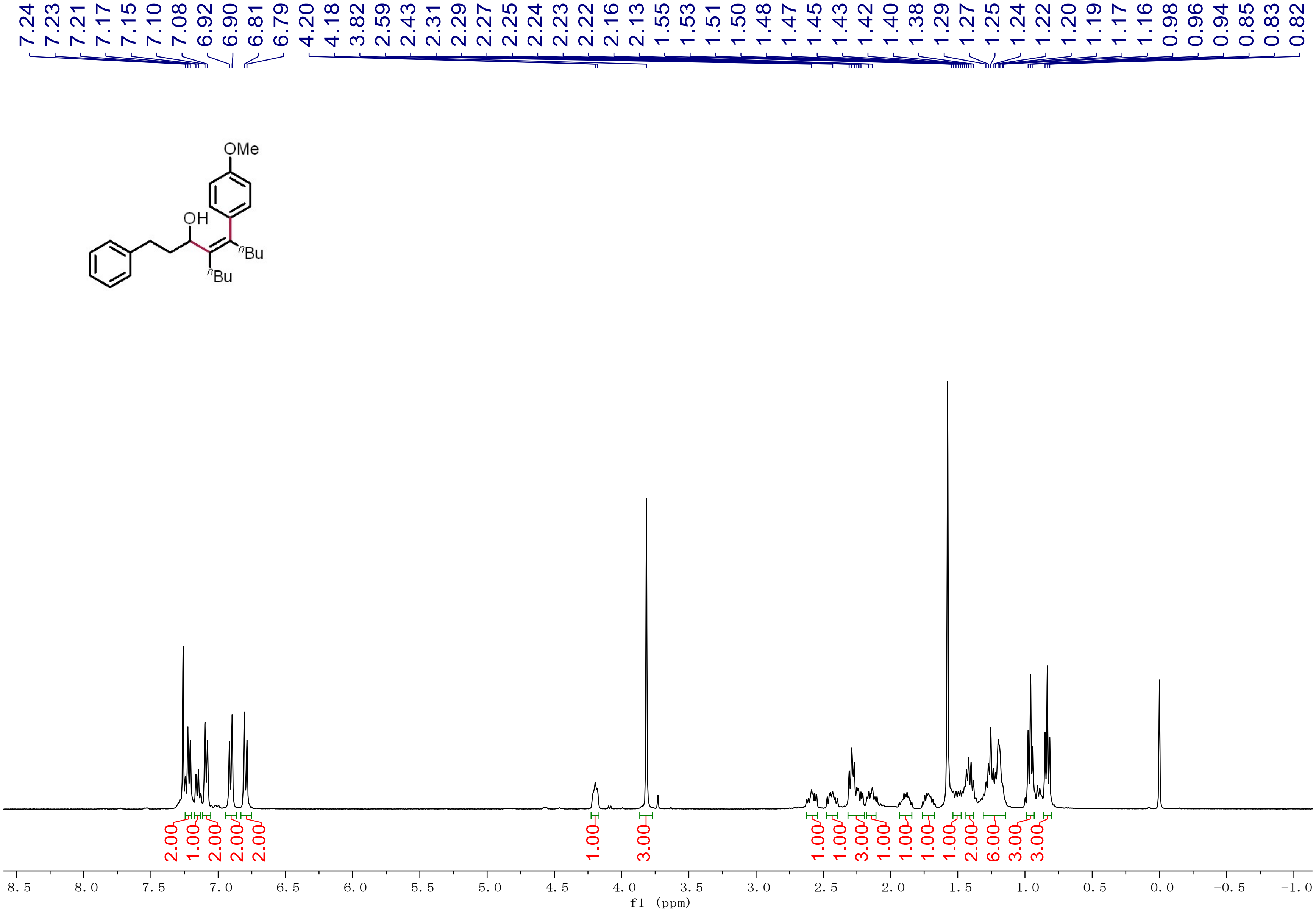

29: (*Z*)-4-butyl-5-(4-methoxyphenyl)-1-phenylnon-4-en-3-ol (^{13}C NMR, $CDCl_3$, 100 MHz)

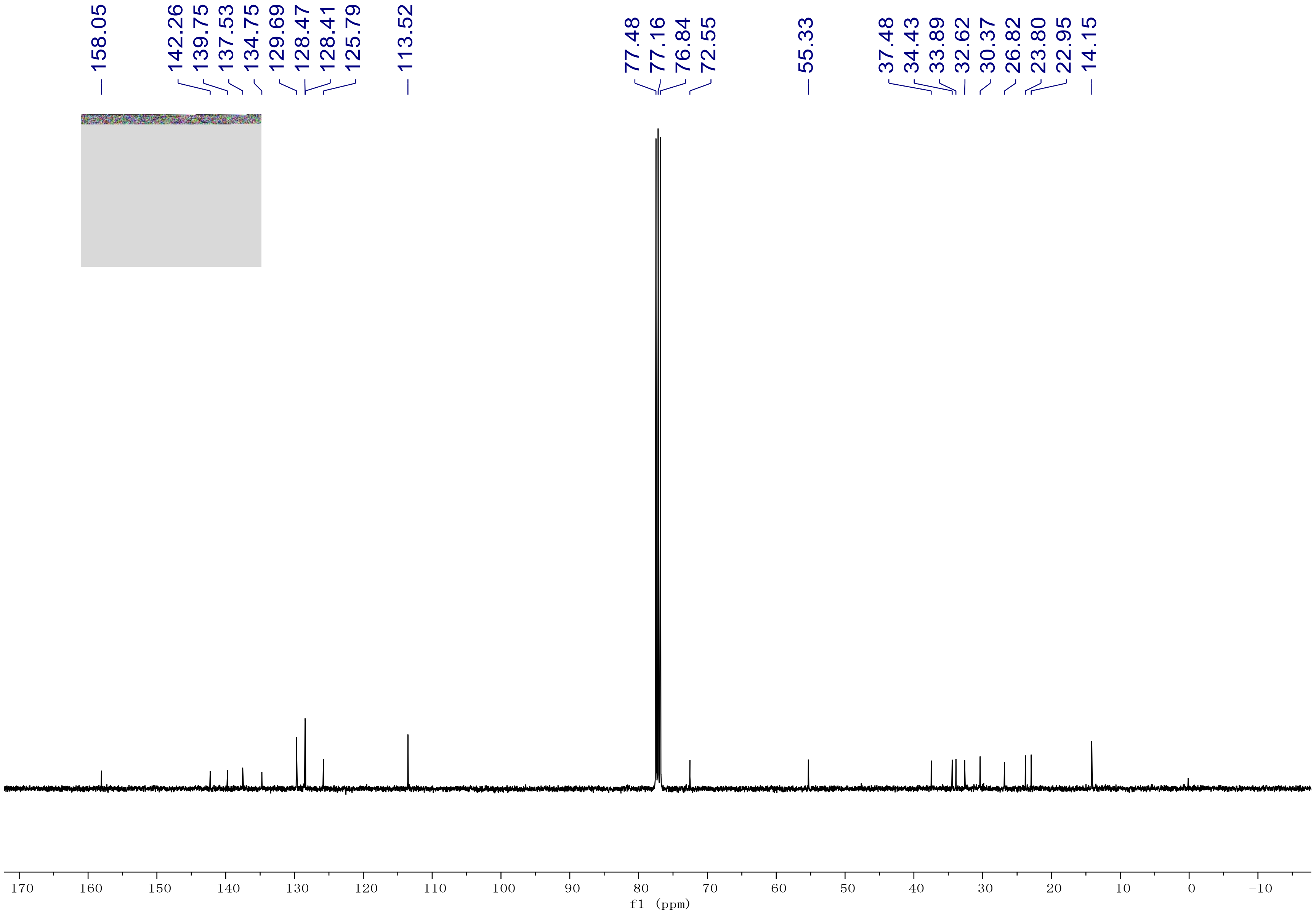

30: (Z)-3-(4-methoxyphenyl)-2-methyl-1-phenylbut-2-en-1-ol (^1H NMR, $CDCl_3$, 400 MHz)

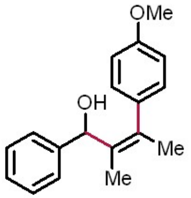

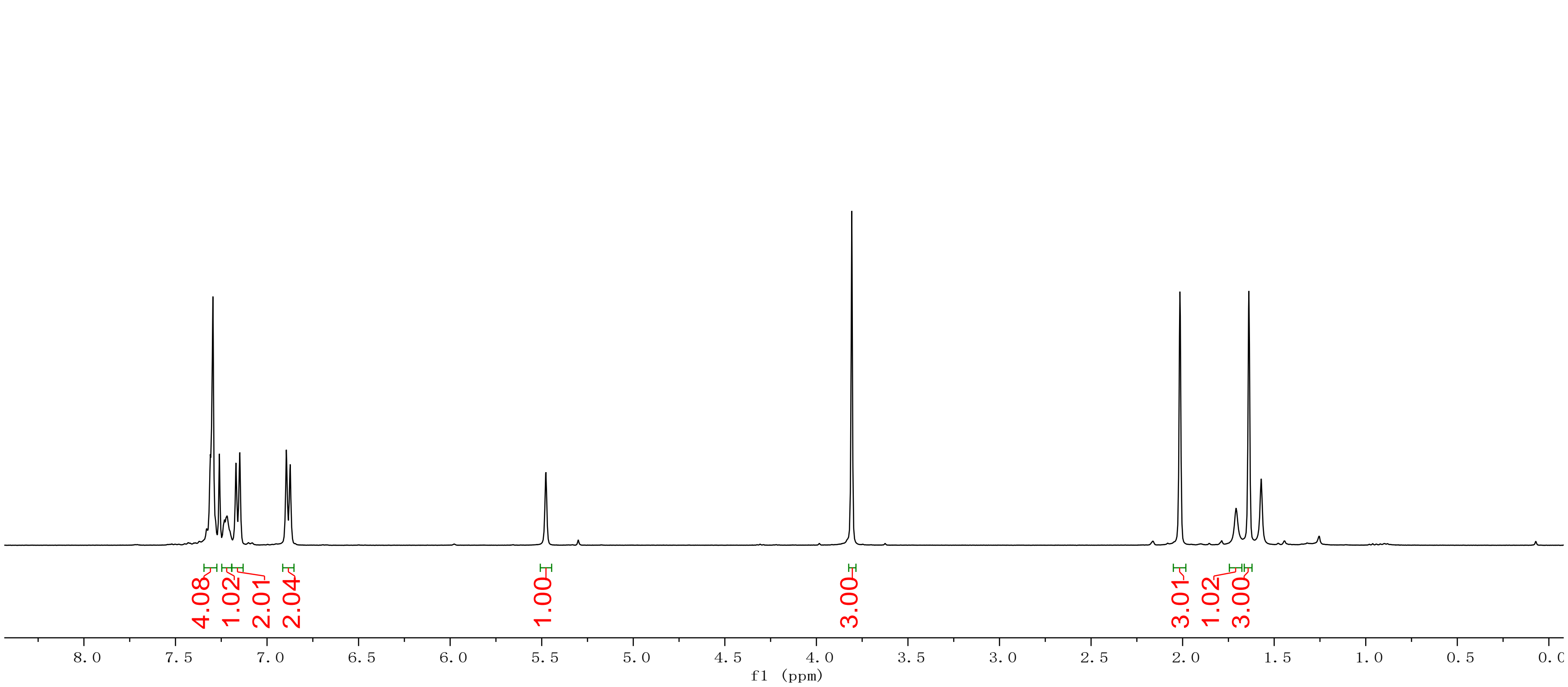

30: (Z)-3-(4-methoxyphenyl)-2-methyl-1-phenylbut-2-en-1-ol (^{13}C NMR, $CDCl_3$, 100 MHz)

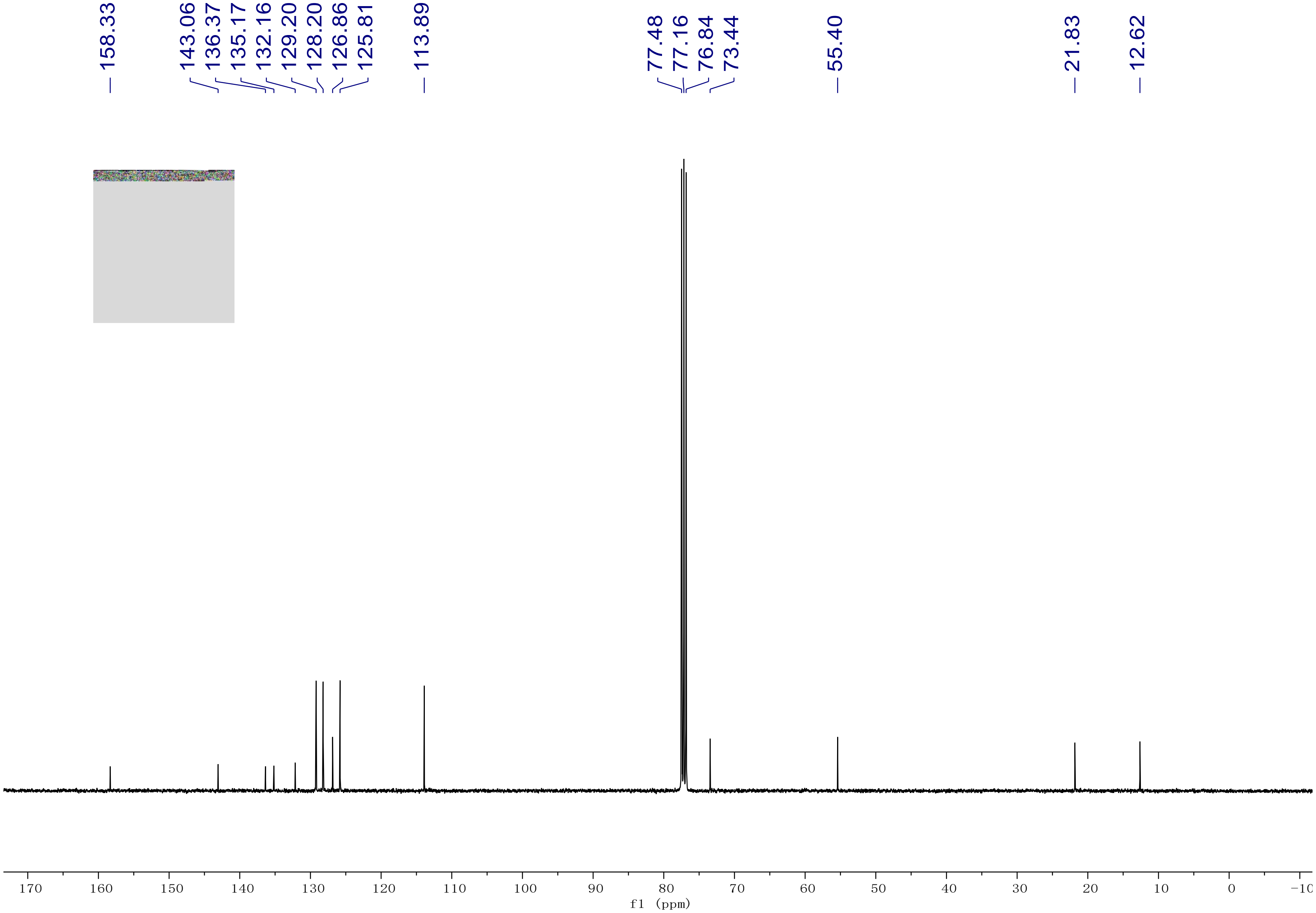

31: (Z)-2-ethyl-3-(4-methoxyphenyl)-1-phenylpent-2-en-1-ol (^{1}H NMR, $CDCl_3$, 400 MHz)

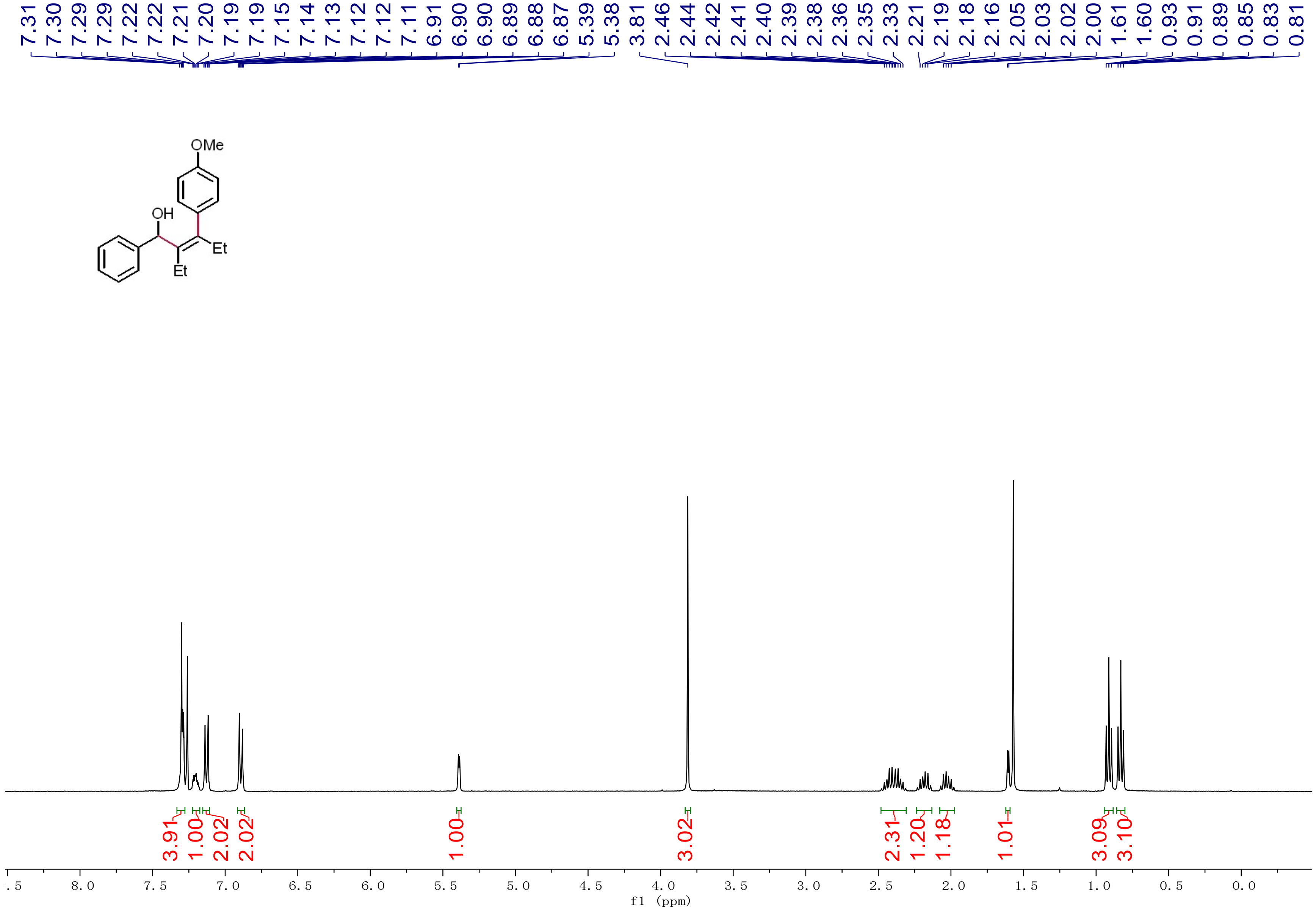

31: (Z)-2-ethyl-3-(4-methoxyphenyl)-1-phenylpent-2-en-1-ol (^{13}C NMR, $CDCl_3$, 100 MHz)

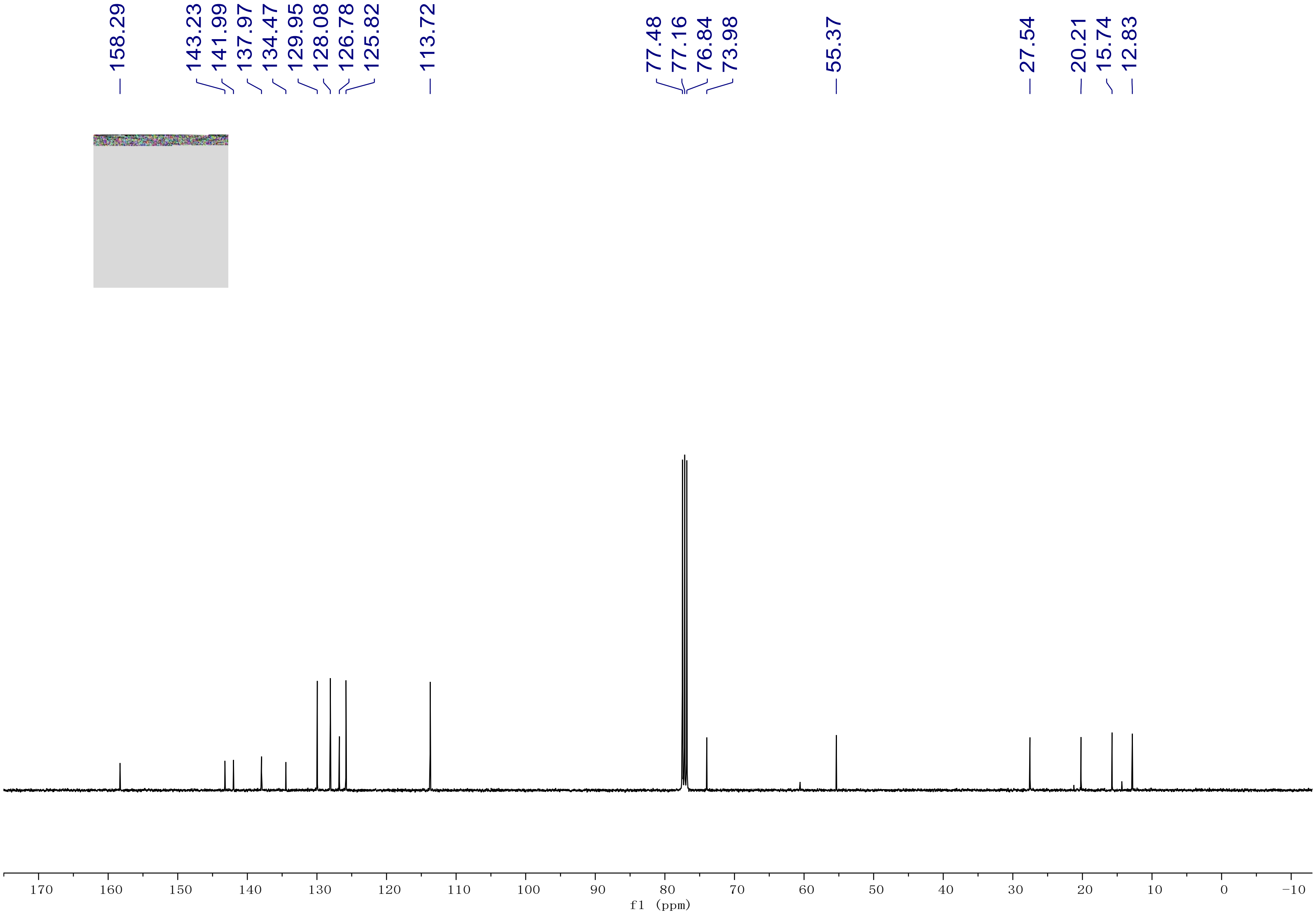

32: (Z)-2-butyl-3-(4-methoxyphenyl)-1-phenylhept-2-en-1-ol (^{1}H NMR, $CDCl_3$, 400 MHz)

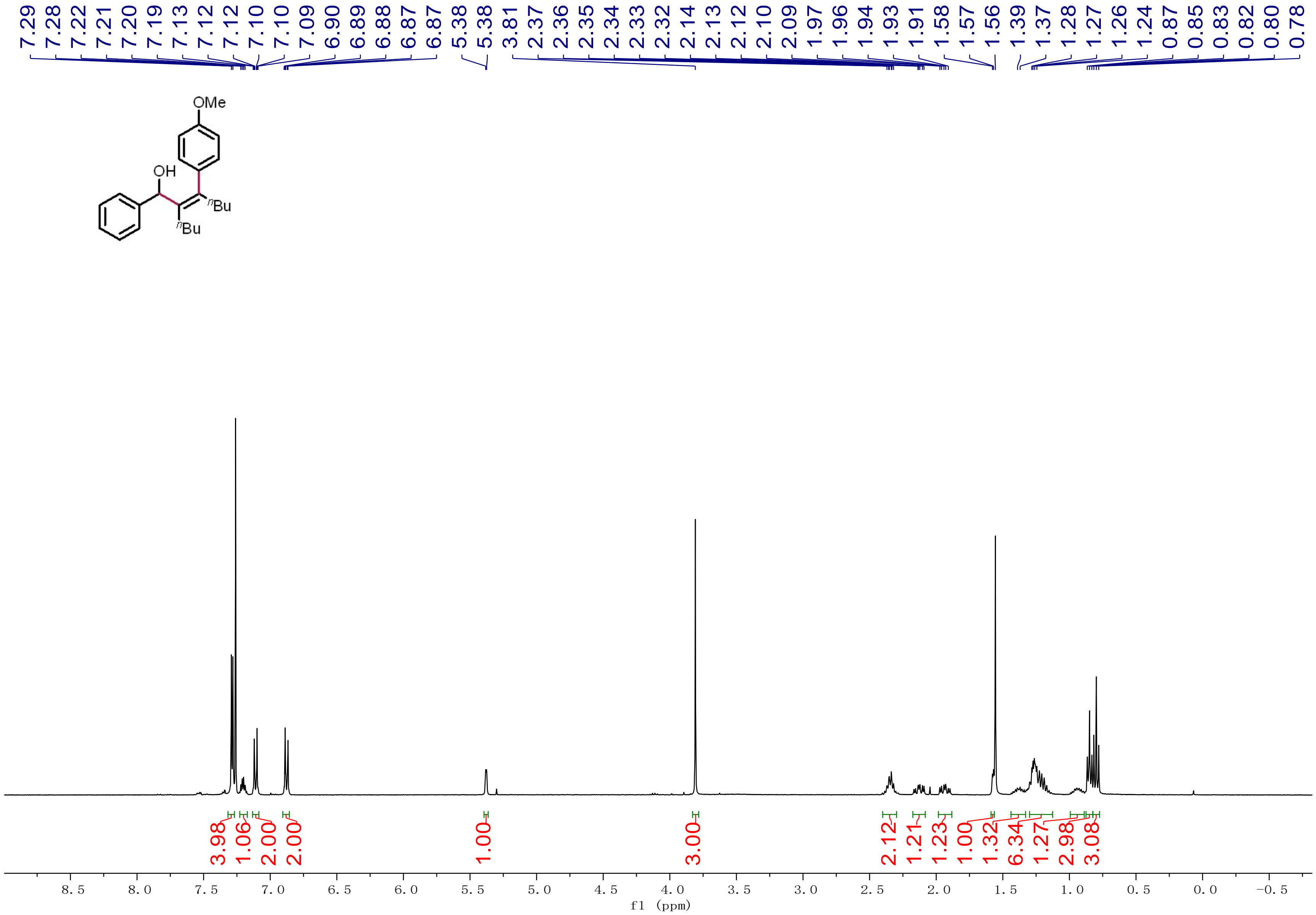

32: (Z)-2-butyl-3-(4-methoxyphenyl)-1-phenylhept-2-en-1-ol (^{13}C NMR, $CDCl_3$, 100 MHz)

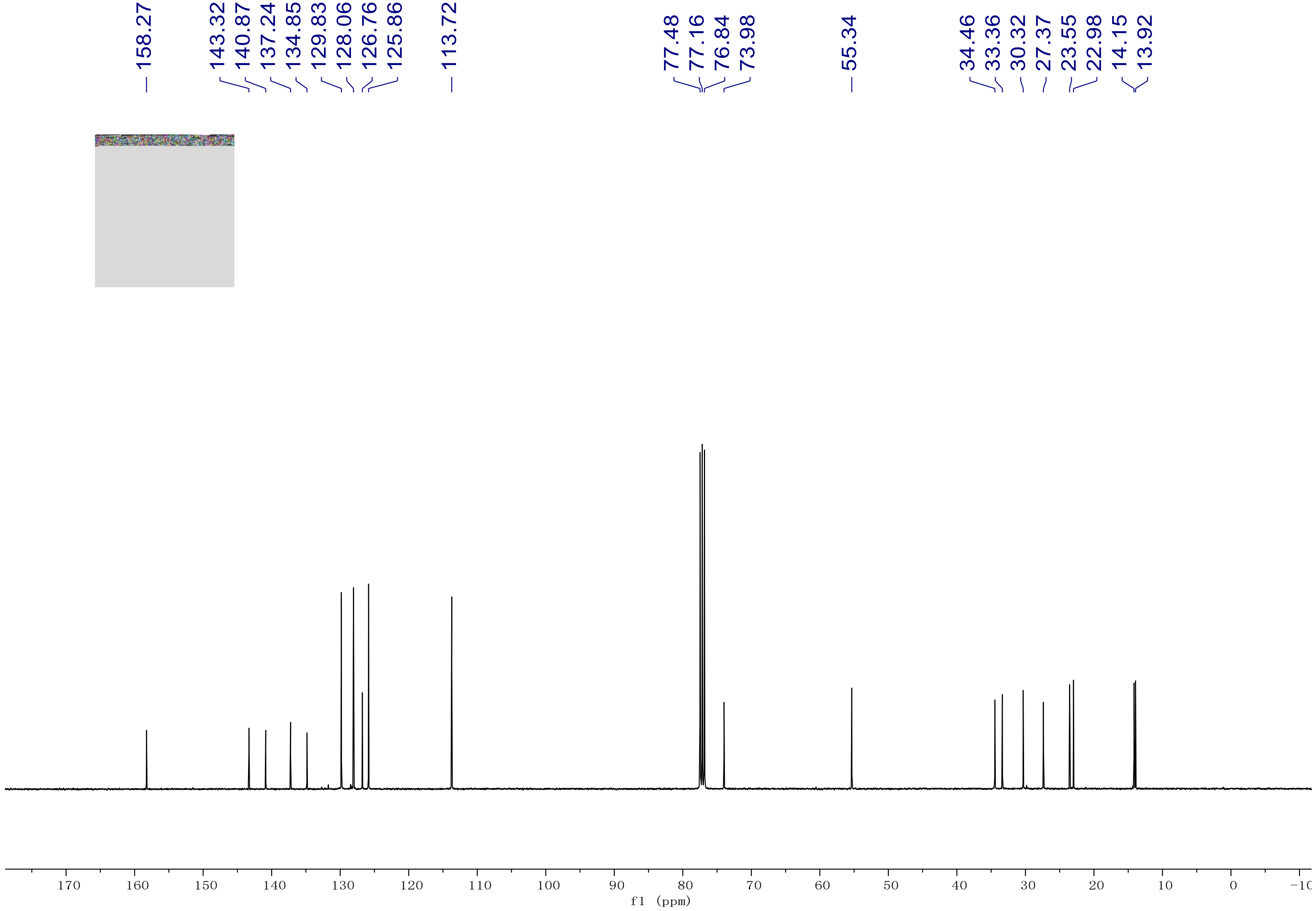

33: (Z)-2-pent-3-(4-methoxyphenyl)-1-phenylhept-2-en-1-ol (^{1}H NMR, $CDCl_3$, 400 MHz)

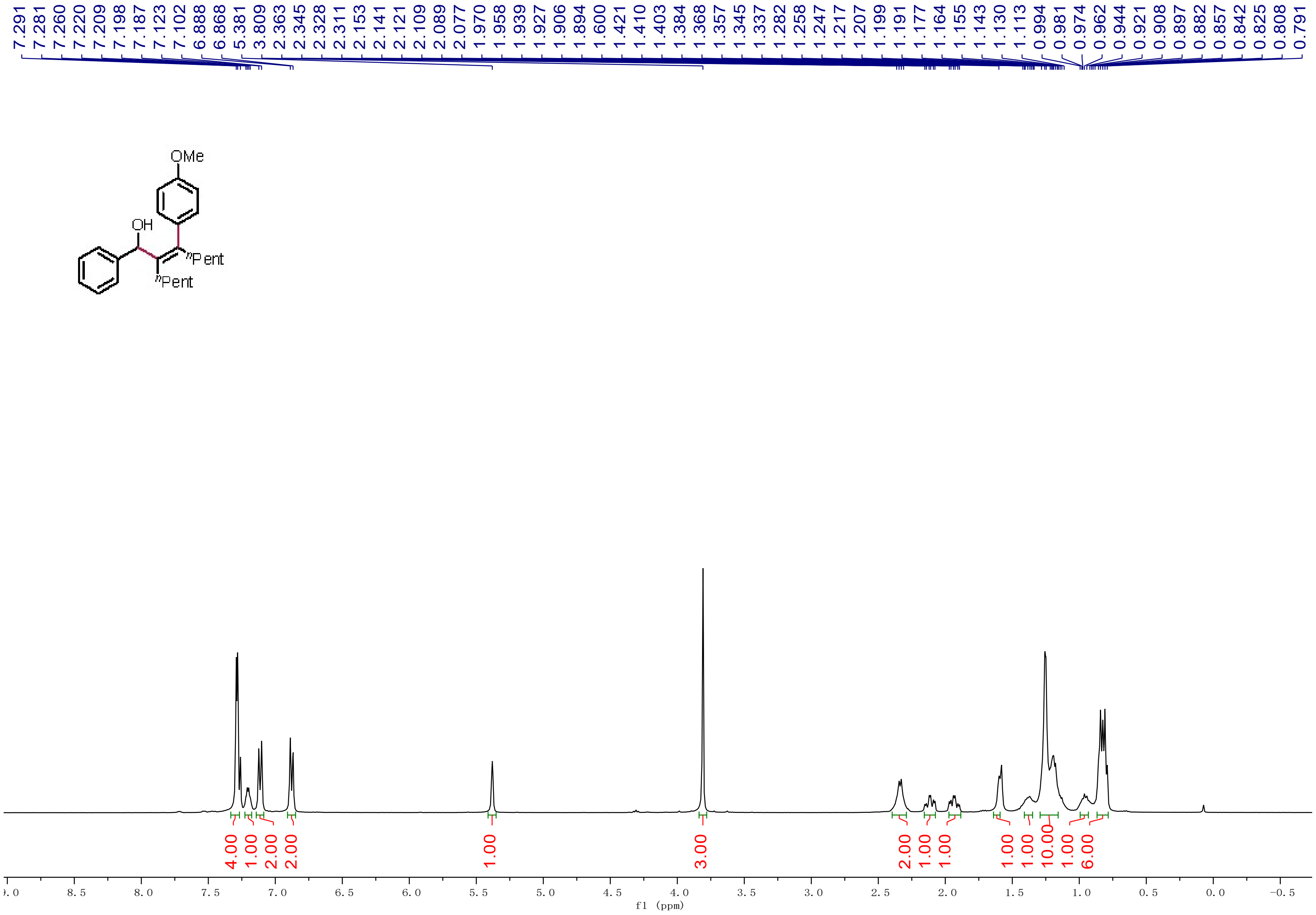

33: (Z)-2-pent-3-(4-methoxyphenyl)-1-phenylhept-2-en-1-ol (^{13}C NMR, $CDCl_3$, 100 MHz)

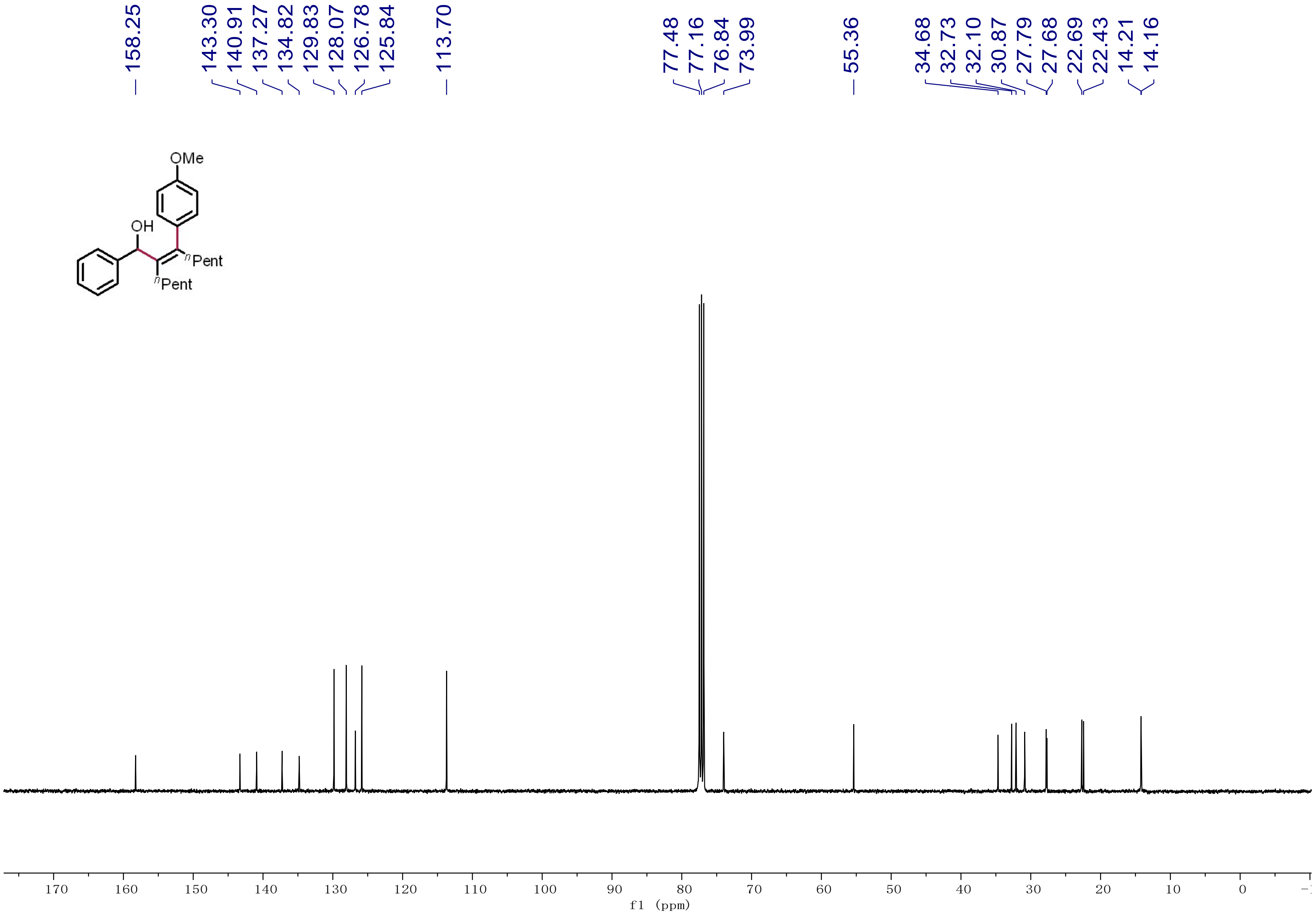

34: (Z)-3-(4-methoxyphenyl)-1,6-diphenyl-2-(3-phenylpropyl)hex-2-en-1-ol (^{1}H NMR, $CDCl_3$, 400 MHz)

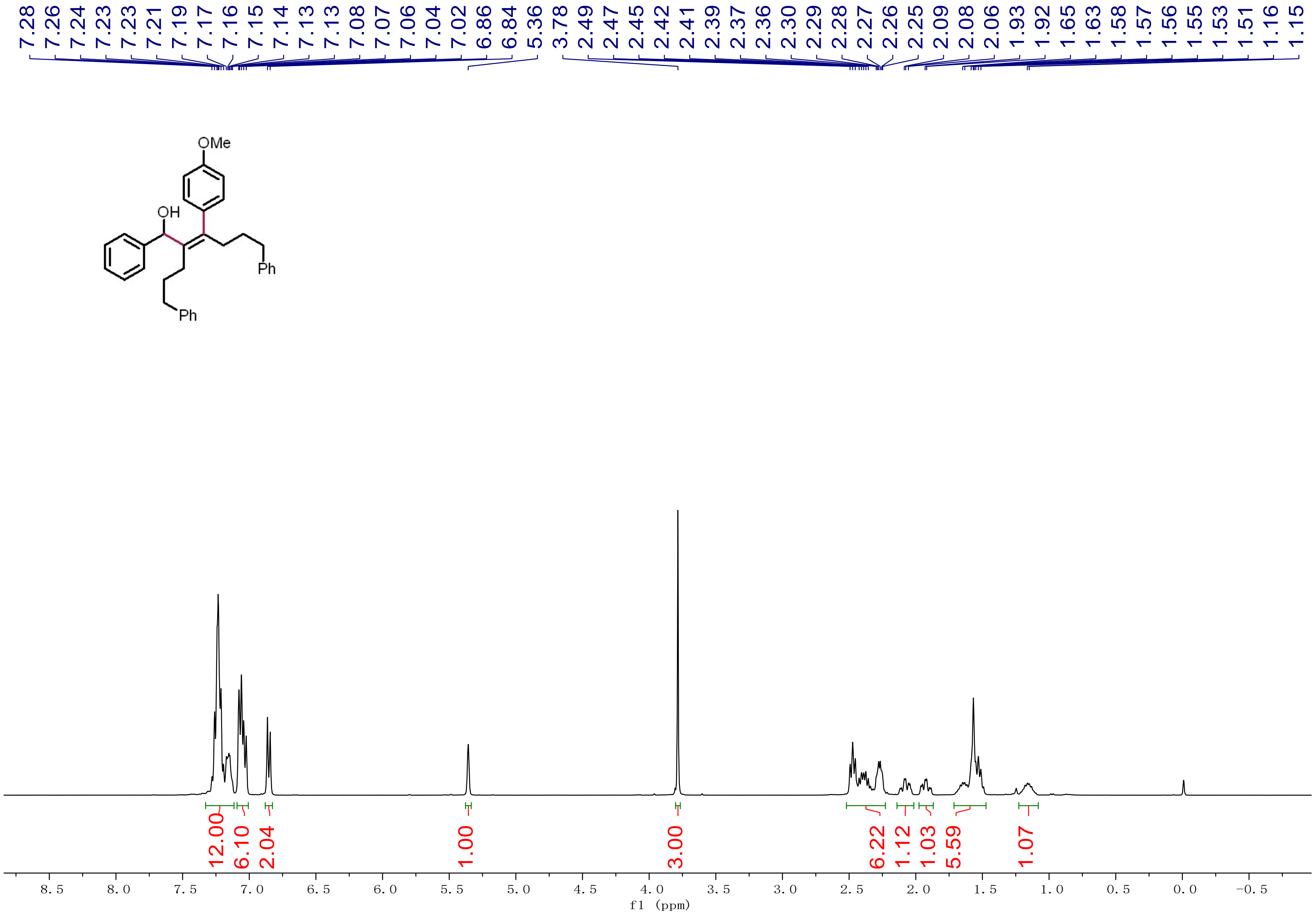

34: (Z)-3-(4-methoxyphenyl)-1,6-diphenyl-2-(3-phenylpropyl)hex-2-en-1-ol (^{13}C NMR, $CDCl_3$, 100 MHz)

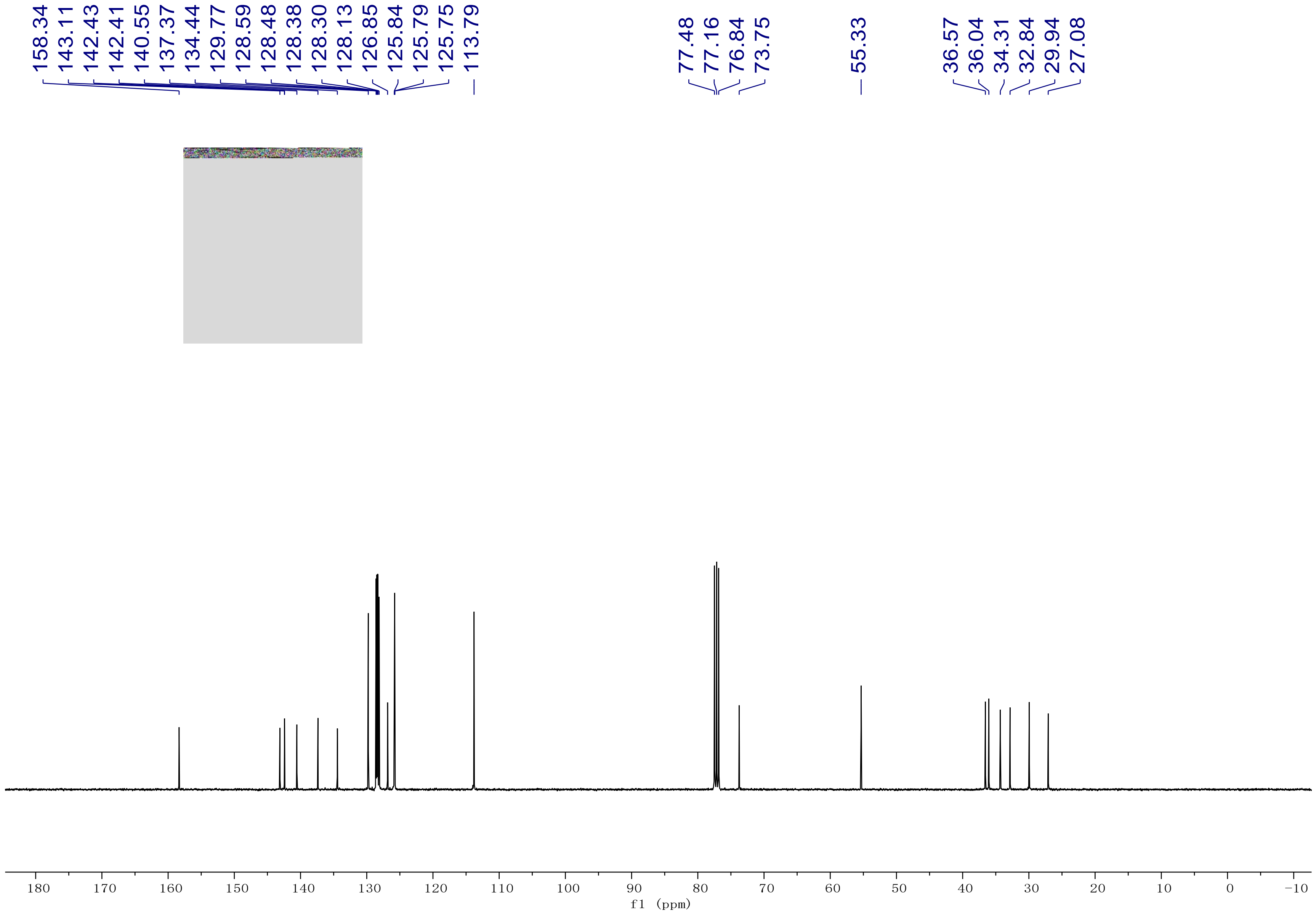

35: (*Z*)-(2-(4-methoxyphenyl)cyclododec-1-en-1-yl)(phenyl)methanol (^{1}H NMR, $CDCl_3$, 400 MHz)

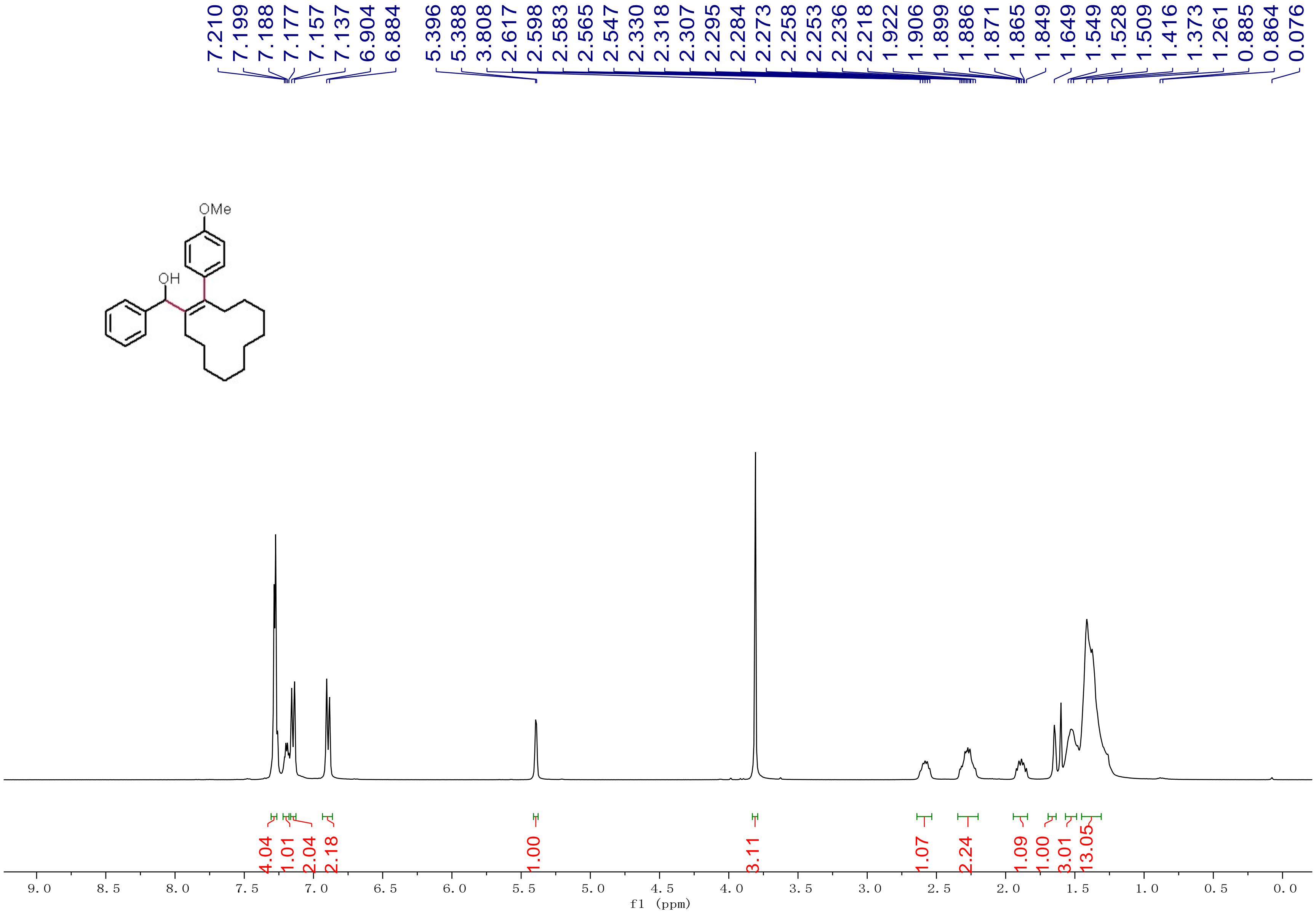

35: (*Z*)-(2-(4-methoxyphenyl)cyclododec-1-en-1-yl)(phenyl)methanol (^{13}C NMR, $CDCl_3$, 100 MHz)

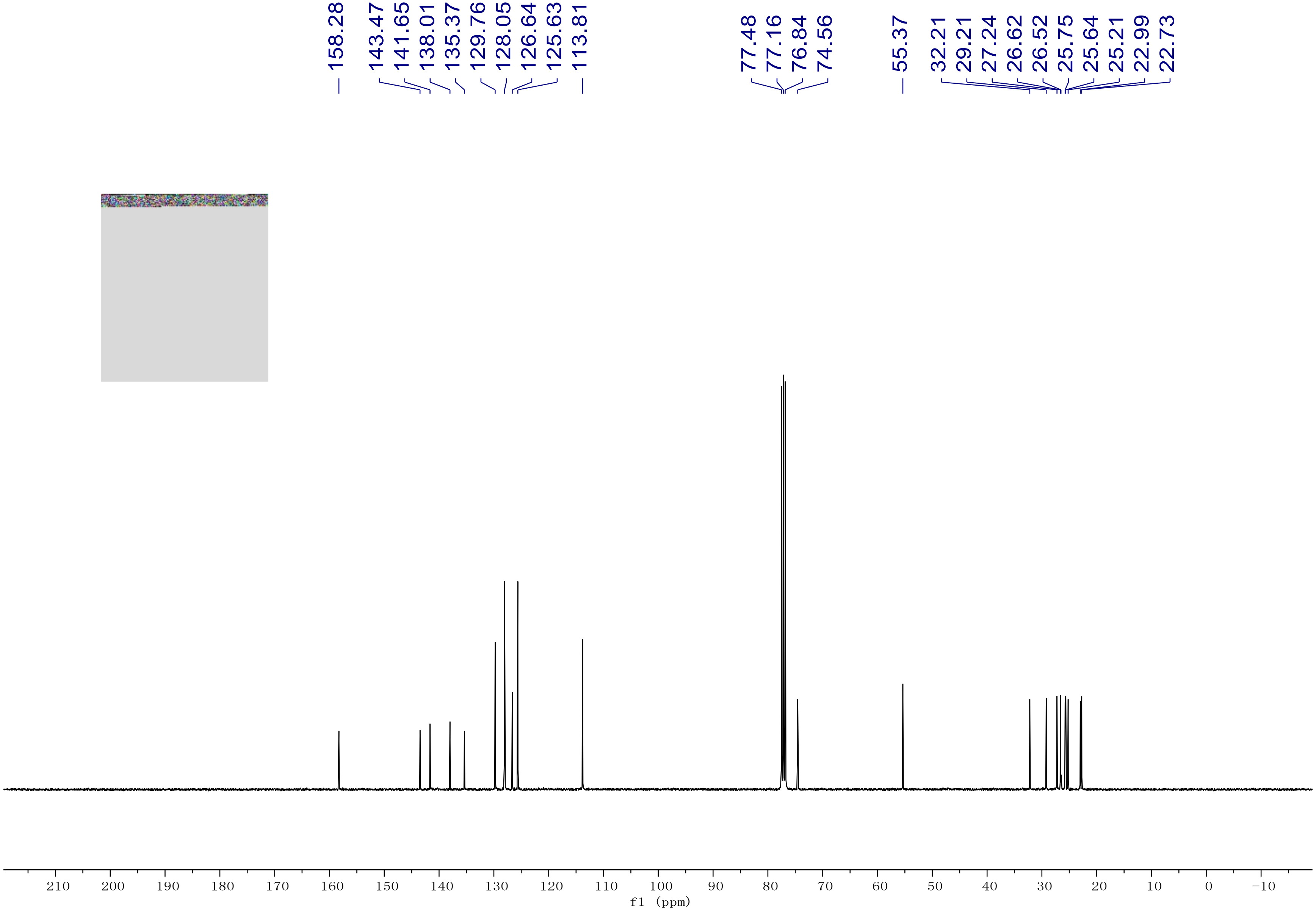

36: methyl (Z)-4-(1-hydroxy-3-(4-methoxyphenyl)non-2-en-1-yl)benzoate (^{1}H NMR, $CDCl_3$, 400 MHz)

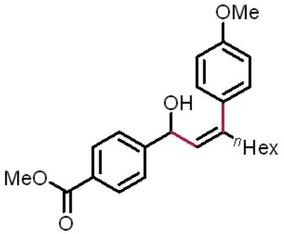

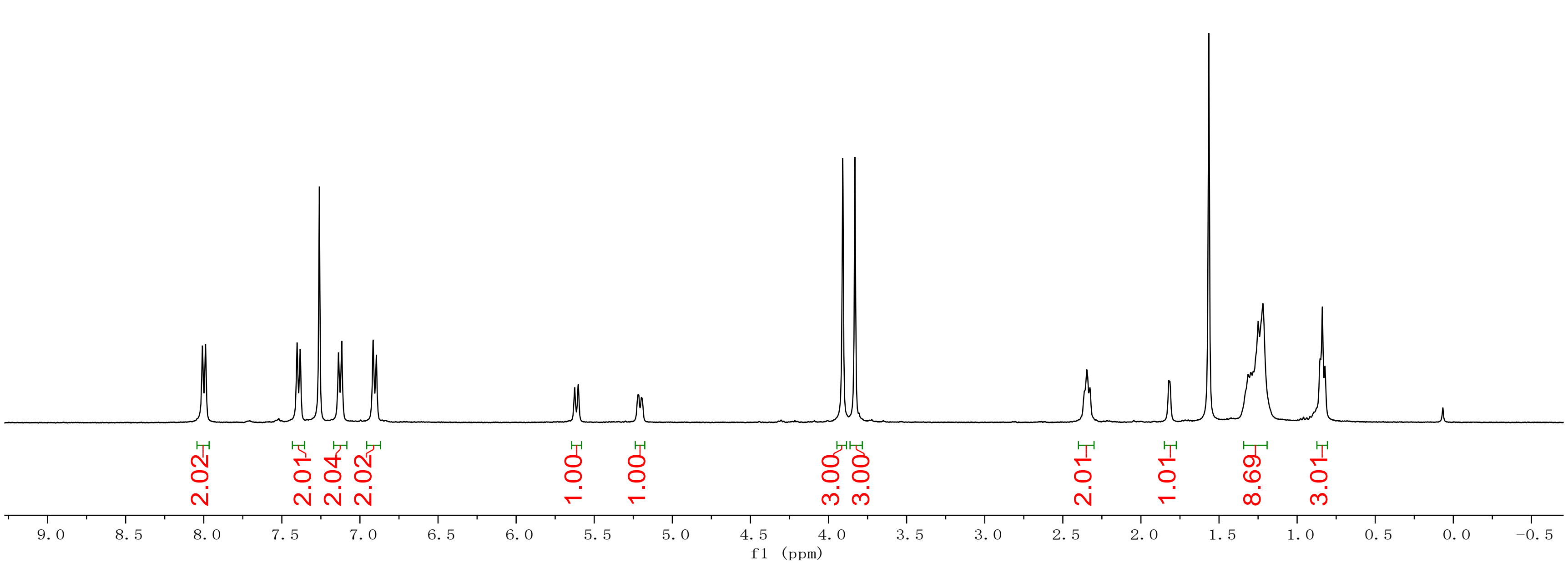

36: methyl (Z)-4-(1-hydroxy-3-(4-methoxyphenyl)non-2-en-1-yl)benzoate (^{13}C NMR, $CDCl_3$, 100 MHz)

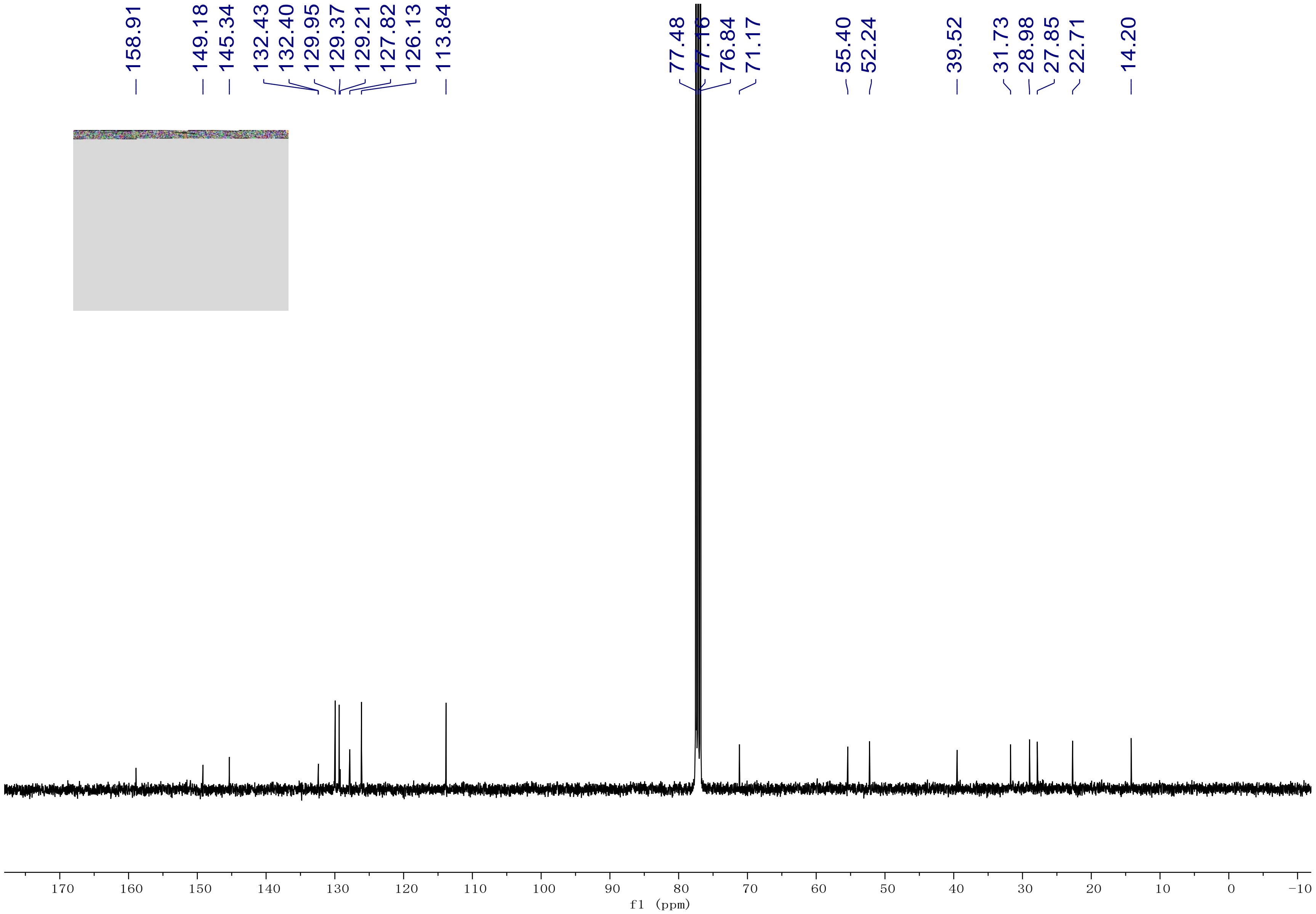

37: (Z)-3-(4-methoxyphenyl)-1-phenyl-2-propylhex-2-en-1-ol (^{1}H NMR, $CDCl_3$, 400 MHz)

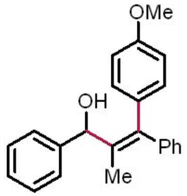

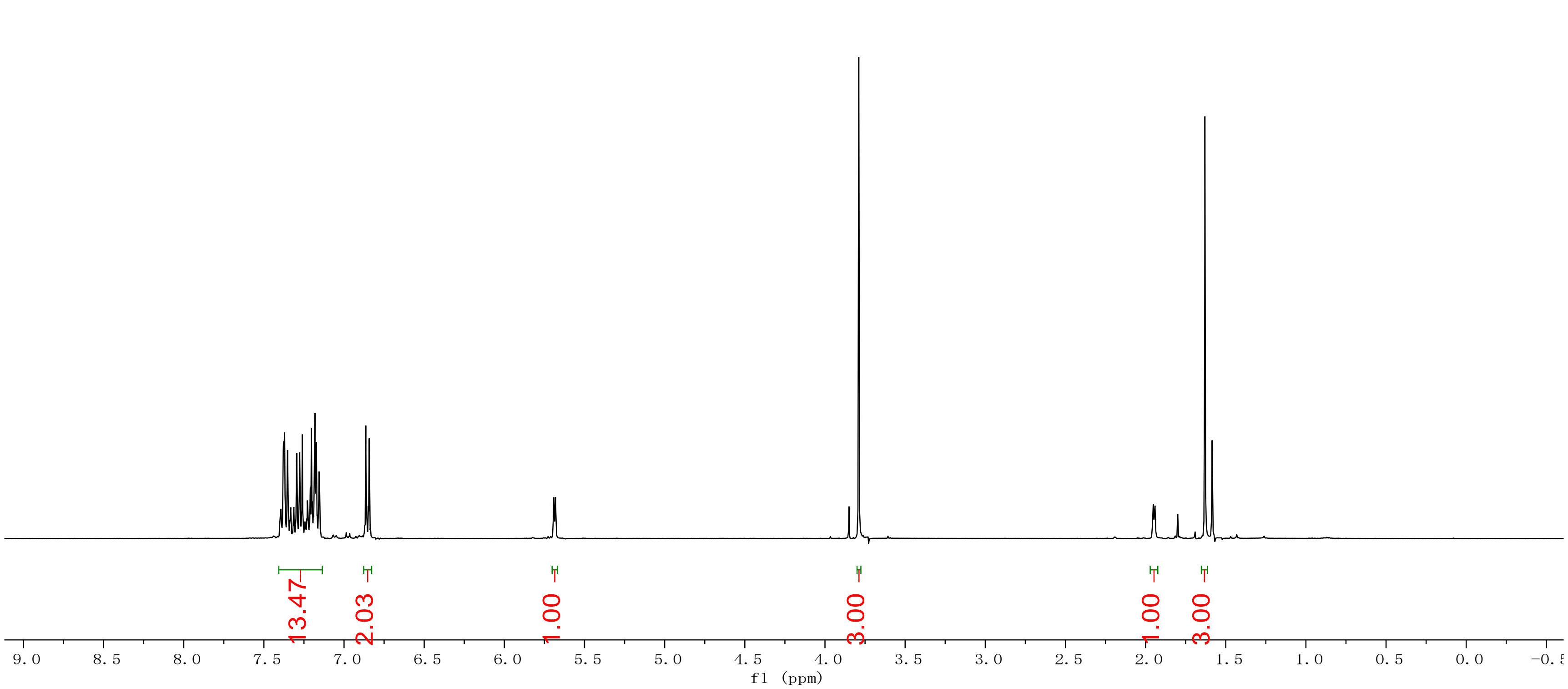

37: (Z)-3-(4-methoxyphenyl)-1-phenyl-2-propylhex-2-en-1-ol (^{13}C NMR, $CDCl_3$, 100 MHz)

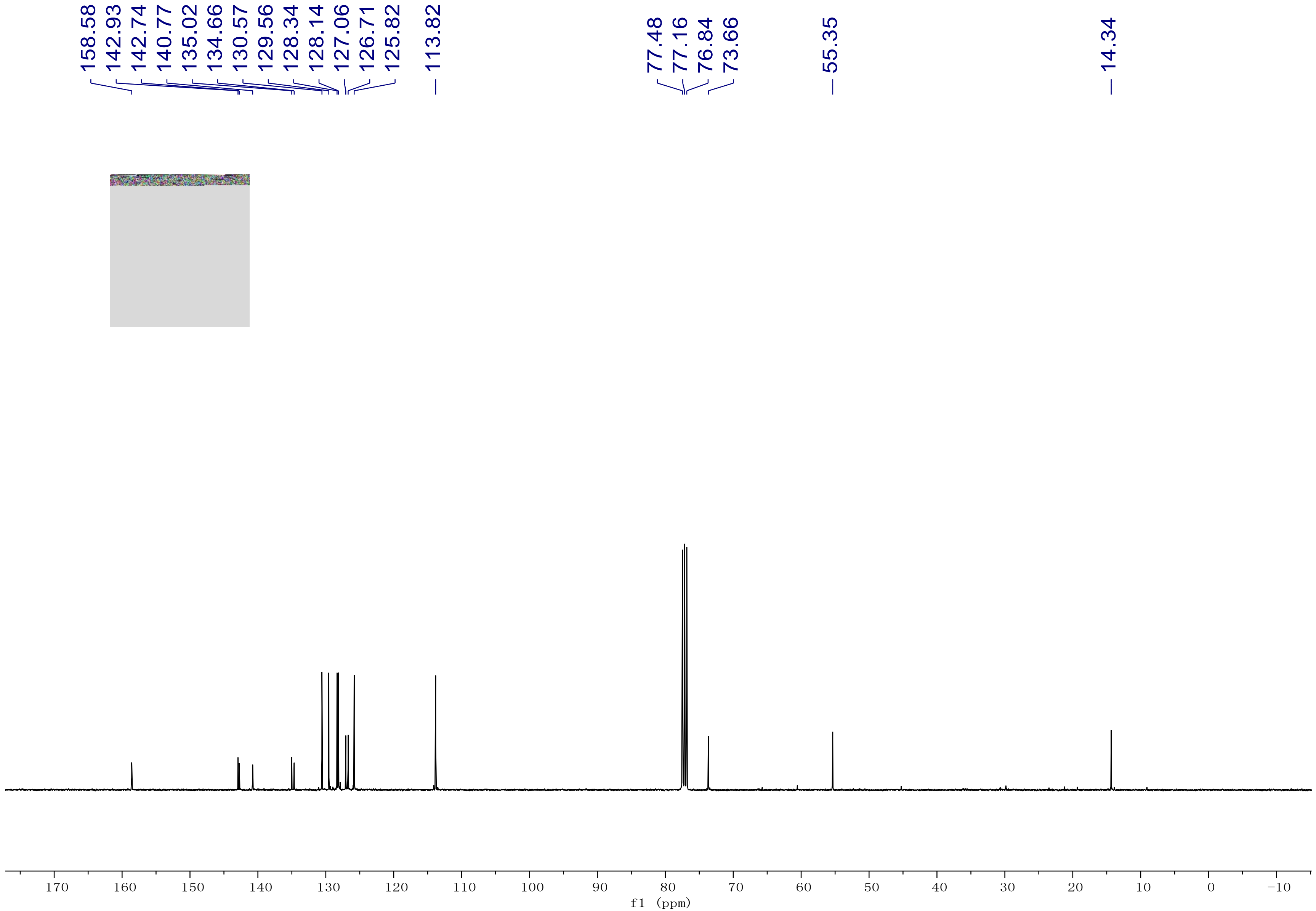

38: (Z)-2-((4-methoxyphenyl)(phenyl)methylene)-1-phenylbutan-1-ol (^{1}H NMR, $CDCl_3$, 400 MHz)

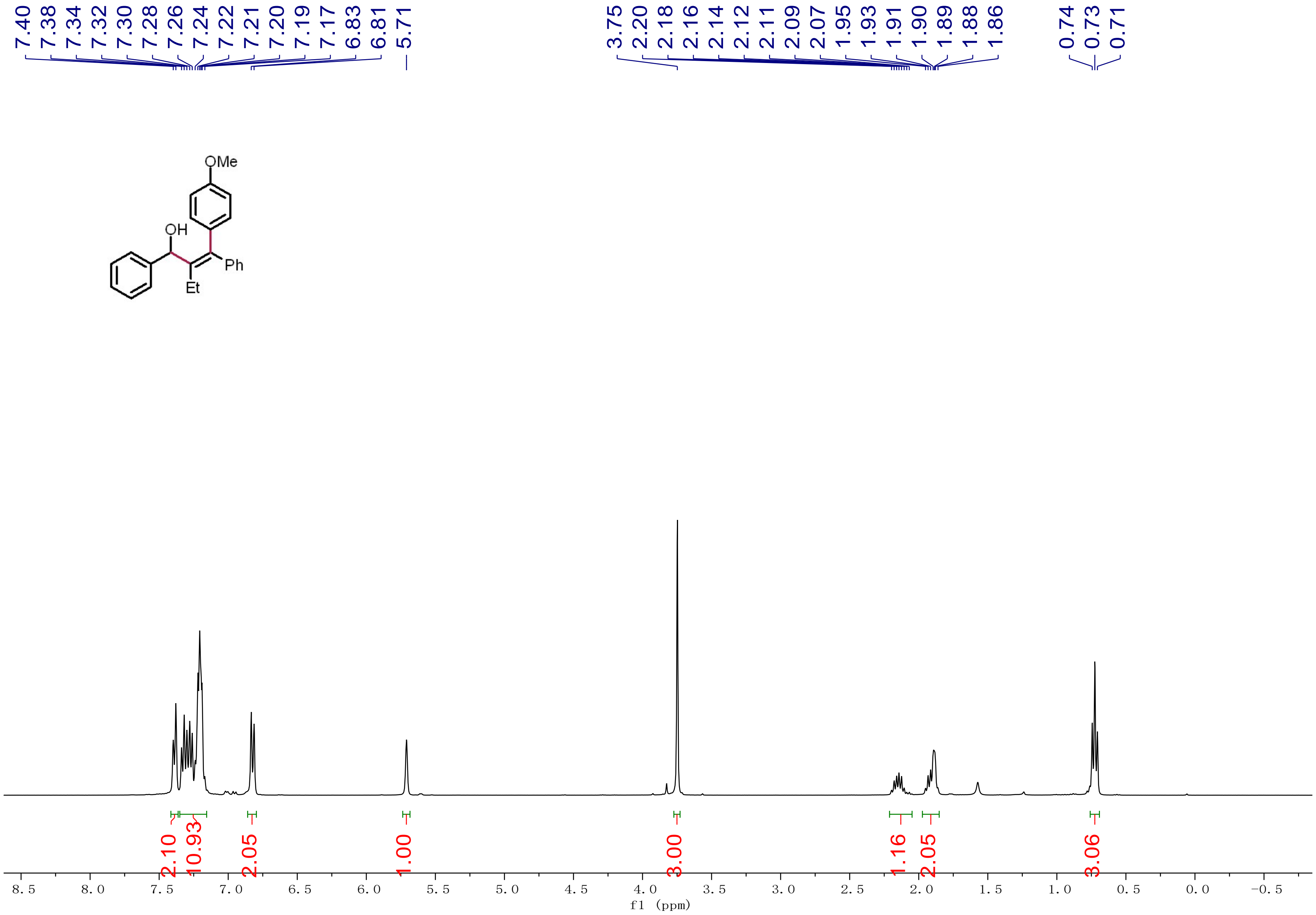

38: (Z)-2-((4-methoxyphenyl)(phenyl)methylene)-1-phenylbutan-1-ol (^{13}C NMR, $CDCl_3$, 100 MHz)

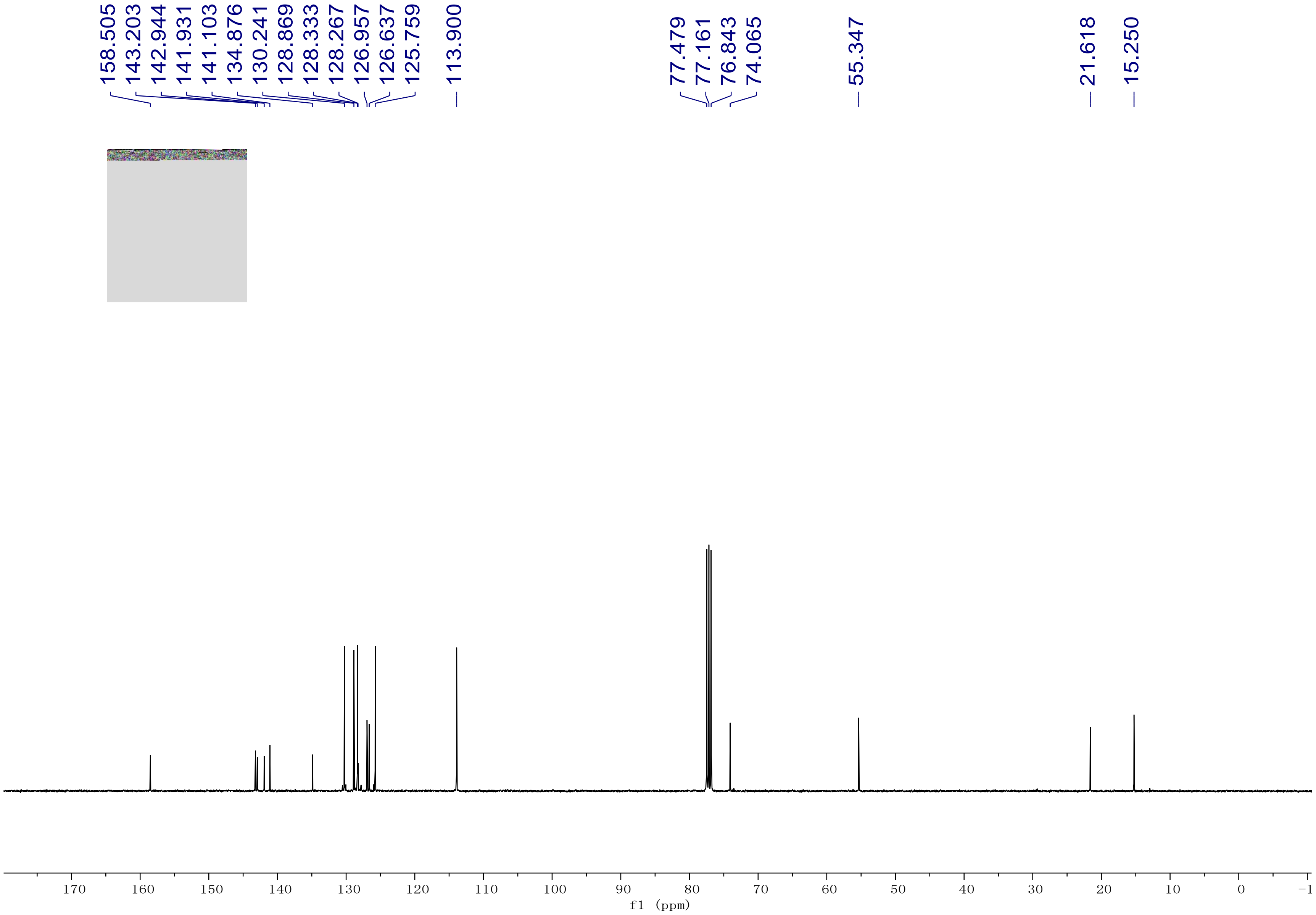

39: 3,3-bis(4-methoxyphenyl)-2-methyl-1-phenylprop-2-en-1-ol (^{1}H NMR, $CDCl_3$, 400 MHz)

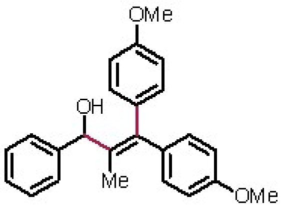

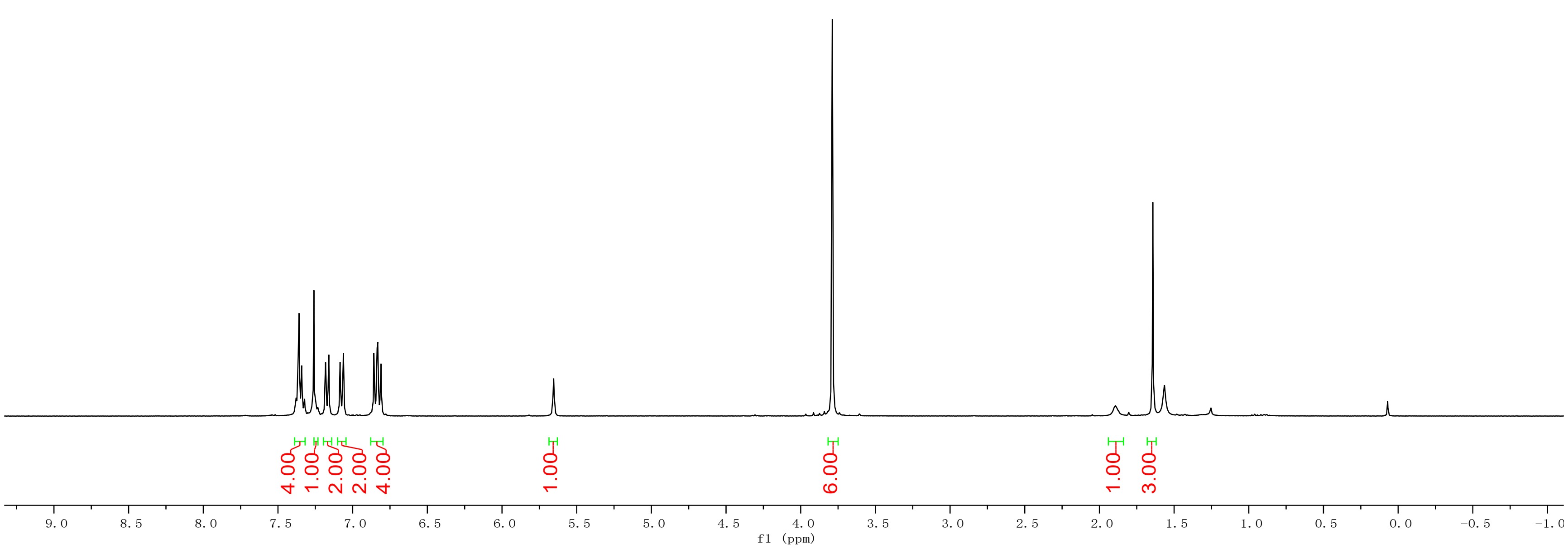

39: 3,3-bis(4-methoxyphenyl)-2-methyl-1-phenylprop-2-en-1-ol (^{13}C NMR, $CDCl_3$, 100 MHz)

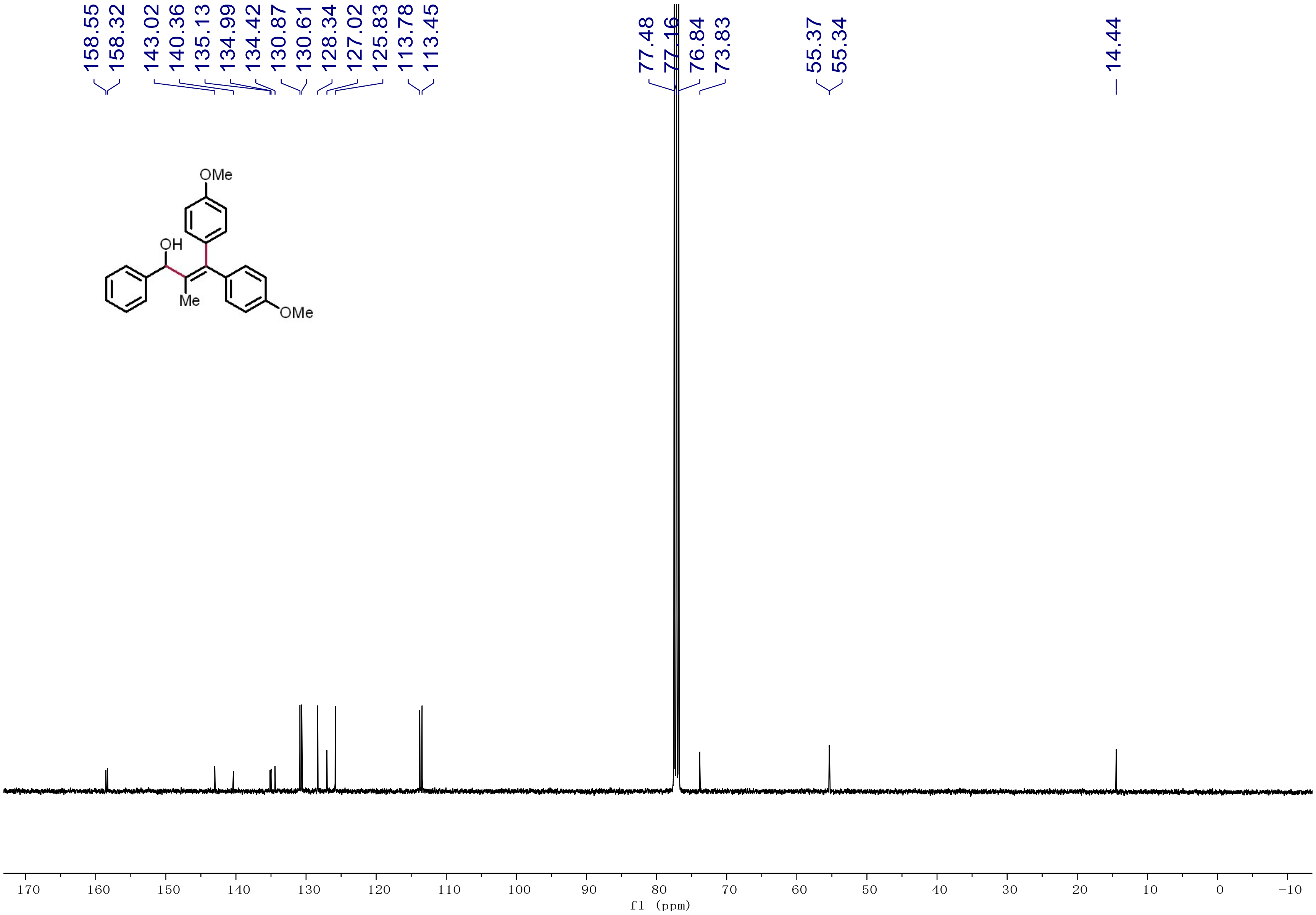

40: (E)-3-(4-methoxyphenyl)-2-methyl-1-phenyl-3-(o-tolyl)prop-2-en-1-ol (^{1}H NMR, $CDCl_3$, 400 MHz)

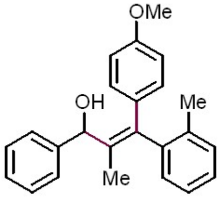

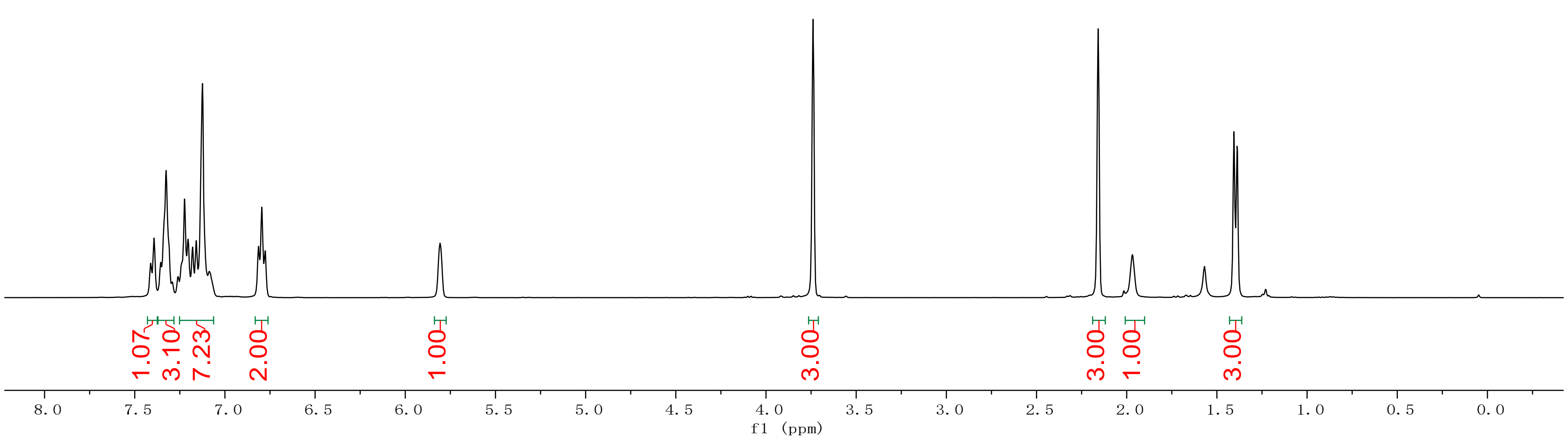

40: (E)-3-(4-methoxyphenyl)-2-methyl-1-phenyl-3-(o-tolyl)prop-2-en-1-ol (^{13}C NMR, $CDCl_3$, 100 MHz)

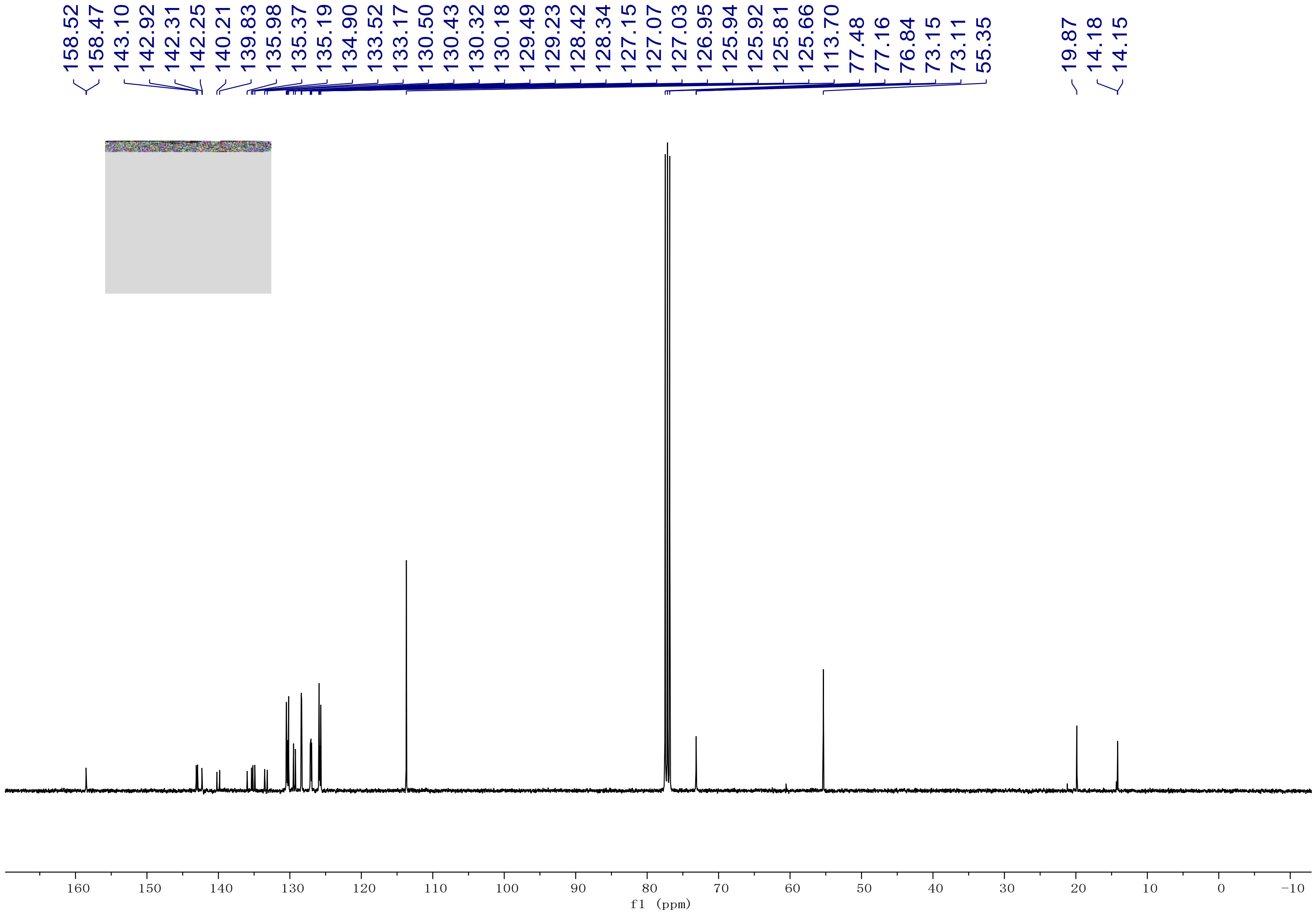

41: (Z)-1-cyclohexyl-3-(4-methoxyphenyl)-2-methyl-3-phenylprop-2-en-1-ol (^{1}H NMR, $CDCl_3$, 400 MHz)

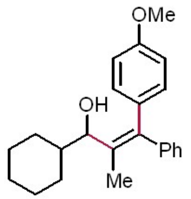

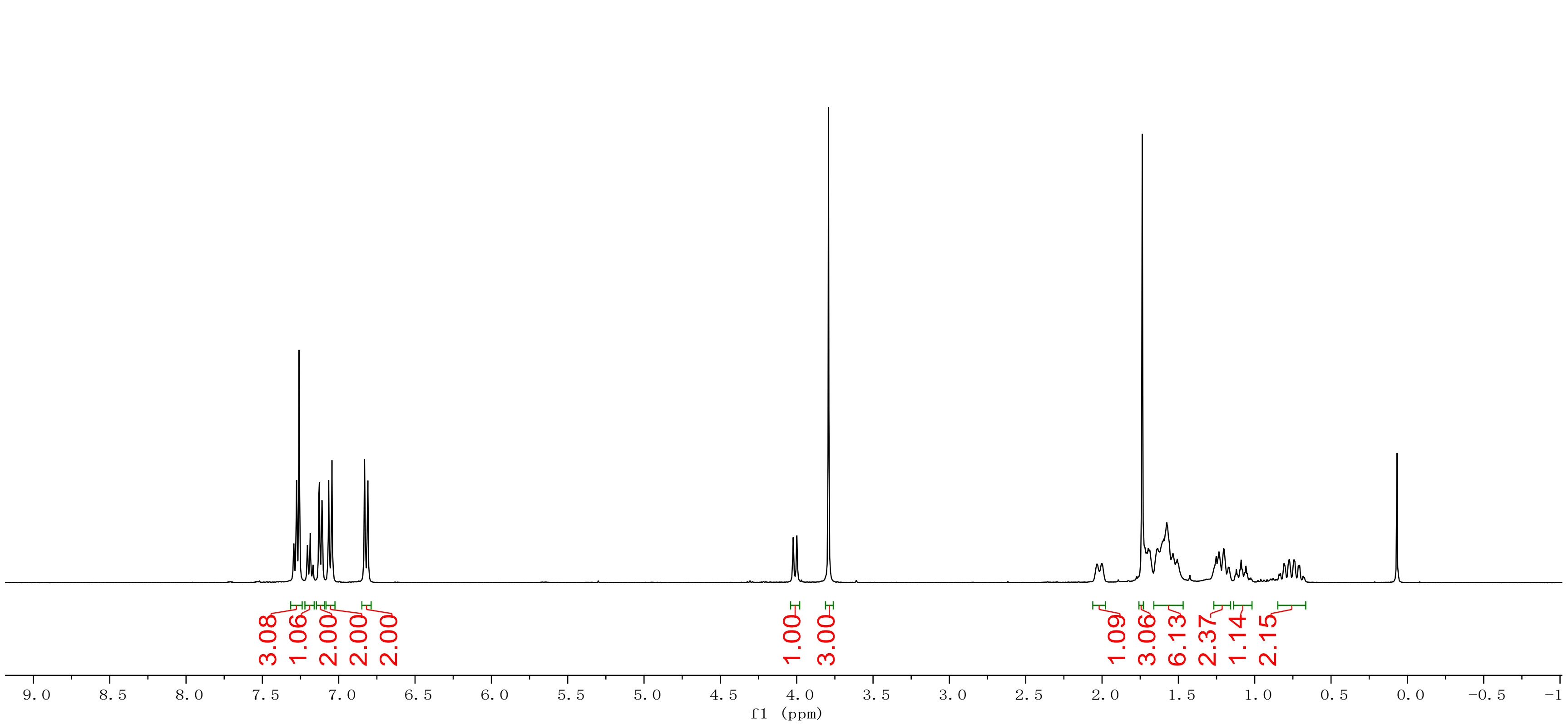

41: (Z)-1-cyclohexyl-3-(4-methoxyphenyl)-2-methyl-3-phenylprop-2-en-1-ol (^{13}C NMR, $CDCl_3$, 100 MHz)

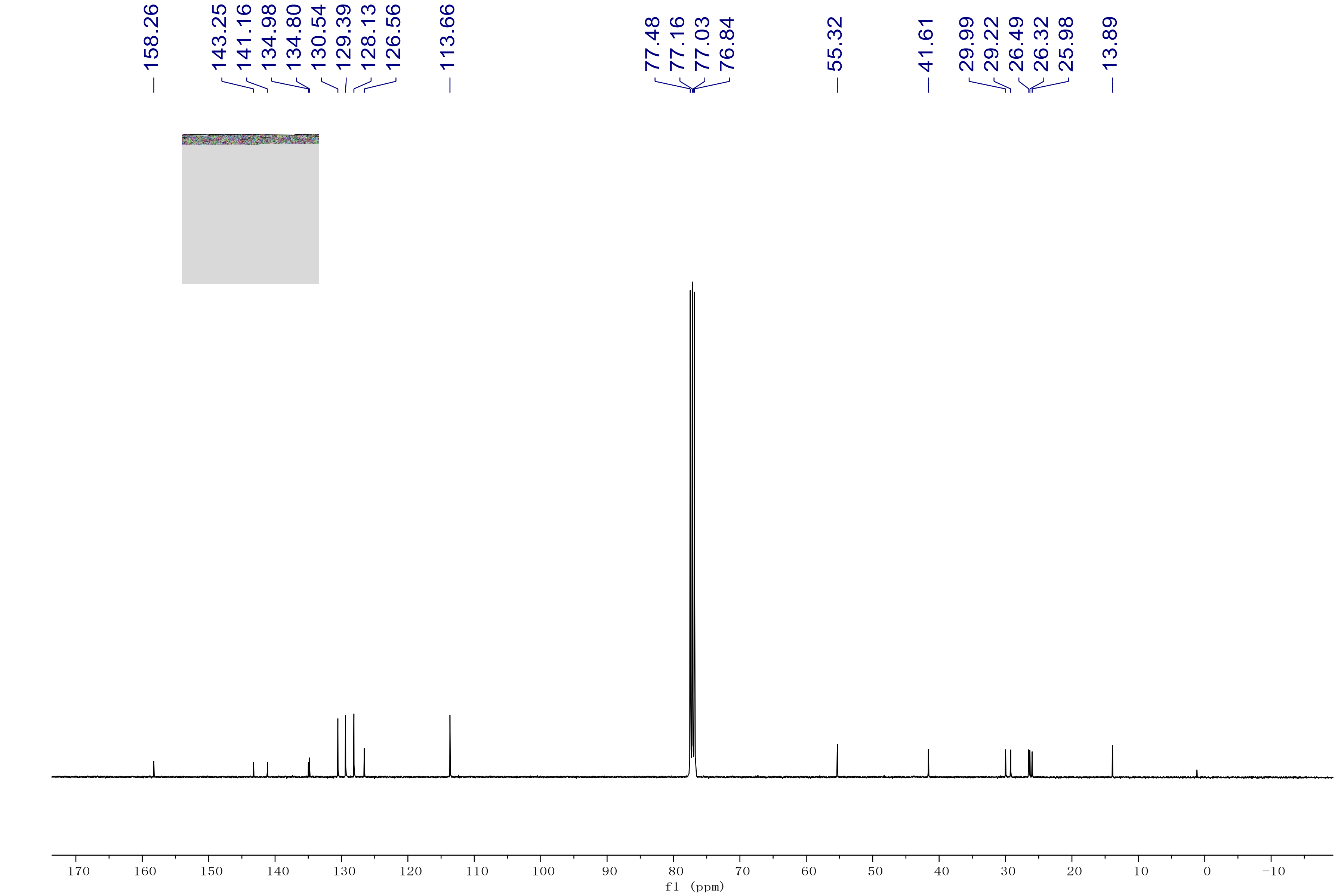

42: 2,3,3-tris(4-methoxyphenyl)-1-phenylprop-2-en-1-ol (^{1}H NMR, $CDCl_3$, 400 MHz)

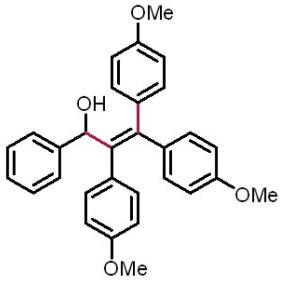

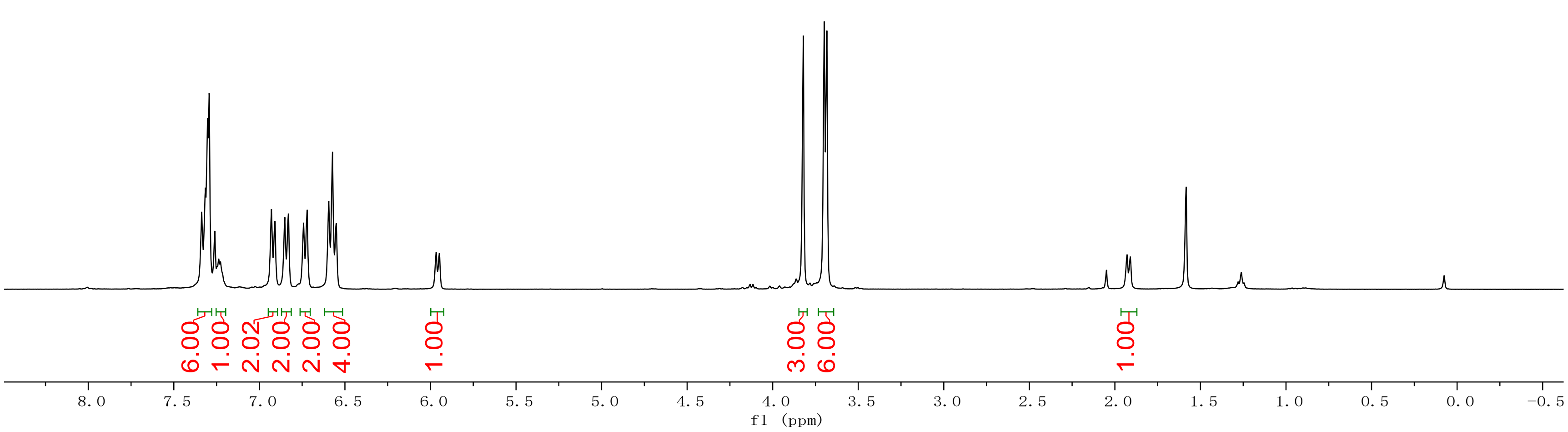

42: 2,3,3-tris(4-methoxyphenyl)-1-phenylprop-2-en-1-ol (^{13}C NMR, $CDCl_3$, 100 MHz)

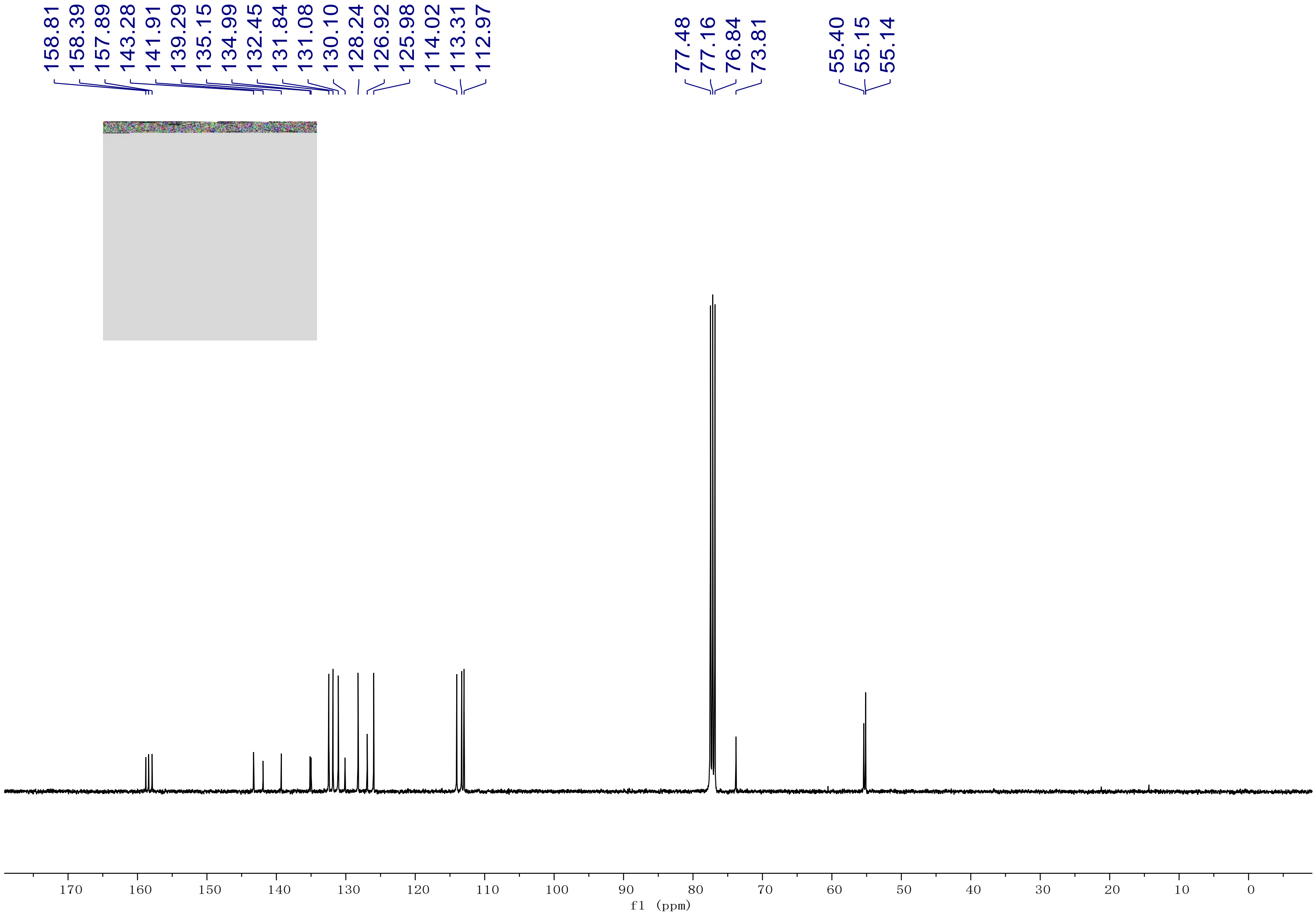

43: (Z)-3-(4-(tert-butyl)phenyl)-1-phenyl-2-propylhex-2-en-1-ol (^{1}H NMR, $CDCl_3$, 400 MHz)

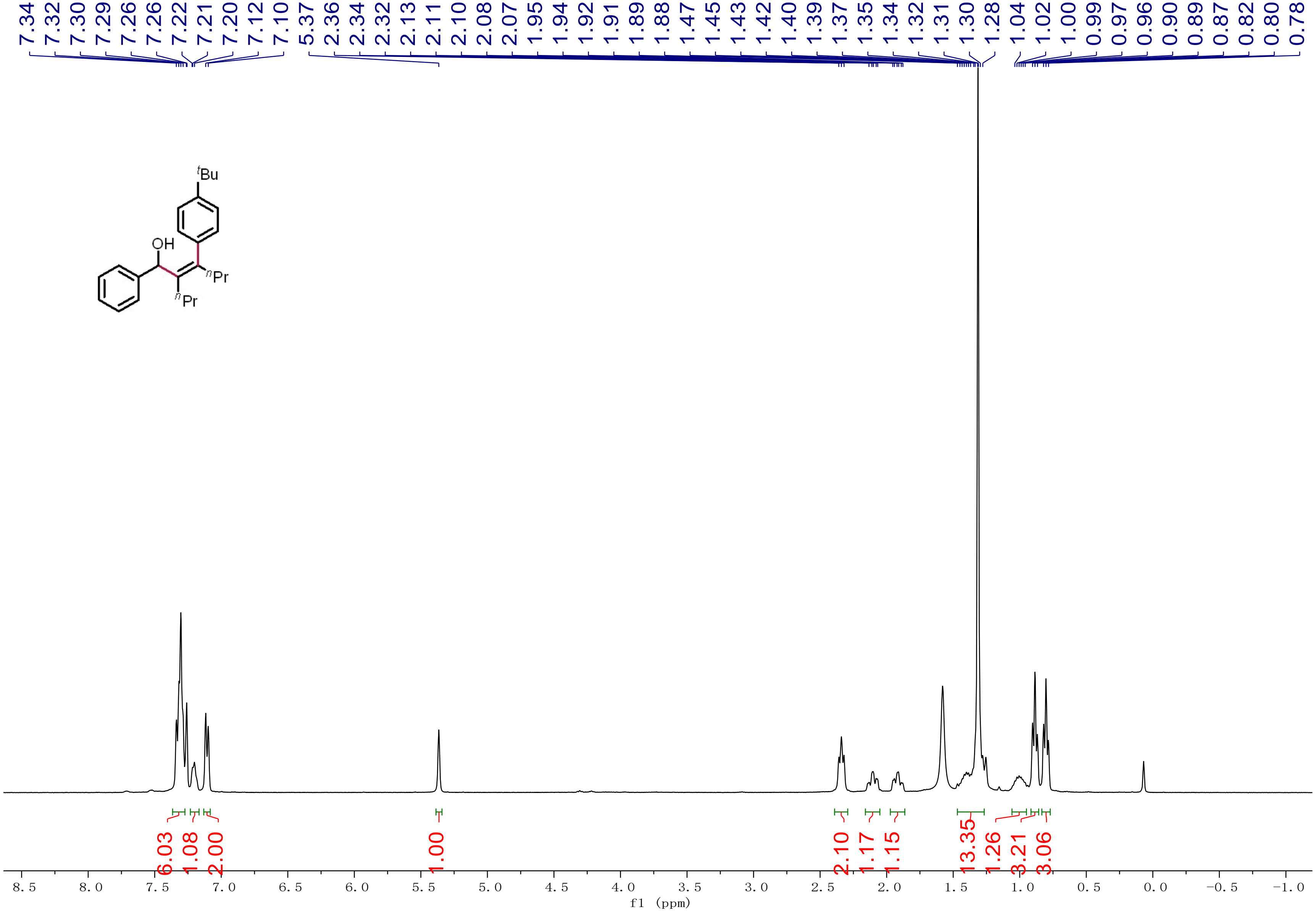

43: (Z)-3-(4-(tert-butyl)phenyl)-1-phenyl-2-propylhex-2-en-1-ol (^{13}C NMR, $CDCl_3$, 100 MHz)

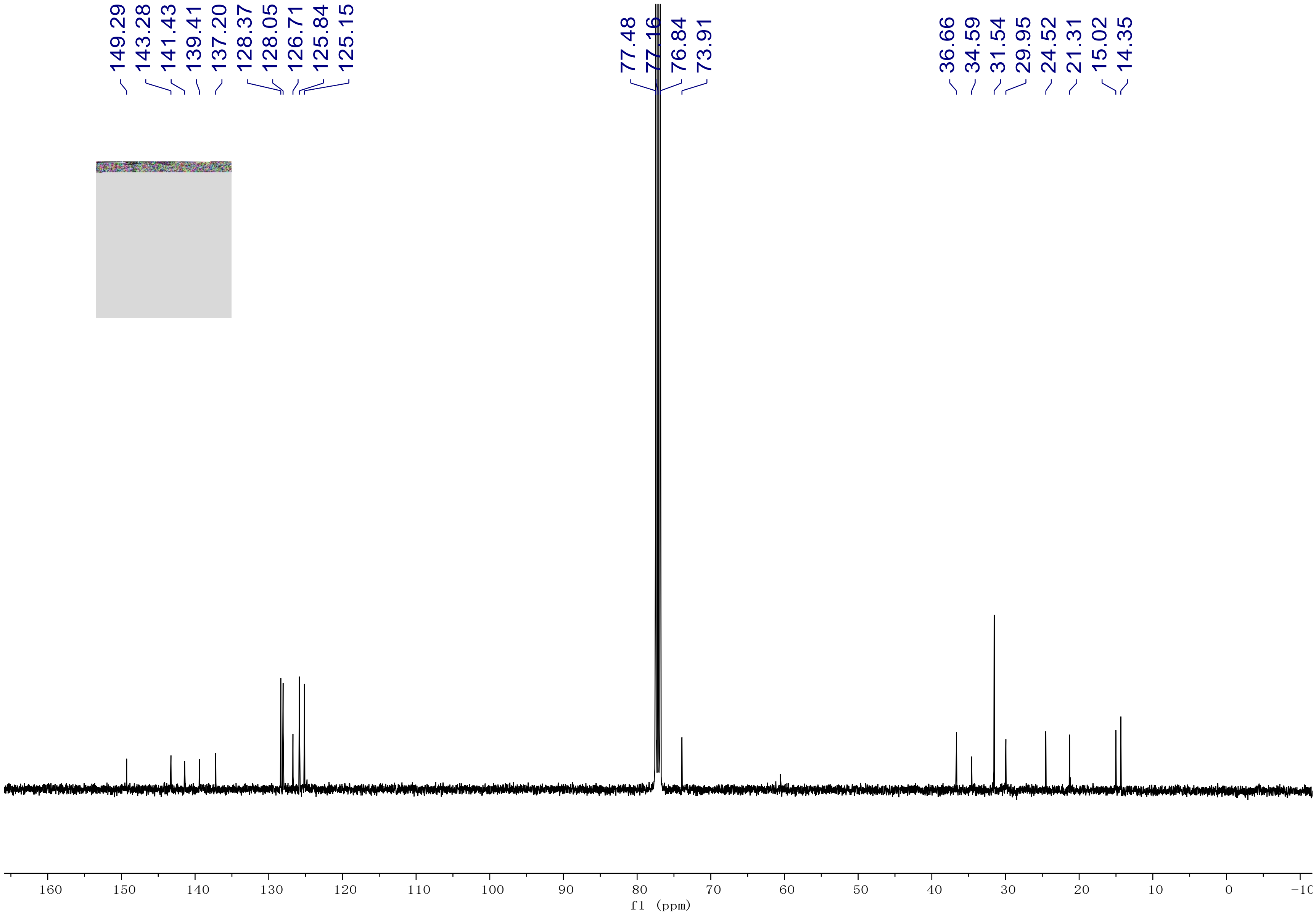

44: (Z)-3-([1,1'-biphenyl]-4-yl)-1-phenyl-2-propylhex-2-en-1-ol (^{1}H NMR, $CDCl_3$, 400 MHz)

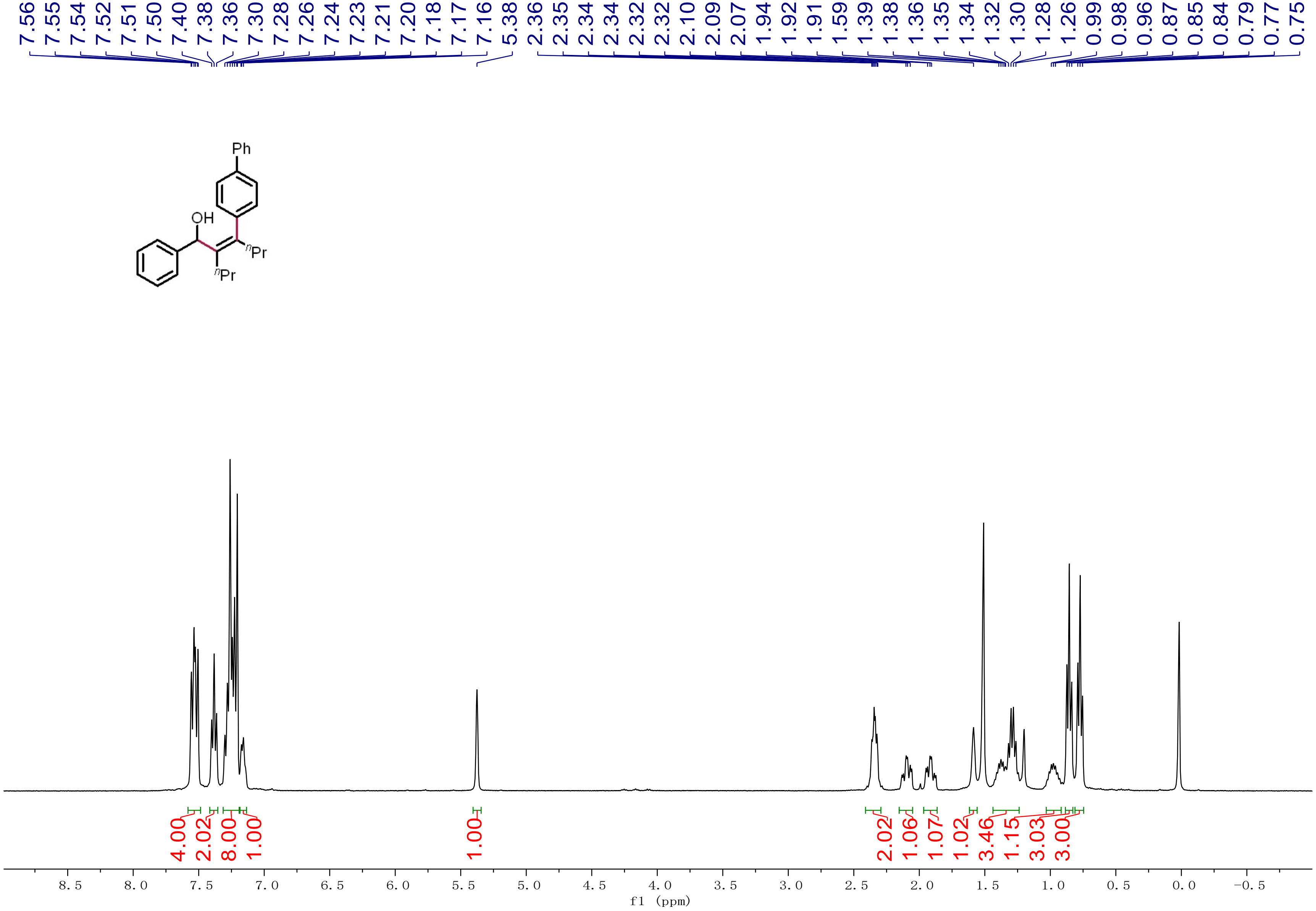

44: (Z)-3-([1,1'-biphenyl]-4-yl)-1-phenyl-2-propylhex-2-en-1-ol (^{13}C NMR, $CDCl_3$, 100 MHz)

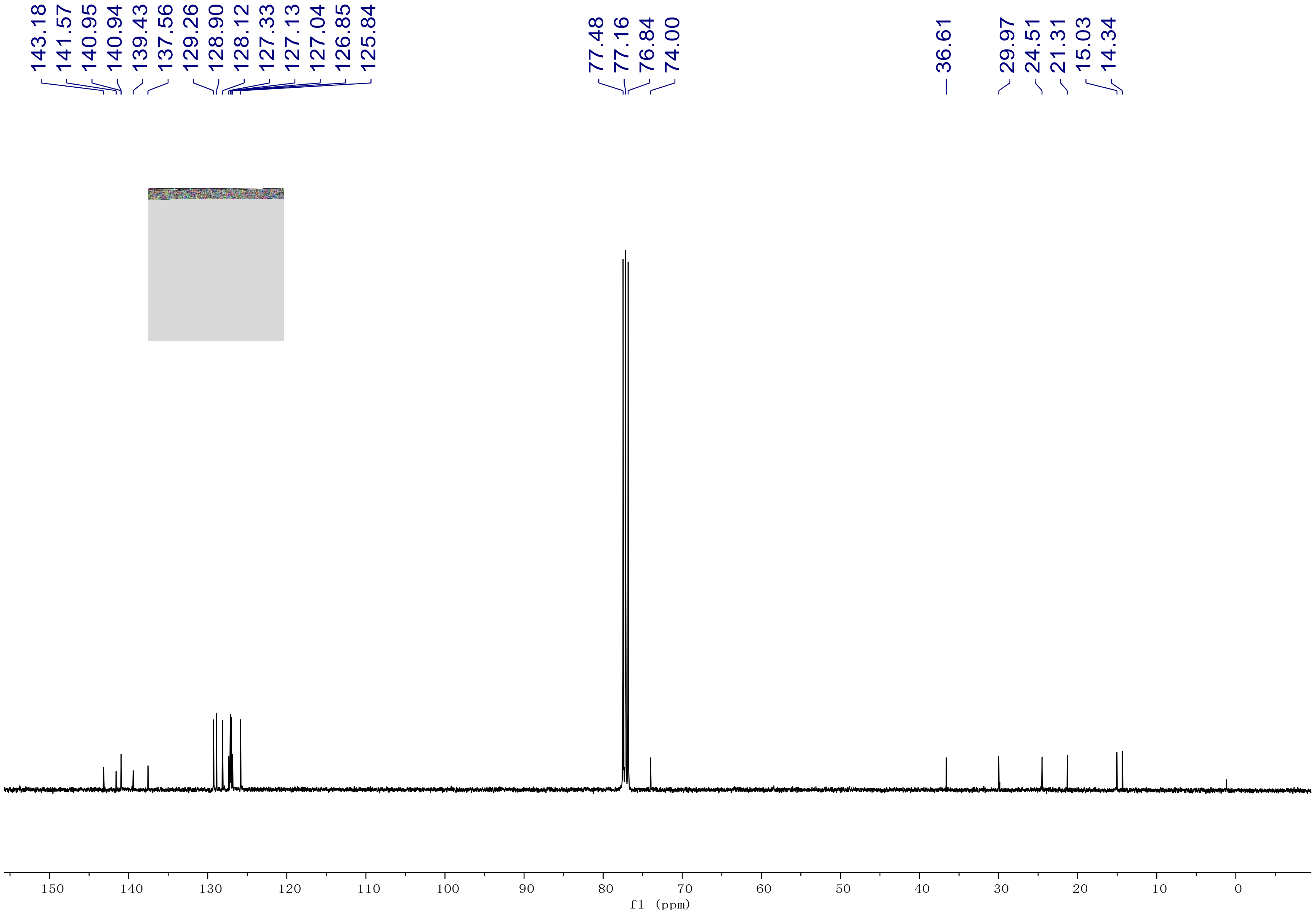

45: (Z)-3-(4-(diphenylamino)phenyl)-1-phenyl-2-propylhex-2-en-1-ol (^{1}H NMR, $CDCl_3$, 400 MHz)

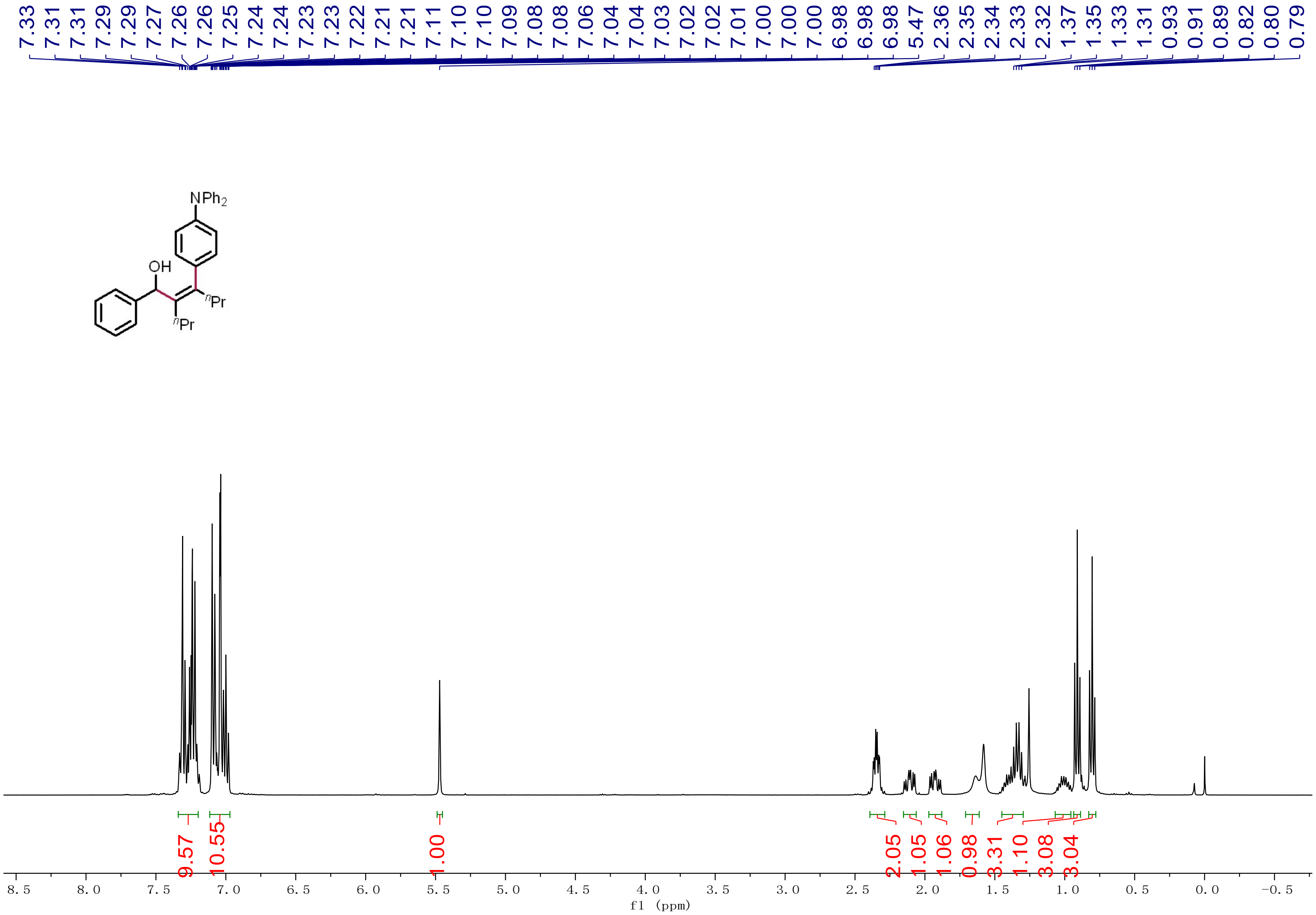

45: (Z)-3-(4-(diphenylamino)phenyl)-1-phenyl-2-propylhex-2-en-1-ol (^{13}C NMR, $CDCl_3$, 100 MHz)

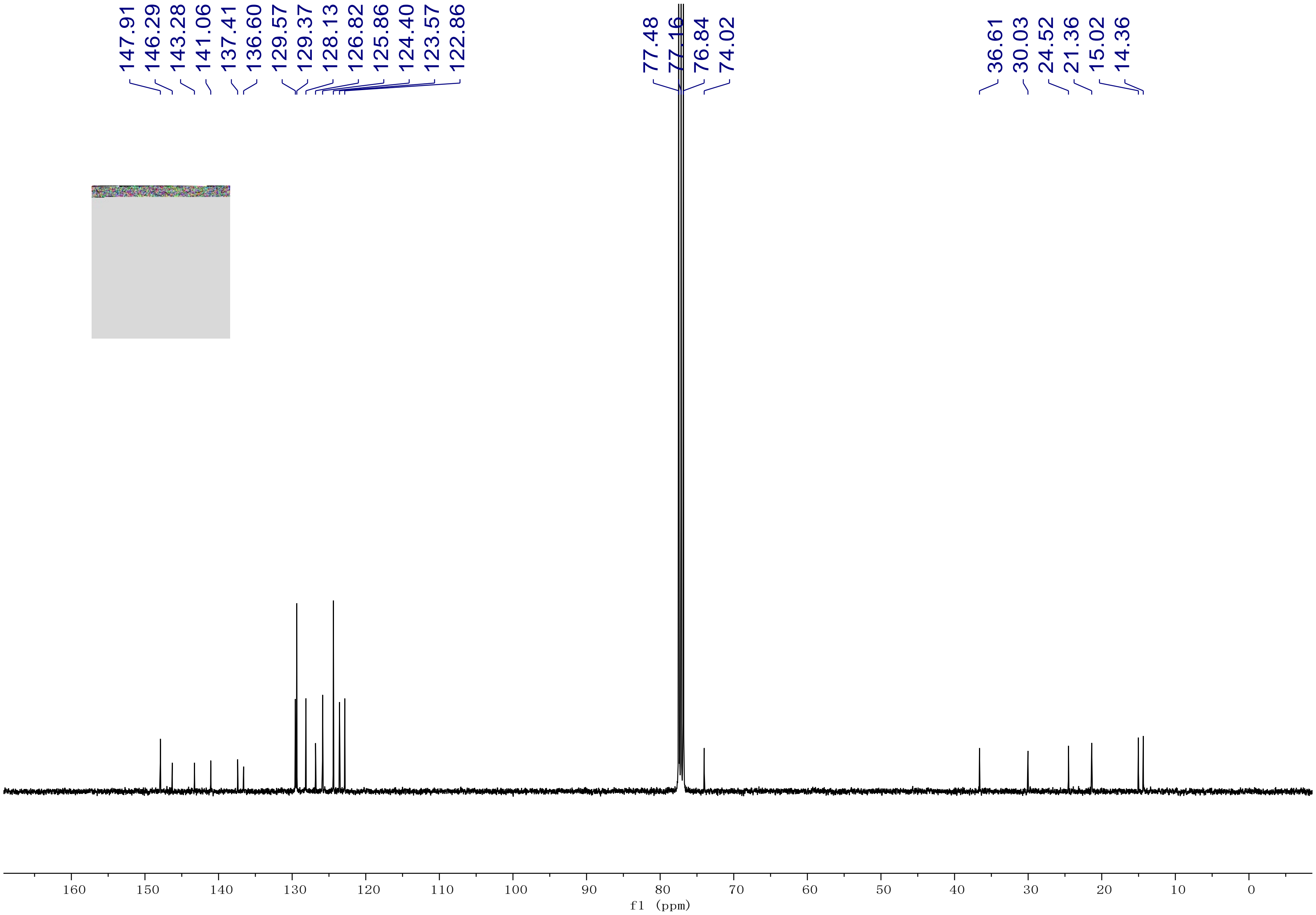

46: (Z)-3-(4-phenoxyphenyl)-1-phenyl-2-propylhex-2-en-1-ol (^{1}H NMR, $CDCl_3$, 400 MHz)

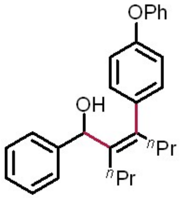

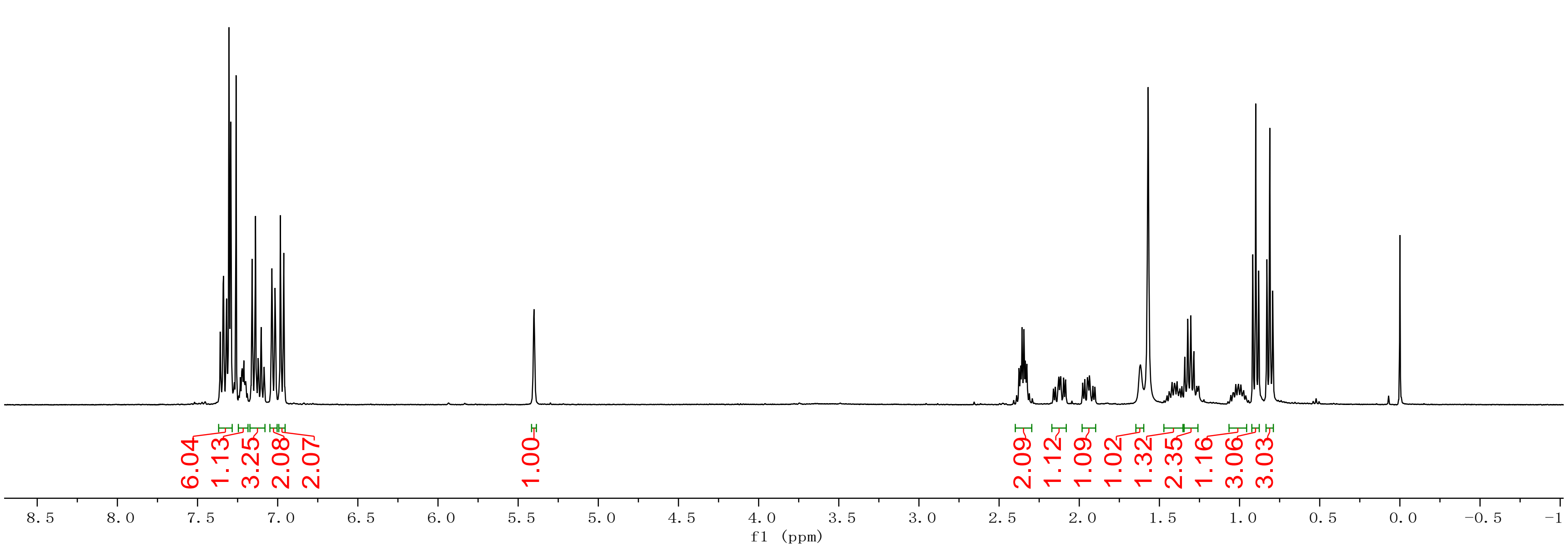

46: (Z)-3-(4-phenoxyphenyl)-1-phenyl-2-propylhex-2-en-1-ol (^{13}C NMR, $CDCl_3$, 100 MHz)

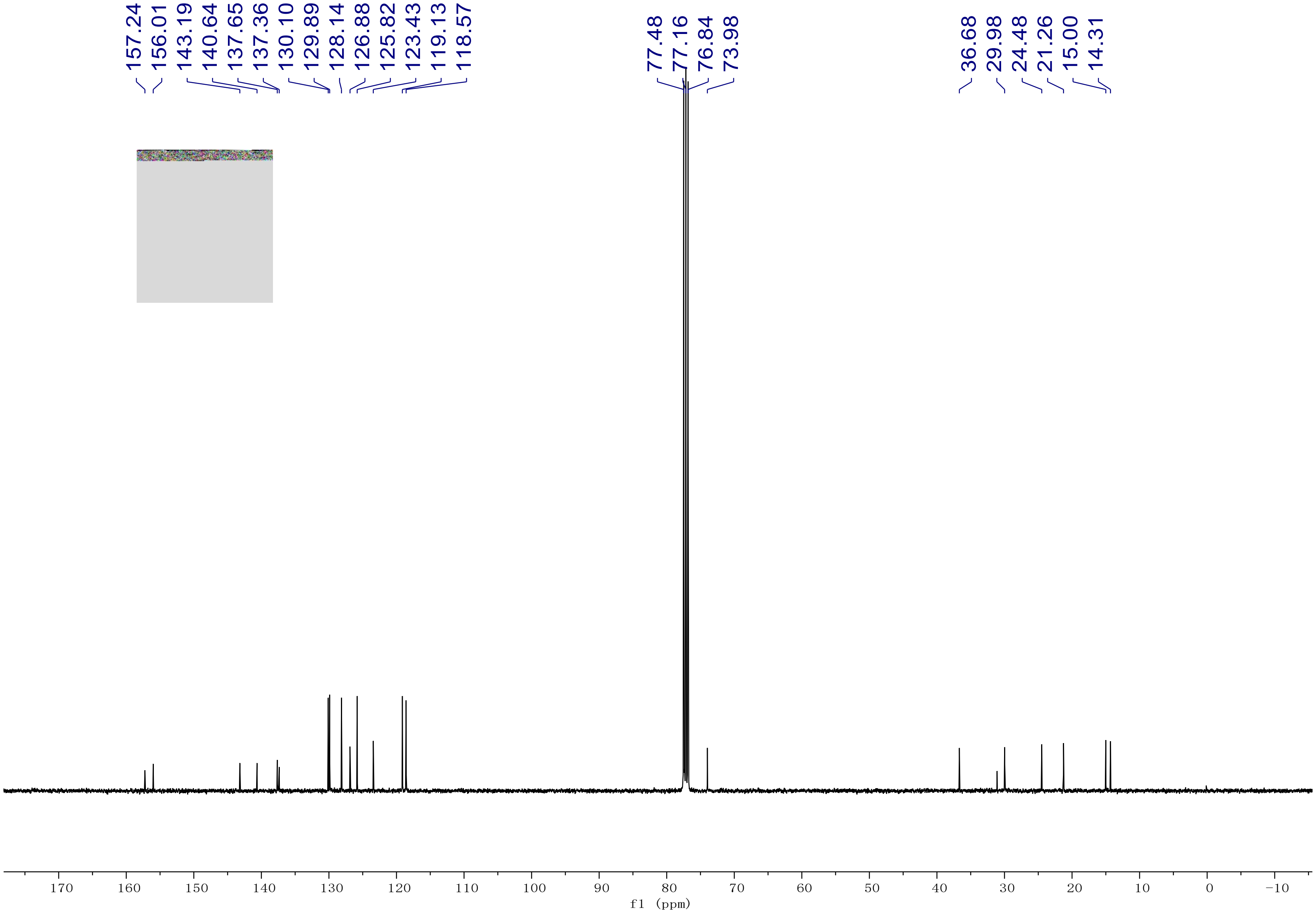

47: (Z)-4-(5-(hydroxy(phenyl)methyl)oct-4-en-4-yl)phenyl acetate (^1H NMR, $CDCl_3$, 400 MHz)

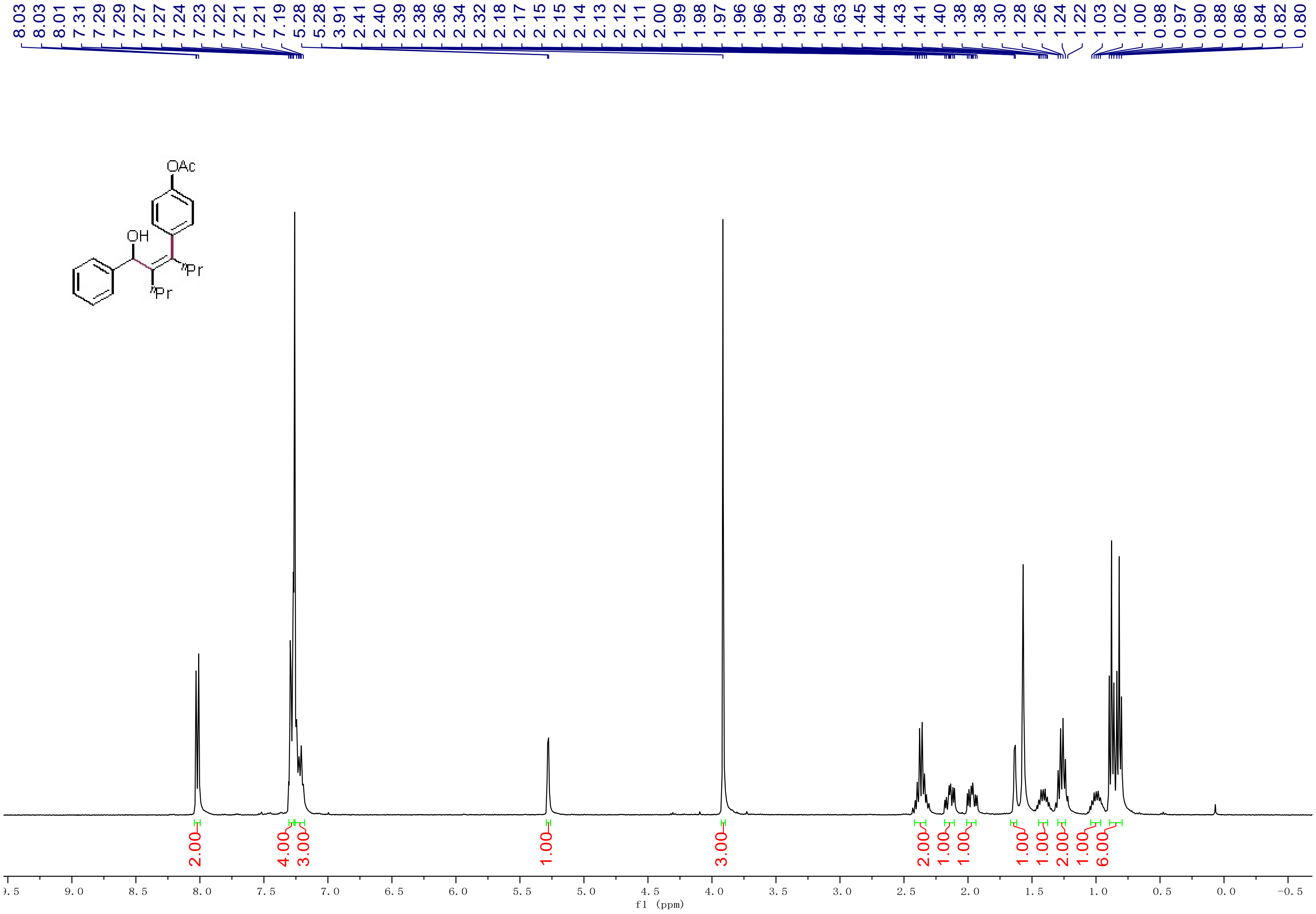

47: (Z)-4-(5-(hydroxy(phenyl)methyl)oct-4-en-4-yl)phenyl acetate (^{13}C NMR, $CDCl_3$, 100 MHz)

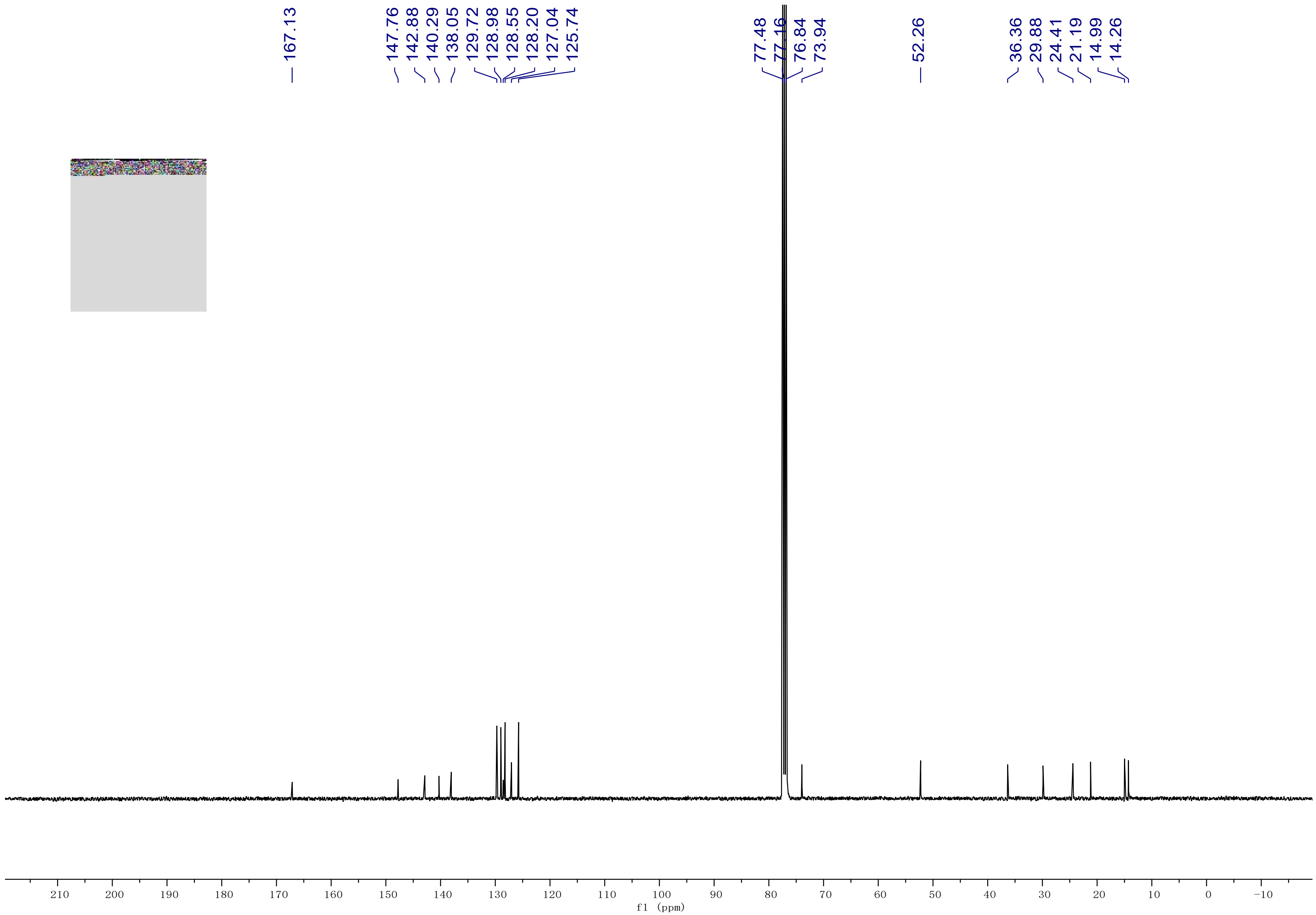

48: (Z)-(4-(5-(hydroxy(phenyl)methyl)oct-4-en-4-yl)phenyl)(phenyl)methanone (^{1}H NMR, $CDCl_3$, 400 MHz)

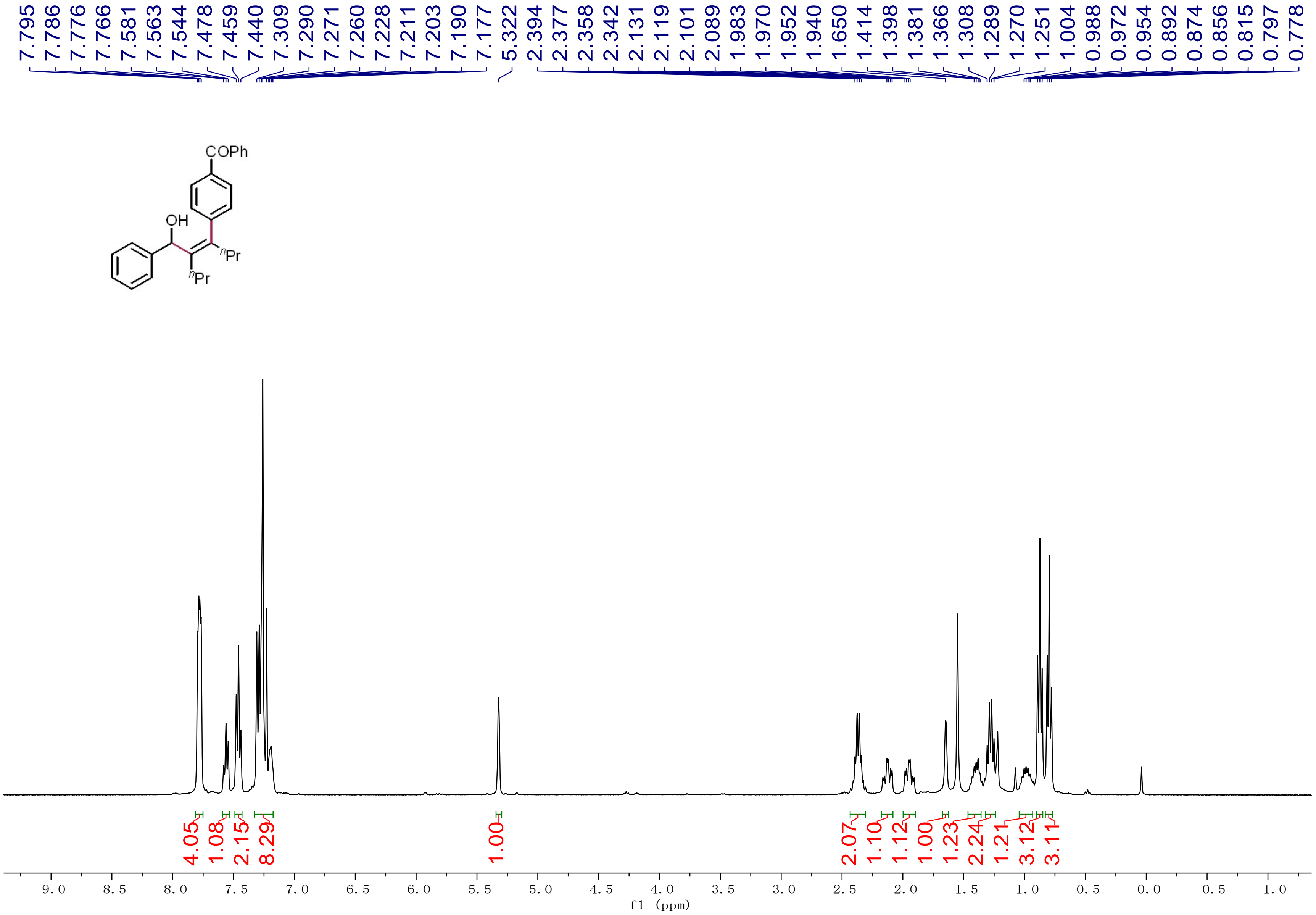

48: (Z)-(4-(5-(hydroxy(phenyl)methyl)oct-4-en-4-yl)phenyl)(phenyl)methanone (^{13}C NMR, $CDCl_3$, 100 MHz)

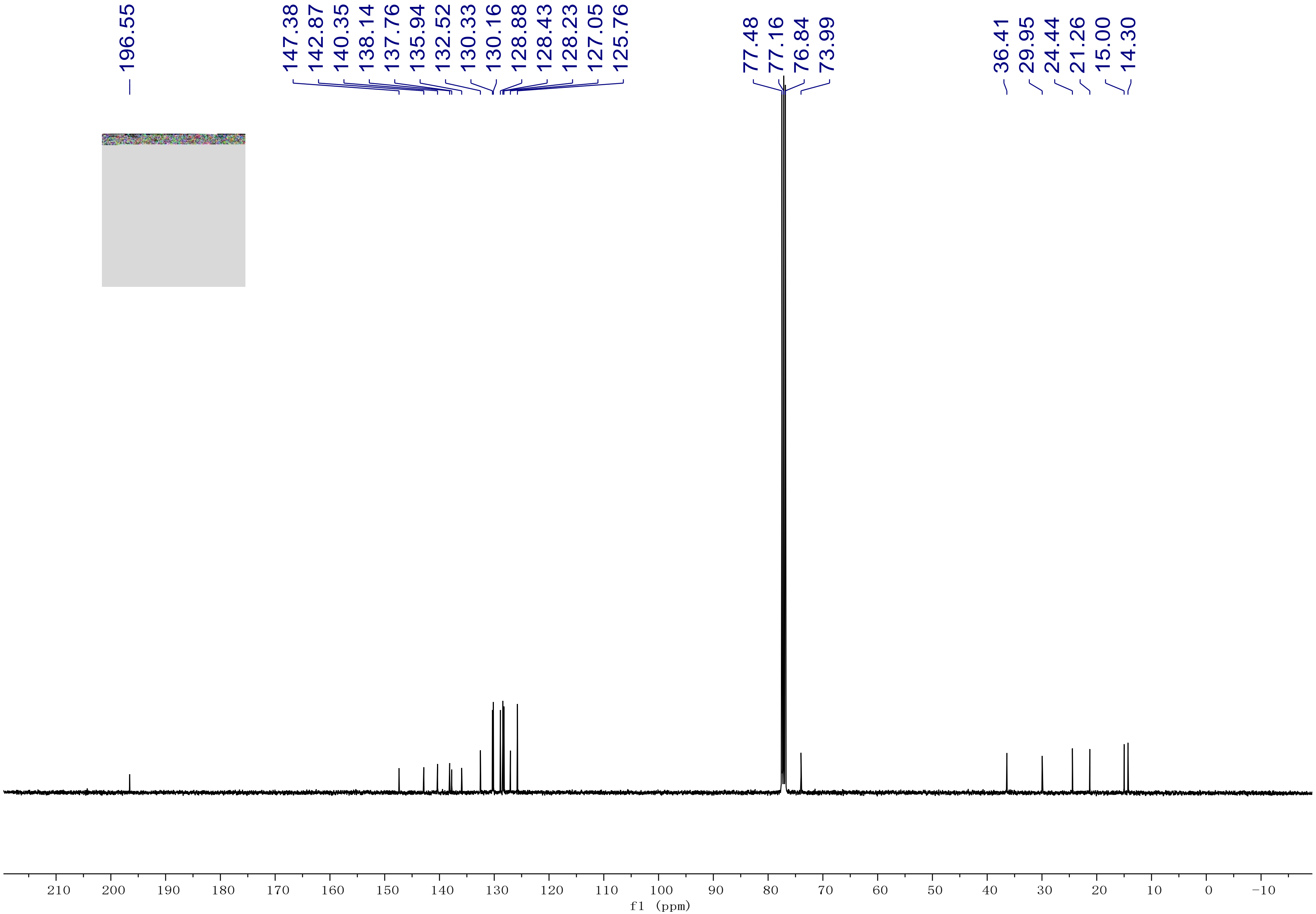

49: (*Z*)-3-(2,3-dihydrobenzofuran-5-yl)-1-phenyl-2-propylhex-2-en-1-ol (^{1}H NMR, $CDCl_3$, 400 MHz)

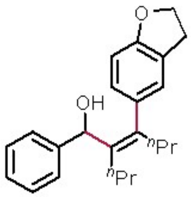

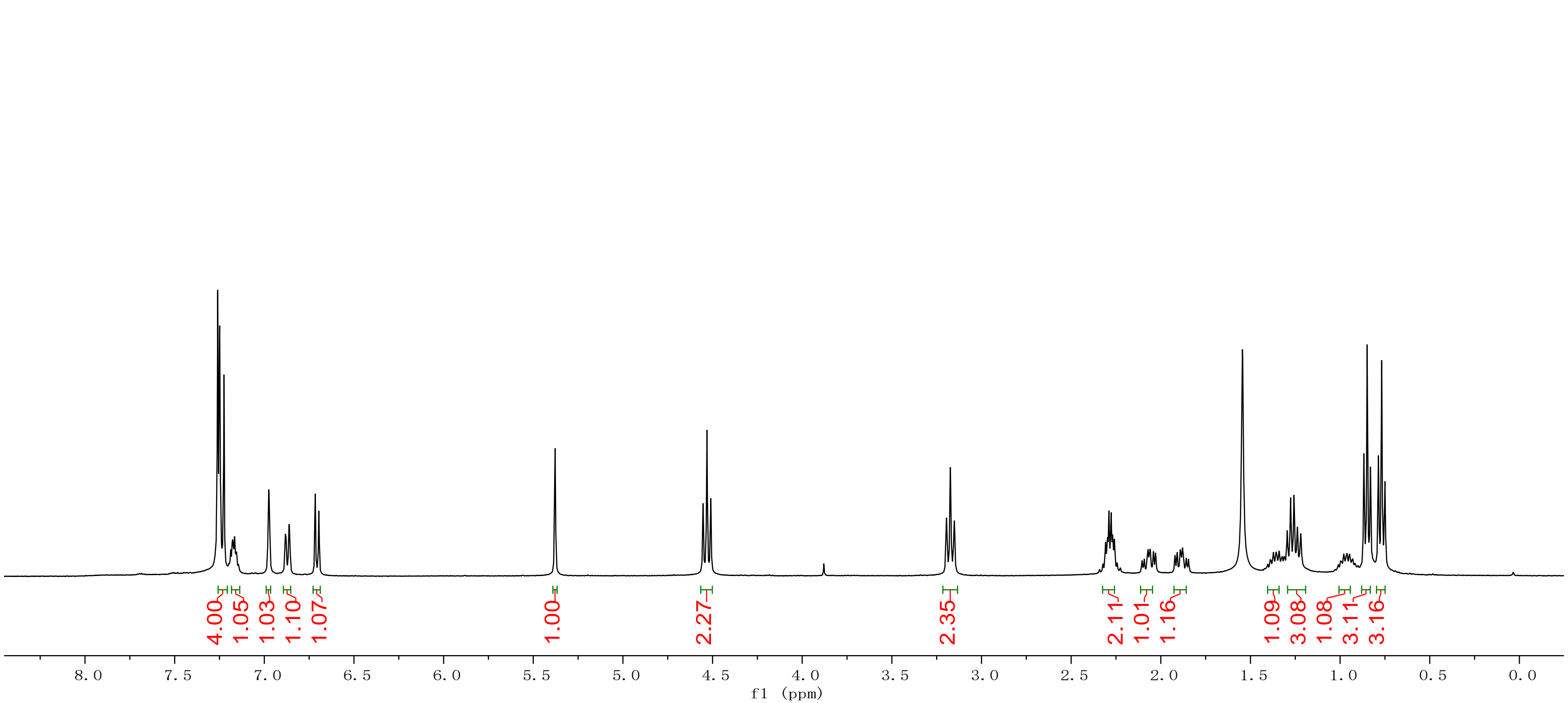

49: (*Z*)-3-(2,3-dihydrobenzofuran-5-yl)-1-phenyl-2-propylhex-2-en-1-ol (^{13}C NMR, $CDCl_3$, 100 MHz)

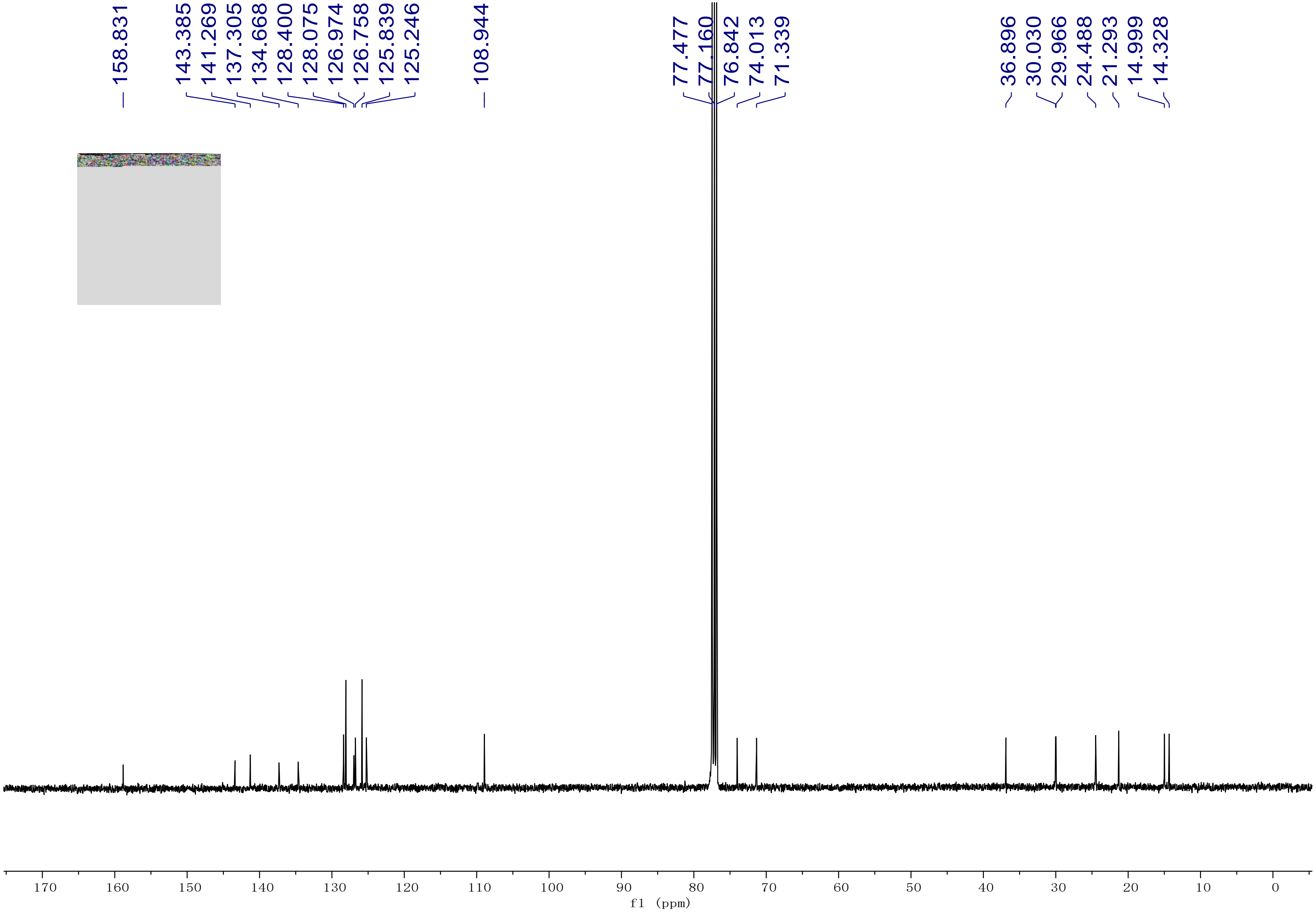

50: (Z)-3-(3,4-dimethoxyphenyl)-1-phenyl-2-propylhex-2-en-1-ol (^{1}H NMR, $CDCl_3$, 400 MHz)

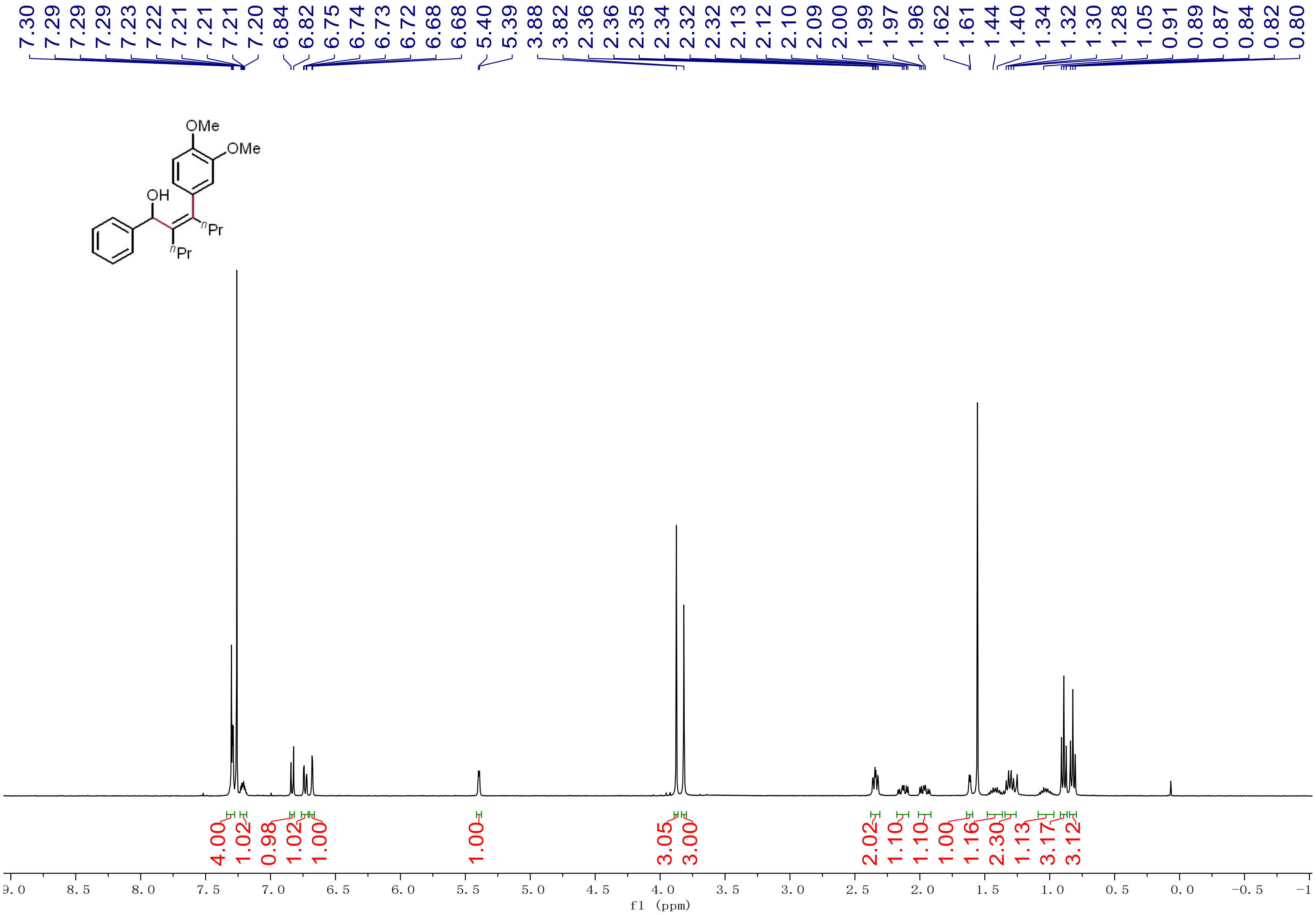

50: (Z)-3-(3,4-dimethoxyphenyl)-1-phenyl-2-propylhex-2-en-1-ol (^{13}C NMR, $CDCl_3$, 100 MHz)

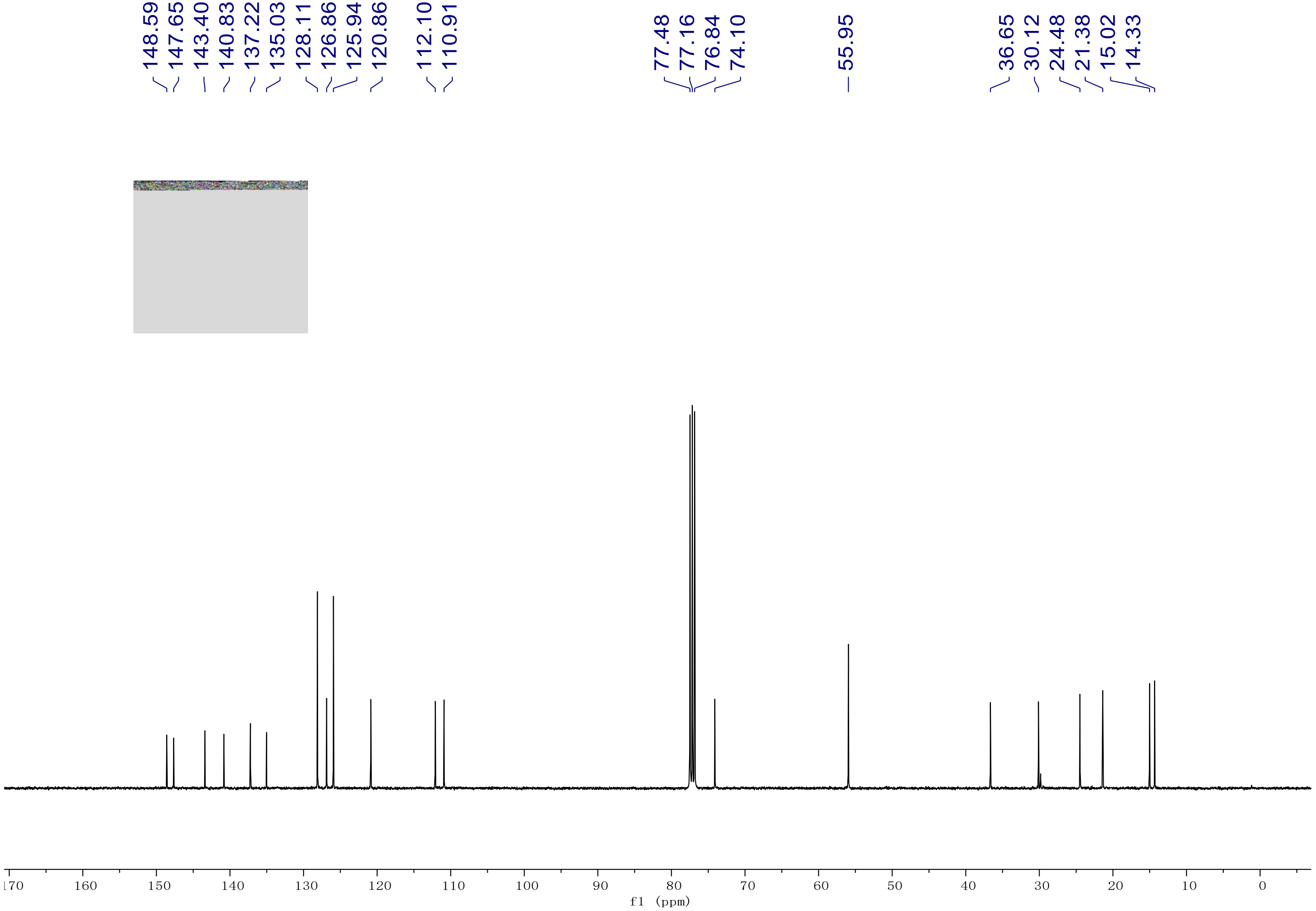

51: methyl (Z)-4-(3-(4-(benzyloxy)phenyl)-1-hydroxy-2-propylhex-2-en-1-yl)benzoate (^{1}H NMR, $CDCl_3$, 400 MHz)

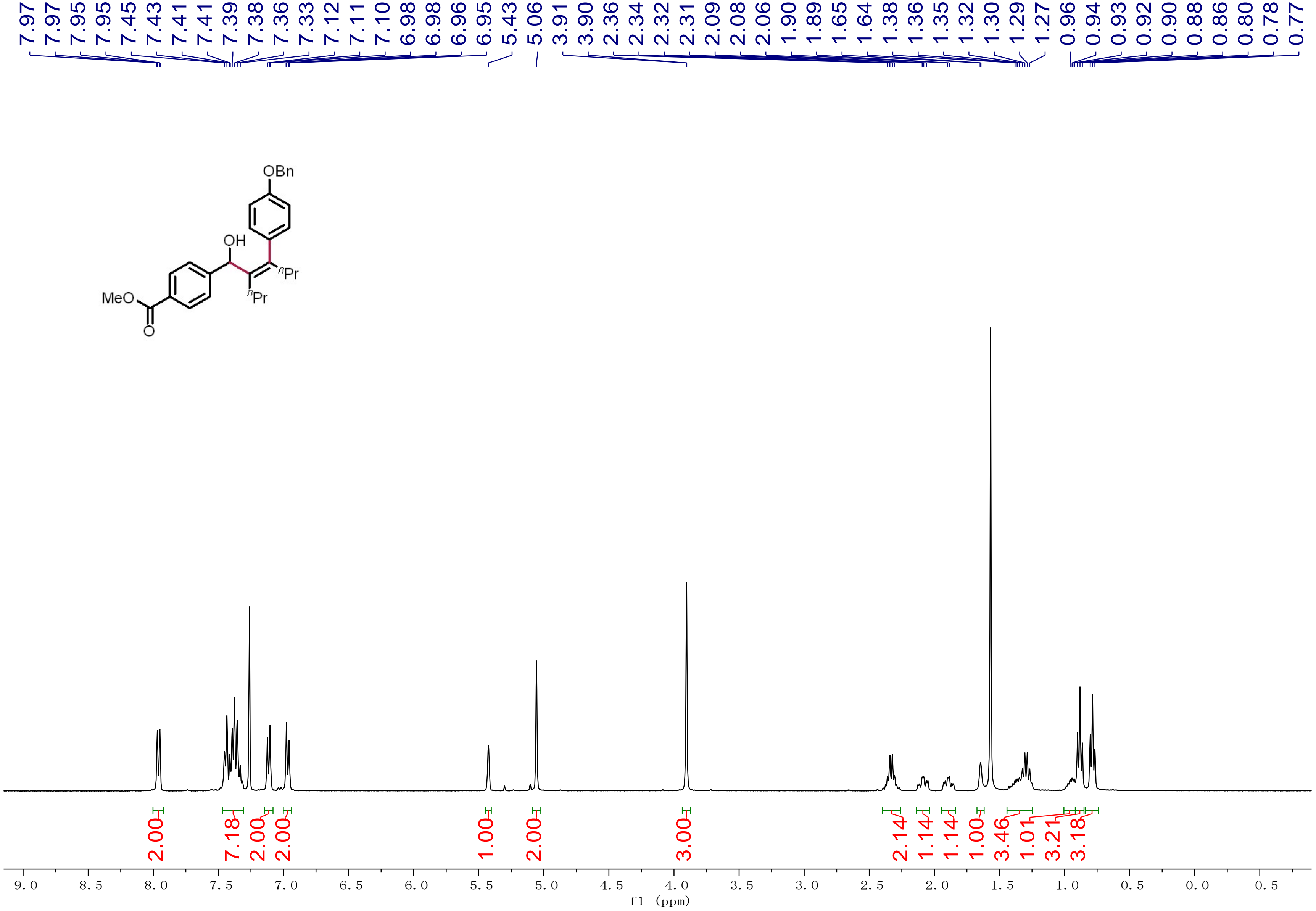

51: methyl (Z)-4-(3-(4-(benzyloxy)phenyl)-1-hydroxy-2-propylhex-2-en-1-yl)benzoate (^{13}C NMR, $CDCl_3$, 100 MHz)

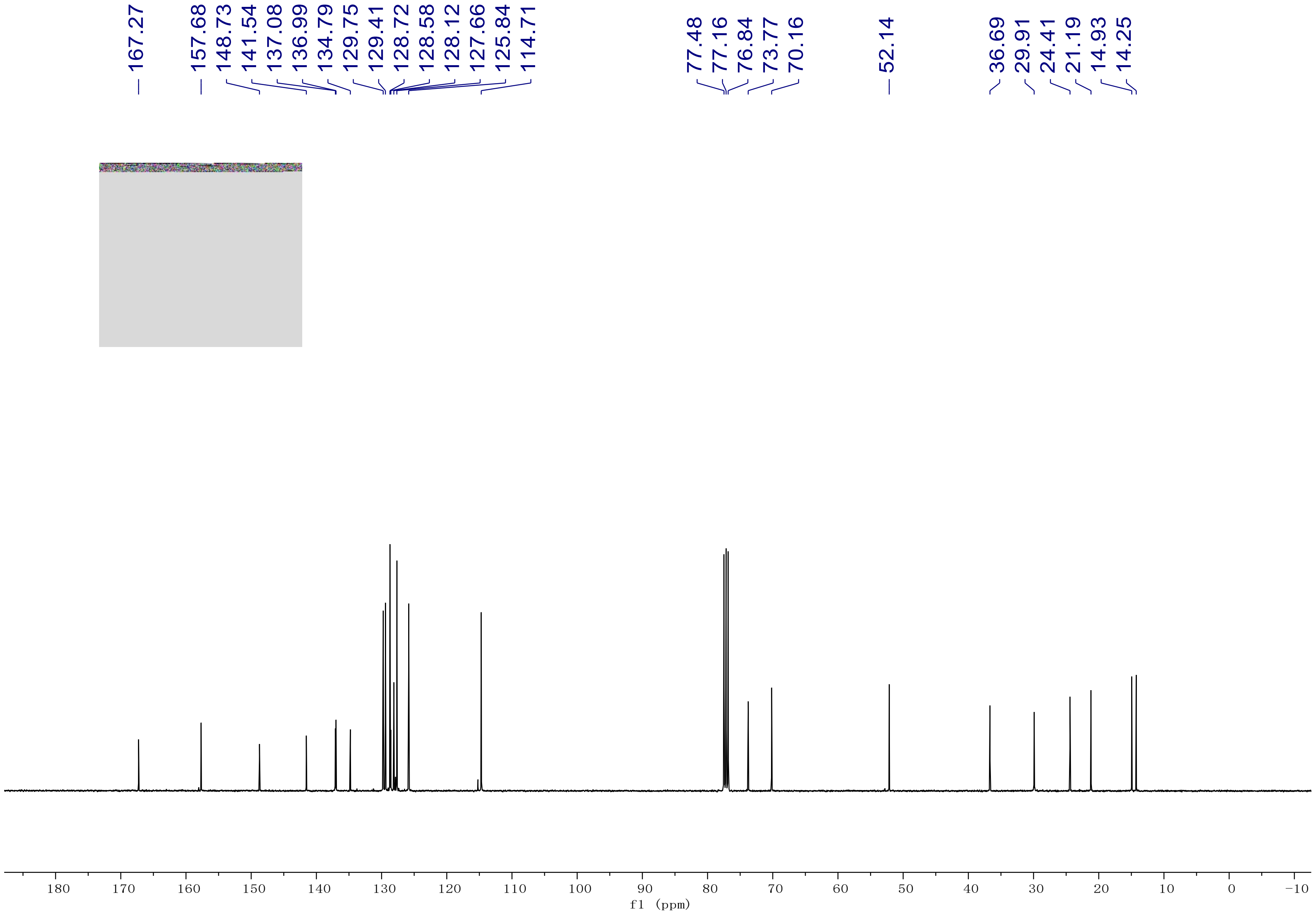

52: (Z)-3-(benzo[d][1,3]dioxol-5-yl)-2-propyl-1-(4-(trifluoromethyl)phenyl)hex-2-en-1-ol (^{1}H NMR, $CDCl_3$, 400 MHz)

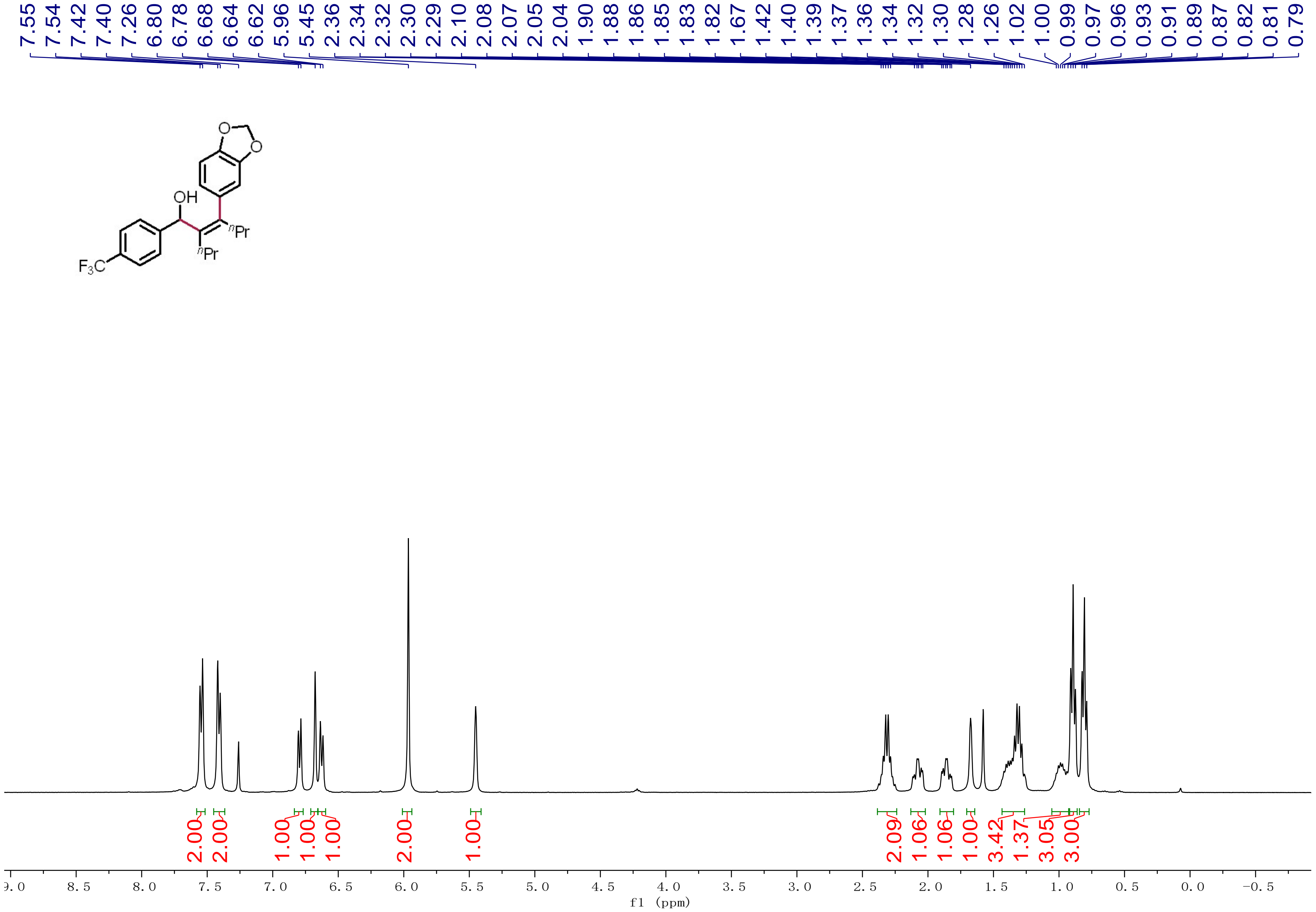

52: (Z)-3-(benzo[d][1,3]dioxol-5-yl)-2-propyl-1-(4-(trifluoromethyl)phenyl)hex-2-en-1-ol (^{13}C NMR, $CDCl_3$, 100 MHz)

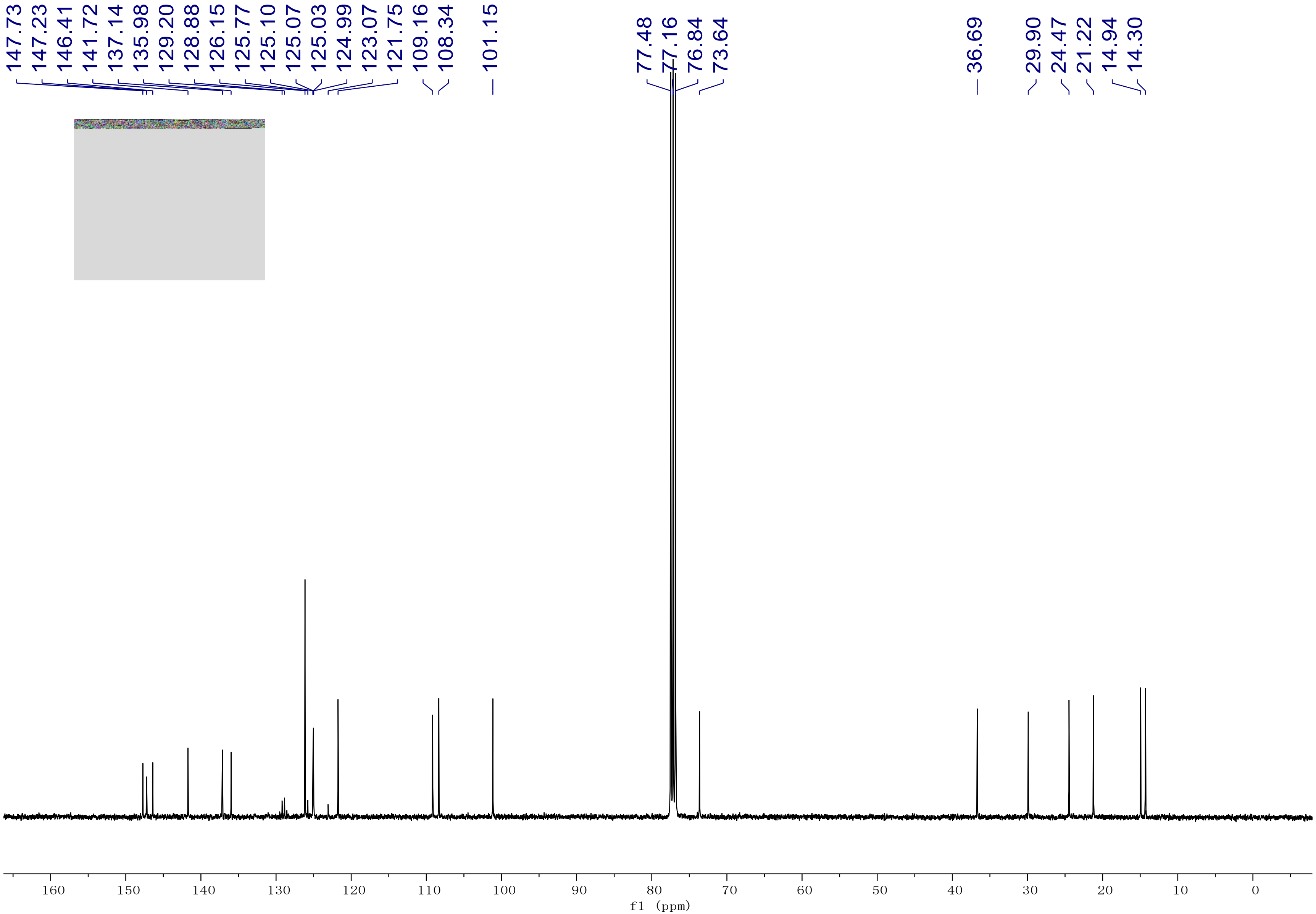

53: (*Z*)-1-(benzofuran-2-yl)-2-ethyl-3-(4-fluorophenyl)pent-2-en-1-ol (^{1}H NMR, $CDCl_3$, 400 MHz)

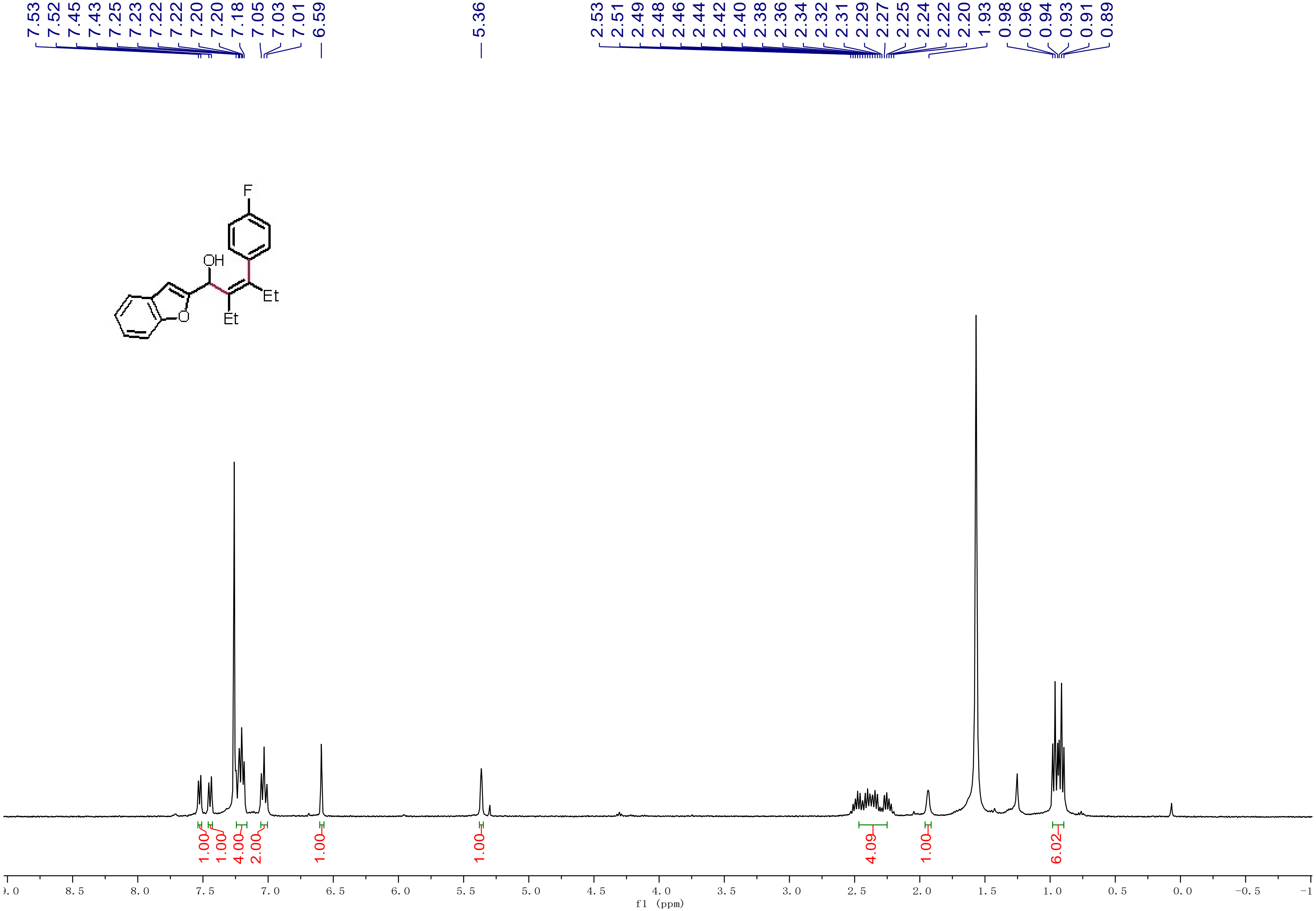

53: (*Z*)-1-(benzofuran-2-yl)-2-ethyl-3-(4-fluorophenyl)pent-2-en-1-ol (^{13}C NMR, $CDCl_3$, 100 MHz)

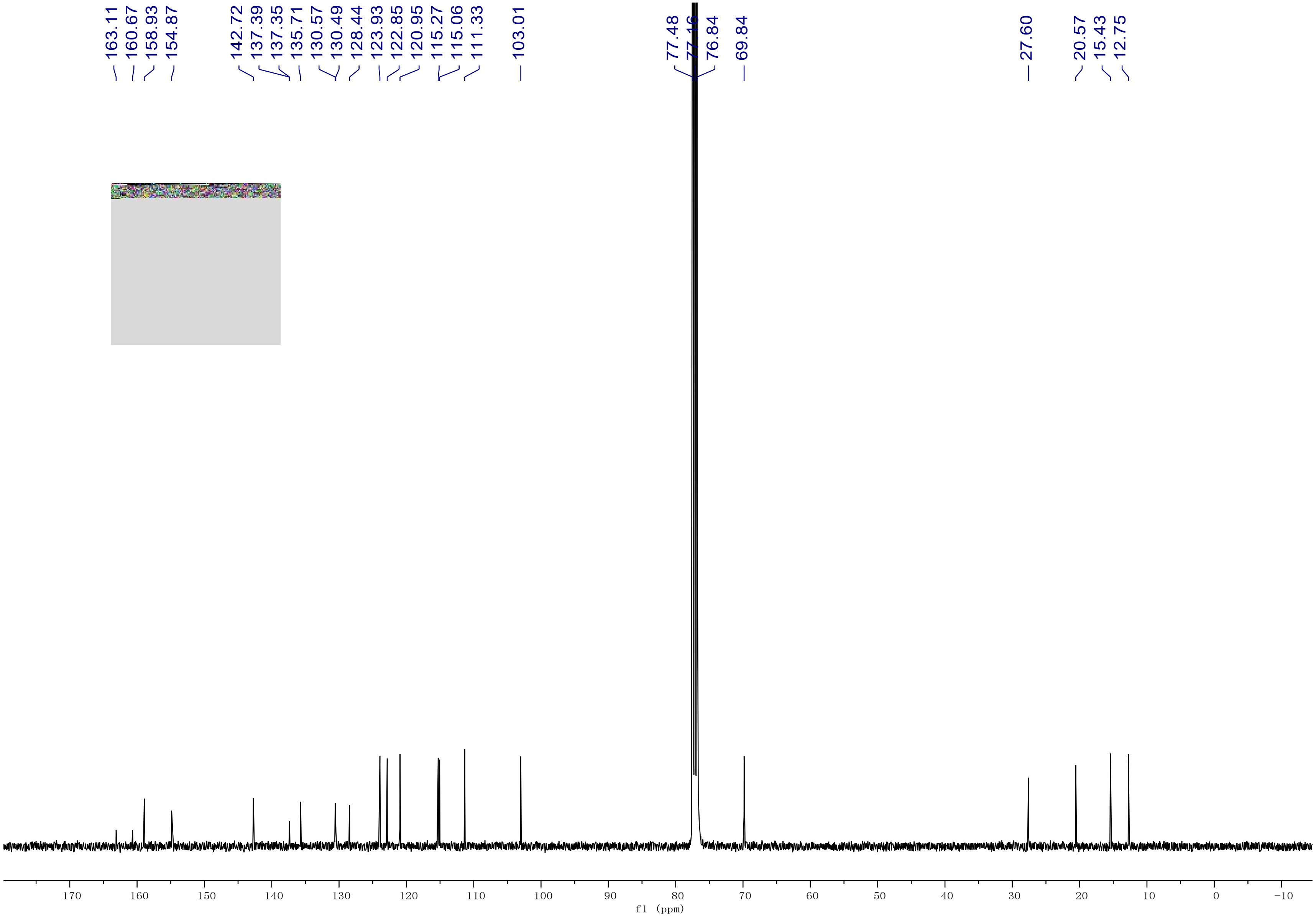

54: (Z)-3-(naphthalen-2-yl)-1-phenyl-2-propylhex-2-en-1-ol (^{1}H NMR, $CDCl_3$, 400 MHz)

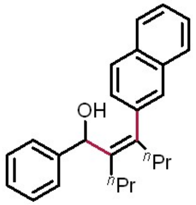

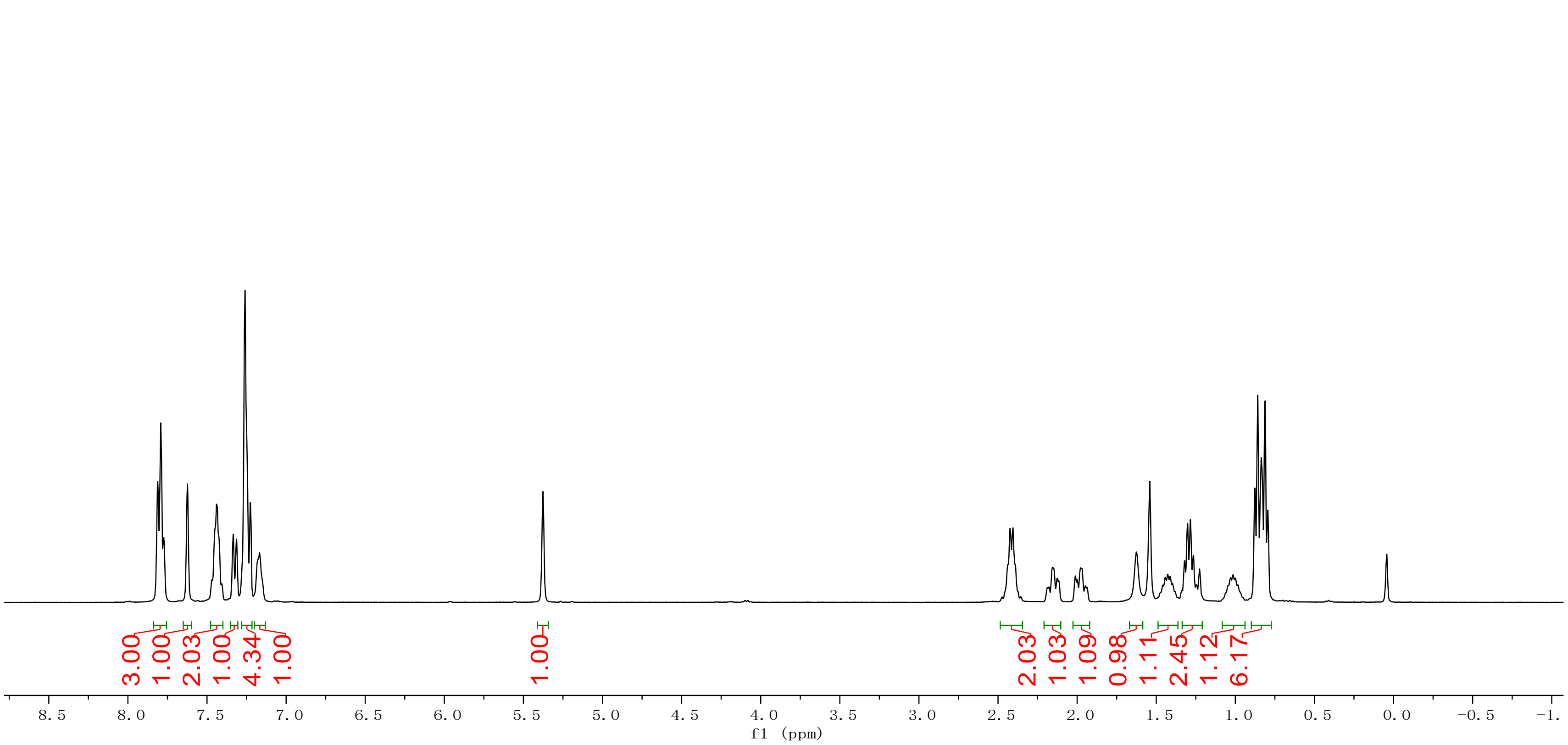

54: (Z)-3-(naphthalen-2-yl)-1-phenyl-2-propylhex-2-en-1-ol (^{13}C NMR, $CDCl_3$, 100 MHz)

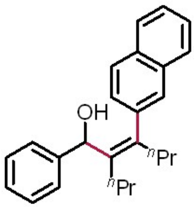

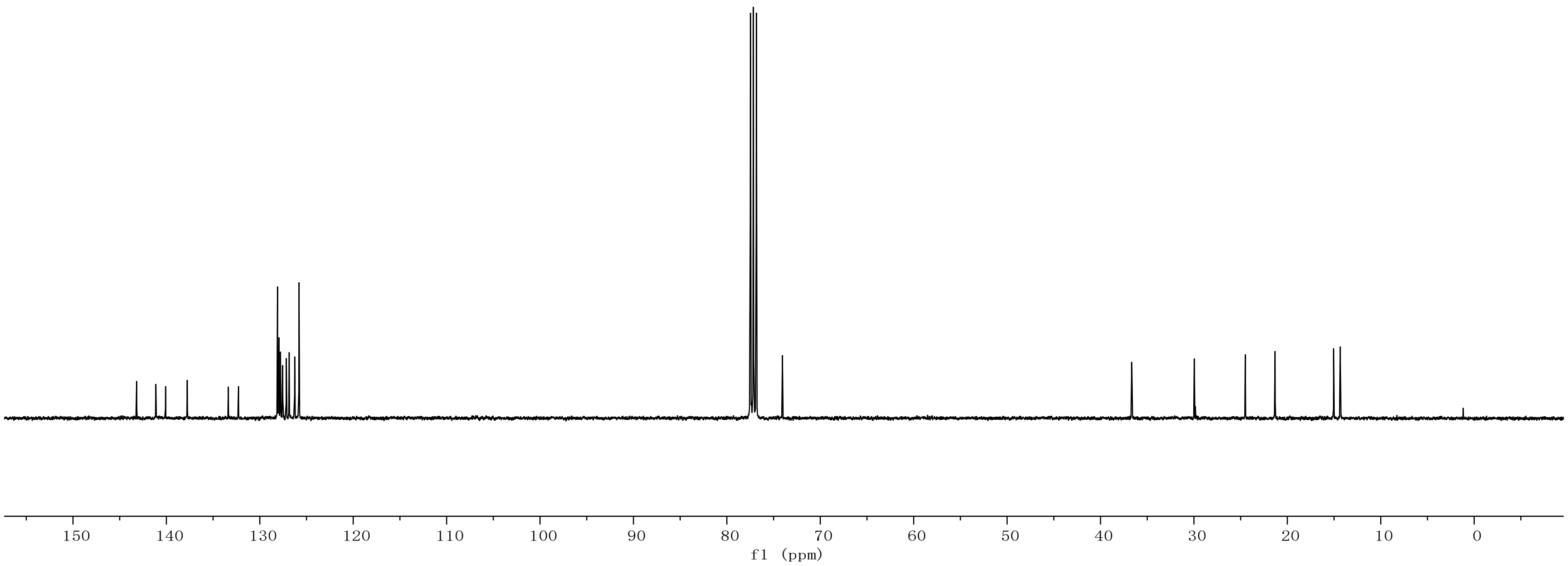

55: (*Z*)-3-(1-methyl-1H-indol-5-yl)-1-phenyl-2-propylhex-2-en-1-ol (^{1}H NMR, $CDCl_3$, 400 MHz)

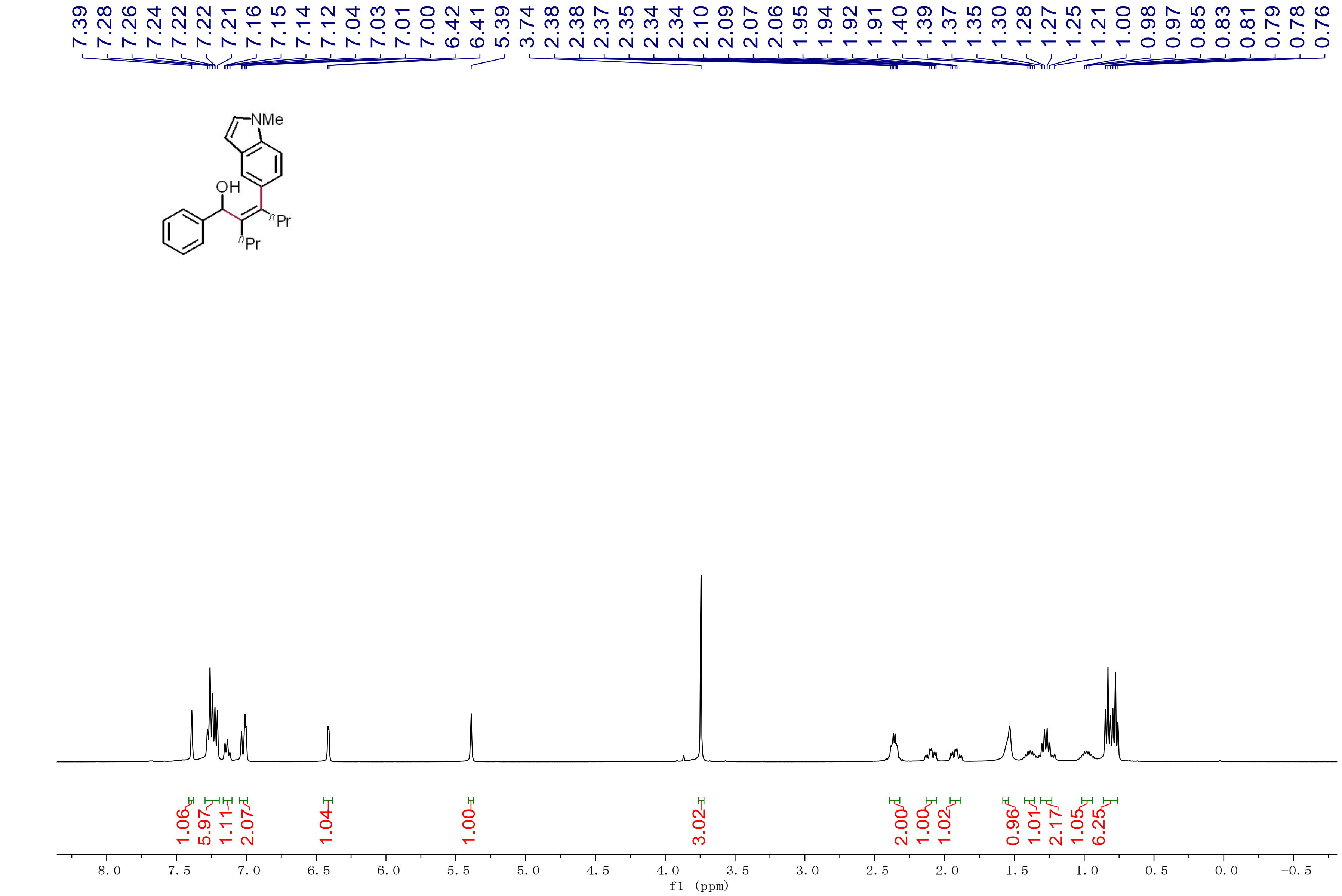

55: (*Z*)-3-(1-methyl-1H-indol-5-yl)-1-phenyl-2-propylhex-2-en-1-ol (^{13}C NMR, $CDCl_3$, 100 MHz)

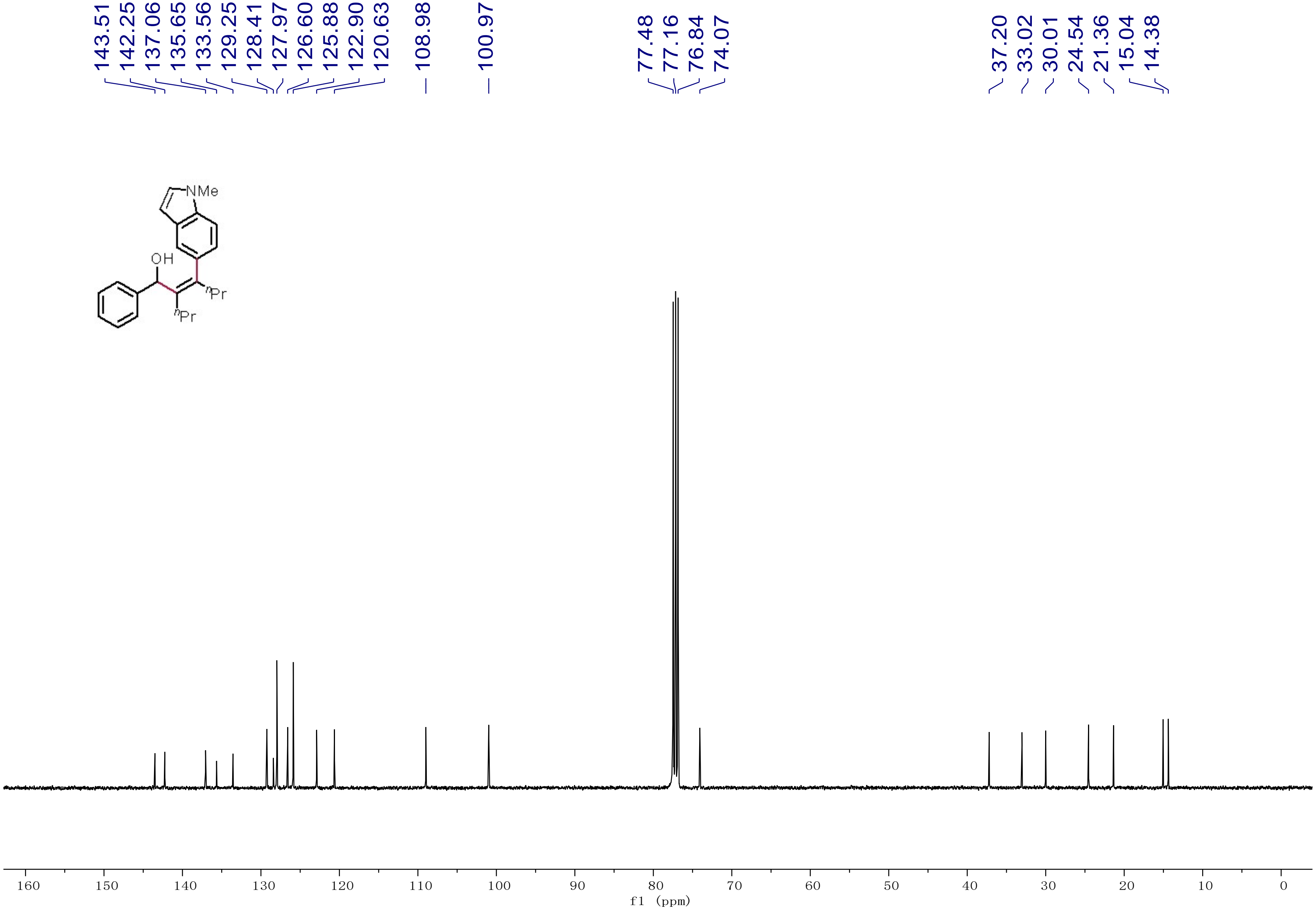

56: (Z)-3-(benzofuran-5-yl)-1-phenyl-2-propylhex-2-en-1-ol (^{1}H NMR, $CDCl_3$, 400 MHz)

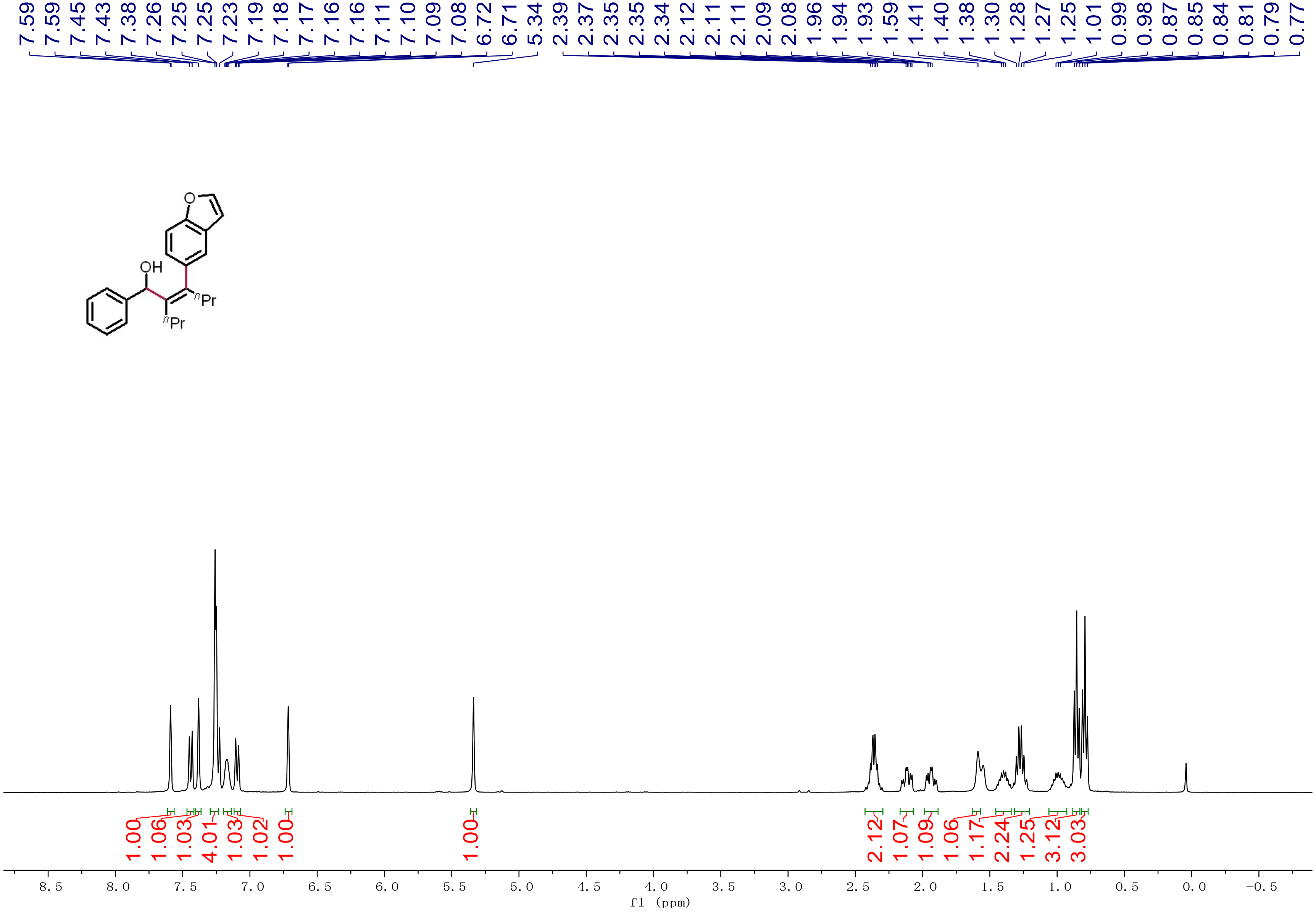

56: (Z)-3-(benzofuran-5-yl)-1-phenyl-2-propylhex-2-en-1-ol (^{13}C NMR, $CDCl_3$, 100 MHz)

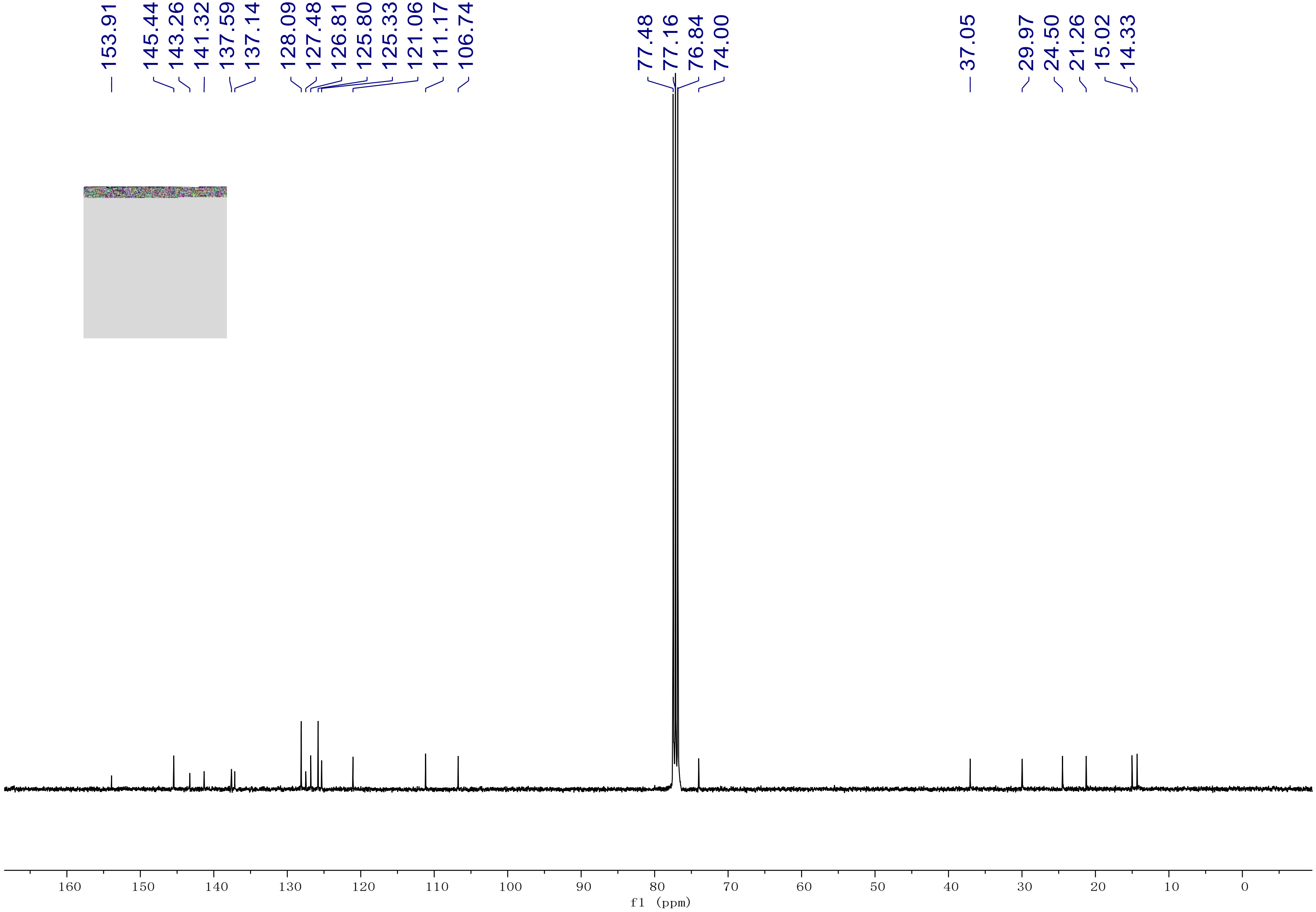

57: (Z)-3-(benzo[b]thiophen-2-yl)-1-phenyl-2-propylhex-2-en-1-ol (^{1}H NMR, $CDCl_3$, 400 MHz)

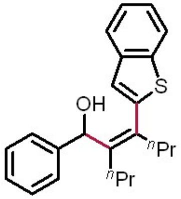

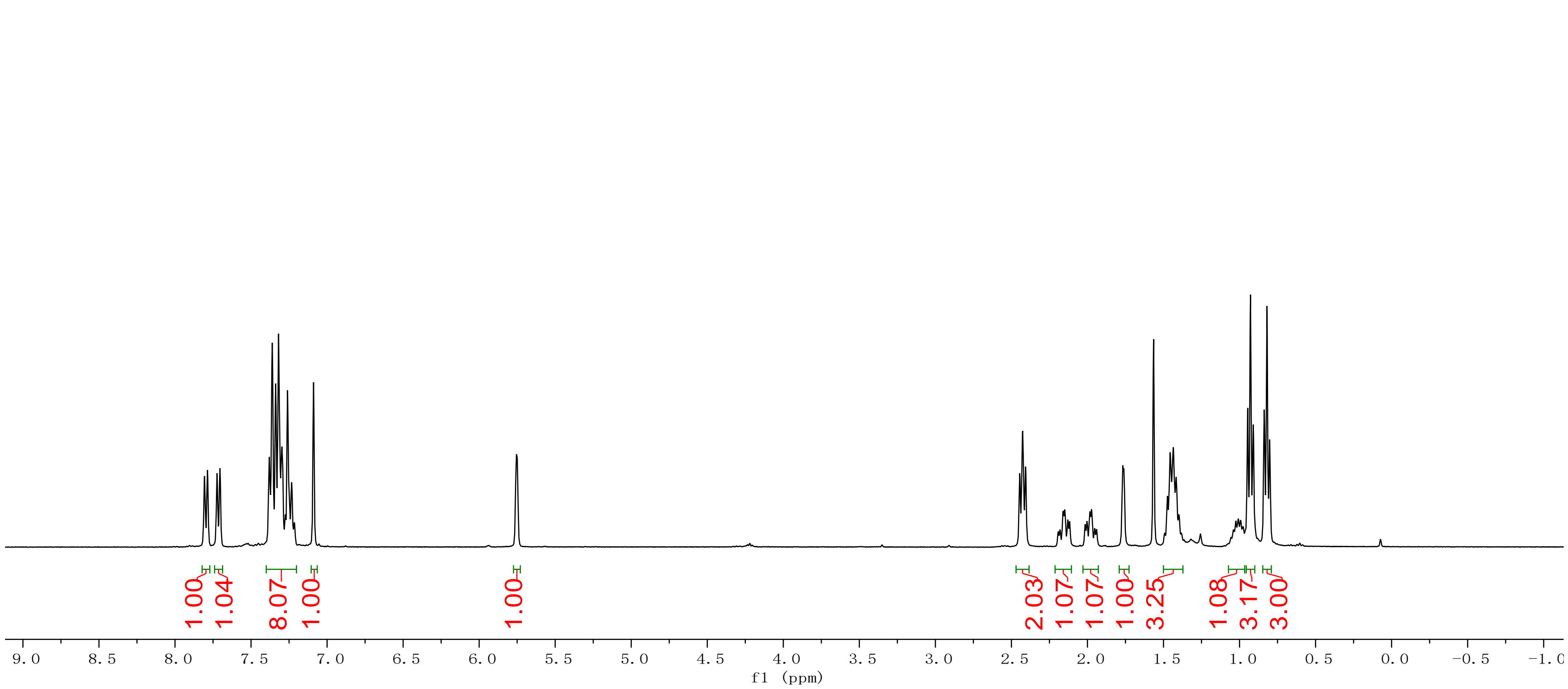

57: (Z)-3-(benzo[b]thiophen-2-yl)-1-phenyl-2-propylhex-2-en-1-ol (^{13}C NMR, $CDCl_3$, 100 MHz)

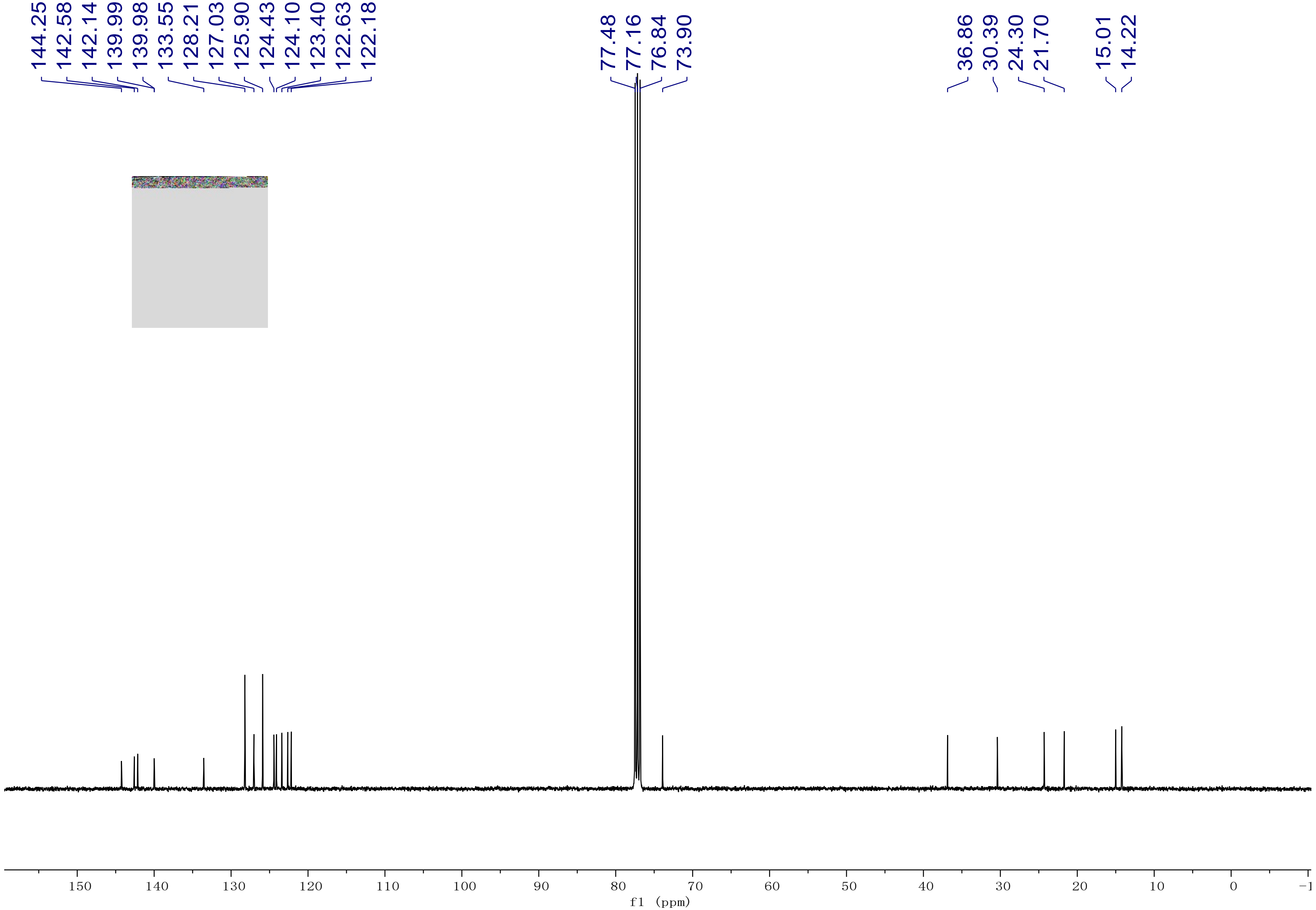

58: (Z)-2-butyl-3-(furan-3-yl)-1-phenylhept-2-en-1-ol (^{1}H NMR, $CDCl_3$, 400 MHz)

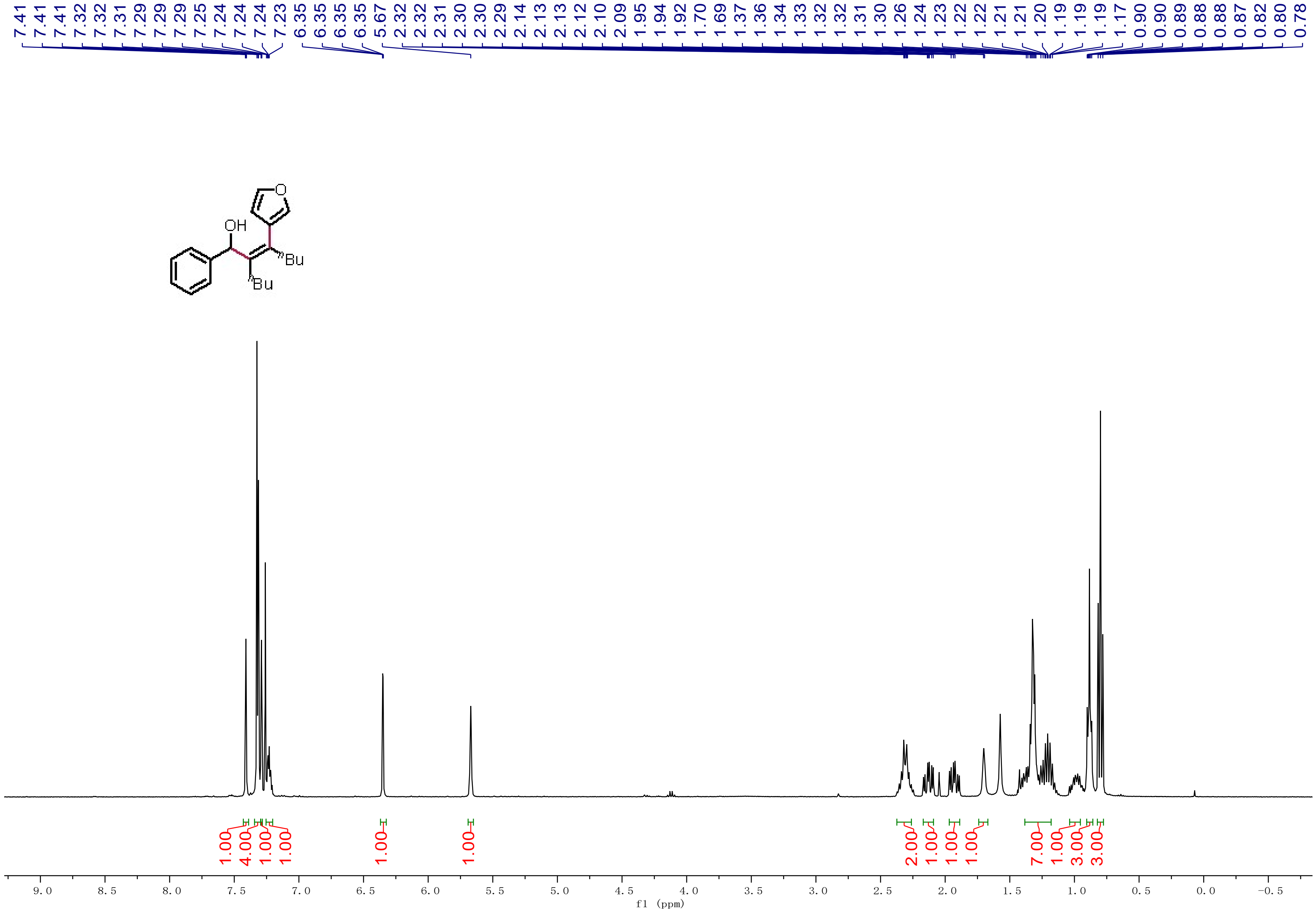

58: (Z)-2-butyl-3-(furan-3-yl)-1-phenylhept-2-en-1-ol (^{13}C NMR, $CDCl_3$, 100 MHz)

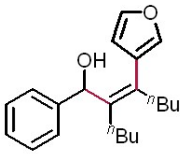

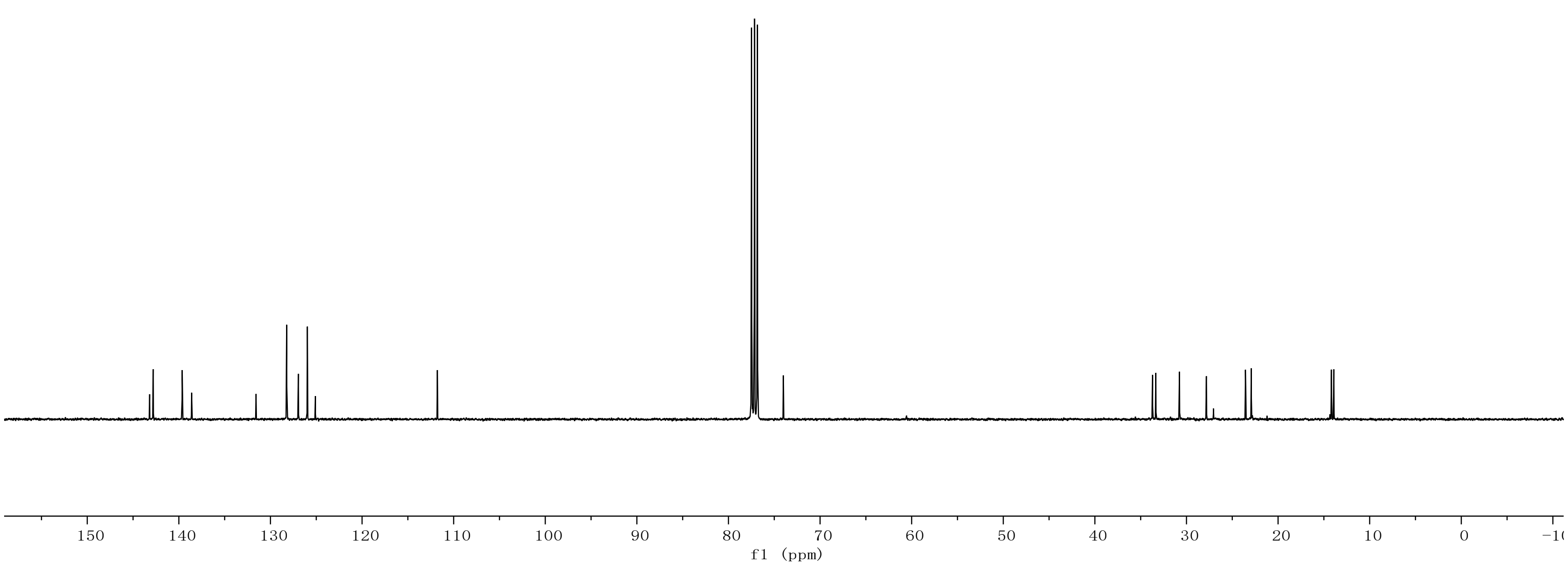

59: (Z)-2-butyl-1-phenyl-3-(thiophen-3-yl)hept-2-en-1-ol (^{1}H NMR, $CDCl_3$, 400 MHz)

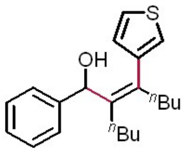

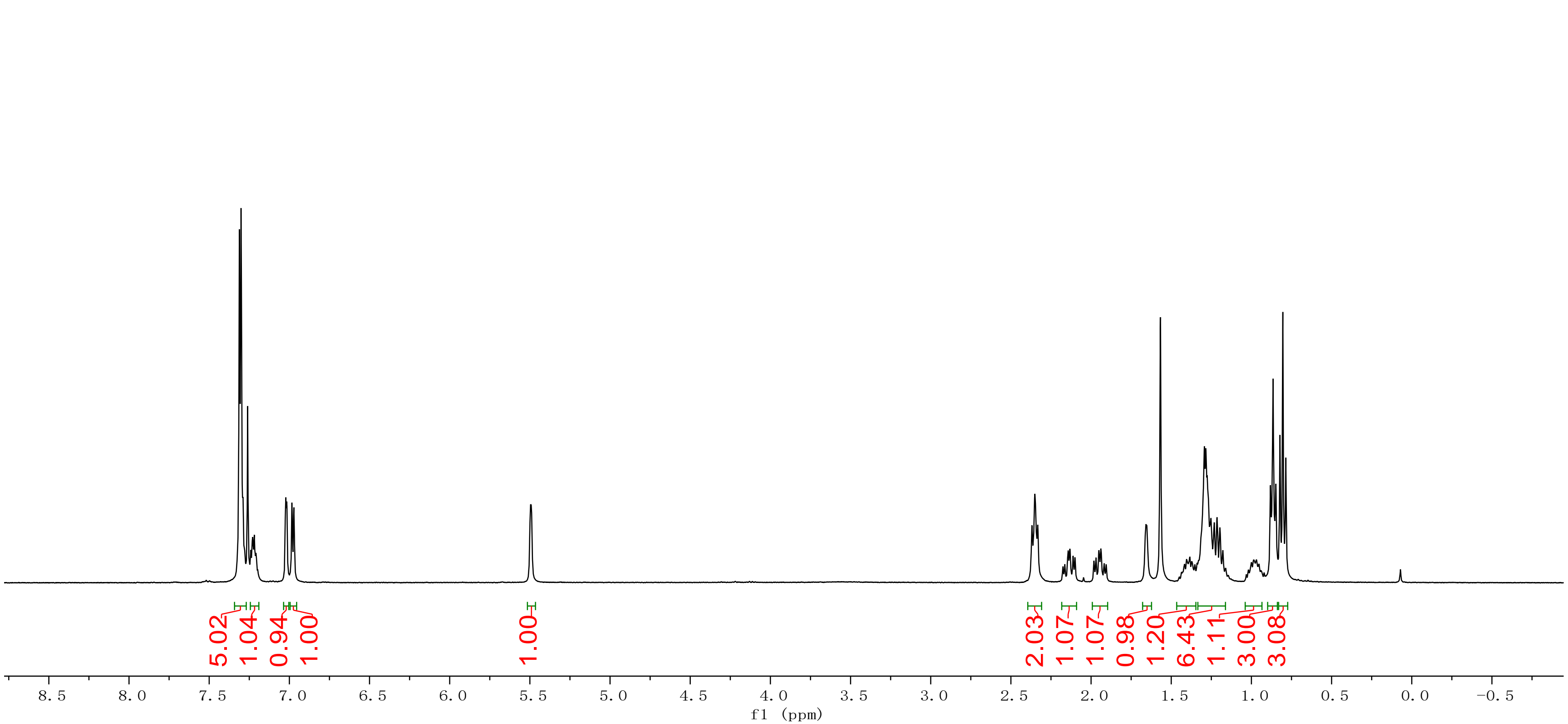

59: (Z)-2-butyl-1-phenyl-3-(thiophen-3-yl)hept-2-en-1-ol (^{13}C NMR, $CDCl_3$, 100 MHz)

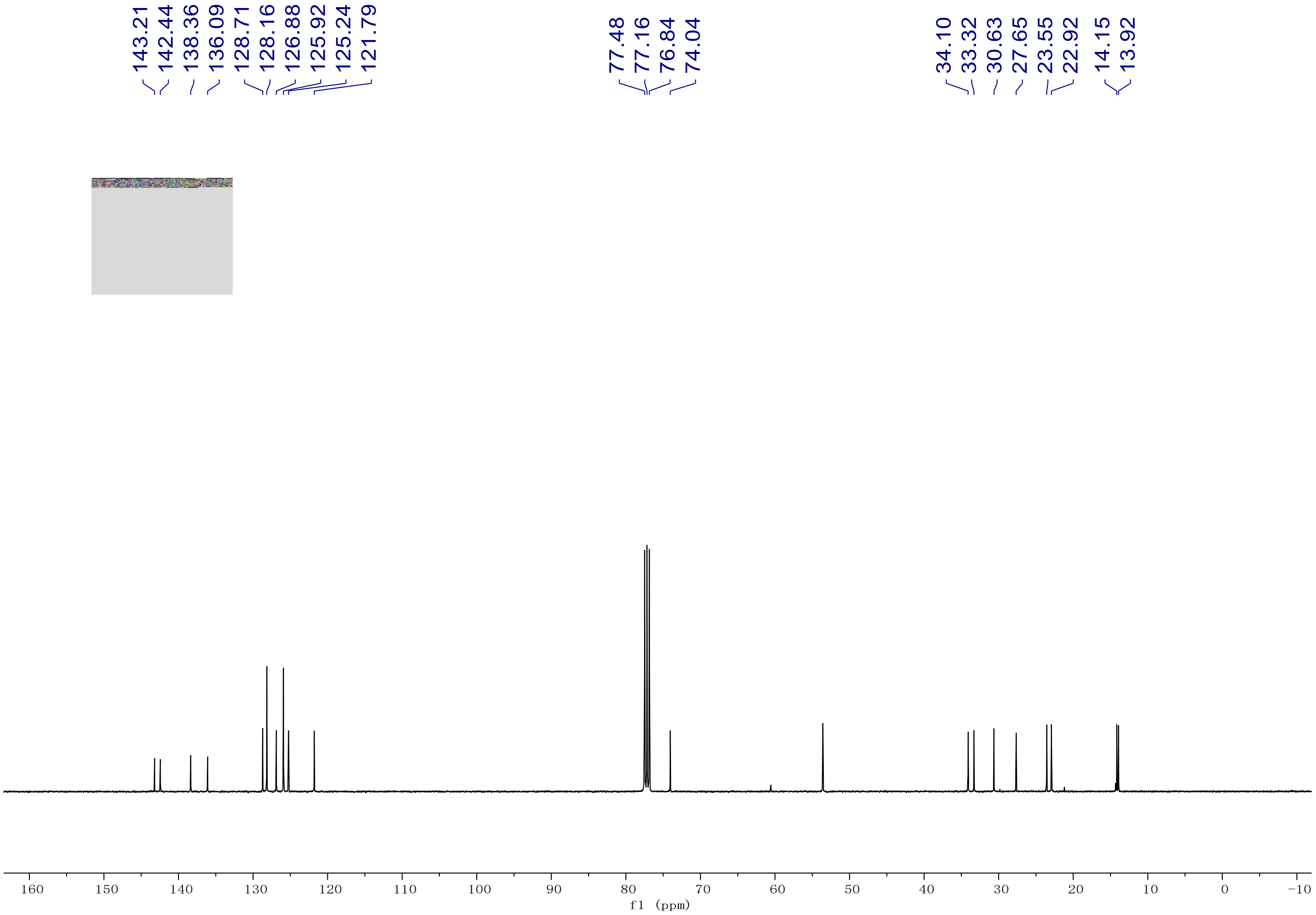

60:(*Z*)-1-(6-(3-((3r,5r,7r)-adamantan-1-yl)-4-methoxyphenyl)naphthalen-2-yl)-2-butyl-3-(4-methoxyphenyl)hept-2-en-1-ol (^{1}H NMR, $CDCl_3$, 400 MHz)

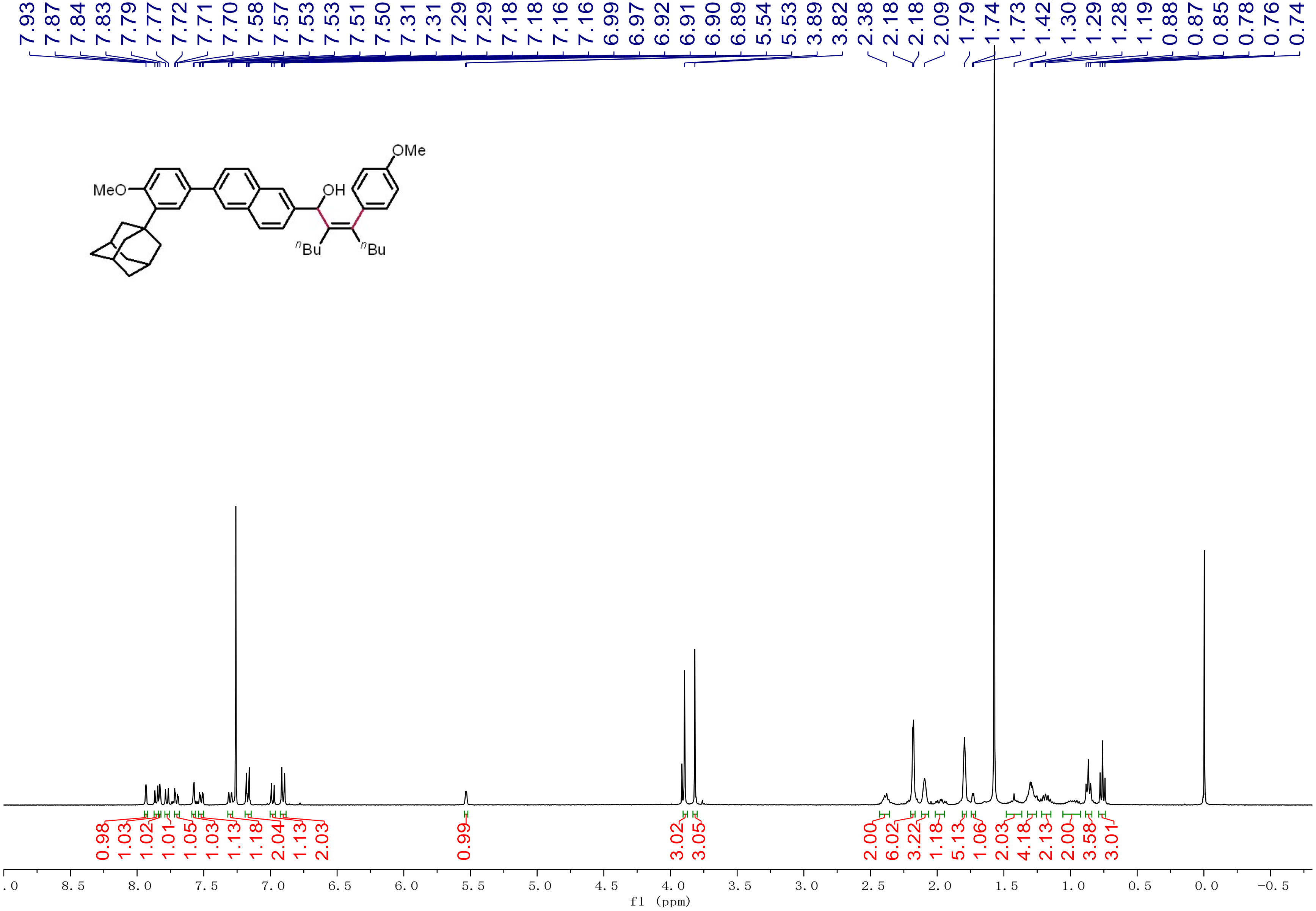

60:(*Z*)-1-(6-(3-((3r,5r,7r)-adamantan-1-yl)-4-methoxyphenyl)naphthalen-2-yl)-2-butyl-3-(4-methoxyphenyl)hept-2-en-1-ol (^{13}C NMR, $CDCl_3$, 100 MHz)

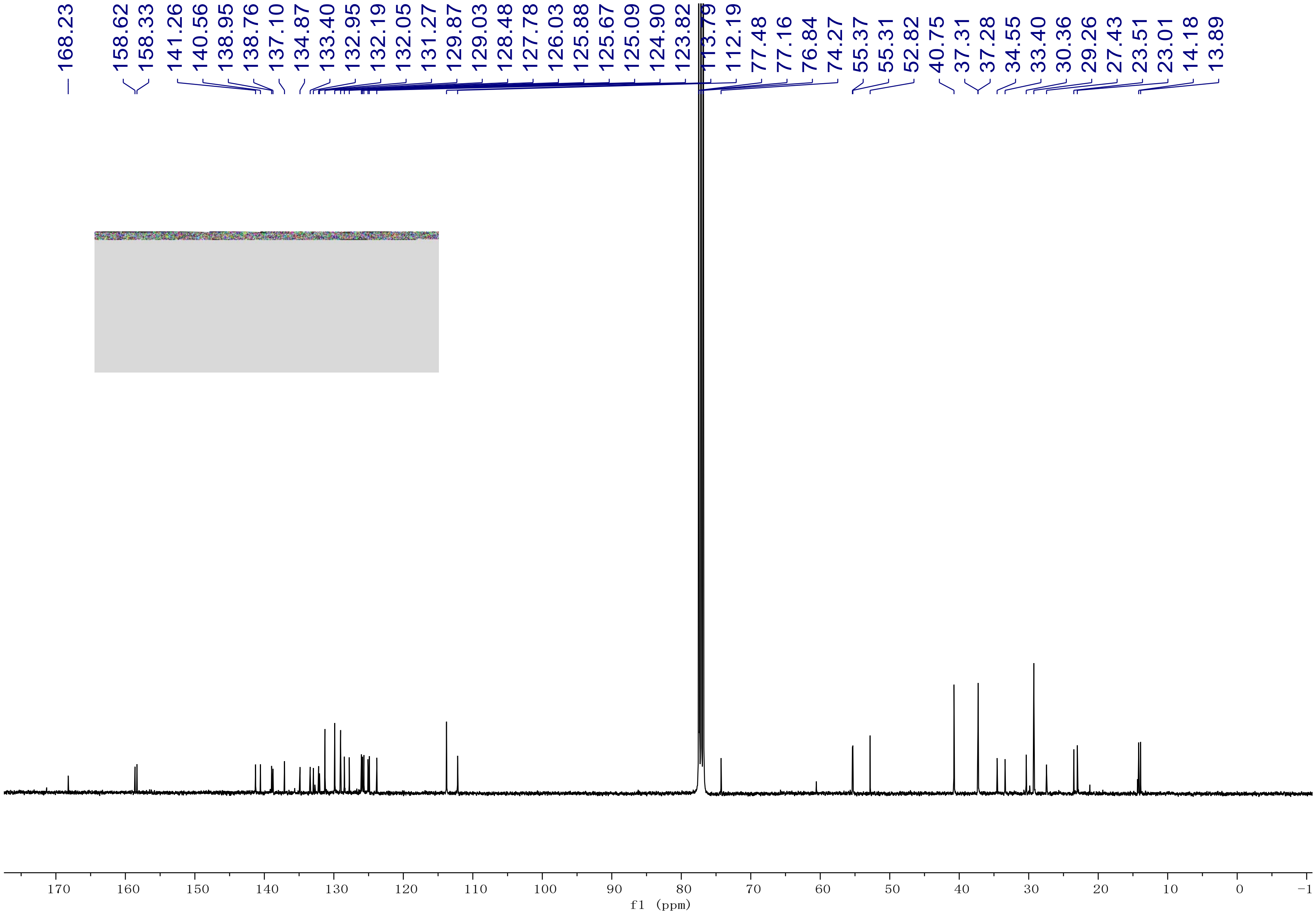

61: (3*S*,8*S*,9*S*,10*R*,13*R*,14*S*,17*R*)-10,13-dimethyl-17-((*R*)-6-methylheptan-2-yl)- 2,3,4,7,8,9,10,11,12,13,14,15,16,17-tetradecahydro-1H-cyclopenta[a]phenanthren-3-yl 4-((Z)-2-ethyl-1-hydroxy-3-(4-methoxyphenyl)pent-2-en-1-yl)benzoate (^{1}H NMR, $CDCl_3$, 400 MHz)

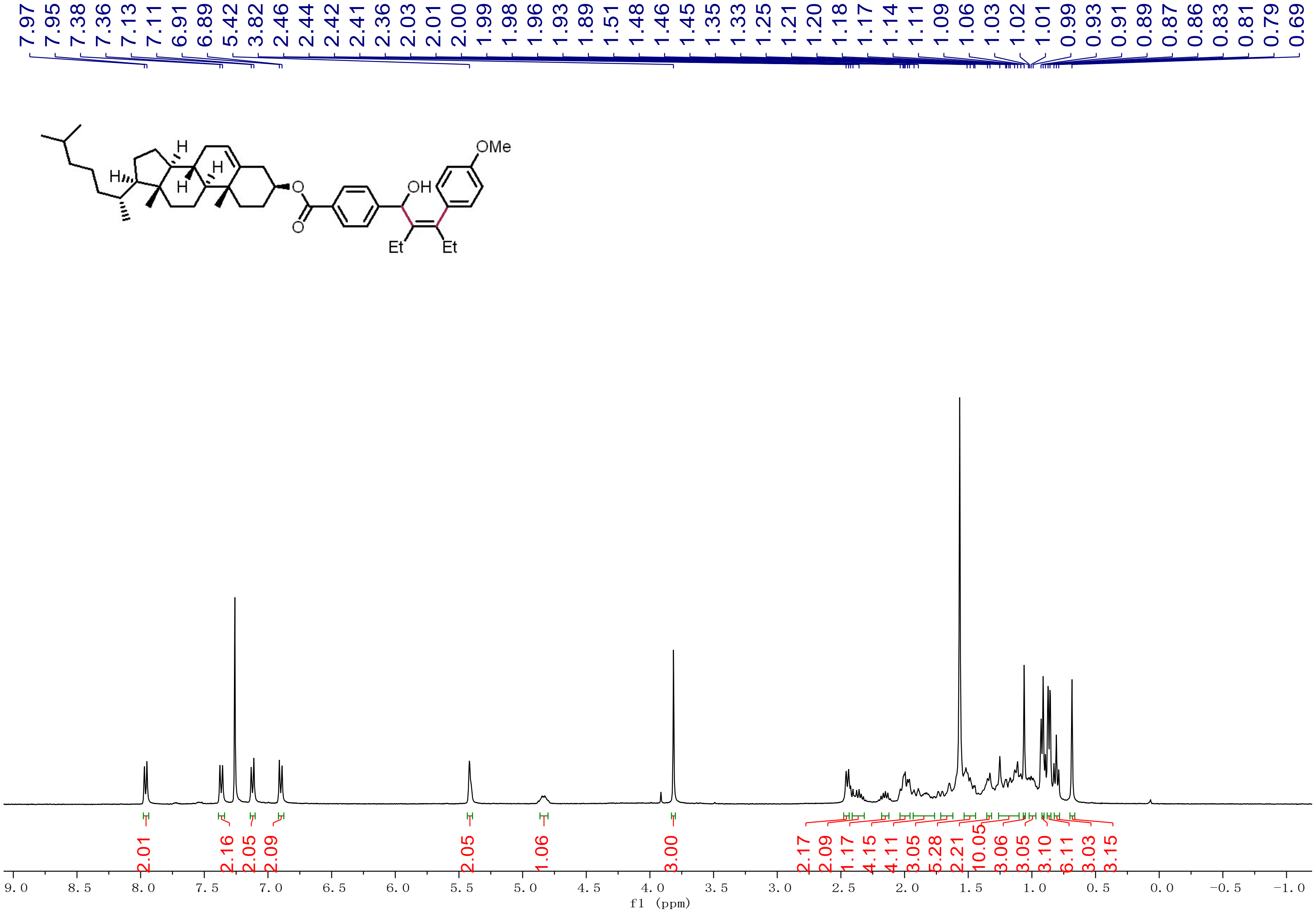

61: (3*S*,8*S*,9*S*,10*R*,13*R*,14*S*,17*R*)-10,13-dimethyl-17-((*R*)-6-methylheptan-2-yl)- 2,3,4,7,8,9,10,11,12,13,14,15,16,17-tetradecahydro-1H-cyclopenta[a]phenanthren-3-yl 4-((Z)-2-ethyl-1-hydroxy-3-(4-methoxyphenyl)pent-2-en-1-yl)benzoate (^{13}C NMR, $CDCl_3$, 100 MHz)

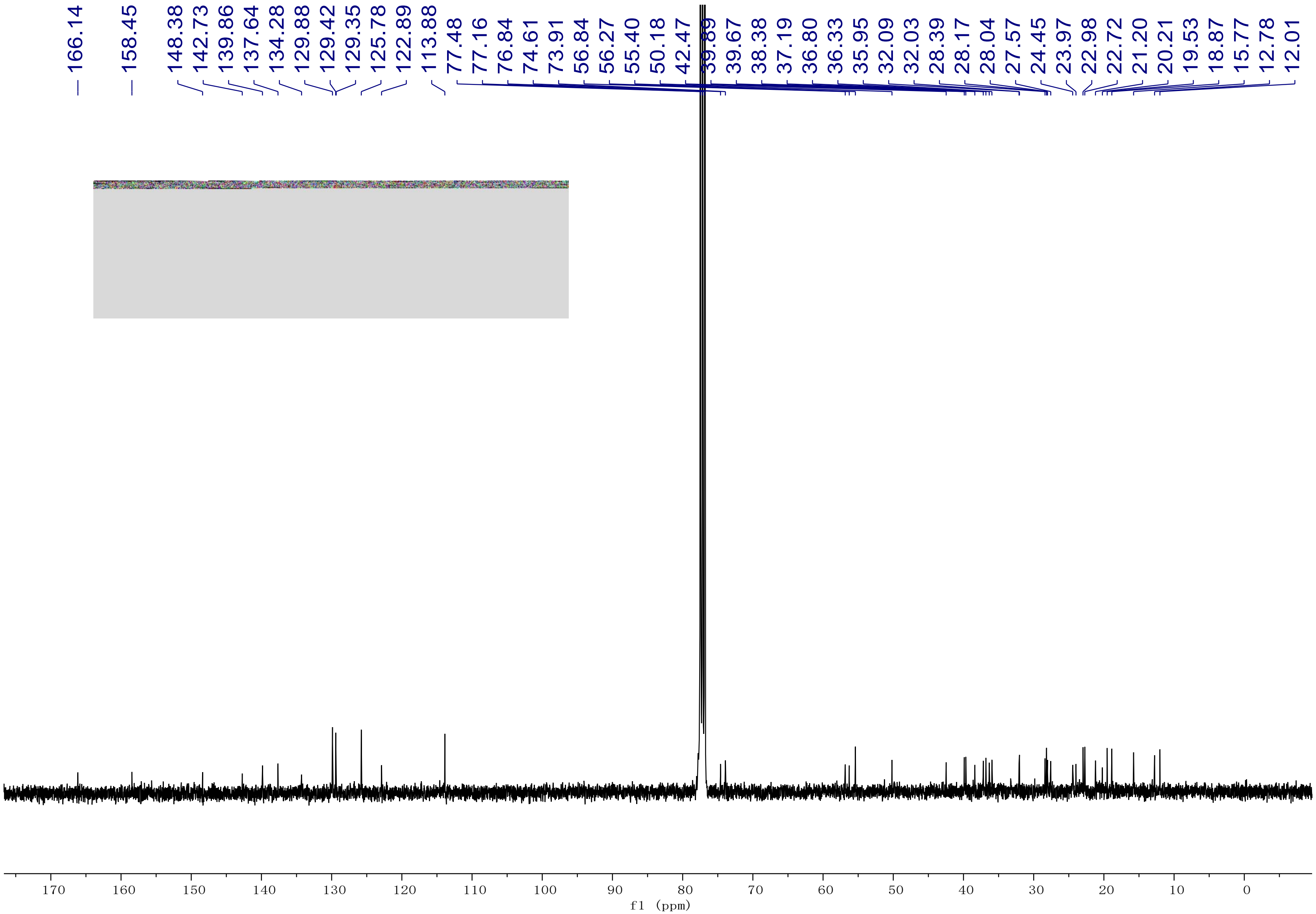

62: (3*S*,8*S*,9*S*,10*R*,13*R*,14*S*,17*R*)-10,13-dimethyl-17-((*R*)-6-methylheptan-2-yl)-2,3,4,7,8,9,10,11,12,13,14,15,16,17-tetradecahydro-1H-cyclopenta[a]phenanthren-3-yl 4-((Z)-2-ethyl-1-hydroxy-3-(4-methoxyphenyl)pent-2-en-1-yl)benzoate (^{1}H NMR, $CDCl_3$, 400 MHz)

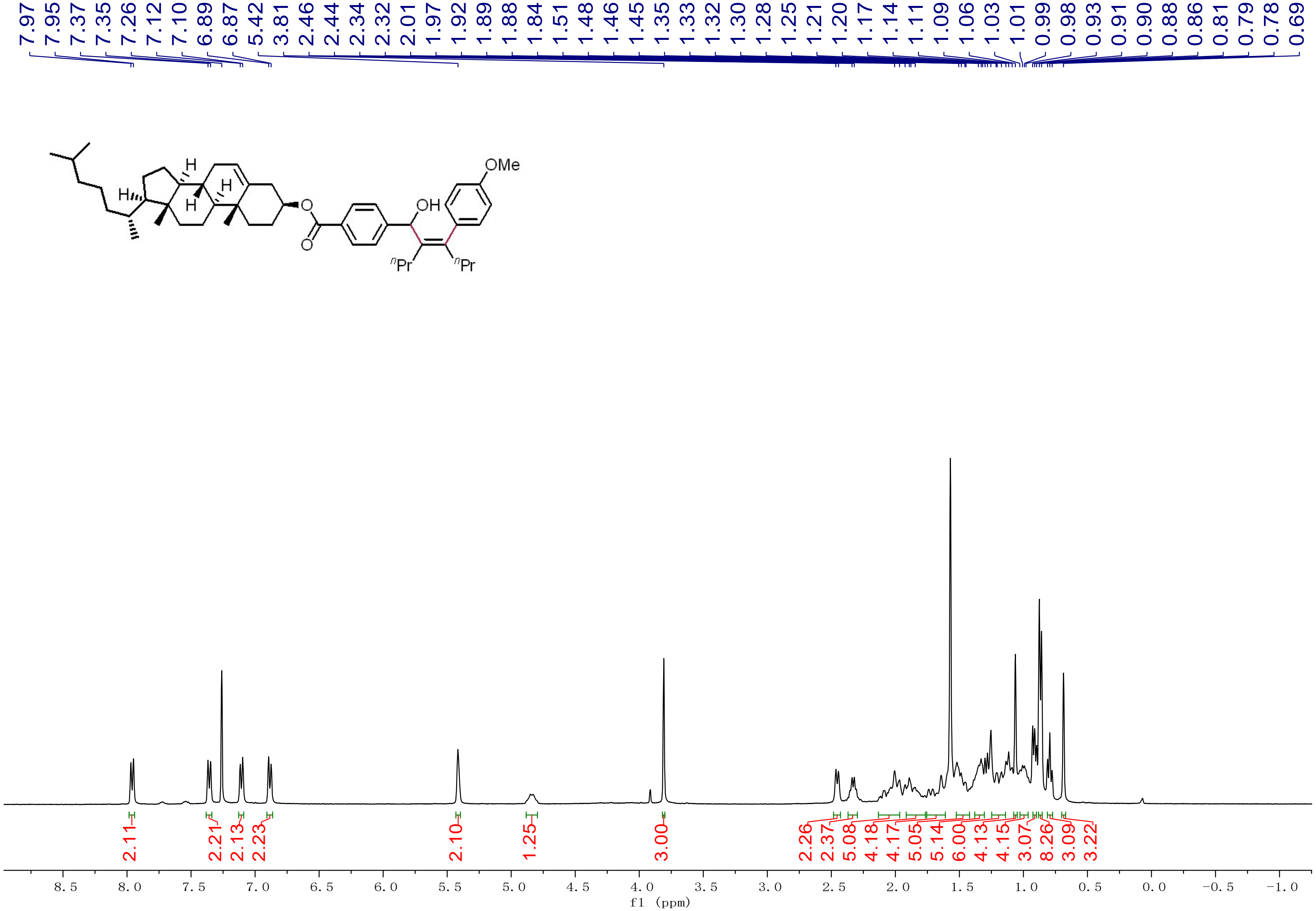

62: (3S,8S,9S,10R,13R,14S,17R)-10,13-dimethyl-17-((R)-6-methylheptan-2-yl)-2,3,4,7,8,9,10,11,12,13,14,15,16,17-tetradecahydro-1H-cyclopenta[a]phenanthren-3-yl 4-((Z)-2-ethyl-1-hydroxy-3-(4-methoxyphenyl)pent-2-en-1-yl)benzoate (^{13}C NMR, $CDCl_3$, 100 MHz)

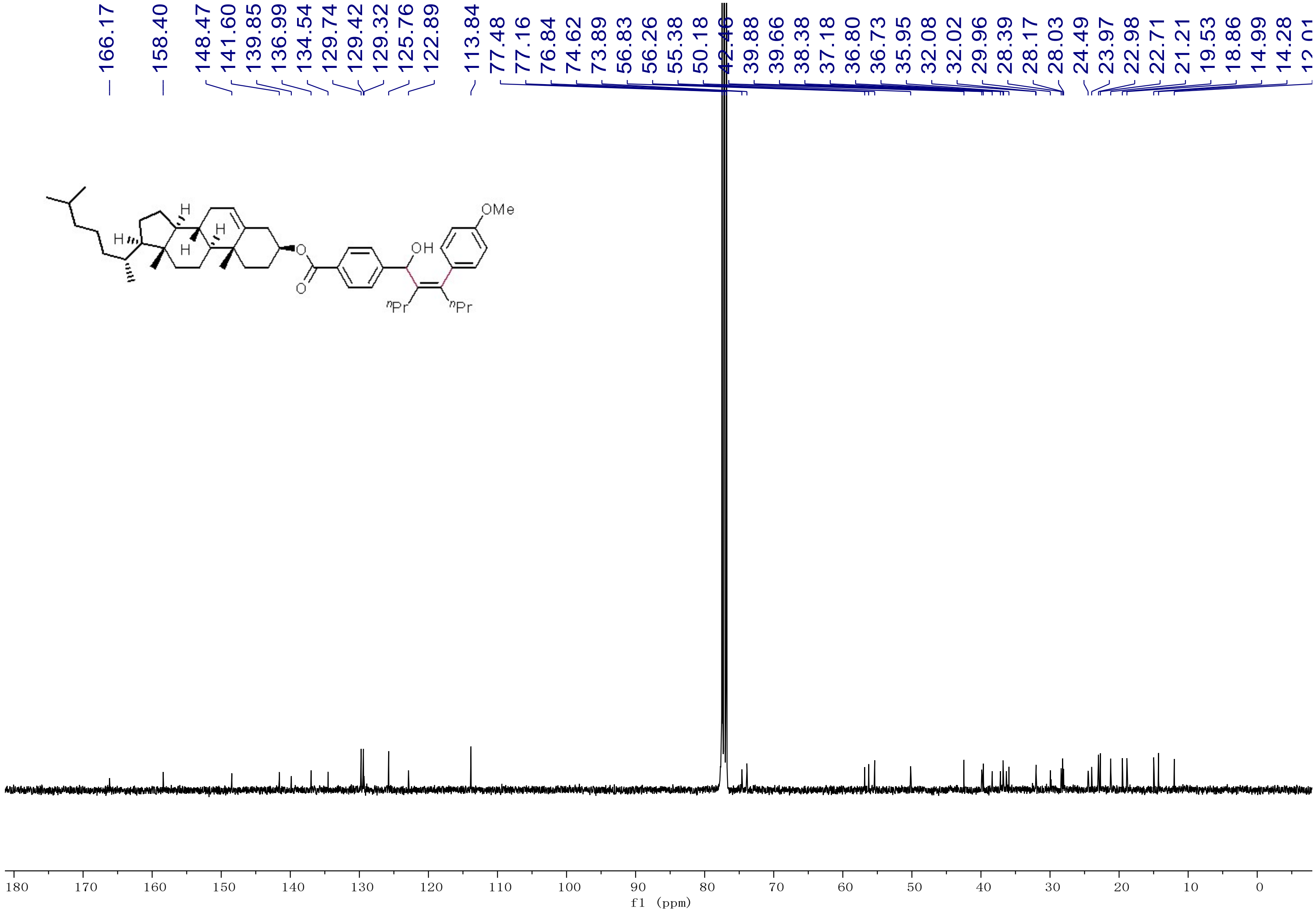

63: (8S,9R,13R,14R)-3-((Z)-2-ethyl-1-hydroxy-3-(4-methoxyphenyl)pent-2-en-1-yl)-13-methyl-6,7,8,9,11,12,13,14,15,16-decahydro-17H-cyclopenta[a]phenanthren-17-one (^{1}H NMR, $CDCl_3$, 400 MHz)

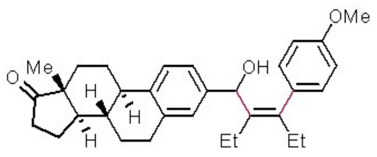

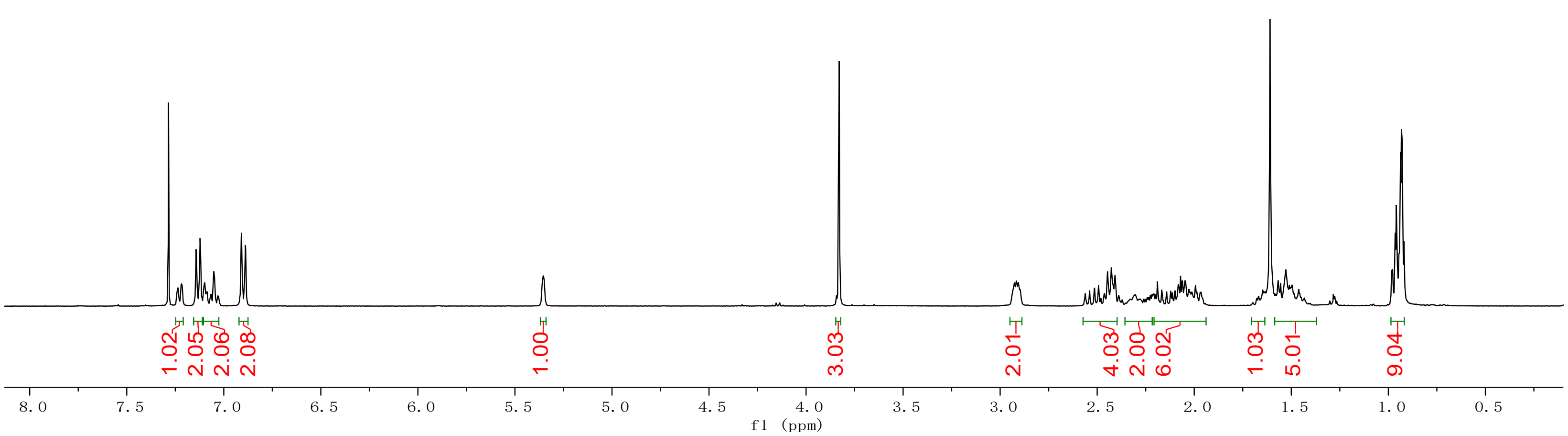

63: (8S,9R,13R,14R)-3-((Z)-2-ethyl-1-hydroxy-3-(4-methoxyphenyl)pent-2-en-1-yl)-13-methyl-6,7,8,9,11,12,13,14,15,16-decahydro-17H-cyclopenta[a]phenanthren-17-one (^{13}C NMR, $CDCl_3$, 100 MHz)

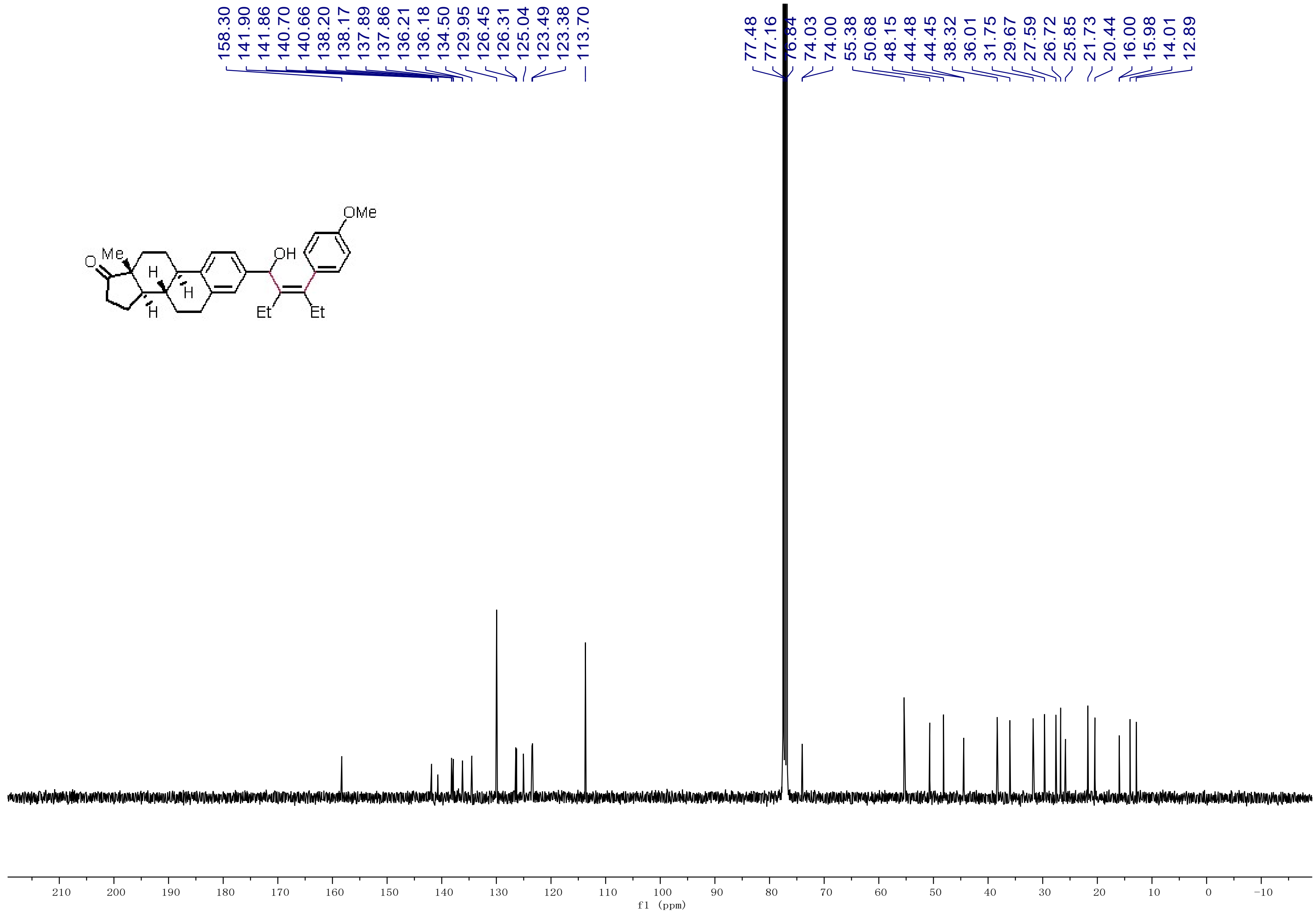

64: (8S,9R,13R,14R)-3-((Z)-2-butyl-1-hydroxy-3-(4-methoxyphenyl)hept-2-en-1-yl)-13-methyl-6,7,8,9,11,12,13,14,15,16-decahydro-17H-cyclopenta[a]phenanthren-17-one (^{1}H NMR, $CDCl_3$, 400 MHz)

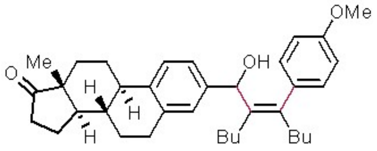

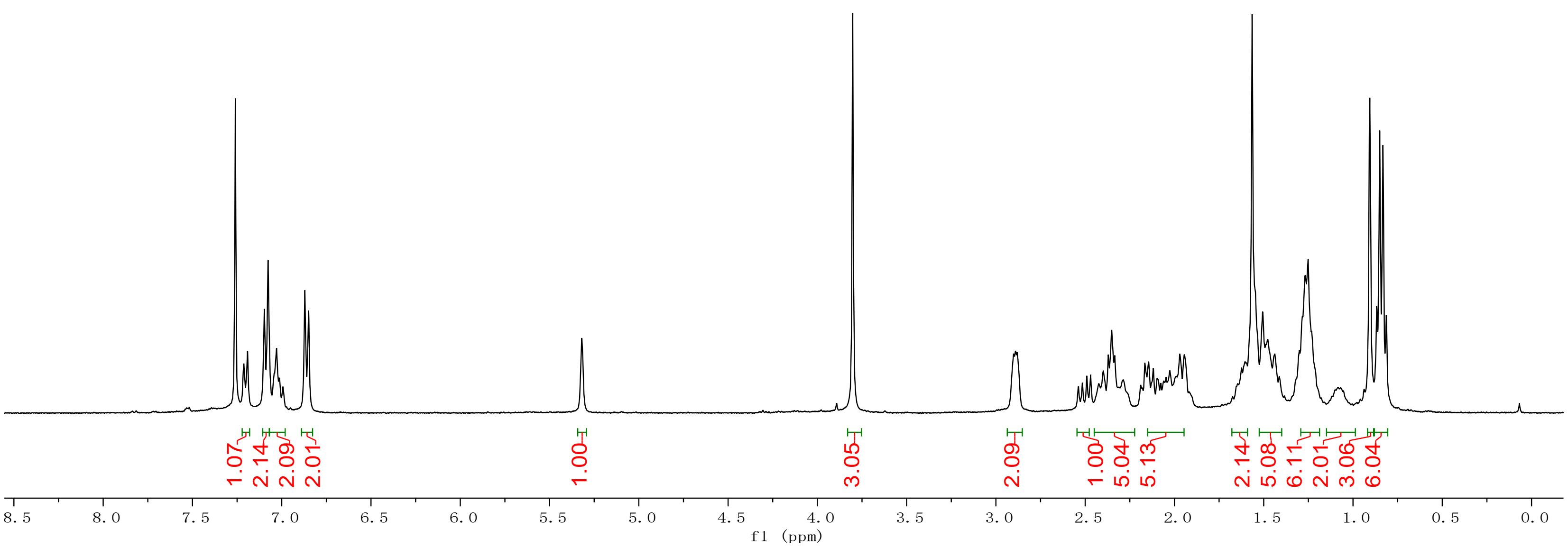

64: (8S,9R,13R,14R)-3-((Z)-2-butyl-1-hydroxy-3-(4-methoxyphenyl)hept-2-en-1-yl)-13-methyl-6,7,8,9,11,12,13,14,15,16-decahydro-17H-cyclopenta[a]phenanthren-17-one (^{13}C NMR, $CDCl_3$, 100 MHz)

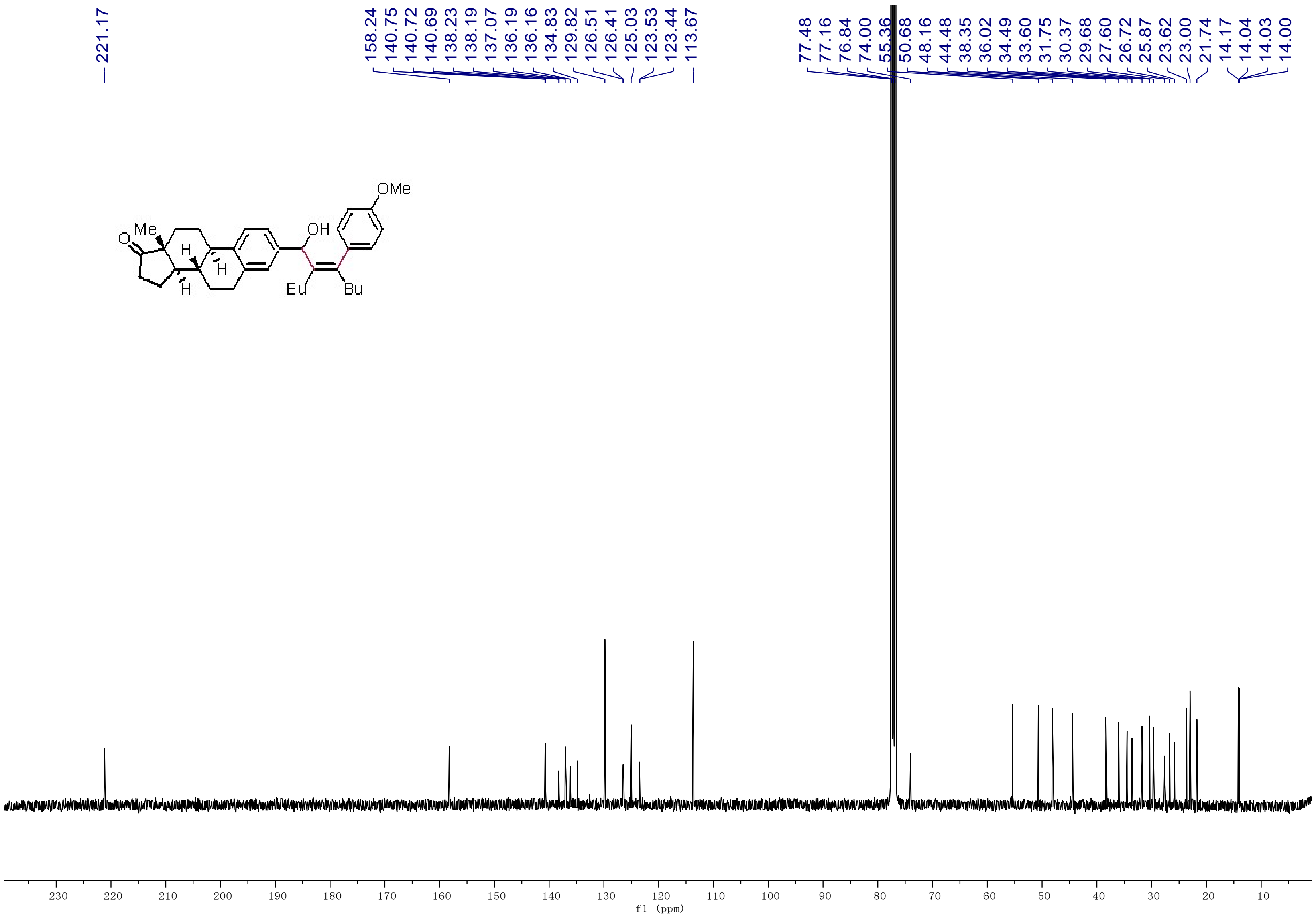

65: (*Z*)-2-butyl-3-(4-methoxyphenyl)-1-(o-tolyl)hept-2-en-1-ol (^{1}H NMR, $CDCl_3$, 400 MHz)

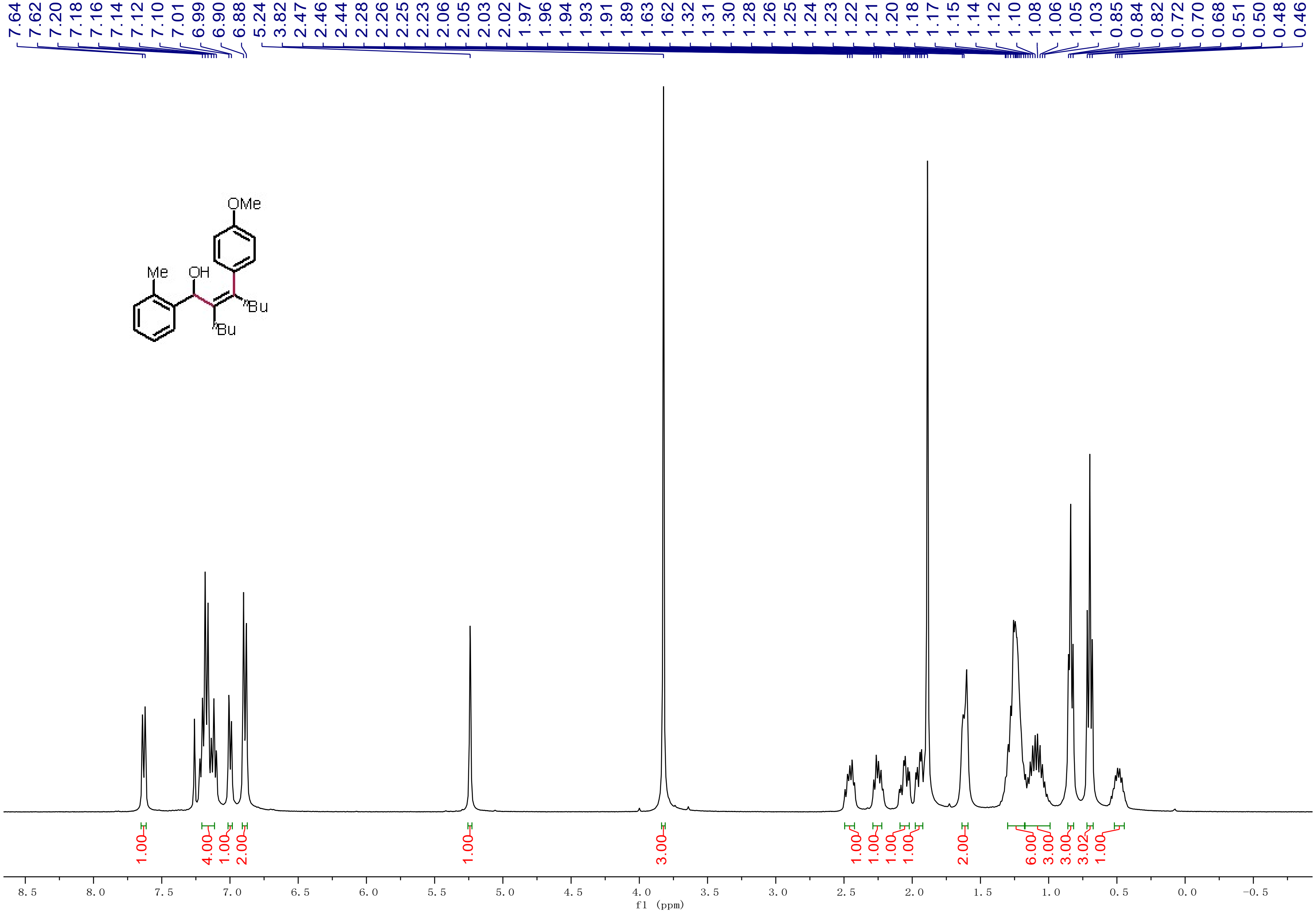

65: (*Z*)-2-butyl-3-(4-methoxyphenyl)-1-(o-tolyl)hept-2-en-1-ol (^{13}C NMR, $CDCl_3$, 100 MHz)

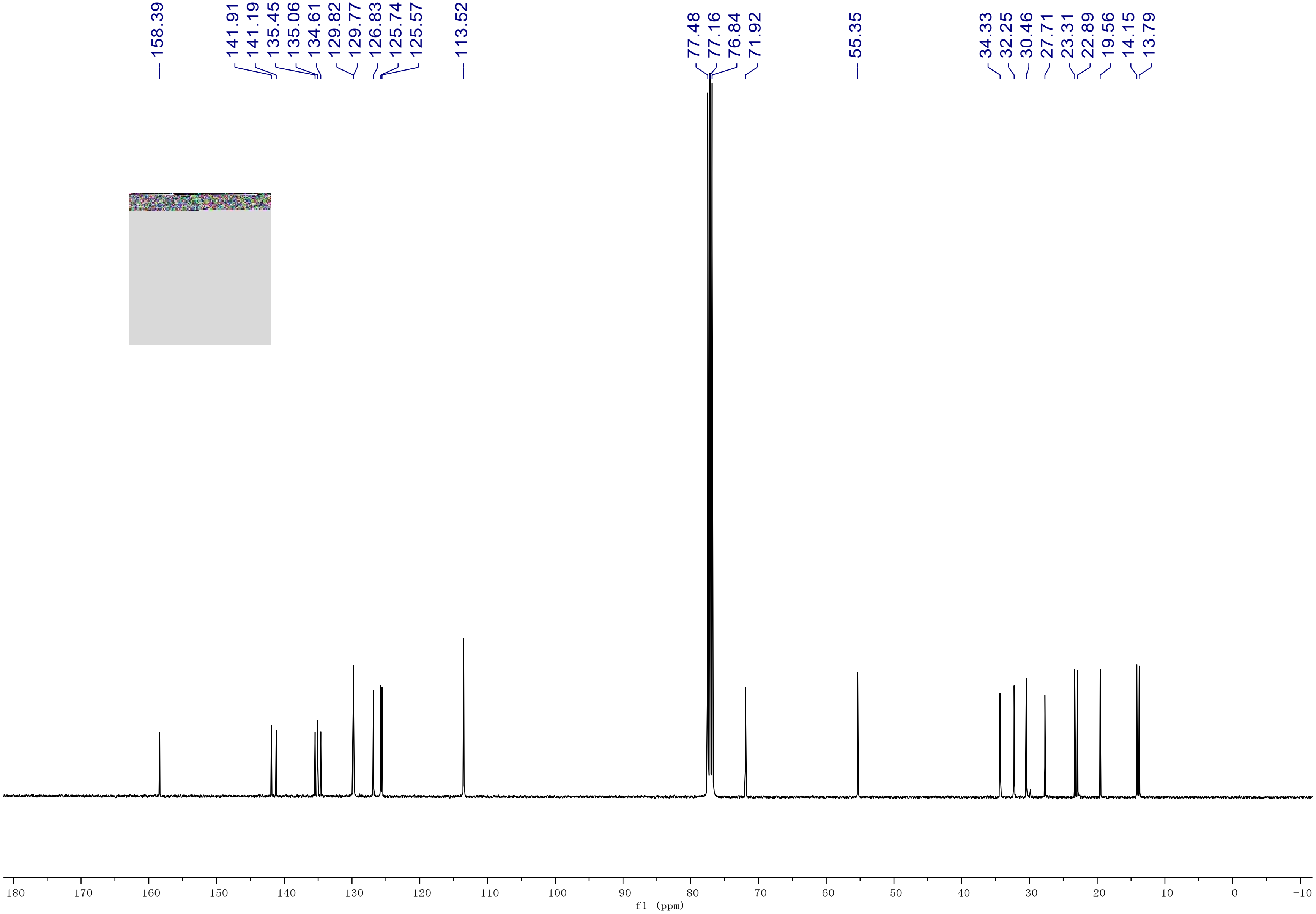

66: (*Z*)-3-(4-(benzyloxy)phenyl)-2-butyl-1-(2-methoxyphenyl)hept-2-en-1-ol (^{1}H NMR, $CDCl_3$, 400 MHz)

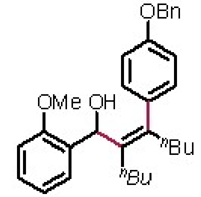

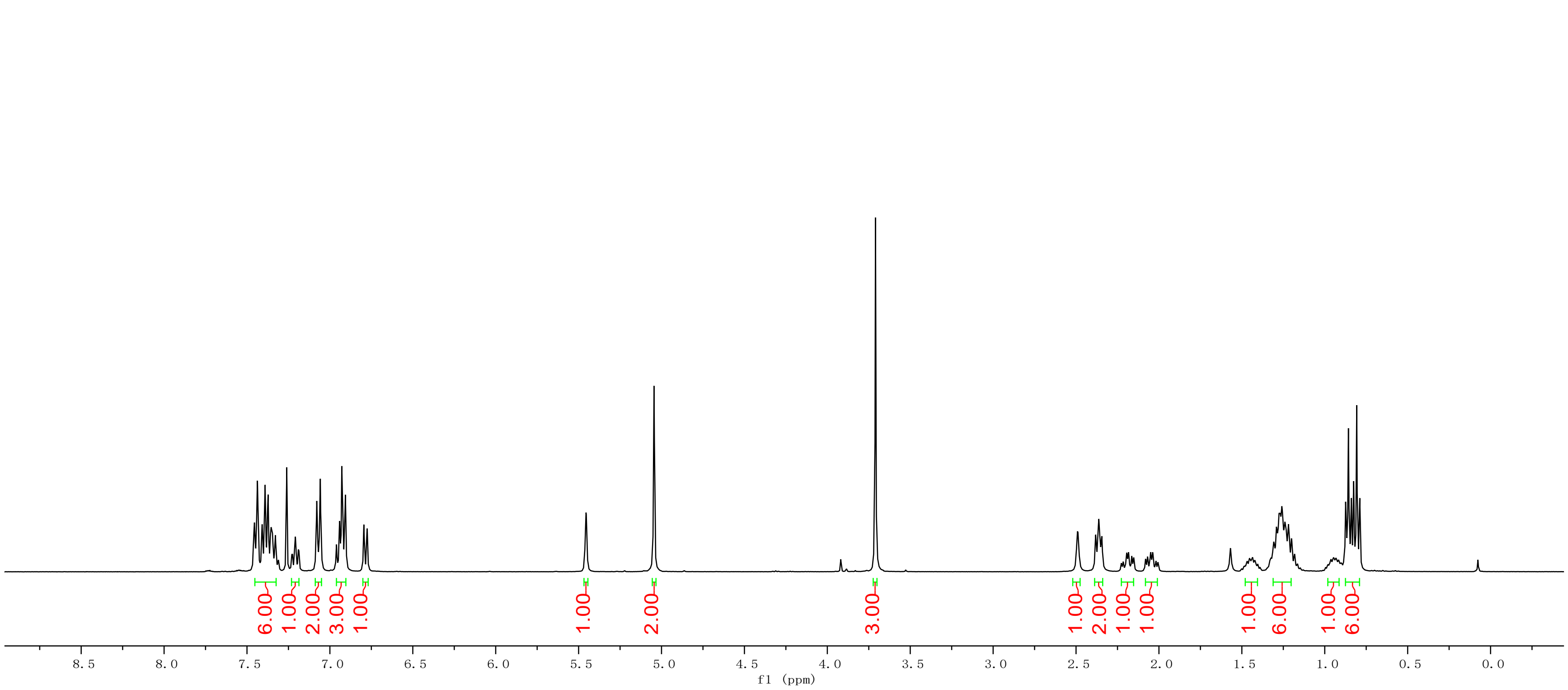

66: (*Z*)-3-(4-(benzyloxy)phenyl)-2-butyl-1-(2-methoxyphenyl)hept-2-en-1-ol (^{13}C NMR, $CDCl_3$, 100 MHz)

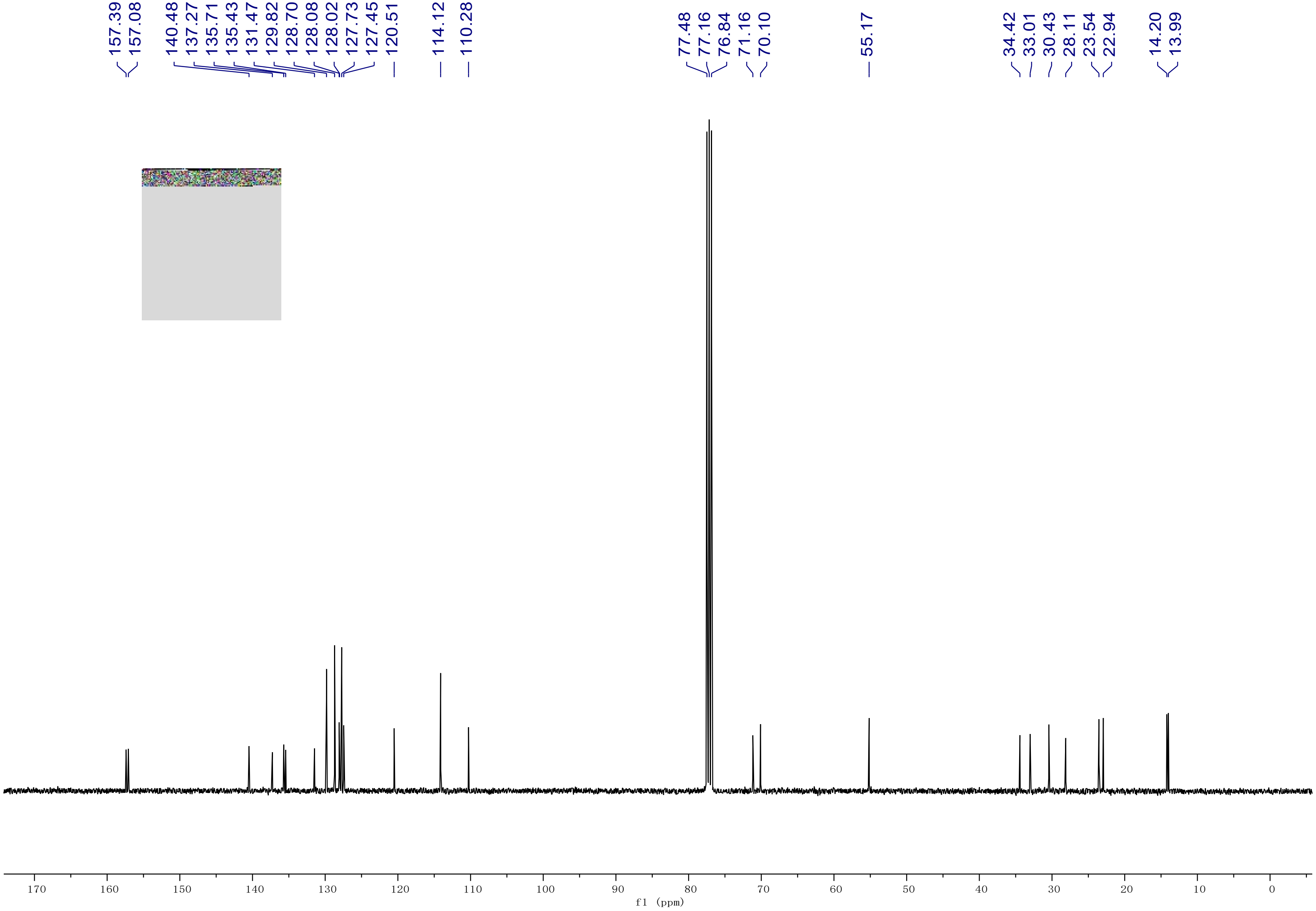

67: (*Z*)-2-butyl-1-(2-methoxyphenyl)-3-(4-methoxyphenyl)hept-2-en-1-ol (^{1}H NMR, $CDCl_3$, 400 MHz)

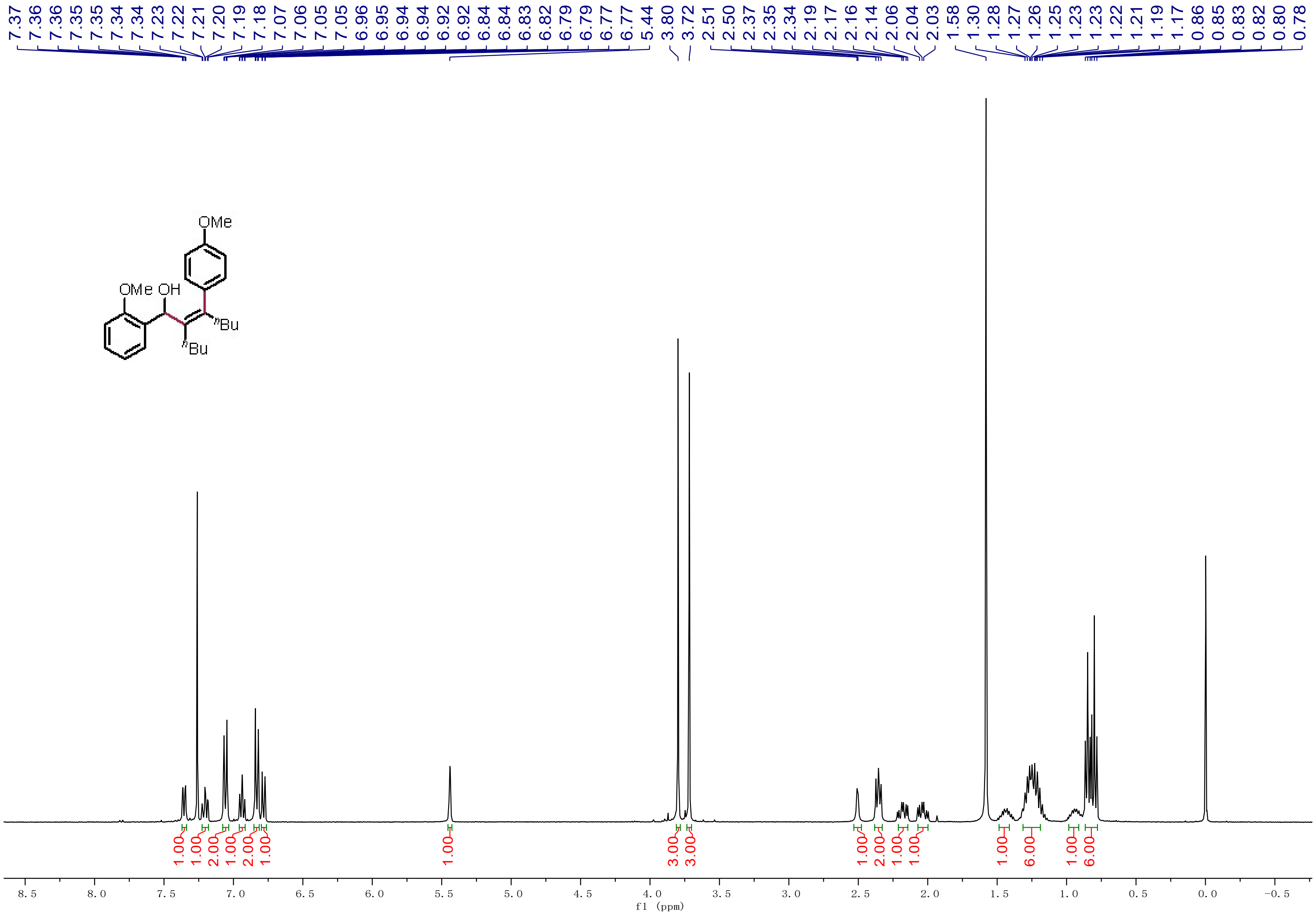

67: (*Z*)-2-butyl-1-(2-methoxyphenyl)-3-(4-methoxyphenyl)hept-2-en-1-ol (^{13}C NMR, $CDCl_3$, 100 MHz)

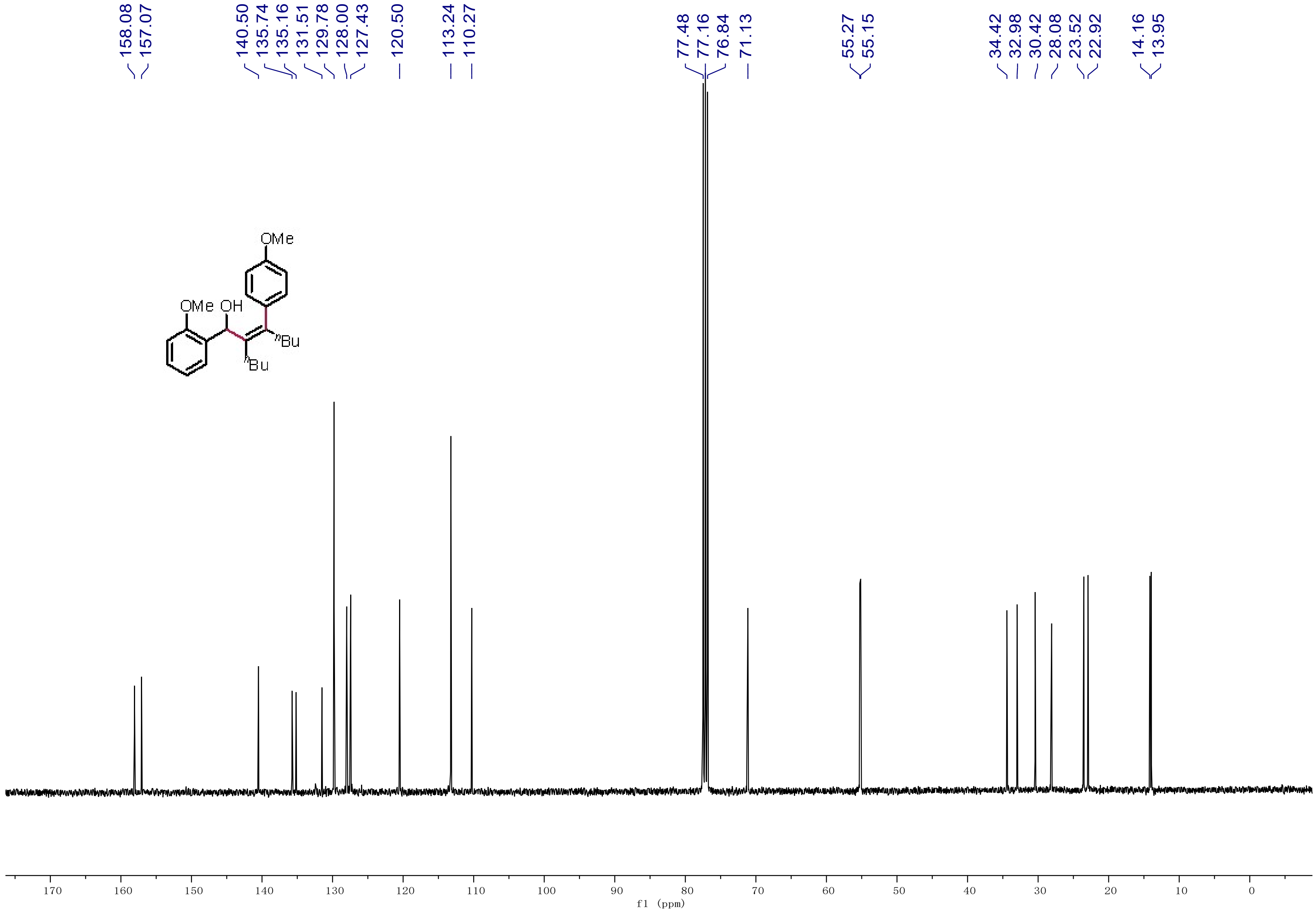

68: (*Z*)-3-(4-(benzyloxy)phenyl)-2-propyl-1-(4-(trifluoromethyl)phenyl)hex-2-en-1-ol (^{1}H NMR, $CDCl_3$, 400 MHz)

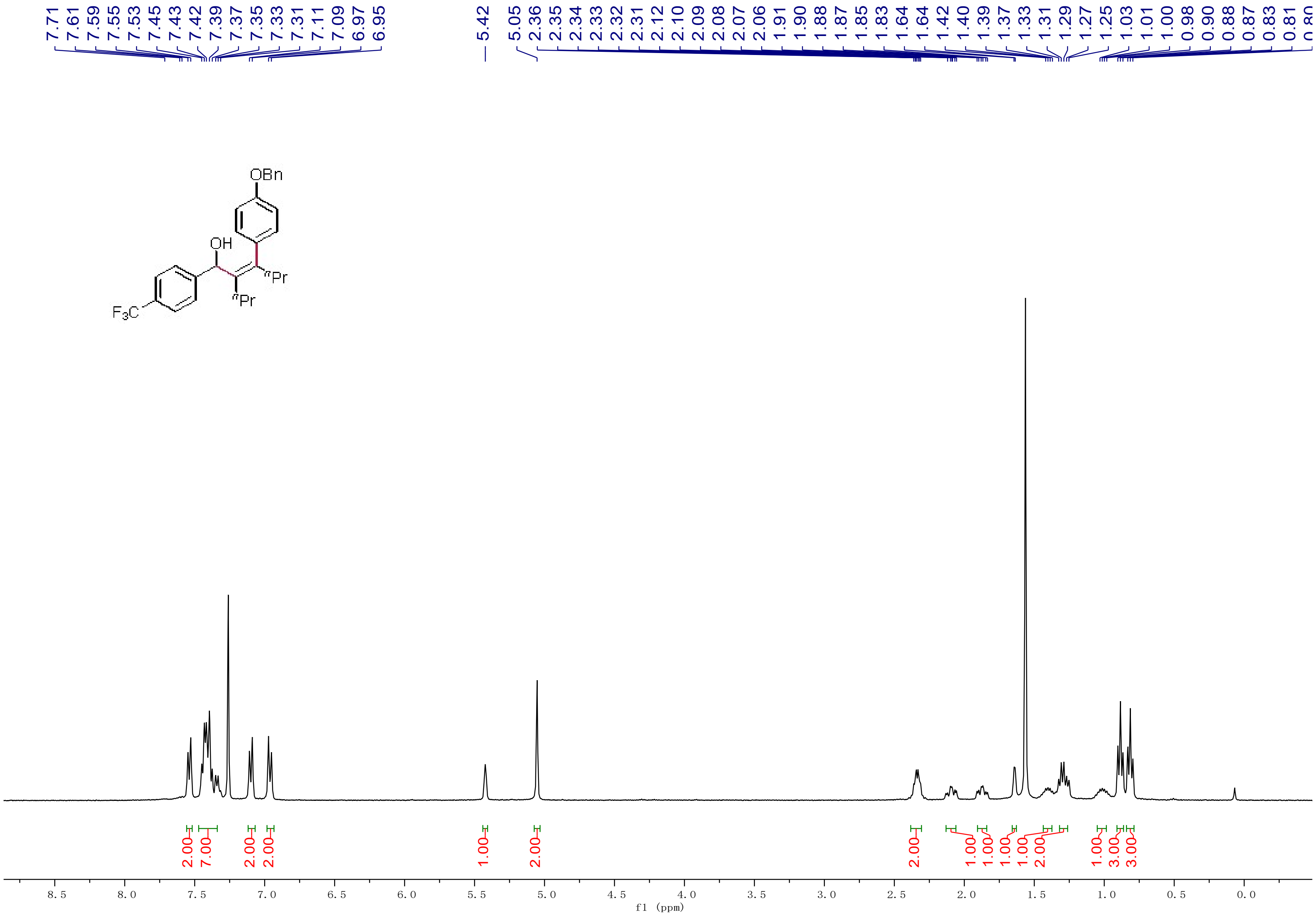

68: (*Z*)-3-(4-(benzyloxy)phenyl)-2-propyl-1-(4-(trifluoromethyl)phenyl)hex-2-en-1-ol (^{13}C NMR, $CDCl_3$, 100 MHz)

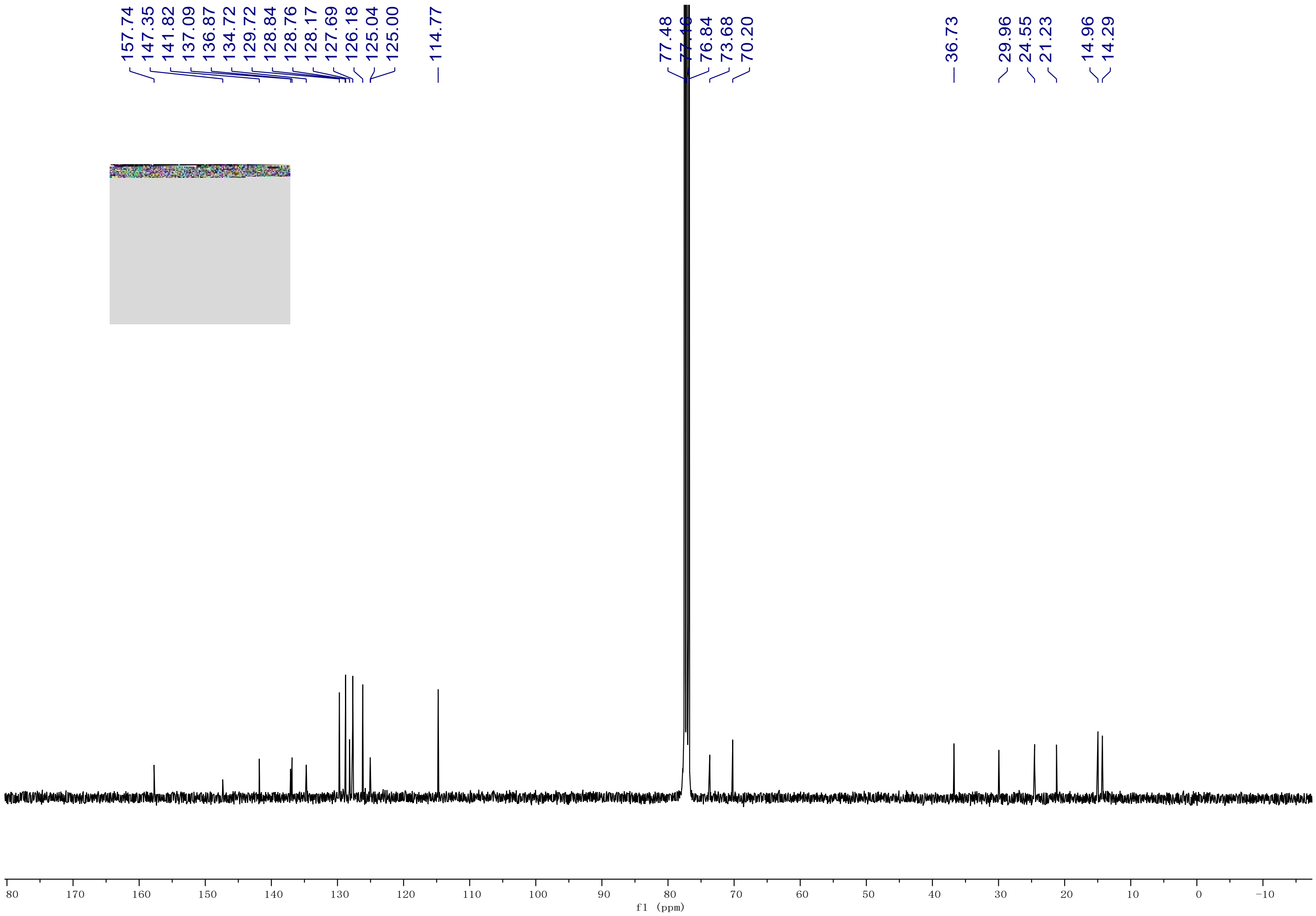

Supplement: SC-014-D2SC05894D-s001 [file SC-014-D2SC05894D-s001.pdf]
